# A poised fragment library enables rapid synthetic expansion yielding the first reported inhibitors of PHIP(2), an atypical bromodomain†
†Electronic supplementary information (ESI) available. See DOI: 10.1039/c5sc03115j


**DOI:** 10.1039/c5sc03115j

**Published:** 2015-12-22

**Authors:** Oakley B. Cox, Tobias Krojer, Patrick Collins, Octovia Monteiro, Romain Talon, Anthony Bradley, Oleg Fedorov, Jahangir Amin, Brian D. Marsden, John Spencer, Frank von Delft, Paul E. Brennan

**Affiliations:** a Structural Genomics Consortium (SGC) , University of Oxford , Oxford OX3 7DQ , UK; b Target Discovery Institute (TDI) , Nuffield Department of Medicine , University of Oxford , Oxford OX3 7FZ , UK . Email: paul.brennan@sgc.ox.ac.uk; c Diamond Light Source (DLS) , Harwell Science and Innovation Campus , Didcot , OX11 0DE , UK . Email: frank.von-delft@diamond.ac.uk; d Department of Chemistry , School of Life Sciences , University of Sussex , Brighton , BN1 9QJ , UK; e Kennedy Institute of Rheumatology , Nuffield Department of Orthopaedics , Rheumatology and Musculoskeletal Sciences , University of Oxford , Roosevelt Drive, Headington , Oxford OX3 7FY , UK; f Department of Biochemistry , University of Johannesburg , Aukland Park 2006 , South Africa

## Abstract

Research into the chemical biology of bromodomains has been driven by the development of acetyl-lysine mimetics. The ligands are typically anchored by binding to a highly conserved asparagine residue. Atypical bromodomains, for which the asparagine is mutated, have thus far proven elusive targets, including PHIP(2) whose parent protein, PHIP, has been linked to disease progression in diabetes and cancers. The PHIP(2) binding site contains a threonine in place of asparagine, and solution screening have yielded no convincing hits. We have overcome this hurdle by combining the sensitivity of X-ray crystallography, used as the primary fragment screen, with a strategy for rapid follow-up synthesis using a chemically-poised fragment library, which allows hits to be readily modified by parallel chemistry both peripherally and in the core. Our approach yielded the first reported hit compounds of PHIP(2) with measurable IC_50_ values by an AlphaScreen competition assay. The follow-up libraries of four poised fragment hits improved potency into the sub-mM range while showing good ligand efficiency and detailed structural data.

## Abstract 2

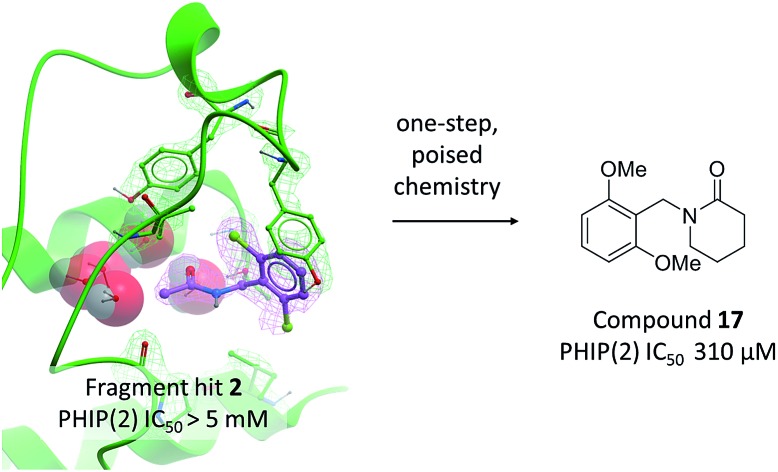
High concentration crystal soaking of poised fragments and one-step elaboration identified compound **17** as an inhibitor of the PHIP(2) bromodomain.

## Introduction

**Fig. 1:**
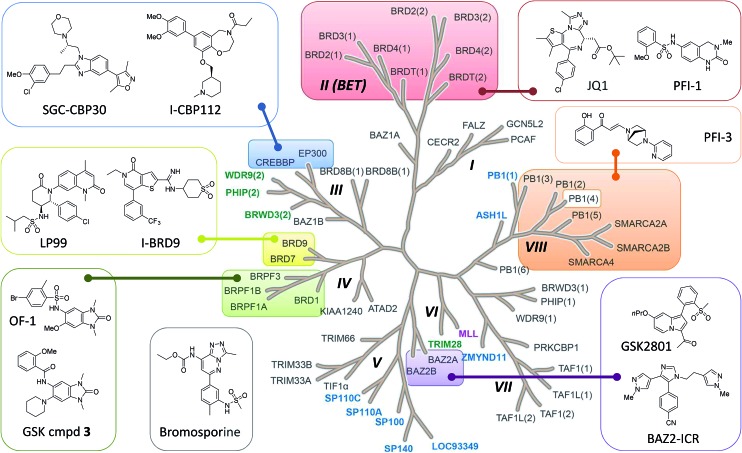
Bromodomain family tree with sub-families (I–VIII) as defined by Filippakopoulos *et al.*^1^ Brds for which SGC Chemical Probes have been released are highlighted and the structures depicted: SGC-CBP30,^11^ I-CBP112, (+)-JQ1,^3^ PFI-1,^7^ PFI-3, BAZ2-ICR,^15^ GSK cmpd 3, OF-1, LP99,^12^ I-BRD9 and the pan-Brd inhibitor bromosporine.^16^ Atypical bromodomains are highlighted for which the conserved asparagine residue in the KAc binding site is replaced by tyrosine (blue), threonine (green) or aspartic acid (purple).

Bromodomains are acetyl-lysine (KAc) reader domains involved in the modulation of gene expression.^1^ The druggability of bromodomains have made them attractive targets for the treatment of diseases such as cancer and inflammation, leading to the development of a range of chemical probes for the investigation of bromodomain (Brd) biology (Fig. 1).^2^ In 2010, JQ-1 and I-BET were reported as potent inhibitors of the BET bromodomains (subfamily II)^3^,^4^ and subsequent academic and industrial research has focused on targeting the BET bromodomains.^5^–^8^ However, recent publications indicated other subfamilies of the Brd family tree could be targeted by small molecule inhibitors,^9^,^10^ including CBP/p300 (subfamily III),^11^ BRD7/9 (IV),^12^,^13^ BAZ2A/B (V),^14^,^15^ CECR2 (I), BRPF1/2/3 (IV) and SMARCA2/4/PB1 (VIII), as well as the pan-Brd inhibitor, bromosporine (Fig. 1).^16^

Most Brd inhibitors are anchored by a highly conserved asparagine (Asn) residue and a network of four water molecules in the KAc binding pocket. Together the Asn and first water in the chain of four donate two H-bonds to a pair of H-bond acceptors on the inhibitor. The importance of the conserved Asn residue in inhibitor binding reflects its critical role in KAc recognition of the natural ligand (ESI Fig. S1A†).^1^ However, only forty-eight of the sixty-one known human Brds (79%) Brds have an Asn residue in the KAc binding pocket. The remaining thirteen Brds (21%) have a tyrosine, threonine or aspartic acid in place of the asparagine (Fig. 1 and ESI Fig. S1B†). To date, no inhibitors of these atypical, non-asparagine bromodomains have been reported.

## PHIP(2), an atypical bromodomain

**Fig. 2:**
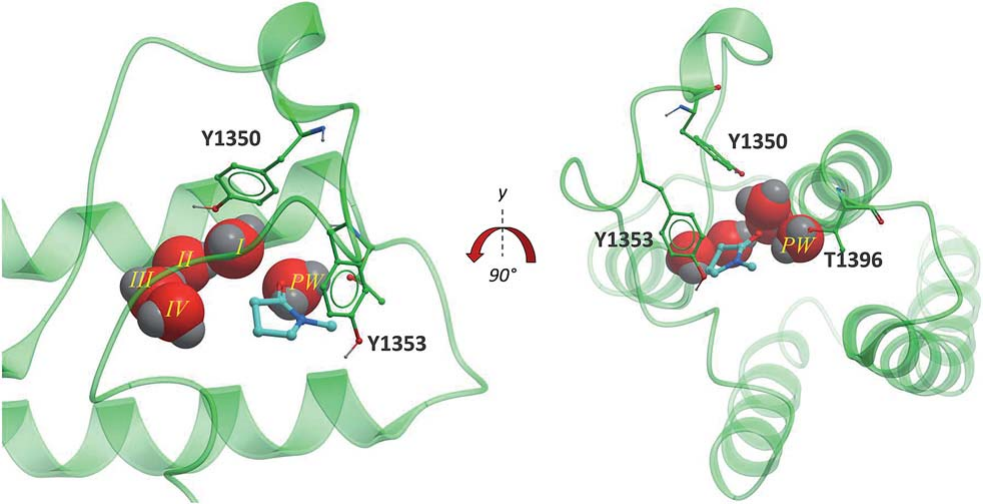
PHIP(2) (green sticks and ribbons) in complex with NMP (cyan sticks). The substitution of an Asn for the smaller Thr allows an extra water molecule, the PHIP water (red CPK, PW) to fill the space between T1396 and the usual four Brd waters (red CPK I–IV) (PDB ID: 3MB3).^1^

The second bromodomain of the pleckstrin homology domain-interacting protein (PHIP(2)) is one of the atypical bromodomains. PHIP is believed to mediate the activity of insulin-receptor (IRS) proteins,^17^ and has been identified as a possible marker in melanoma prognosis.^18^ More recently, PHIP has been observed to be overexpressed in metastatic melanomas.^17^ The PHIP protein contains two bromodomains, PHIP(1) and PHIP(2). The first bromodomain, from family VII of the bromodomain phylogenetic tree, has no reported crystal or NMR structures. However, in 2010 the crystal structure of the second PHIP bromodomain was deposited in the Protein Data Bank (PDB) (NMP, PDB ID ; 3MB3) (Fig. 2), bound to *N*-methyl pyrrolidone but in a flipped conformation compared to typical Brds, such as CREBBP (PDB ID ; 3P1D).^1^

PHIP(2) belongs to the bromodomain subfamily III alongside CREBBP (Fig. 1).^1^ The atypical binding site of PHIP(2) is characterised by a threonine (Thr) residue (T1396) in place of the conserved asparagine (Fig. 2). Because the Thr side chain is one atom shorter than Asn, there is space for an additional water molecule, dubbed the ‘PHIP water’, to mediate an interaction between T1396 and the typical water network at the back of the binding pocket. The water-network is otherwise comparable to the network observed in most Brd binding sites, consisting of four water molecules and anchored by a tyrosine residue (Y1353). Despite being identified as a druggable Brd,^19^ to date no small molecule inhibitors of PHIP(2) have been reported. An unpublished screening campaign by the Structural Genomics Consortium (SGC) did not yield any hits from a library of approximately seven thousand Brd-focused compounds.

## Fragment based drug discovery by X-ray crystallography

Fragment based drug discovery (FBDD) has become an increasingly important tool for finding hit compounds for difficult targets.^20^ The technique utilises smaller than drug-like compounds to identify low potency, high quality leads. Libraries containing hundreds to thousands of compounds achieve similar coverage of chemical space as the millions required for traditional high throughput screening (HTS) campaigns.^21^ As a result, FBDD is considerably more affordable as a hit-finding method and has enjoyed widespread success in both academia and industry.^22^

Fragment screening has been especially effective in discovering Brd inhibitors using both focused and diverse libraries.^23^,^24^ Fragments have been optimized into more potent chemical probes but in all cases the Brds targeted contained the key Asn residue to anchor an acetyl lysine mimetic fragment.^11^,^25^

A major challenge of fragment screening is that, although fragment sized compounds make high quality interactions with their targets and bind with high ligand efficiency, overall binding affinity is typically weak due to their small size.^26^ Therefore the techniques used to detect fragment binding must be sensitive on the μM to mM scale. Biophysical and biochemical solution-based techniques are convenient and widely used but drawbacks include insufficient sensitivity, high rates of false positives and screening concentrations limited to low mM.^27^ In contrast, NMR and X-ray crystallography are ideal for detecting weak binding by yielding direct structural information, although historically both techniques have been labour intensive and low-throughput. However, in recent years X-ray crystallography has seen an order-of-magnitude speed-up thanks to robotics, improved algorithms and detectors and technical advances at synchrotron facilities.^28^,^29^ Moreover, at beamline I04-1 at Diamond Light Source (DLS),^30^ other recent developments around soaking, harvesting and data analysis have further reduced the effort to such an extent that a 1000-compound fragment screen by crystallography can be completed within a week. As a consequence, X-ray crystallography is now a viable primary screen for academic and industrial researchers.^31^

In particular, this makes it realistic to screen by soaking single compounds per crystal, rather than cocktails, so that concentrations >100 mM can be achieved, one hundred to one thousand times higher than possible in solution-based techniques, with accordingly increased sensitivity.^31^

The very weak hits identified by X-ray screening pose a practical problem, in that they are in general too weak to be verified by orthogonal solution assays; yet such information is a common requirement in medicinal chemistry operations, especially if the crystallographic evidence is ambiguous. Optimising weak compounds by traditional SAR is equally undermined without reliable assay readout in the range of the compounds' binding affinity. In any case, progressing weak compounds to potency in general requires changes not only to the periphery, where conventional SAR operates, but also to the core of the compound.

To overcome the problem of optimising weak compounds discovered by high concentration fragment soaking, we report the design and use of a poised fragment screening library to identify hits and the parallel synthesis of analogues to deliver inhibitors with measurable activity. Furthermore, we demonstrate how the method is effective on the previously intractable target, PHIP(2).

## Results and discussion

### Poised fragments

**Fig. 3:**
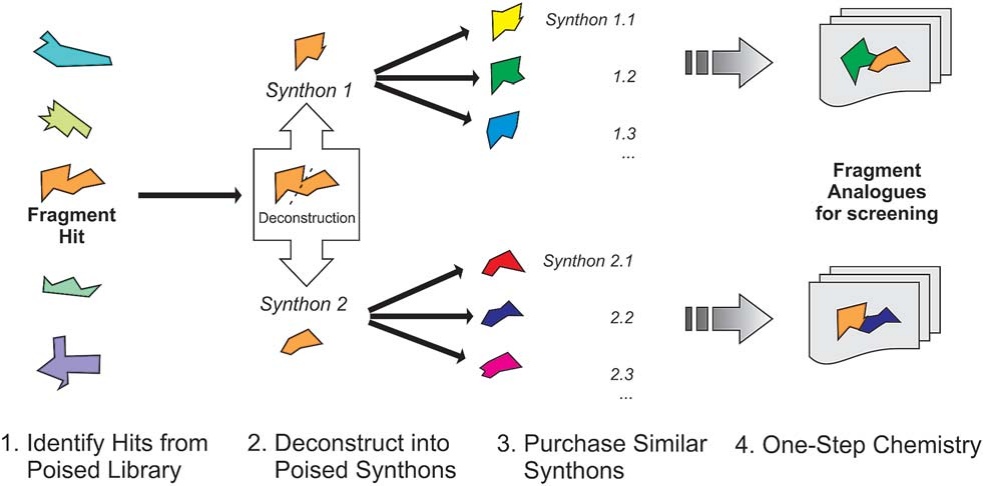
Schematic depicting how a poised fragment library can be used to rapidly synthesise a follow-up library of analogues. If the orange fragment is identified as a hit, a library of similar compounds can be rapidly constructed using parallel synthesis with commercial analogues of synthons 1 and 2.

We define a poised fragment as a fragment synthesised from a robust and general synthetic reaction such that rapid elaboration of the fragment hit into a library of analogues can be done using parallel chemistry. Identification of a poised bond (or bonds) in a fragment allows the compound to be deconstructed into at least two synthons (Fig. 3). As a simple example, an amide bond can be deconstructed into an acid chloride and an amine. Upon discovery of a poised amide hit, purchase of similar synthons to those found in the hit allows the synthesis of a library of analogous amides, which can be soaked in crystals just days after the initial hit has been obtained. The result is detailed structural information of the ligand binding site and, potentially, an improvement in binding activity large enough to allow measurement using a biophysical or biochemical assay. Poised chemistry requires reliable, robust reactions which tolerate a range of substrates and can be performed using a wide range of commercially available starting materials. The reaction products should be formed in high yield and contain drug-like functionalities with no known toxicological moieties.

**Fig. 4:**
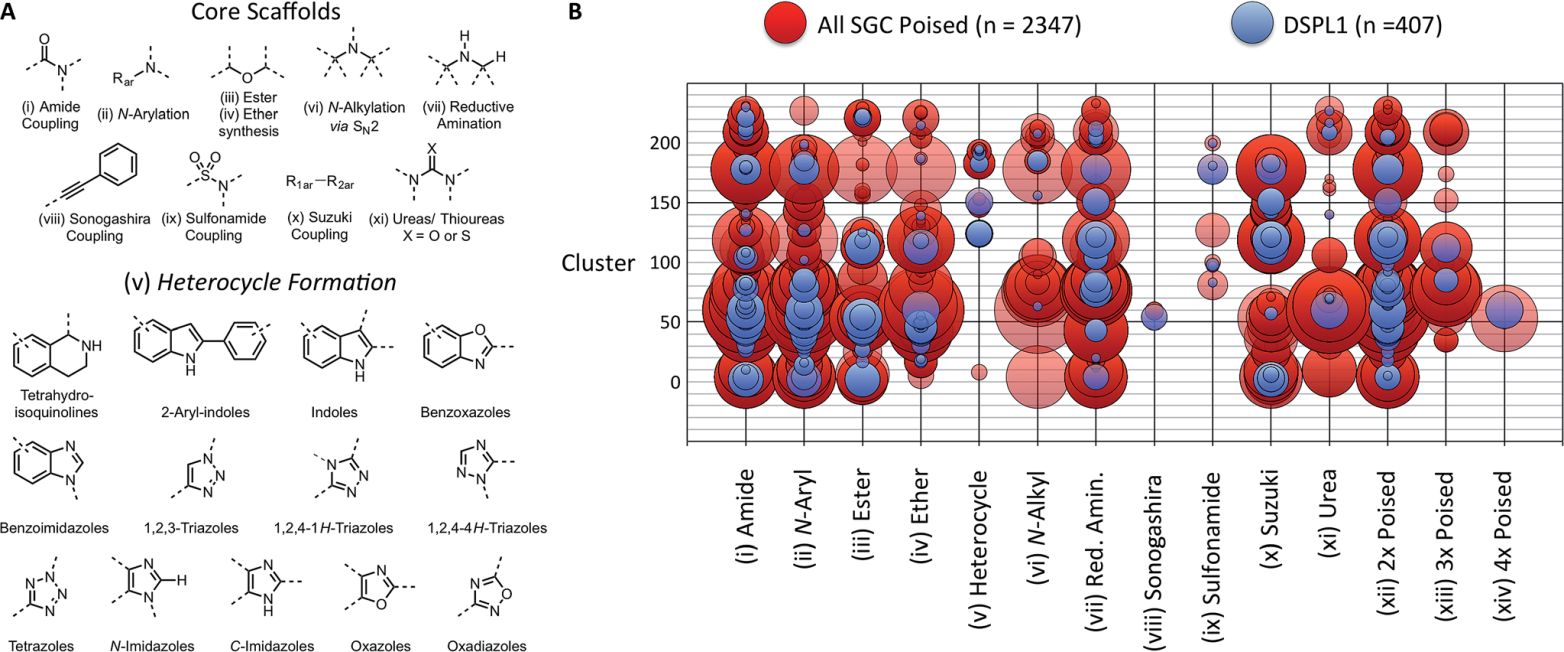
Design of DSPL1 (A) scaffolds used to identify poised fragments. Core scaffolds based upon the most commonly used medicinal chemistry reactions as described by Roughley and Jordan.^32^ Heterocycle scaffolds based on reactions described by Hartenfeller *et al.*^37^ (B) Poised reaction motif *vs.* cluster. Circle size represents the number of compounds in each group. 407 compounds for DSPL1 were selected from all SGC poised fragments based on diversity and poised reaction motif.

In 2011, Roughley and Jordan published an analysis of the most commonly used reactions in drug discovery.^32^ The authors propose the reactions are regularly used because experienced medicinal chemists know they provide reliable methods to synthesise drug-like molecules. The top ten reactions identified by the analysis, including amide coupling, reductive amination and Suzuki-type aryl–aryl coupling, can be performed using standardised procedures for a wide range of substrates. The starting materials for each reaction contain common functionalities which are well represented in commercial supplier space and are compatible with this diverse range of commercial compounds. The ten reactions were selected to create poised scaffolds with which to perform substructure searches. The poised fragment chemical space was further augmented with twelve heterocycle forming reactions as defined by Hartenfeller *et al.*^33^ and an oxazole formation developed in our own lab.^34^,^35^ The complete list of poised scaffolds is depicted in Fig. 4A.

### Design of poised fragment libraries

To construct an initial poised fragment library, the Diamond and SGC Poised Fragment Library 1.0 (DSPL1), the large collection of SGC fragments assembled in-house and from collaborator collections (11 677 in total), was analysed for the presence of poised substructures and prevalent synthons. Of the eleven thousand SGC fragments, 2347 could be considered poised by our definition. As screening the entire set of >2000 poised SGC fragments would not have been possible and as some of the compounds were very similar to each other, a subset of 406 compounds was selected for DSPL1 ensuring diversity of chemotype and poised classification. All compounds were clustered by fingerprints using the default method in MolSoft's ICM to give 233 clusters. The compounds in DPSL1 were selected based on diversity of structure and diversity of poised motif (Fig. 4).

Encouraged by the success of soaking DSPL1 in PHIP(2) (see below), a second generation poised library was designed (DSPL2). DSPL2 was designed using similar methodology as DSPL1 but was assembled entirely from commercial fragments to ensure that anyone could purchase and use the library. The ZINC fragment-like library was downloaded^36^ and filtered based on vendor ID for suppliers from which we had routinely sourced compounds, yielding ∼192k compounds. Filters were applied to the compound library to ensure drug-like characteristics (ESI Fig. S2†). From the resulting library of 41 271 compounds, 28 438 (68%) were found to be poised by our definition. While initially surprising that such a large number of commercial fragments could be synthesised by only twenty-one different chemical reactions, we believe this reflects the very limited coverage of potential synthetic compounds by commercial vendors. Further filters were applied to ensure each compound is compatible with the reaction by which it was found to be poised. For example an amine substituted amide would be removed from the library as an asymmetric diamine synthons could give a mixture of amides if used to synthesise a poised fragment. Compounds without commercially available starting materials were also removed.

In theory, the resulting library of 10 448 compounds covers the whole of commercially accessible poised fragment space using our chosen reactions. The library is dominated by amides, with 55.1% of fragments containing a poised amide bond; ether bonds (18.2%) and reductive amination products (10.2%) were also heavily represented. The least represented chemistries were Sonogashira products (0.2%) and sulfonamides (2.0%) (ESI Fig. S3†). Unfortunately, poised heterocyclic reactions were observed in just 2.2% of the compounds, despite *thirteen* reactions (only eight of which were found to be present) being used to identify such fragments and the importance of such scaffolds in drugs and chemical tools.^38^ The finding demonstrates the bias of commercially available compounds towards the most commonly used reactions identified by Roughley and Jordan.^32^

Finally, the USRCAT algorithm was used to ensure chemical diversity was maintained upon selecting 1000 compounds for DSPL2 from the total of >10 000.^39^ USRCAT was selected because it was believed the additional conformation information makes it a superior measure of diversity for library design in comparison to traditional chemical diversity measures (MACCS, Morgan fingerprints, *etc.*). Although DSPL1 and 2 do not contain the exact same compounds, they were constructed using similar principles and occupy similar chemical space (ESI Fig. S4†). The full identities of DSPL1 and DSPL2 can be downloaded as sdf files from the ESI.†

### Soaking PHIP(2) with DSPL1

To test the utility of the poised fragment library methodology, the PHIP(2) Brd was screened using high concentration crystal soaking. The PHIP(2) Brd was crystallised in its apo-form to generate enough crystals to screen DSPL1.^40^

Due to the relatively potent binding of DMSO to Brds,^41^ compounds were dissolved in ethylene glycol at a nominal concentration of 400 mM. The supernatant of compounds which did not fully dissolve was used with the assumption it was a saturated solution. The solutions were soaked into protein crystals in crystal buffer in a 1 : 1 volume ratio to give an approximate final compound concentration of 200 mM. All 406 poised fragments in DSPL1 were soaked into PHIP(2) Brd crystals and when the structures were solved, compounds **1–4** were found to have bound to the protein (1.2% hit rate).

**Fig. 5:**
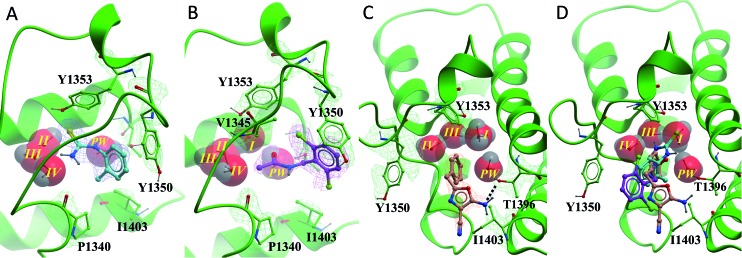
PHIP(2) (green sticks, ribbons and mesh (2Fo-Fc)) in complex with fragment hits. (A) Thiourea **1** (cyan sticks and mesh (2Fo-Fc)) displaces waters *I*. (B) *N*-Benzylacetamide **2** (purple sticks and mesh (2Fo-Fc)) binds with all water molecules intact and forms an edge–face interaction with Y1350. (C) Oxazole **3** (cyan sticks and mesh (2Fo-Fc)) forms H-bonds to PW (black dashed line). PHIP(2) orientation has been changed for clarity. (D) Overlay of compounds **1–3** in PHIP(2). In (C) and (D) water II is hidden behind water *I*.

All of the poised fragment hits **1–4** bound to PHIP(2) Brd in the KAc recognition pocket (Fig. 5). The thiourea sulfur of compound **1** displaces water *I* at the back of the binding pocket allowing the formation of hydrogen bonds with Y1353 and water *II*. The secondary thiourea nitrogen forms an H-bond to T1396 *via* the structural PHIP water (*PW*). The primary thiourea nitrogen lies in the hydrophilic pocket made by waters *II*, *III* and *IV*. The phenyl ring of compound **1** makes an edge–face aromatic interaction with Y1350, which is shifted significantly towards the ligand compared to the NMP-bound conformation (Fig. 2). The *ortho*-methyl substituent on the phenyl ring makes additional interactions with the hydrophobic residues I1403 and P1340.

Compound **2** binds like a classical acetyl-lysine mimetic (Fig. 5B). The acetyl group lies deep in the binding pocket allowing the methyl group to sit in the hydrophobic pocket created by the four structural waters in a manner analogous to inhibitors of typical Brds and acetyl lysine. The carbonyl oxygen interacts with T1396 through a hydrogen bond mediated by the PHIP water. Like compound **1**, the phenyl ring of compound **2** appears to make an edge–face aromatic interaction with the shifted Y1350 side chain, allowing the 2,6-dichloro substituents to occupy the hydrophobic pockets formed by V1345 and I1403 above and below the ring.

Oxazoles **3** and **4** share the same binding mode. The primary amine and oxazole oxygen form a donor and acceptor interaction with T1396 (Fig. 5C, compound **4** not shown for clarity). Y1350 forms an H-bond to the oxazole nitrogen. The orientation of the oxazole rings of compounds **3** and **4** is striking, and allows for the respective lipophilic benzyl and isobutyl groups to lie in the methyl-binding pocket created by the four structural waters. Furthermore, the 4-cyano and 4-amino groups of compounds **3** and **4** interact with a neighbouring protein in the crystal lattice. The cyano group acts as an H-bond acceptor with the backbone N–H of D1352, while the amino group forms a salt bridge with the D1352 carboxylate group.

### Rapid synthesis of follow-up libraries

As each of the hit compounds, **1–4**, is a poised fragment, the three distinct series were rapidly populated with analogues *via* parallel, solution-phase synthesis.

#### (a) Thioureas

**Scheme 1:**
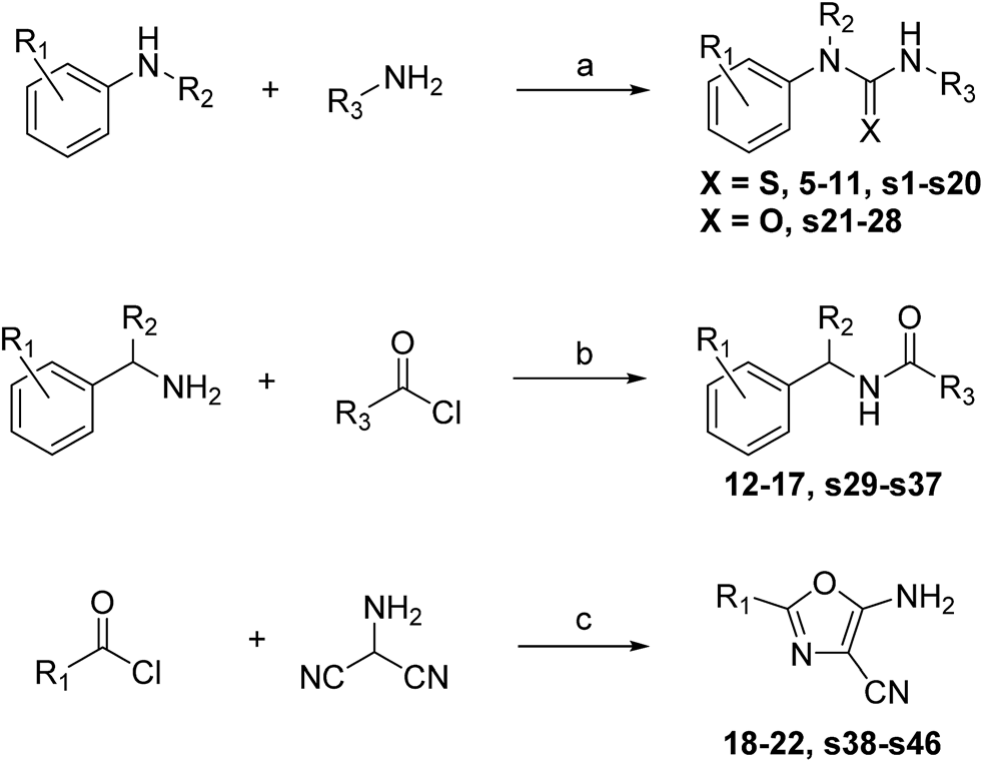
Reagents and conditions: (a) amine 1, thio-CDI, DCM, rt, 30 min, then amine 2, rt, 12 h. (b) Triethylamine, DCM, rt, 3 h. (c) 4-Toluenesulfonic acid, NMP, MW irradiation, 120 °C.

The thioureas were synthesised by the sequential addition of the relevant amines to 1,1′-thiocarbonyldiimidazole (thio-CDI) (Scheme 1). Analysis of the crystal data obtained for compound **1** indicated a lipophilic pocket adjacent to the 3-position of the benzene ring so emphasis was placed on exploring this position. Substitution of the terminal thiourea nitrogen was also proposed in an attempt to displace more of the structural waters at the back of the binding site. In total thirty-five compounds were proposed for synthesis and twenty-nine (83%) were synthesised and purified in moderate to good yield within two weeks (Table 1, compounds **5–11** and ESI Table S1,† compounds **s1–s22**).

**Table 1:** Synthesis and screening of thioureas **1**, **5–11**

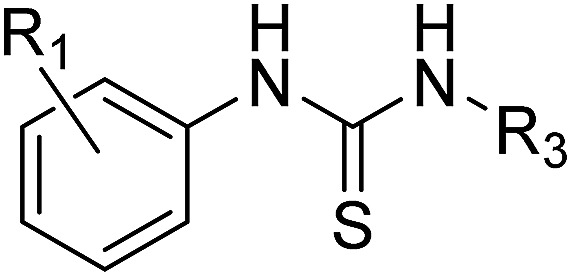						
Cmpd	R_1_	R_3_	Yield^*a*^	X-tal soak^*b*^	PHIP(2) pIC_50_^*d*^	LE
**1**	2-Me	H	66%	Success	3.11 ± 0.06 (2)	0.40
**5**	2-Me-3-OMe	H	71%	Error	2.97 ± 0.18 (2)	0.32
**6**	2,3-Me_2_	H	12%	Success^*c*^	3.78 ± 0.06 (2)	0.45
**7**	2-Me-3-CF_3_	H	33%	NL	3.85 ± 0.21 (2)	0.36
**8**	2-Me-3-Cl	H	26%	Success^*c*^	3.59 ± 0.03 (2)	0.42
**9**	2,6-Me_2_	H	8%	NL	3.32 ± 0.08 (2)	0.39
**10**	2-Me	Me	14%	NL	3.38 ± 0.30 (2)	0.39
**11**	2-Cl	Me	19%	NL	3.89 ± 0.05 (2)	0.45

^*a*^Synthesised using procedure described in Scheme 1. NI: not isolated.

^*b*^NL: no ligand found in model. Error: experiment failure during soaking, at beamline or during data processing or model refinement.

^*c*^Binding pose uncertain as a result of partial occupancy.

^*d*^By AlphaScreen peptide displacement assay.

The thiourea series was tested for inhibition of PHIP(2) ligand binding using an AlphaScreen assay based upon a previously reported method.^41^ Ligand efficiencies (LE) were calculated using the relationship described by Hopkins *et al.* in which LE is taken to be proportional to the IC_50_ contribution per non-hydrogen atom in the ligand.^26^

The original fragment hit **1** had pIC_50_ of 3.11 in the AlphaScreen assay. Addition of a substituent at the 3-position of the phenyl ring to give compounds **5–8** increased potency. The methyl derivative **6** and the trifluoromethyl derivative **7** had improved pIC_50_ ∼ 3.8. The addition of a methyl group at the 6-position of the phenyl ring to give compound **9** slightly increased potency to pIC_50_ 3.32 as did methylation of the primary thiourea nitrogen to give compound **10** (pIC_50_ 3.38). The SAR improvements were not cumulative (see ESI,† compounds **s1–s20**), but replacement of 2-methylphenyl with 2-chlorophenyl on the methylated thiourea, giving compound **11**, improved PHIP(2) activity to pIC_50_ 3.89. The LE of fragment hit **1** was calculated to be 0.40. The value was increases to 0.45 for the most potent thioureas **6** and **11**.

When the thiourea compounds **5–11** were subjected to soaking experiments with PHIP(2) crystals, two of the most potent compounds, **6** and **8**, appeared to bind in the PHIP(2) KAc recognition site (ESI Fig. S5A and B†). The apparent poses are similar to compound **1**, displacing structural water.

#### (b) *N*-Benzyl amides

The *N*-benzyl amides were synthesised using amine derivatisation with acyl chlorides (Scheme 1). Substitution of the benzene ring was proposed as well as extension of the acetyl group with the aim of displacing structural waters. In total twelve analogues were proposed for synthesis and eleven (92%) were synthesised in good to excellent yield within one week (Table 2, compounds **12–17** and ESI Table S2,† compounds **s23–s27**).

**Table 2:** Synthesis and screening of *N*-benzylamides **2**, **12–17**

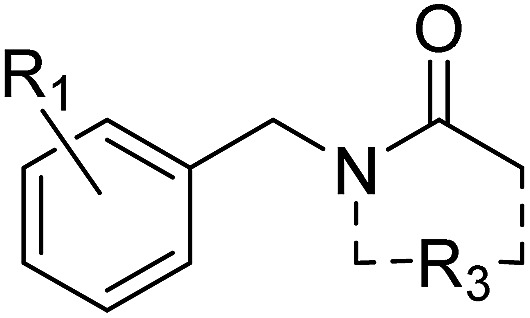						
Cmpd	R_1_	R_3_^*a*^	Yield^*b*^	X-tal soak^*f*^	PHIP(2) pIC_50_^*h*^	LE
**2**	2,6-Cl_2_	C–Me	44%	Success	<2.30 (2)	<0.25
**12**	2,6-(OMe)_2_	C–Me	79%	Success	3.72 ± 0.04 (2)	0.35
**13**	2,6-Cl_2_	C–CH_2_OH	15%^*c*^	Success	<2.30 (2)	<0.23
**14**	2,6-Cl_2_	N–Me, C–Me	83%	Success	<2.30 (2)	<0.23
**15**	2,6-(OMe)_2_	C–CH_2_OH	23%^*d*^	Success^*g*^	<2.30 (2)	<0.20
**16**	2,6-(OMe)_2_	5-Lactam	6%^*e*^	NL	3.25 ± 0.04 (2)	0.27
**17**	2,6-(OMe)_2_	6-Lactam	18%^*e*^	NL	3.51 ± 0.04 (2)	0.27

^*a*^R_3_ = N–H unless stated.

^*b*^Synthesised using procedure described in Scheme 1 unless stated.

^*c*^Reagents and conditions: EDC, DMAP, triethylamine, DMF, rt.

^*d*^Over three steps. See ESI for details.

^*e*^Triethylamine, AcN, rt.

^*f*^NL: no ligand found in model.

^*g*^Binding pose uncertain as a result of partial occupancy.

^*h*^By AlphaScreen peptide displacement assay.

Compounds **2** and **12–17** were tested for inhibition of PHIP(2) ligand binding using the AlphaScreen assay. The original fragment hit **2** was inactive against PHIP(2) at the highest possible assay concentration and would not have been detected as a hit if AlphaScreen had been used as the primary screen. Attempts to modify the acetyl amide group or the benzene ring gave no improvements in potency. The exception was the 2,6-dimethoxy analogue **12** with a pIC_50_ of 3.72 and LE of 0.35.

**Fig. 6:**
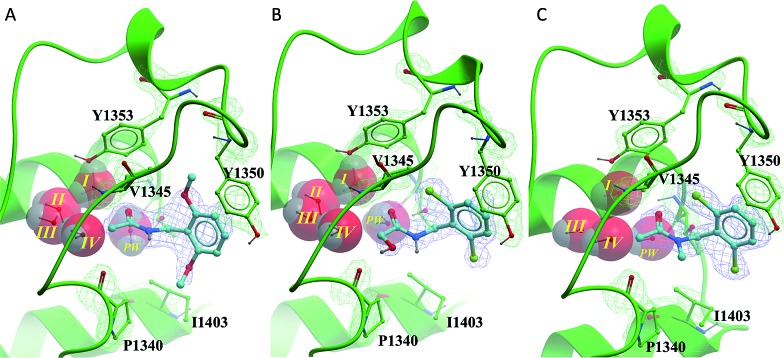
PHIP(2) (green sticks and ribbons) in complex with *N*-benzylacetamides **12–14** (cyan sticks). Electron density shown as green and blue mesh (2Fo-Fc). (A) The amide **12** binds in the same pose as fragment hit **2**. (B) The hydroxyacetamide **13** forms an H-bond to P1340. (C) Tertiary amide **14** appears to displace structural water *II*.

The benzyl amide compounds **12–17** were soaked into PHIP(2) crystals. Surprisingly the inactive compounds **13** and **14** were found to bind in the KAc binding site as well as the active compound **12**. Compound **12** has the same binding mode as fragment hit **2**, with the 2,6-dimethoxy substitution pattern on the benzene ring occupying the V1345 and I1403 lipophilic pockets above and below the ring (Fig. 6A).

Alcohol **13** binds in the same position as fragment hit **2**, with the additional hydroxyl group making an H-bond interaction to the protein backbone at P1340 (Fig. 6B). In contrast, the *N*-methyl group of the tertiary amide **14** forms a hydrophobic interaction with the P1340 sidechain (Fig. 6C).

Despite binding in a similar way to the other amide compounds, tertiary amide **14** appears to displace structural water *II*.

The activity and crystallographic data resulted in three new compounds being proposed for synthesis. All three were synthesized in low yield within two weeks (Table 2, compounds **15–17**). The 2,6-dimethoxy analogue of alcohol **13** (compound **15**) is inactive against PHIP(2) but appears to bind to the protein when subjected to soaking experiments (ESI Fig. S5C†).

Overlay of fragment hit **2** with the previously reported NMP-bound structure (Fig. 2) showed good overlap between the amide binding modes for each compound. A scaffold merging approach was adopted and the lactam containing compounds **16** and **17** proposed and synthesised. Both lactams were found to inhibit the activity of PHIP(2). pIC_50_’s of 3.25 and 3.51 for compounds **16** and **17** respectively demonstrate good activity but LE of only 0.27. The best compound of the amide series, compound **12**, has a calculated LE of 0.35.

#### (c) Oxazoles

The aminooxazoles **3–4**, **18–22** and **s38–s46** were synthesised by addition of aminomalononitrile *p*-toluenesulfonate to the relevant acyl chloride following a previously published method (Scheme 1).^34^ The fragment hits **3** and **4** demonstrated good activity against PHIP(2) with pIC_50_’s of 3.23 and 3.57 respectively (Table 3). The cyclopropyl analogue **18** showed a pIC_50_ of 3.48, comparable to the isobutyl hit **4**. However the isopropyl analogue **19** showed complete loss of activity in the AlphaScreen. The 4-chlorobenzyl derivative **20** showed the best activity with a pIC_50_ of 3.95, although the bulky phenyl ring ensured LE was kept to just 0.35. In comparison, the lighter compounds **4** and **18** have LEs of 0.42 and 0.44 respectively. The cyclohexyl analogue **21** showed good activity (pIC_50_ = 3.31) but substitution of the 5-amino group with *N*-pyrrolidine to give compound **22** increased the pIC_50_ by 0.4. The observation indicates the amine group interacts with T1396 as an H-bond acceptor and a tertiary amine can be tolerated at the 5-position of the oxazole ring. Despite the good activity of the oxazole series, no further crystal structures of the bound ligand were observed following soaking experiments.

**Table 3:** Synthesis and screening of oxazoles **3–4**, **18–22**

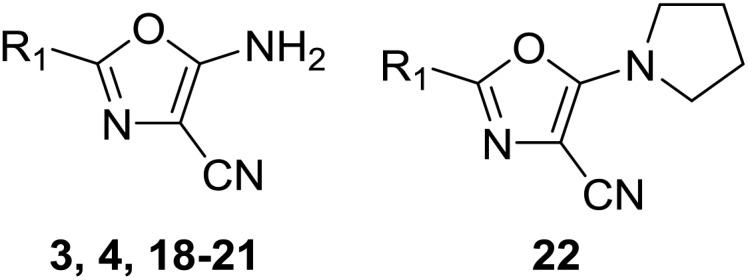					
Cmpd	R_1_	Yield^*a*^	X-tal soak^*c*^	PHIP(2) pIC_50_^*d*^	LE
**3**	Bn	62%	Success	3.23 ± 0.10 (2)	0.30
**4**	^i^Bu	42%	Success	3.57 ± 0.10 (2)	0.42
**18**	Pr^*c*^	93%	NL	3.48 ± 0.03 (2)	0.44
**19**	^i^Pr	54%	NL	<2.30 (2)	<0.29
**20**	4-Cl-Bn	46%	Error	3.95 ± 0.03 (2)	0.35
**21**	Hex^*c*^	71%	NL	3.31 ± 0.06 (2)	0.33
**22**	Hex^*c*^	30%^*b*^	NL	3.71 ± 0.16 (2)	0.29

^*a*^Synthesised using procedure described in Scheme 1 unless stated.

^*b*^Over three steps. See Spencer *et al.* for details.^34^

^*c*^NL: no ligand found in model. Error: experiment failure during soaking, at beamline or during data processing or model refinement.

^*d*^By AlphaScreen peptide displacement assay.

### X-ray structures in screening and follow-up

The need for screening at very high-concentrations has been borne out by the hits identified for PHIP(2) through the X-ray approach, where conventional solution screening using far more compounds had yielded no hits. Moreover, as we have now shown at DLS beamline I04-1 that crystal-based screening is achievable with throughput comparable to NMR, we foresee a re-emergence of its popularity over the historically more practical but less sensitive solution-based techniques.

In contrast, the assessment of the follow-up compounds requires different criteria. Our observation that some of the more potent compounds such as **11** are not visible in the crystal structure upon soaking is line with common anecdotal experience, that crystallographic clarity is not a useful ranking criterion for micromolar compounds. In the fragment field, this manifests as the widely-reported lack of hit overlap between techniques.^42^ A variety of factors influence whether soaking is successful, including compound solubility in the crystallization buffer.

## Conclusions

We have designed and assembled a library of poised fragments to enable rapid elaboration of fragment hits weak enough to be identified only by X-ray crystallography. The identity of the library, called the Diamond SGC Poised Library 2.0 (DSPL2), is freely available (ESI†). The library will be regularly updated to reflect findings regarding compound suitability for soaking, storage and chemical elaboration.

Our in-house poised fragments were used in combination with a novel medium-throughput crystallographic screen to identify the first reported inhibitors of PHIP(2), an atypical bromodomain. By exclusively utilising poised fragments in our screen, we have rapidly generated follow-up leads and shown increases in compound activity alongside highly detailed structural data.

The thiourea **1** displaces one of the structural waters at the back of the PHIP(2) binding site, something rarely seen in other bromodomain ligand–protein complexes. The series was expanded to give highly ligand efficient inhibitors with IC_50_'s of 100–200 μM and calculated LE values greater than 0.40 (compounds **6**, **7**, and **11**).

Amide **2** was found to be inactive in an AlphaScreen assay against PHIP(2), yet one round of poised synthesis yielded compound **12** with an IC_50_ of 190 μM and LE 0.35. Structural data from AlphaScreen-inactive compounds show displacement of structural waters and allowed further development of the series. The 5- and 6-member lactams, **16** and **17**, showed sub-millimolar activity and provide a number of additional vectors for elaboration into unexploited regions of the PHIP(2) binding pocket.

The binding mode of the aminooxazoles **3** and **4** appears to be driven by interactions with T1396, with no displacement of the structural waters or the PHIP water deeper in the binding pocket. The 2-isobutyl and 2-cyclopropyl oxazoles, **4** and **18**, show excellent ligand efficiency, with IC_50_'s 226 μM and 329 μM respectively and LE > 0.40.

Ongoing studies are focusing on improving the affinities of the PHIP(2) hit series reported here with the aim of developing a viable PHIP(2) chemical probe to help further explore the role of this Brd in disease, notably cancer. In parallel, through the SGC's network of synthetic chemistry collaborators, we are actively expanding our poised fragment library beyond the simple chemistry described here with compounds derived from sp^3^ rich, stereochemically controlled poised reactions.

We submit that the use of a crystallographic primary screen followed by rapid poised chemistry to generate a follow-up library offers a new, powerful method in hit discovery and lead series selection which can be utilised by both academic and industrial researchers. The success in finding efficient hits for the low druggability PHIP(2) bromodomain indicates the power of this method in addressing difficult protein–protein interaction targets.

## Supplementary Material

Supplementary information

Supplementary information

Supplementary information

Supplementary information
