# Supplementary material for: A poised fragment library enables rapid synthetic expansion yielding the first reported inhibitors of PHIP(2), an atypical bromodomain
Source: Chem Sci. 2015 Dec 22;7(3):2322–30. doi: 10.1039/c5sc03115j (PMC5977933; doi:10.1039/c5sc03115j)

Compound **1**

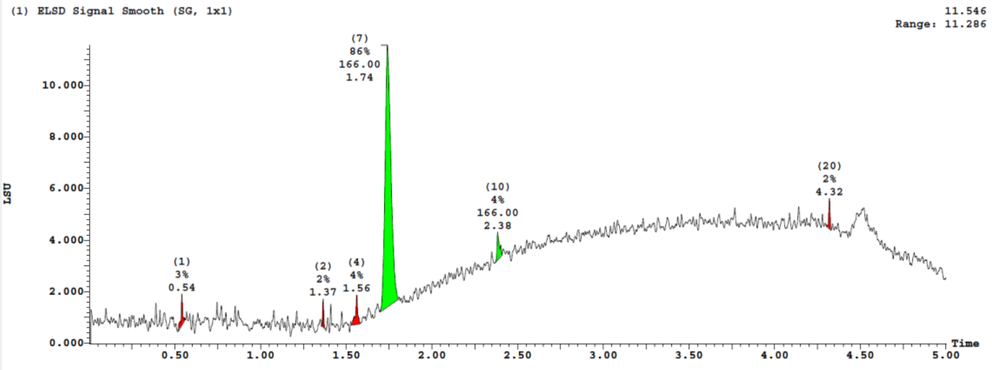

Acquisition Time (sec)	4.0894	***Comment***	OC 032 H1	***Date***	11 Jul 2014 05:01:04		
Date Stamp	11 Jul 2014 05:01:04			***File Name***	\\hestia\work\chemistry\NMR Files\NMR data\OCox\140710 OC 001-032\12 H1\pdata\1\1r		
Frequency (MHz)	400.13	***Nucleus***	1H	***Number of Transients***	16	***Origin***	Spect
Original Points Count	32768	***Owner***	sgcnmr	***Points Count***	65536	***Pulse Sequence***	zg30
Receiver Gain	194.71	***SW(cyclical) (Hz)***	8012.82	***Solvent***	DMSO-d6	***Spectrum Offset (Hz)***	2467.8220
Spectrum Type	STANDARD	***Sweep Width (Hz)***	8012.70	***Temperature (degree C)***	25.154		

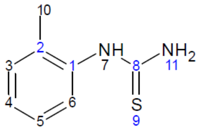

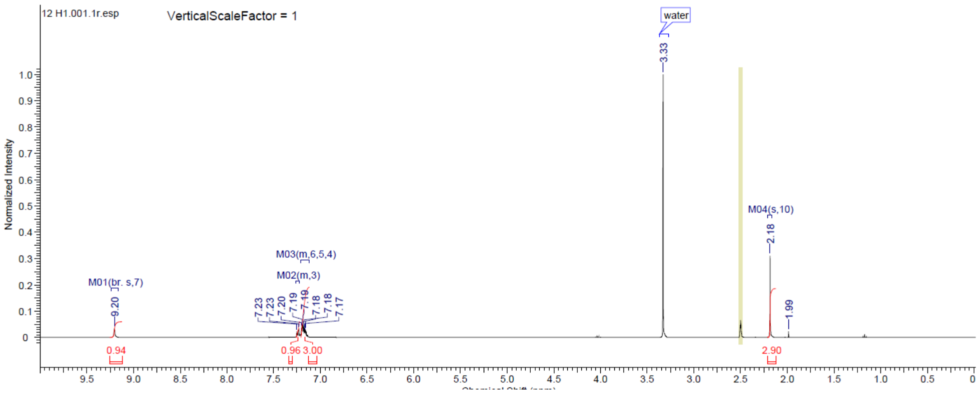

Acquisition Time (sec)	1.3631	***Comment***	OC 032 C13	***Date***	11 Jul 2014 04:39:44		
Date Stamp	11 Jul 2014 04:39:44			***File Name***	\\hestia\work\chemistry\NMR Files\NMR data\OCox\140710 OC 001-032\10 C13\pdata\1\1r		
Frequency (MHz)	100.61	***Nucleus***	13C	***Number of Transients***	1024	***Origin***	Spect
Original Points Count	32768	***Owner***	sgcnmr	***Points Count***	32768	***Pulse Sequence***	zgpg30
Receiver Gain	194.71	***SW(cyclical) (Hz)***	24038.46	***Solvent***	DMSO-d6	***Spectrum Offset (Hz)***	10059.8594
Spectrum Type	STANDARD	***Sweep Width (Hz)***	24037.73	***Temperature (degree C)***	25.159		

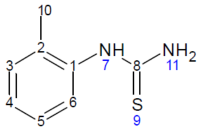

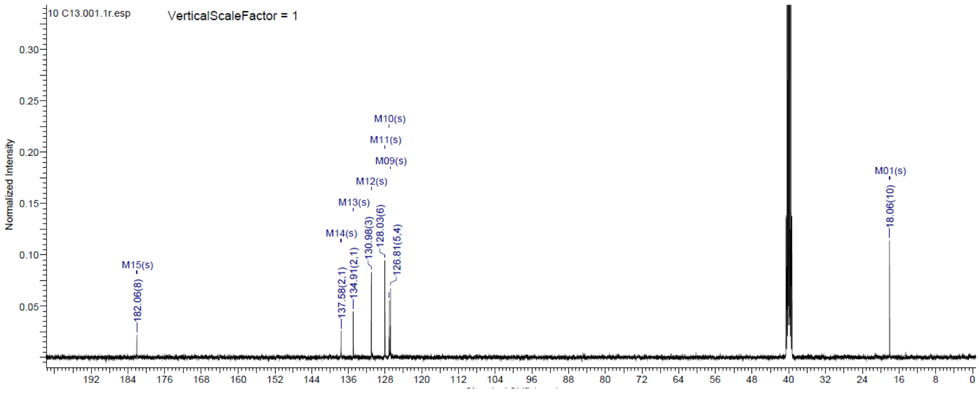

Compound **2**

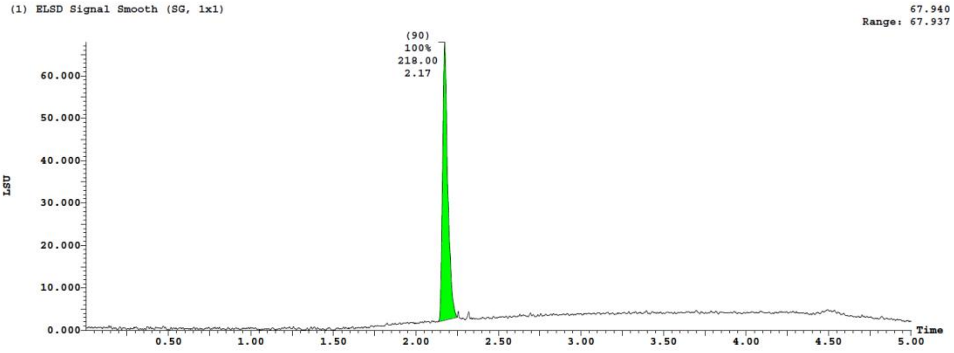

Acquisition Time (sec)	4.0894	Comment	OC_028_H1	Date	12 Jul 2014 05:50:08		
Date Stamp	12 Jul 2014 05:50:08			File Name	\\hestia\work\chemistry\NMR Files\NMR_data\OCox\140710_OC_001-028\12 H1\pdata\1\1r		
Frequency (MHz)	400.13	Nucleus	1H	Number of Transients	16	Origin	Spect
Original Points Count	32768	Owner	sgcnmr	Points Count	65536	Pulse Sequence	zg30
Receiver Gain	194.71	SW(cyclical) (Hz)	8012.82	Solvent	DMSO-d6	Spectrum Offset (Hz)	2467.8220
Spectrum Type	STANDARD	Sweep Width (Hz)	8012.70	Temperature (degree C)	25.155		

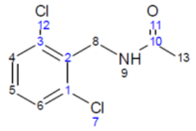

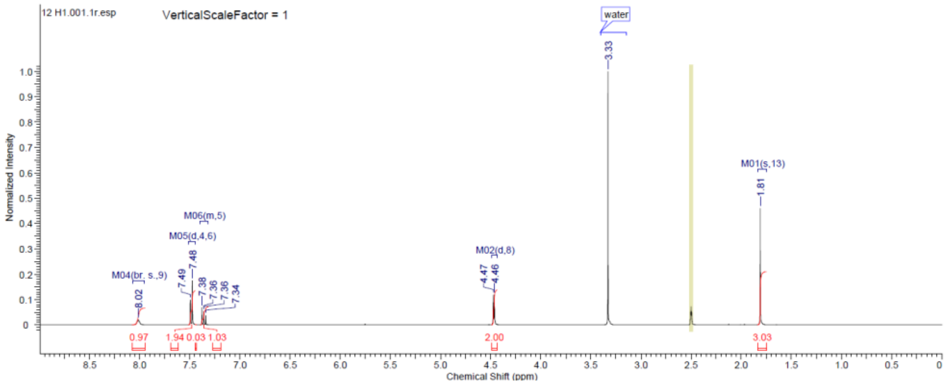

Acquisition Time (sec)	1.3631	Comment	OC_028_C13	Date	12 Jul 2014 05:28:48		
Date Stamp	12 Jul 2014 05:28:48			File Name	\\hestia\work\chemistry\NMR Files\NMR_data\OCox\140710_OC_001-028\10 C13\pdata\1\1r		
Frequency (MHz)	100.61	Nucleus	13C	Number of Transients	1024	Origin	Spect
Original Points Count	32768	Owner	sgcnmr	Points Count	32768	Pulse Sequence	zgpg30
Receiver Gain	194.71	SW(cyclical) (Hz)	24038.46	Solvent	DMSO-d6	Spectrum Offset (Hz)	10059.8594
Spectrum Type	STANDARD	Sweep Width (Hz)	24037.73	Temperature (degree C)	25.160		

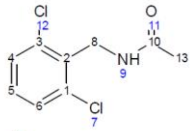

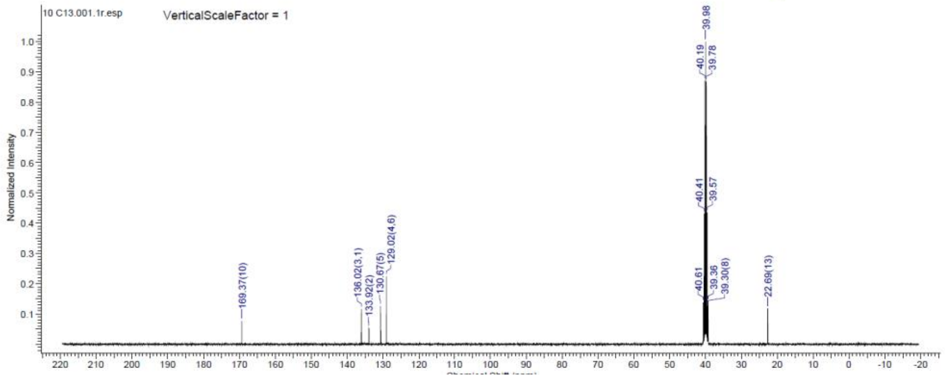

Compound **3**

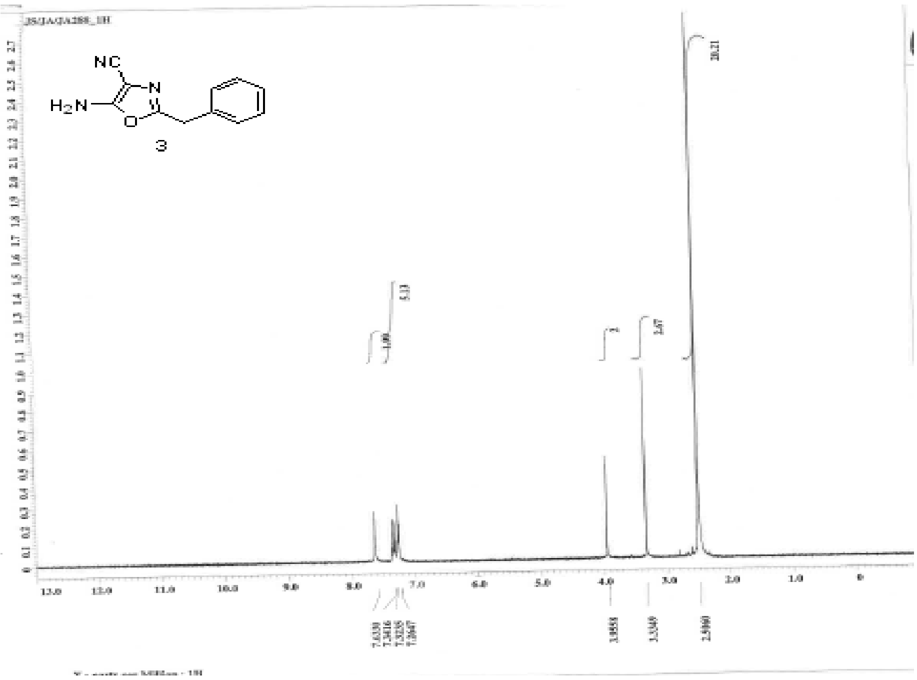

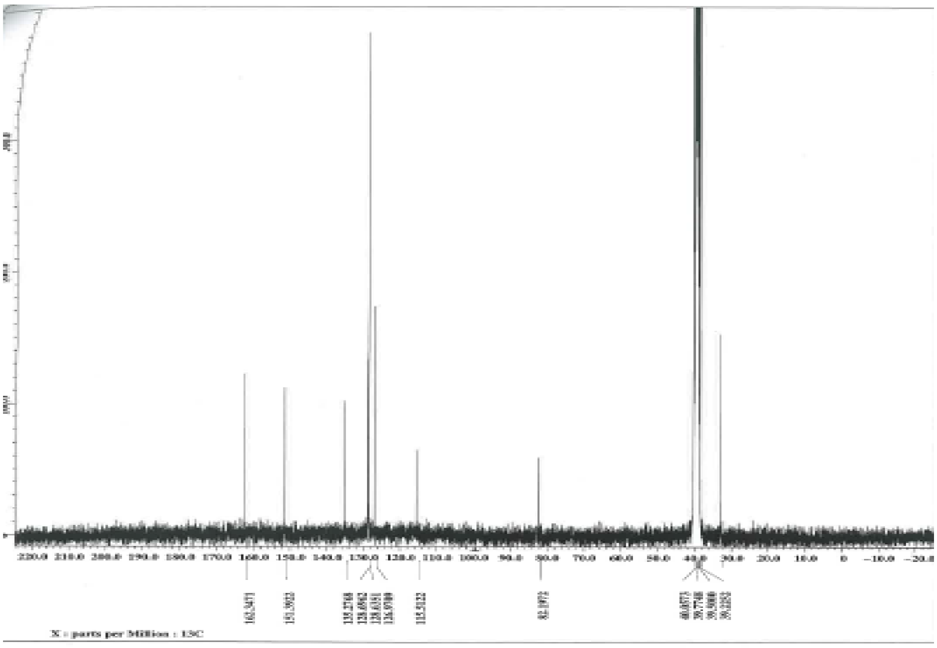

Compound **4**

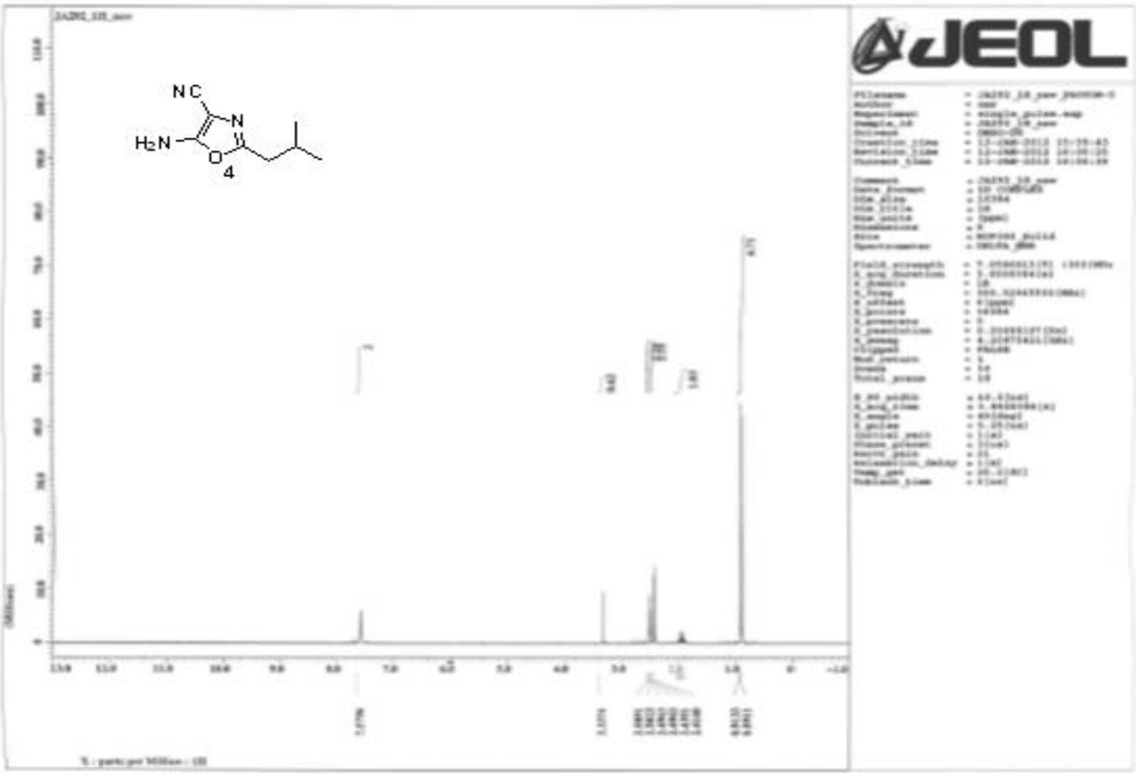

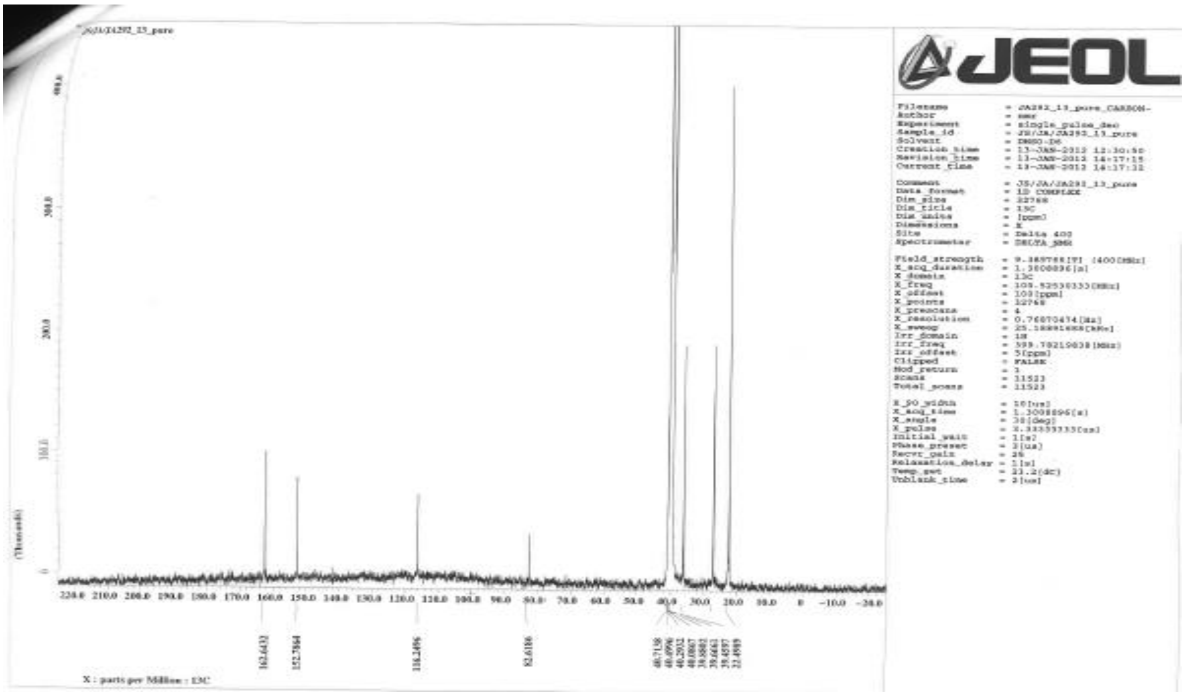

Compound **5**

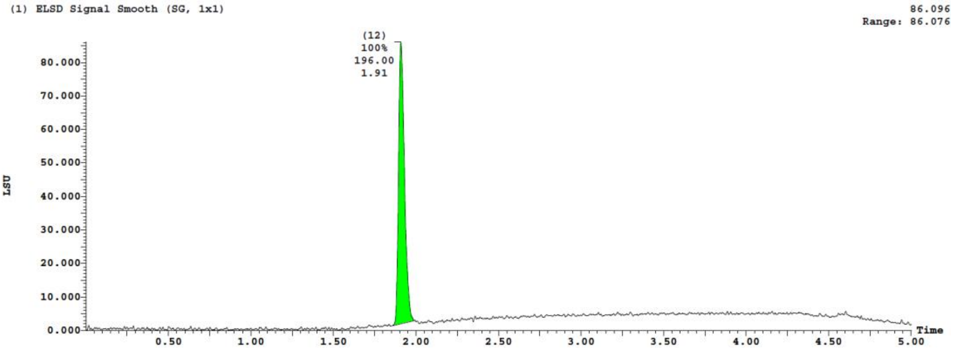

Acquisition Time (sec)	4.0894	***Comment***	OC_040_D_H1	***Date***	08 Aug 2014 21:05:20		
Date Stamp	08 Aug 2014 21:05:20			***File Name***	\\hestia\work\chemistry\NMR Files\NMR_data\OCox\140807_OC_001-040D\12-H1\pdata\1\1r		
Frequency (MHz)	400.13	***Nucleus***	1H	***Number of Transients***	16	***Origin***	Spect
Original Points Count	32768	***Owner***	sgcnmr	***Points Count***	65536	***Pulse Sequence***	zg30
Receiver Gain	77.20	***SW(cyclical) (Hz)***	8012.82	***Solvent***	DMSO-d6	***Spectrum Offset (Hz)***	2470.9668
Spectrum Type	STANDARD	***Sweep Width (Hz)***	8012.70	***Temperature (degree C)***	25.149		

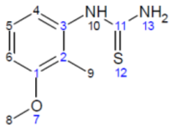

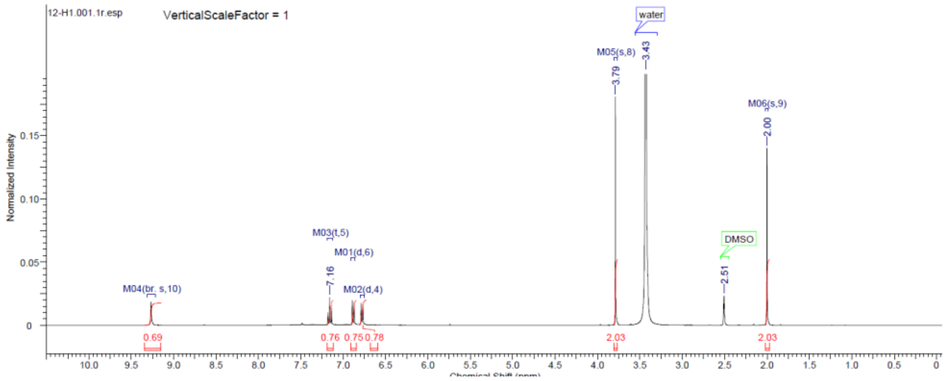

Acquisition Time (sec)	1.3631	***Comment***	OC_040_D_C13	***Date***	08 Aug 2014 21:03:12		
Date Stamp	08 Aug 2014 21:03:12			***File Name***	\\hestia\work\chemistry\NMR Files\NMR_data\OCox\140807_OC_001-040D\11-C13\pdata\1\1r		
Frequency (MHz)	100.61	***Nucleus***	13C	***Number of Transients***	1024	***Origin***	Spect
Original Points Count	32768	***Owner***	sgcnmr	***Points Count***	32768	***Pulse Sequence***	jmod
Receiver Gain	194.71	***SW(cyclical) (Hz)***	24038.46	***Solvent***	DMSO-d6	***Spectrum Offset (Hz)***	10060.3320
Spectrum Type	APT	***Sweep Width (Hz)***	24037.73	***Temperature (degree C)***	25.159		

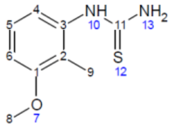

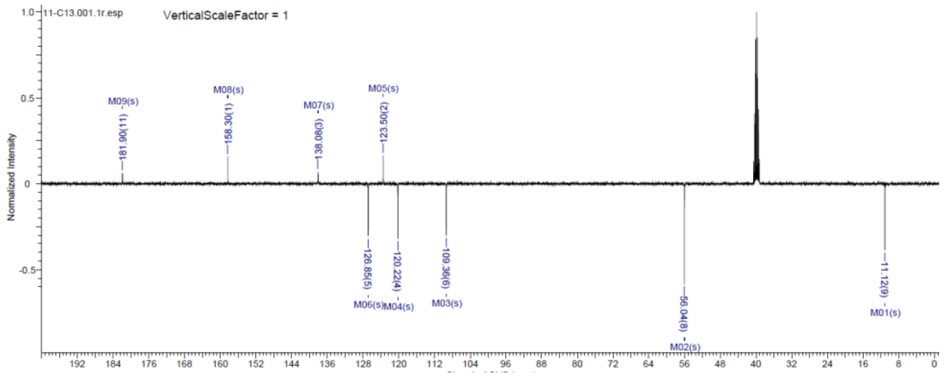

Compound **6**

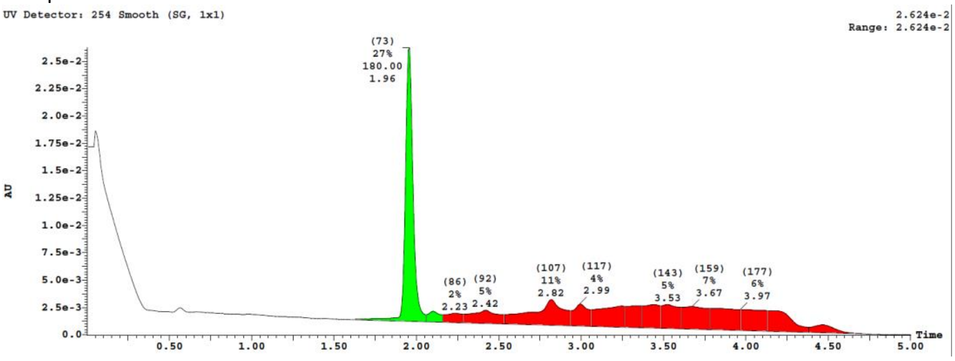

Acquisition Time (sec)	4.0894	***Comment***	OC 039 I H1	***Date***	06 Aug 2014 00:28:00		
Date Stamp	06 Aug 2014 00:28:00			***File Name***	\\hestia\work\chemistry\NMR Files\NMR data\OCox\140805 OC 001-039I\12-H1\pdata\1\1r		
Frequency (MHz)	400.13	***Nucleus***	1H	***Number of Transients***	16	***Origin***	Spect
Original Points Count	32768	***Owner***	sgcnmr	***Points Count***	65536	***Pulse Sequence***	zg30
Receiver Gain	155.34	***SW(cyclical) (Hz)***	8012.82	***Solvent***	DMSO-d6	***Spectrum Offset (Hz)***	2470.9668
Spectrum Type	STANDARD	***Sweep Width (Hz)***	8012.70	***Temperature (degree C)***	25.151		

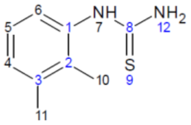

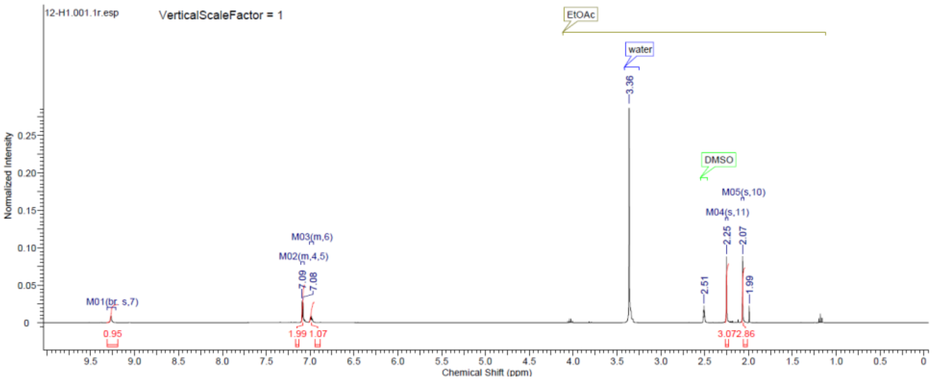

Acquisition Time (sec)	1.3631	***Comment***	OC 039 I C13	***Date***	06 Aug 2014 00:25:52		
Date Stamp	06 Aug 2014 00:25:52			***File Name***	\\hestia\work\chemistry\NMR Files\NMR data\OCox\140805 OC 001-039I\11-C13\pdata\1\1r		
Frequency (MHz)	100.61	***Nucleus***	13C	***Number of Transients***	1024	***Origin***	Spect
Original Points Count	32768	***Owner***	sgcnmr	***Points Count***	32768	***Pulse Sequence***	jmod
Receiver Gain	194.71	***SW(cyclical) (Hz)***	24038.46	***Solvent***	DMSO-d6	***Spectrum Offset (Hz)***	10060.3320
Spectrum Type	APT	***Sweep Width (Hz)***	24037.73	***Temperature (degree C)***	25.161		

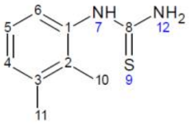

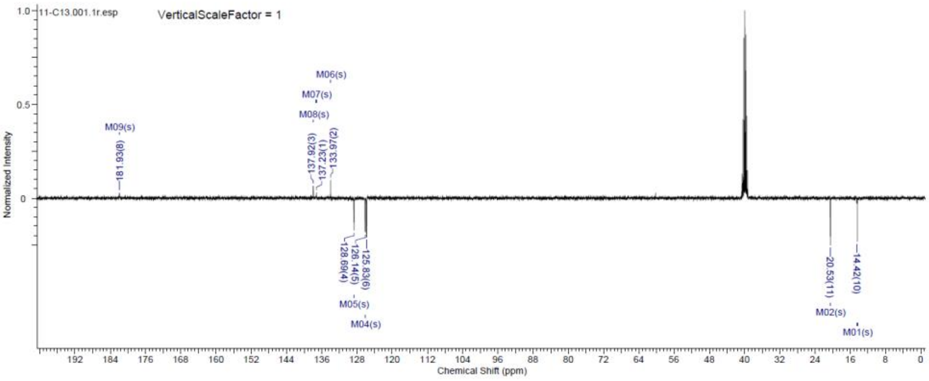

Compound **7**

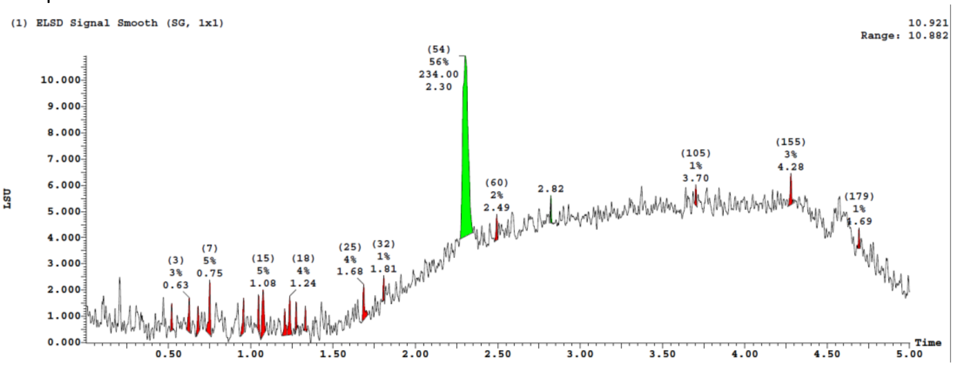

Acquisition Time (sec)	4.0894	Comment	OC 039 J H1	Date	06 Aug 2014 02:33:52		
Date Stamp	06 Aug 2014 02:33:52			File Name	\\hestia\work\chemistry\NMR Files\NMR data\OCox\140805 OC 001-039J\12-H1\pdata\1\1r		
Frequency (MHz)	400.13	Nucleus	1H	Number of Transients	16	Origin	Spect
Original Points Count	32768	Owner	sgcnmr	Points Count	65536	Pulse Sequence	zg30
Receiver Gain	174.26	SW(cyclical) (Hz)	8012.82	Solvent	DMSO-d6	Spectrum Offset (Hz)	2470.9668
Spectrum Type	STANDARD	Sweep Width (Hz)	8012.70	Temperature (degree C)	25.150		

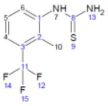

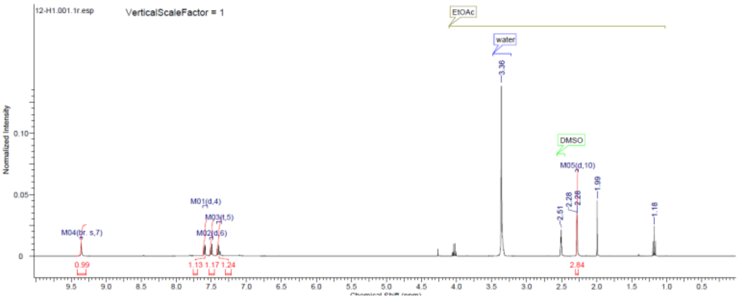

Acquisition Time (sec)	1.3631	Comment	OC 039 J C13			Date	06 Aug 2014 02:31:44
Date Stamp	06 Aug 2014 02:31:44			File Name	\\hestia\work\chemistry\NMR Files\NMR data\OCox\140805 OC 001-039J\11-C13\pdata\1\1r		
Frequency (MHz)	100.61	Nucleus	13C	Number of Transients	1024	Origin	Spect
Original Points Count	32768	Owner	sgcnmr	Points Count	32768	Pulse Sequence	jmod
Receiver Gain	194.71	SW(cyclical) (Hz)	24038.46	Solvent	DMSO-d6	Spectrum Offset (Hz)	10060.3320
Spectrum Type	APT	Sweep Width (Hz)	24037.73	Temperature (degree C)	25.162		

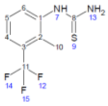

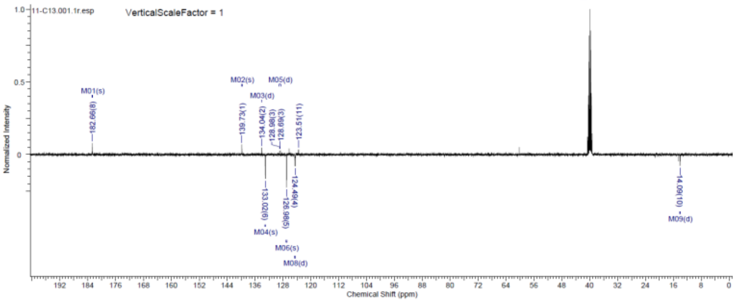

Acquisition Time (sec)	0.7330	Comment	OC 039J F19	Date	22 Aug 2014 17:46:56		
Date Stamp	22 Aug 2014 17:46:56			File Name	\\hestia\work\chemistry\NMR Files\NMR data\OCox\140822 OC 001-039J F19\10\pdata\1\1r		
Frequency (MHz)	376.50	Nucleus	19F	Number of Transients	16	Origin	Spect
Original Points Count	65446	Owner	[illegible]	Points Count	131072	Pulse Sequence	zgflqn
Receiver Gain	194.71	SW(cyclical) (Hz)	89285.71	Solvent	DMSO-d6	Spectrum Offset (Hz)	-37649.6211
Spectrum Type	STANDARD	Sweep Width (Hz)	89285.03	Temperature (degree C)	25.159		

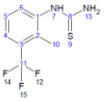

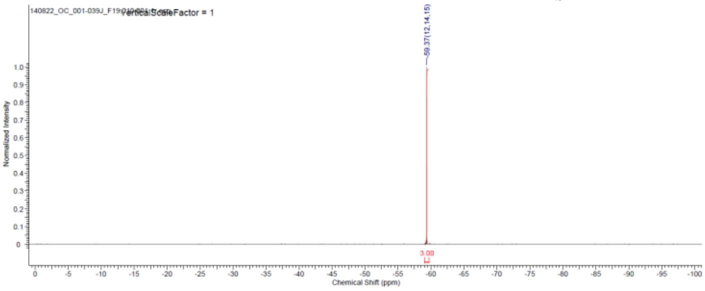

Compound **8**

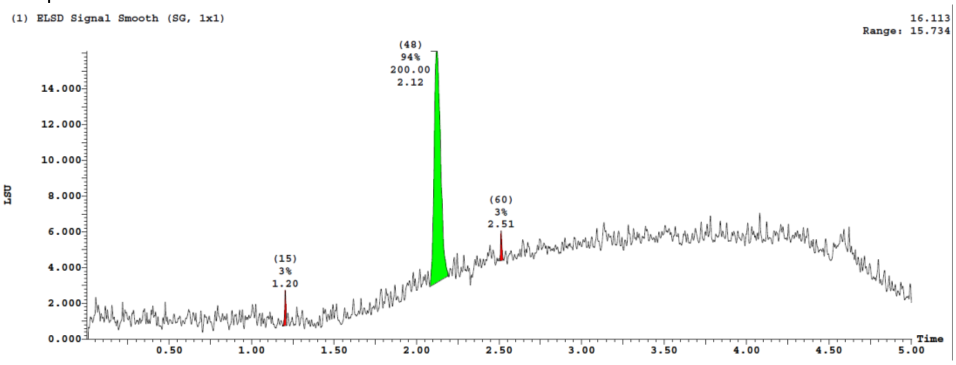

Acquisition Time (sec)	4.0894	Comment	OC_039G_H1	Date	14 Aug 2014 00:04:32		
Date Stamp	14 Aug 2014 00:04:32			File Name	\\hestia\work\chemistry\NMR Files\NMR_data\OCox\140813_OC_001-039G_HMBC\10\pdata\1\1r		
Frequency (MHz)	400.13	Nucleus	1H	Number of Transients	16	Origin	Spect
Original Points Count	32768	Owner	sgcnmr	Points Count	65536	Pulse Sequence	zg30
Receiver Gain	174.26	SW(cyclical) (Hz)	8012.82	Solvent	DMSO-d6	Spectrum Offset (Hz)	2470.9668
Spectrum Type	STANDARD	Sweep Width (Hz)	8012.70	Temperature (degree C)	25.162		

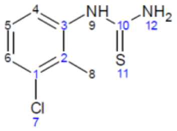

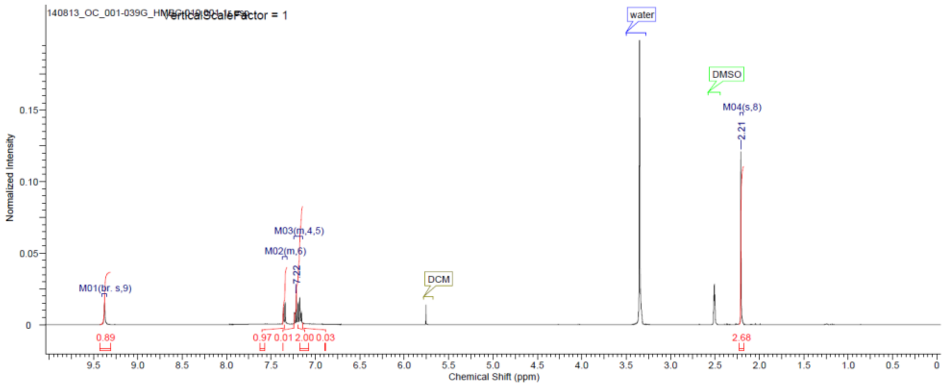

Acquisition Time (sec)	1.3631	Comment	OC_039_G_C13			Date	04 Aug 2014 21:03:12
Date Stamp	04 Aug 2014 21:03:12			File Name	\\hestia\work\chemistry\NMR Files\NMR_data\OCox\140804_OC_001-039G\20-C13\pdata\1\1r		
Frequency (MHz)	100.61	Nucleus	13C	Number of Transients	1024	Origin	Spect
Original Points Count	32768	Owner	sgcnmr	Points Count	32768	Pulse Sequence	jmod
Receiver Gain	194.71	SW(cyclical) (Hz)	24038.46	Solvent	DMSO-d6	Spectrum Offset (Hz)	10060.3320
Spectrum Type	APT	Sweep Width (Hz)	24037.73	Temperature (degree C)	25.159		

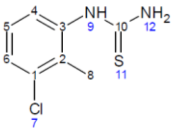

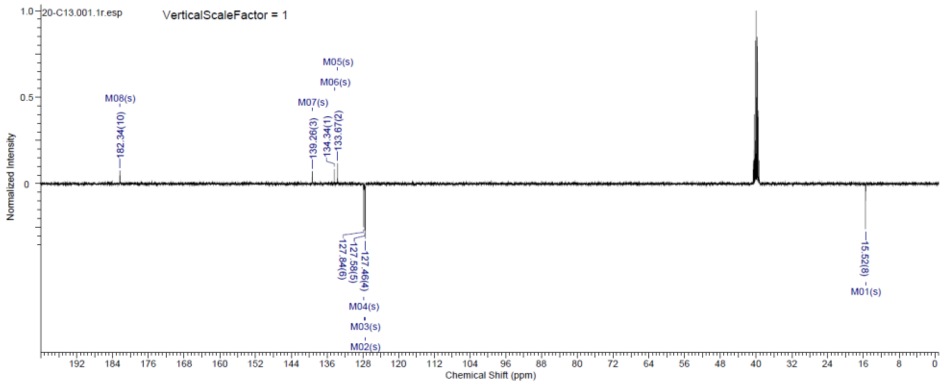

Compound **9**

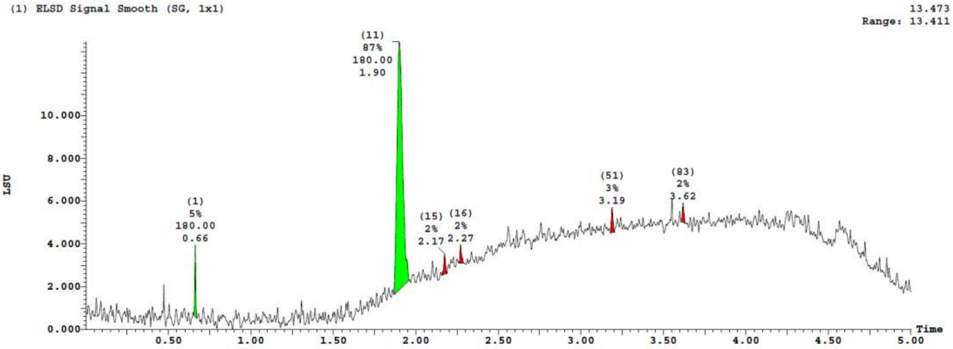

Acquisition Time (sec)	4.0894	Comment	OC 040 C H1	Date	07 Aug 2014 23:11:12		
Date Stamp	07 Aug 2014 23:11:12			File Name	\\hestia\work\chemistry\NMR Files\NMR data\OCox\140807 OC 001-040C\12-H1\pdata\1\1r		
Frequency (MHz)	400.13	Nucleus	1H	Number of Transients	16	Origin	Spect
Original Points Count	32768	Owner	sgcnmr	Points Count	65536	Pulse Sequence	zg30
Receiver Gain	77.20	SW(cyclical) (Hz)	8012.82	Solvent	DMSO-d6	Spectrum Offset (Hz)	2470.9668
Spectrum Type	STANDARD	Sweep Width (Hz)	8012.70	Temperature (degree C)	25.148		

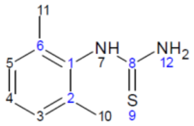

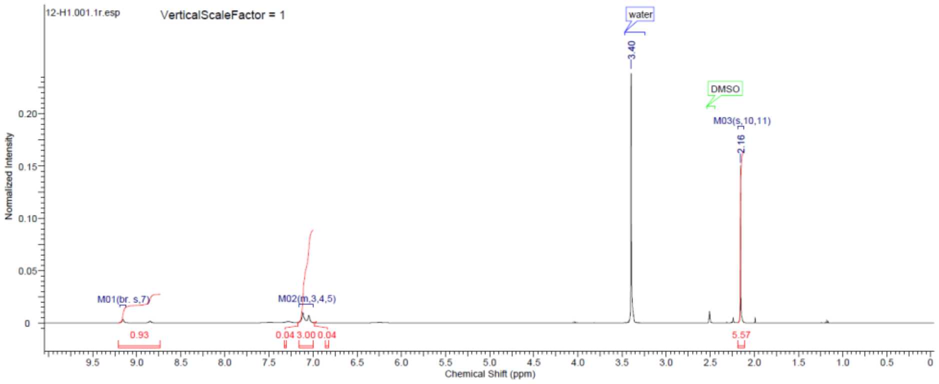

Acquisition Time (sec)	1.3631	Comment	OC 040 C C13			Date	07 Aug 2014 23:09:04
Date Stamp	07 Aug 2014 23:09:04			File Name	\\hestia\work\chemistry\NMR Files\NMR data\OCox\140807 OC 001-040C\11-APT\pdata\1\1r		
Frequency (MHz)	100.61	Nucleus	13C	Number of Transients	1024	Origin	Spect
Original Points Count	32768	Owner	sgcnmr	Points Count	32768	Pulse Sequence	jmod
Receiver Gain	194.71	SW(cyclical) (Hz)	24038.46	Solvent	DMSO-d6	Spectrum Offset (Hz)	10060.3320
Spectrum Type	APT	Sweep Width (Hz)	24037.73	Temperature (degree C)	25.159		

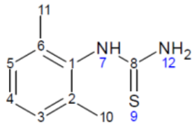

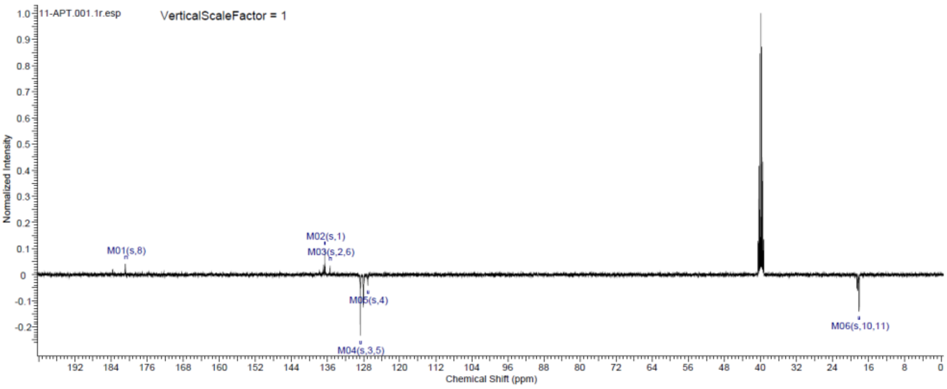

Compound **10**

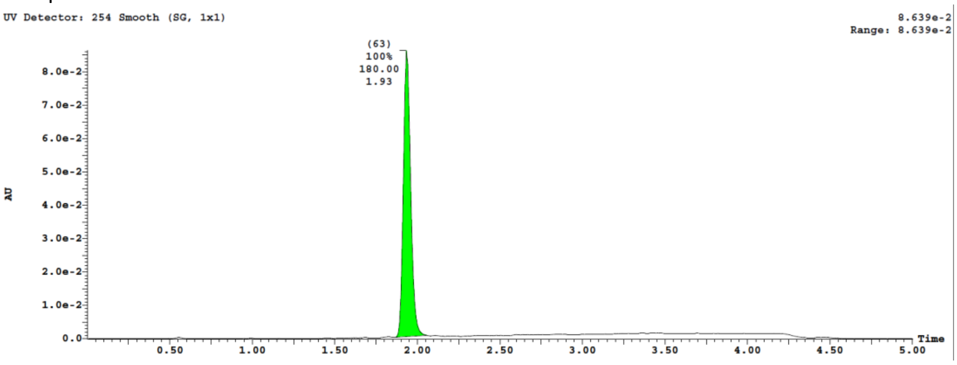

Acquisition Time (sec)	4.0894	Comment	OC 029 A H1	Date	12 Jul 2014 03:25:04		
Date Stamp	12 Jul 2014 03:25:04			File Name	\\hestia\work\chemistry\NMR Files\NMR data\OCox\140710 OC 001-029A\12 H1\pdata\1\1r		
Frequency (MHz)	400.13	Nucleus	1H	Number of Transients	16	Origin	Spect
Original Points Count	32768	Owner	sgcnmr	Points Count	65536	Pulse Sequence	zg30
Receiver Gain	194.71	SW(cyclical) (Hz)	8012.82	Solvent	CHLOROFORM-d		
Spectrum Offset (Hz)	2465.3035	Spectrum Type	STANDARD	Sweep Width (Hz)	8012.70	Temperature (degree C)	25.157

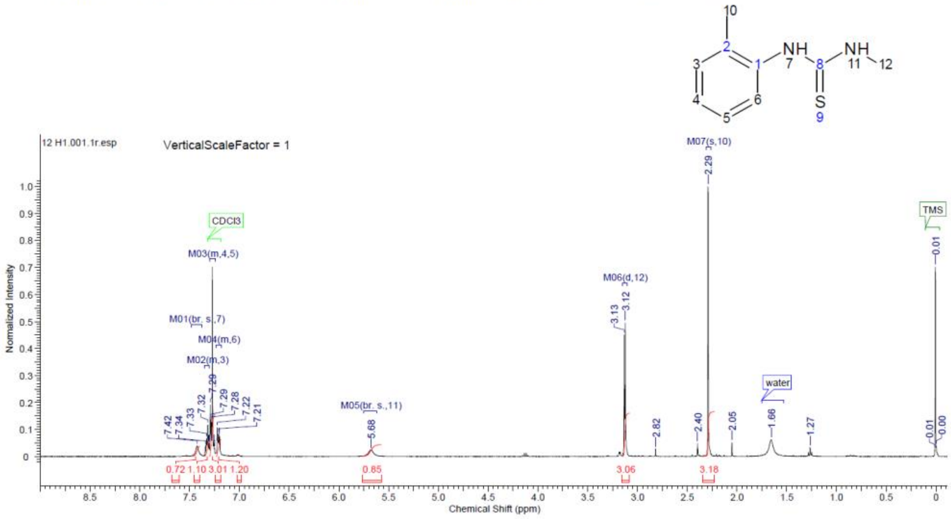

Acquisition Time (sec)	1.3631	Comment	OC 029 A C13	Date	12 Jul 2014 03:03:44		
Date Stamp	12 Jul 2014 03:03:44			File Name	\\hestia\work\chemistry\NMR Files\NMR data\OCox\140710 OC 001-029A\10 C13\pdata\1\1r		
Frequency (MHz)	100.61	Nucleus	13C	Number of Transients	1024	Origin	Spect
Original Points Count	32768	Owner	sgcnmr	Points Count	32768	Pulse Sequence	zgpg30
Receiver Gain	194.71	SW(cyclical) (Hz)	24038.46	Solvent	CHLOROFORM-d		
Spectrum Offset (Hz)	10058.3760	Spectrum Type	STANDARD	Sweep Width (Hz)	24037.73	Temperature (degree C)	25.158

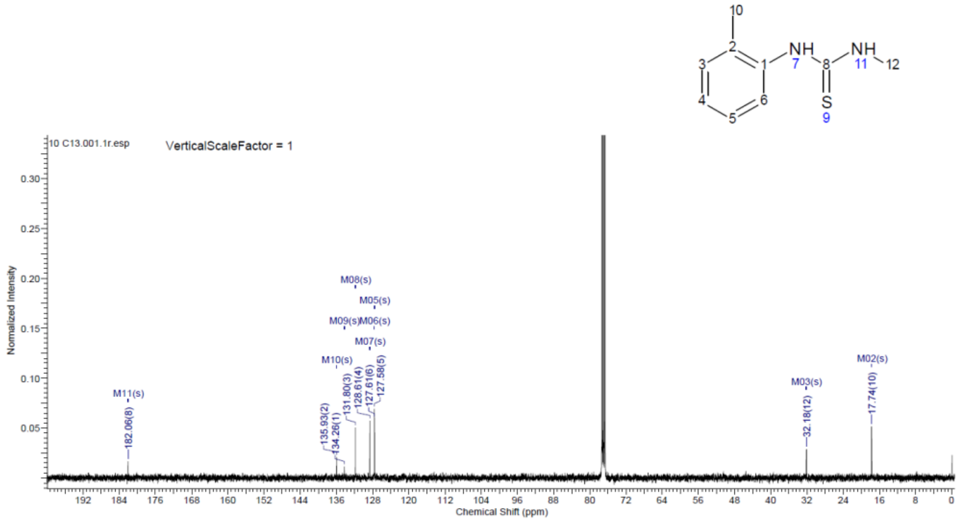

Compound **11**

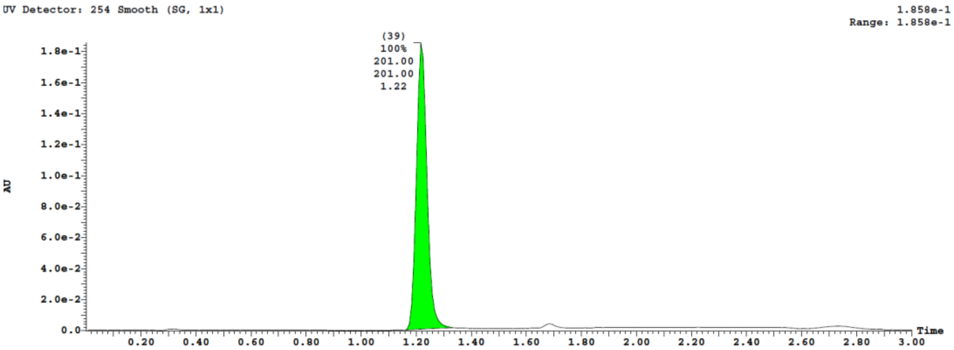

Acquisition Time (sec)	4.0894	*Comment*	OC_050_F	*Date*	09 Dec 2014 09:44:32		
Date Stamp	09 Dec 2014 09:44:32			*File Name*	\\hestia\work\chemistry\NMR Files\NMR_data\OCox\141209_OC_001-050F\10-H1\pdata\1\1r		
Frequency (MHz)	400.13	*Nucleus*	1H	*Number of Transients*	16	*Origin*	Spect
Original Points Count	32768	*Owner*	sgcnmr	*Points Count*	65536	*Pulse Sequence*	zg30
Receiver Gain	194.71	*SW(cyclical) (Hz)*	8012.82	*Solvent*	CHLOROFORM-d		
Spectrum Offset (Hz)	2470.9668	*Spectrum Type*	STANDARD	*Sweep Width (Hz)*	8012.70	*Temperature (degree C)*	25.157

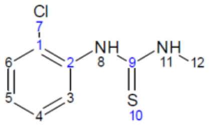

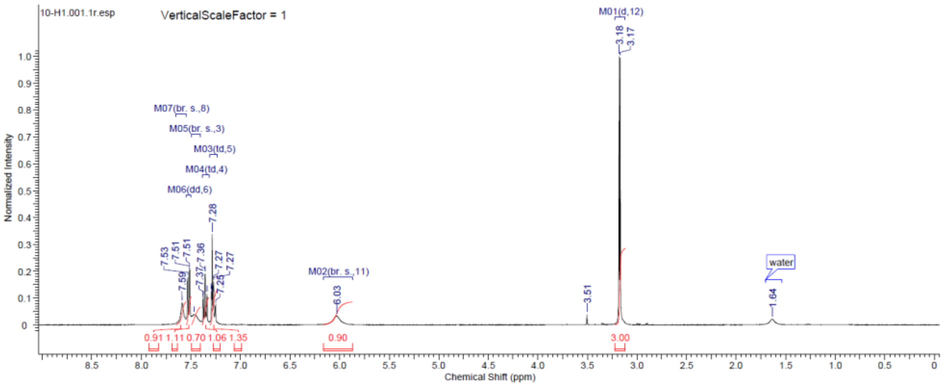

Acquisition Time (sec)	1.3631	*Comment*	OC_050_F_C13	*Date*	10 Dec 2014 02:01:36		
Date Stamp	10 Dec 2014 02:01:36			*File Name*	\\hestia\work\chemistry\NMR Files\NMR_data\OCox\141209_OC_001-050F\15-C13\pdata\1\1r		
Frequency (MHz)	100.61	*Nucleus*	13C	*Number of Transients*	4096	*Origin*	Spect
Original Points Count	32768	*Owner*	sgcnmr	*Points Count*	32768	*Pulse Sequence*	zgpg30
Receiver Gain	194.71	*SW(cyclical) (Hz)*	24038.46	*Solvent*	CHLOROFORM-d		
Spectrum Offset (Hz)	10059.8594	*Spectrum Type*	STANDARD	*Sweep Width (Hz)*	24037.73	*Temperature (degree C)*	25.160

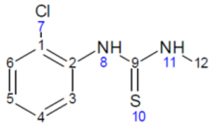

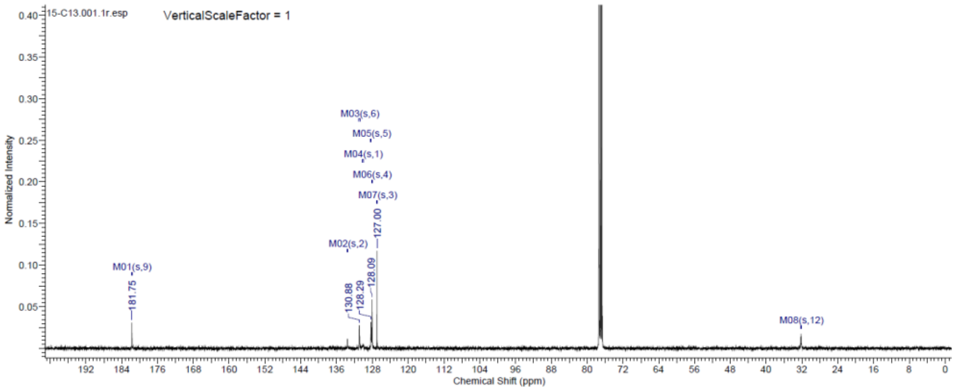

Compound **12**

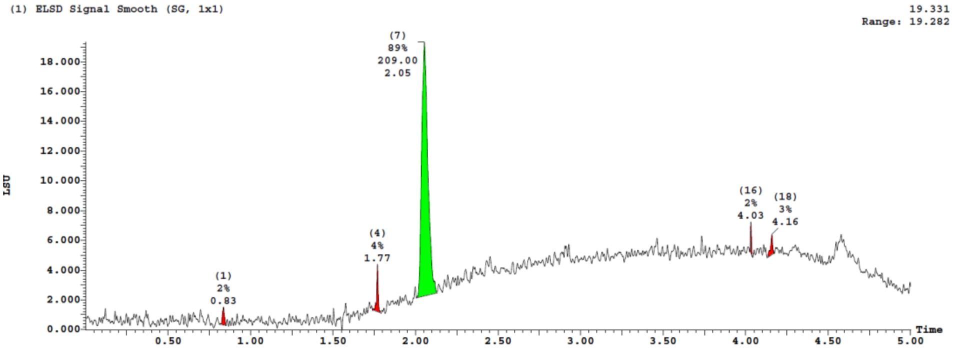

Acquisition Time (sec)	4.0894	Comment	OC_037_G_H1	Date	31 Jul 2014 07:13:20		
Date Stamp	31 Jul 2014 07:13:20			File Name	\\hestia\work\chemistry\NMR Files\NMR_data\OCox\140730_OC_001-037G\11-H1\pdata\1\1r		
Frequency (MHz)	400.13	Nucleus	1H	Number of Transients	16	Origin	Spect
Original Points Count	32768	Owner	sgcnmr	Points Count	65536	Pulse Sequence	zg30
Receiver Gain	194.71	SW(cyclical) (Hz)	8012.82	Solvent	CHLOROFORM-d		
Spectrum Offset (Hz)	2434.1191	Spectrum Type	STANDARD	Sweep Width (Hz)	8012.70	Temperature (degree C)	25.154

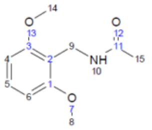

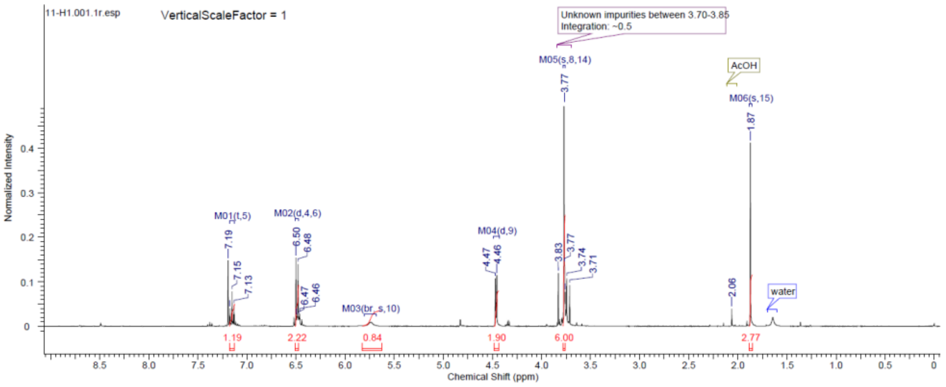

Acquisition Time (sec)	1.3631	Comment	OC_037_G_C13			Date	01 Aug 2014 01:59:44
Date Stamp	01 Aug 2014 01:59:44			File Name	\\hestia\work\chemistry\NMR Files\NMR_data\OCox\140730_OC_001-037G\10-C13\pdata\1\1r		
Frequency (MHz)	100.61	Nucleus	13C	Number of Transients	1024	Origin	Spect
Original Points Count	32768	Owner	sgcnmr	Points Count	32768	Pulse Sequence	jmod
Receiver Gain	194.71	SW(cyclical) (Hz)	24038.46	Solvent	CHLOROFORM-d		
Spectrum Offset (Hz)	10060.3320	Spectrum Type	APT	Sweep Width (Hz)	24037.73	Temperature (degree C)	25.160

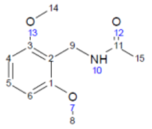

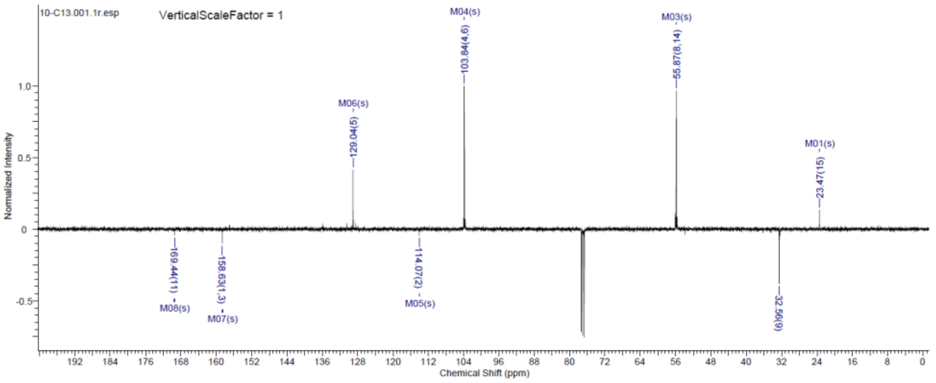

Compound **13**

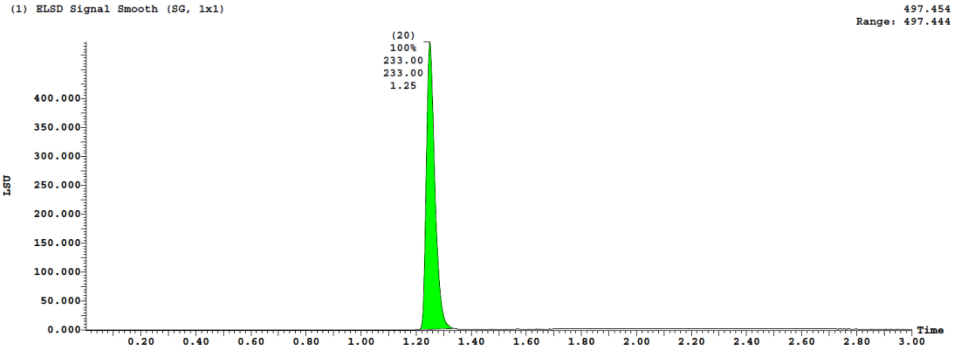

Acquisition Time (sec)	4.0894	Comment	OC 035 H1	Date	10 Jul 2014 15:26:08		
Date Stamp	10 Jul 2014 15:26:08			File Name	\\hestia\work\chemistry\NMR Files\NMR data\OCox\140710 OC 001-035\12-H1\pdata\1\1r		
Frequency (MHz)	400.13	Nucleus	1H	Number of Transients	16	Origin	Spect
Original Points Count	32768	Owner	sgcnmr	Points Count	65536	Pulse Sequence	zg30
Receiver Gain	194.71	SW(cyclical) (Hz)	8012.82	Solvent	CHLOROFORM-d		
Spectrum Offset (Hz)	2462.7417	Spectrum Type	STANDARD	Sweep Width (Hz)	8012.70	Temperature (degree C)	25.157

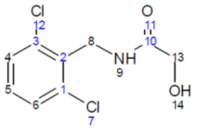

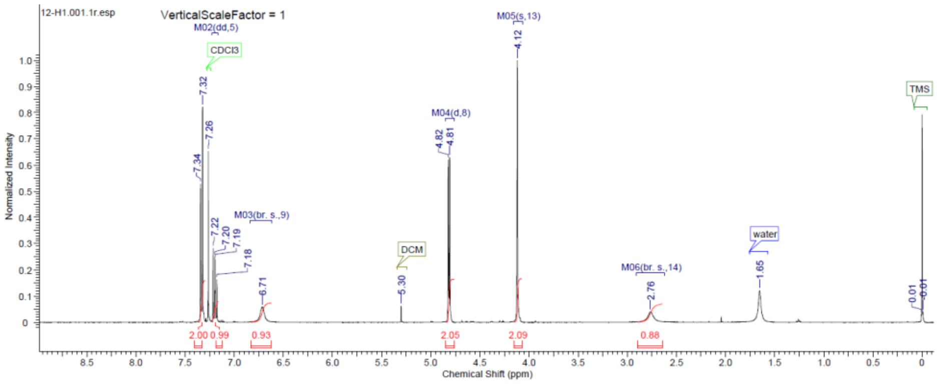

Acquisition Time (sec)	1.3631	Comment	OC 035 HMBC			Date	12 Jul 2014 08:40:48
Date Stamp	12 Jul 2014 08:40:48			File Name	\\hestia\work\chemistry\NMR Files\NMR data\OCox\140710 OC 001-035\16-C13\pdata\1\1r		
Frequency (MHz)	100.61	Nucleus	13C	Number of Transients	1024	Origin	Spect
Original Points Count	32768	Owner	sgcnmr	Points Count	32768	Pulse Sequence	jmod
Receiver Gain	194.71	SW(cyclical) (Hz)	24038.46	Solvent	CHLOROFORM-d		
Spectrum Offset (Hz)	10060.8965	Spectrum Type	APT	Sweep Width (Hz)	24037.73	Temperature (degree C)	25.160

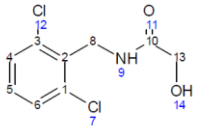

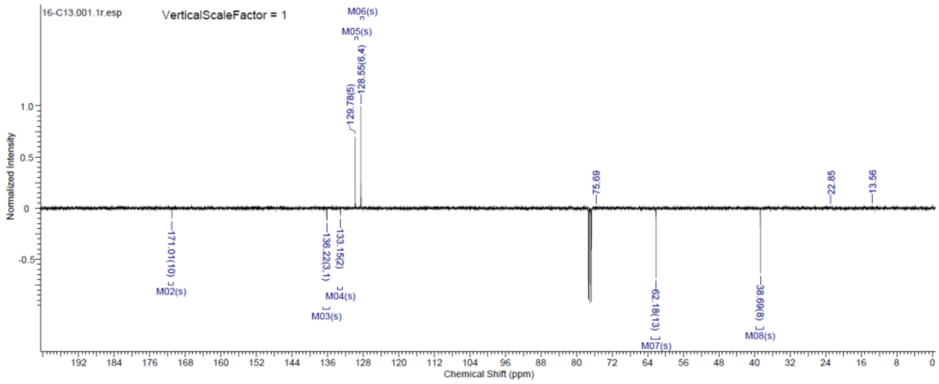

Compound **14**

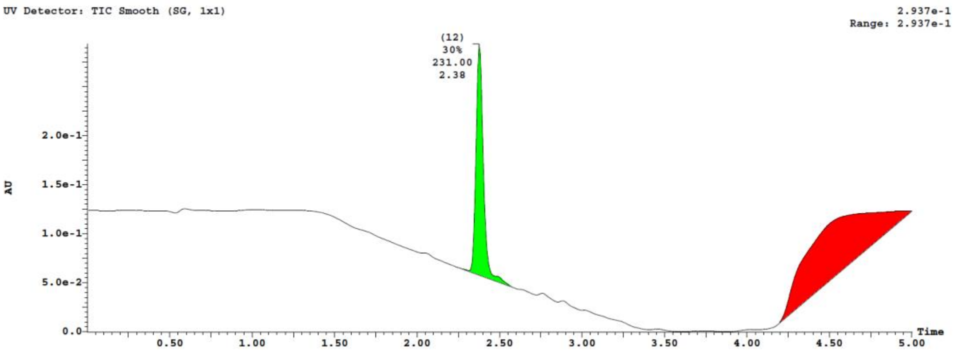

Acquisition Time (sec)	4.0894	***Comment***	OC_037_A_H1	***Date***	30 Jul 2014 21:05:20		
Date Stamp	30 Jul 2014 21:05:20			***File Name***	\\hestia\work\chemistry\NMR Files\NMR_data\OCox\140730_OC_001-037A\21-H1\pdata\1\1r		
Frequency (MHz)	400.13	***Nucleus***	1H	***Number of Transients***	16	***Origin***	Spect
Original Points Count	32768	***Owner***	sgcnmr	***Points Count***	65536	***Pulse Sequence***	zg30
Receiver Gain	194.71	***SW(cyclical) (Hz)***	8012.82	***Solvent***	CHLOROFORM-d		
Spectrum Offset (Hz)	2435.8118	***Spectrum Type***	STANDARD	***Sweep Width (Hz)***	8012.70	***Temperature (degree C)***	25.152

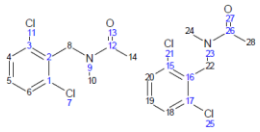

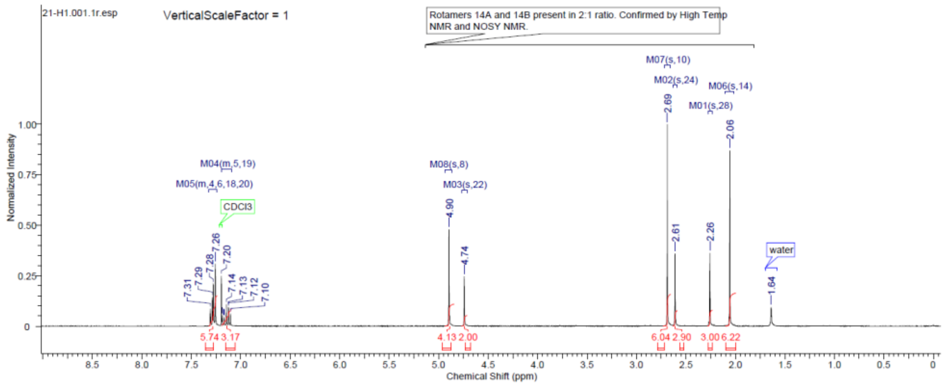

Acquisition Time (sec)	1.3631	***Comment***	OC_037_A_C13			***Date***	30 Jul 2014 21:03:12
Date Stamp	30 Jul 2014 21:03:12			***File Name***	\\hestia\work\chemistry\NMR Files\NMR_data\OCox\140730_OC_001-037A\20-C13\pdata\1\1r		
Frequency (MHz)	100.61	***Nucleus***	13C	***Number of Transients***	1024	***Origin***	Spect
Original Points Count	32768	***Owner***	sgcnmr	***Points Count***	32768	***Pulse Sequence***	jmod
Receiver Gain	194.71	***SW(cyclical) (Hz)***	24038.46	***Solvent***	CHLOROFORM-d		
Spectrum Offset (Hz)	10060.3320	***Spectrum Type***	APT	***Sweep Width (Hz)***	24037.73	***Temperature (degree C)***	25.160

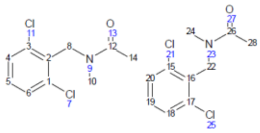

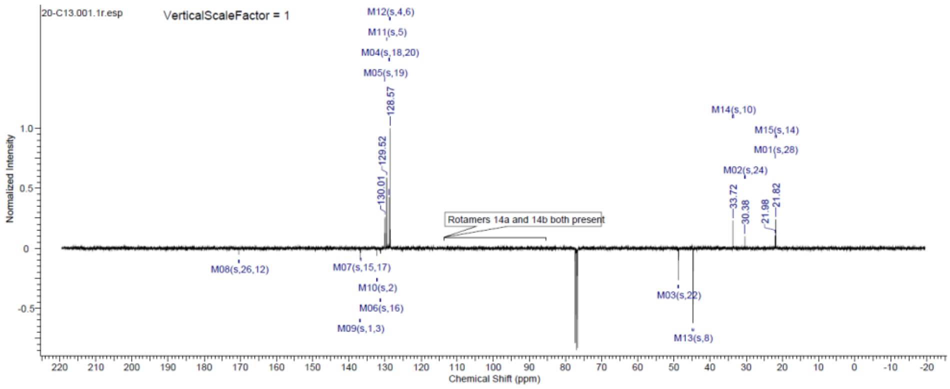

Compound **14** continued

Acquisition Time (sec)	7.9299	*Comment*	OC 037A DMSOH1			*Date*	22 Aug 2014 10:29:36
Date Stamp	22 Aug 2014 10:29:36			*File Name*	\\hestia\work\chemistry\NMR Files\NMR data\OCox\140822 OC 001-037A DMSO\11\pdata\1\1r		
Frequency (MHz)	400.13	*Nucleus*	1H	*Number of Transients*	16	*Origin*	Spect
Original Points Count	65536	*Owner*	sgcnmr	*Points Count*	131072	*Pulse Sequence*	zg60
Receiver Gain	141.83	*SW(cyclical) (Hz)*	8264.46	*Solvent*	DMSO-d6	*Spectrum Offset (Hz)*	2479.3008
Spectrum Type	STANDARD	*Sweep Width (Hz)*	8264.40	*Temperature (degree C)*	70.130		

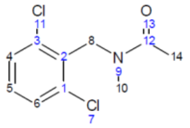

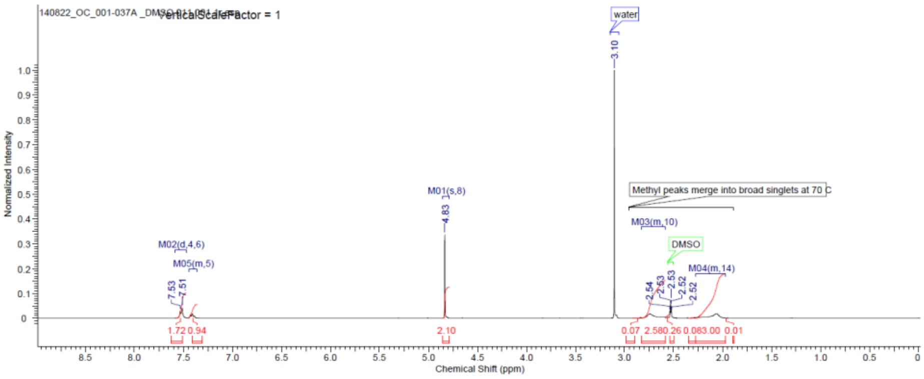

140814_OC_001-037A_NOE.011.001.2rr.esp

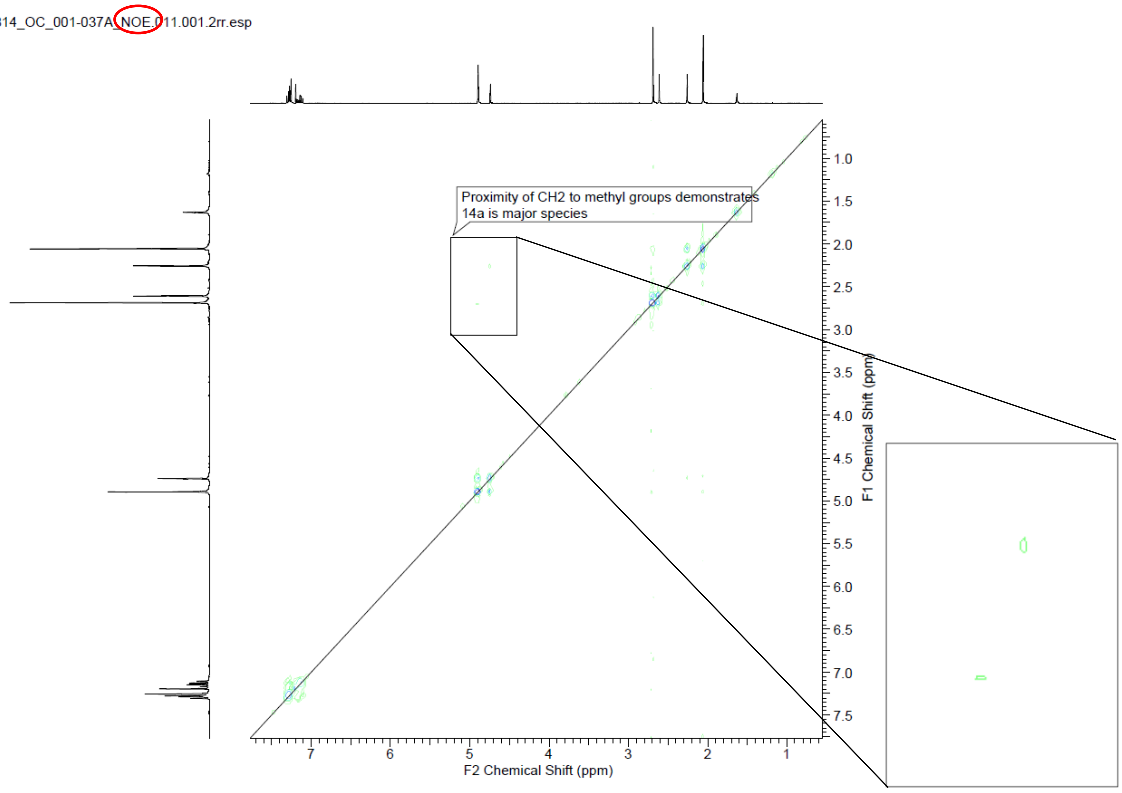

Compound **15**

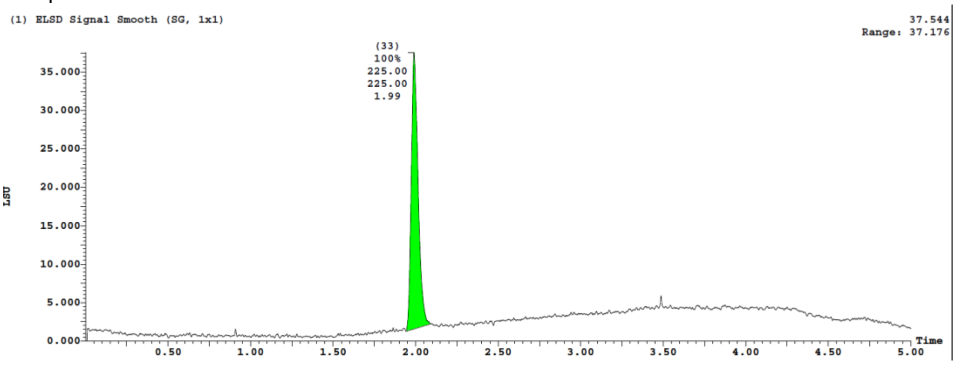

Acquisition Time (sec)	4.0894	Comment	OC_067	Date	26 Jan 2015 16:49:04		
Date Stamp	26 Jan 2015 16:49:04			File Name	\\hestia\work\chemistry\NMR Files\NMR_data\OCox\150126_OC_001-067\10\pdata\1\1r		
Frequency (MHz)	400.13	Nucleus	1H	Number of Transients	16	Origin	Spect
Original Points Count	32768	Owner	sgcnmr	Points Count	65536	Pulse Sequence	zg30
Receiver Gain	194.71	SW(cyclical) (Hz)	8012.82	Solvent	CHLOROFORM-d		
Spectrum Offset (Hz)	2470.9668	Spectrum Type	STANDARD	Sweep Width (Hz)	8012.70	Temperature (degree C)	25.158

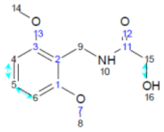

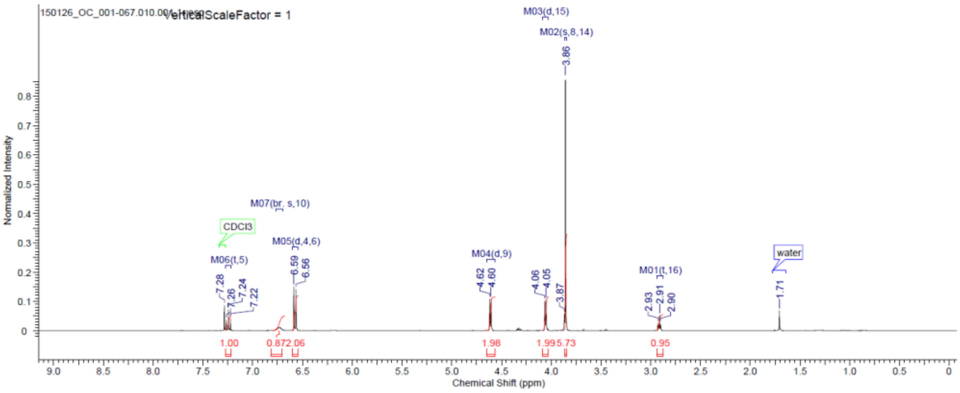

Acquisition Time (sec)	1.3631	Comment	OC_067_C13	Date	27 Jan 2015 04:16:00		
Date Stamp	27 Jan 2015 04:16:00			File Name	\\hestia\work\chemistry\NMR Files\NMR_data\OCox\150126_OC_001-067\11-C13\pdata\1\1r		
Frequency (MHz)	100.61	Nucleus	13C	Number of Transients	1024	Origin	Spect
Original Points Count	32768	Owner	sgcnmr	Points Count	32768	Pulse Sequence	jmod
Receiver Gain	194.71	SW(cyclical) (Hz)	24038.46	Solvent	CHLOROFORM-d		
Spectrum Offset (Hz)	10060.3320	Spectrum Type	APT	Sweep Width (Hz)	24037.73	Temperature (degree C)	25.159

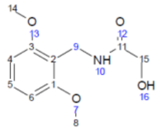

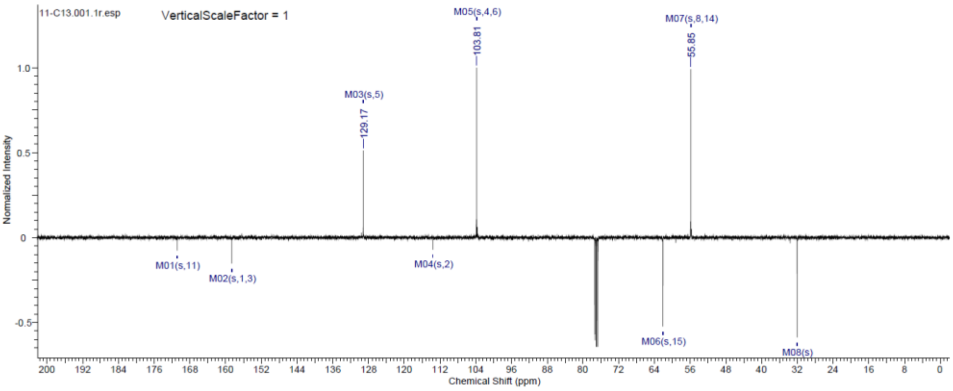

Compound **16**

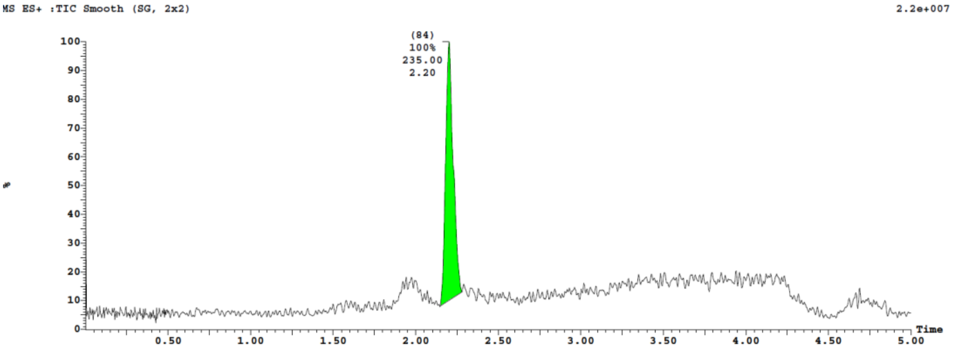

Acquisition Time (sec)	4.0894	Comment	OC_060_A	Date	21 Jan 2015 17:04:00		
Date Stamp	21 Jan 2015 17:04:00			File Name	\\hestia\work\chemistry\NMR Files\NMR_data\OCox\150121_OC_001-060A\10\pdata\1\1r		
Frequency (MHz)	400.13	Nucleus	1H	Number of Transients	16	Origin	Spect
Original Points Count	32768	Owner	sgcnmr	Points Count	65536	Pulse Sequence	zg30
Receiver Gain	194.71	SW(cyclical) (Hz)	8012.82	Solvent	CHLOROFORM-d		
Spectrum Offset (Hz)	2434.1472	Spectrum Type	STANDARD	Sweep Width (Hz)	8012.70	Temperature (degree C)	25.157

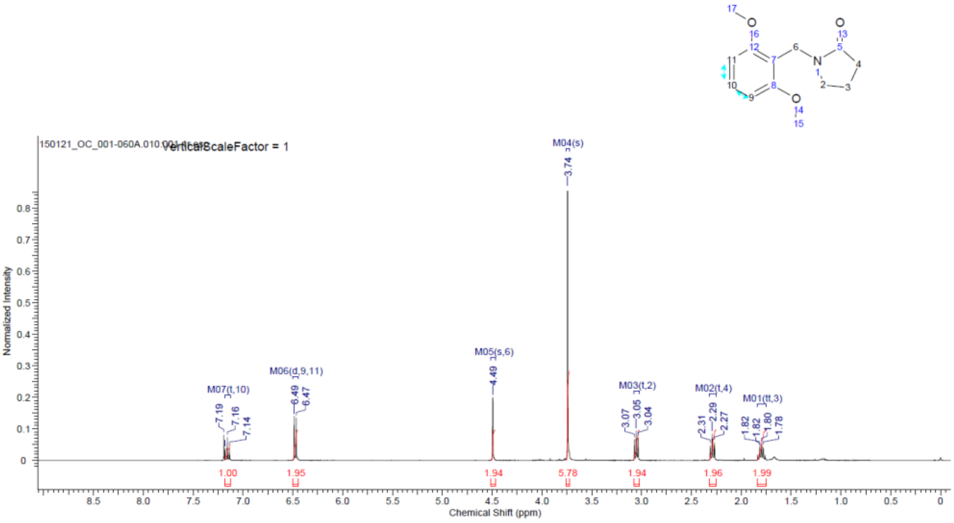

Acquisition Time (sec)	1.3631	Comment	OC_060_A_C13			Date	22 Jan 2015 00:10:40
Date Stamp	22 Jan 2015 00:10:40			File Name	\\hestia\work\chemistry\NMR Files\NMR_data\OCox\150121_OC_001-060A\11-C13\pdata\1\1r		
Frequency (MHz)	100.61	Nucleus	13C	Number of Transients	1024	Origin	Spect
Original Points Count	32768	Owner	sgcnmr	Points Count	32768	Pulse Sequence	jmod
Receiver Gain	194.71	SW(cyclical) (Hz)	24038.46	Solvent	CHLOROFORM-d		
Spectrum Offset (Hz)	10060.3320	Spectrum Type	APT	Sweep Width (Hz)	24037.73	Temperature (degree C)	25.541

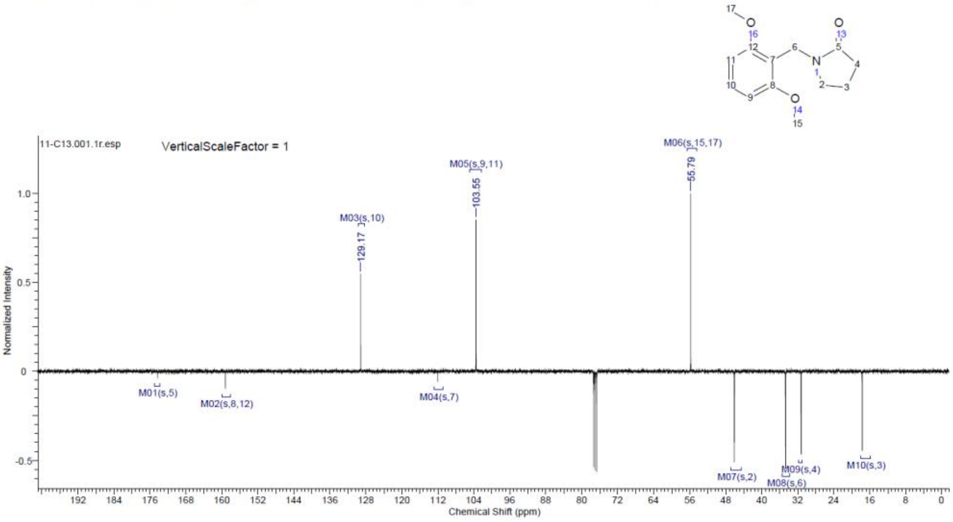

Compound **17**

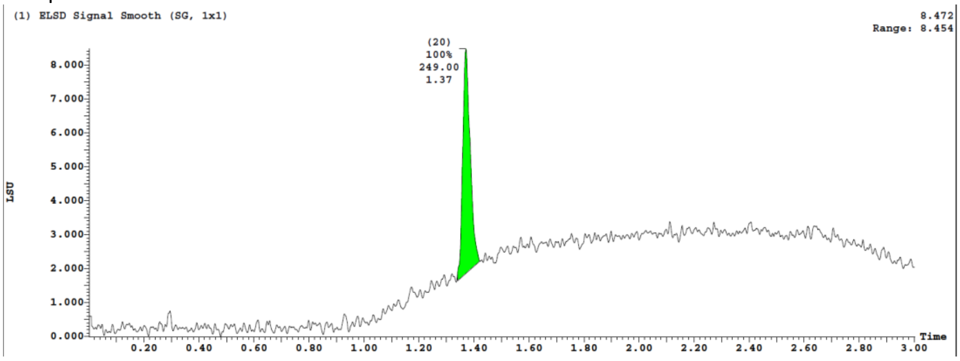

Acquisition Time (sec)	4.0894	Comment	OC_068_B	Date	12 Feb 2015 09:10:24		
Date Stamp	12 Feb 2015 09:10:24			File Name	\\hestia\work\chemistry\NMR Files\NMR_data\OCox\150212_OC_001-068B column2\10\pdata\1\1r		
Frequency (MHz)	400.13	Nucleus	1H	Number of Transients	16	Origin	Spect
Original Points Count	32768	Owner	sgcnmr	Points Count	65536	Pulse Sequence	zg30
Receiver Gain	155.34	SW(cyclical) (Hz)	8012.82	Solvent	CHLOROFORM-d		
Spectrum Offset (Hz)	2470.9668	Spectrum Type	STANDARD	Sweep Width (Hz)	8012.70	Temperature (degree C)	25.157

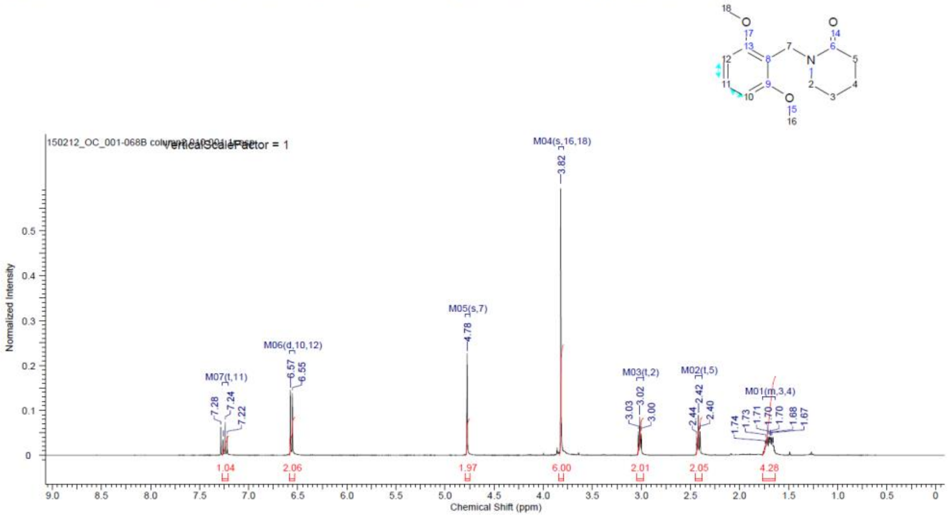

Acquisition Time (sec)	1.3631	Comment	OC_068_B_C13	Date	12 Feb 2015 21:02:56		
Date Stamp	12 Feb 2015 21:02:56						
File Name	\\hestia\work\chemistry\NMR Files\NMR_data\OCox\150212_OC_001-068B column2\11-C13\pdata\1\1r					Frequency (MHz)	100.61
Nucleus	13C	Number of Transients	1024	Origin	Spect	Original Points Count	32768
Owner	sgcnmr	Points Count	32768	Pulse Sequence	jmod	Receiver Gain	194.71
SW(cyclical) (Hz)	24038.46	Solvent	CHLOROFORM-d			Spectrum Offset (Hz)	10060.3320
Spectrum Type	APT	Sweep Width (Hz)	24037.73	Temperature (degree C)	25.162		

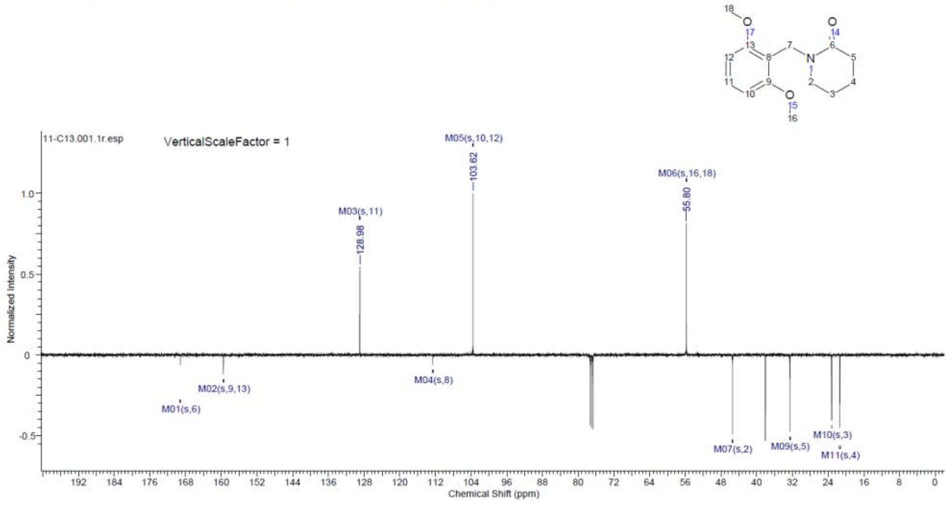

Compound **19**

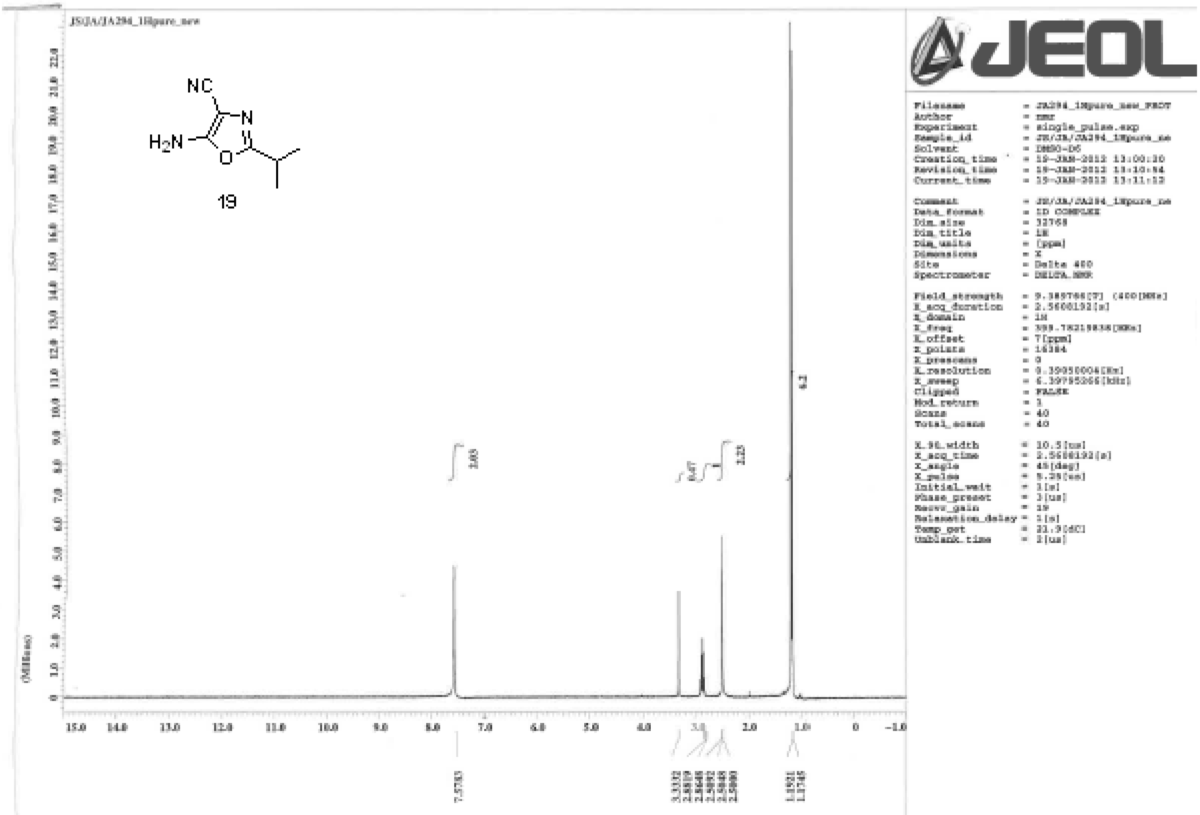

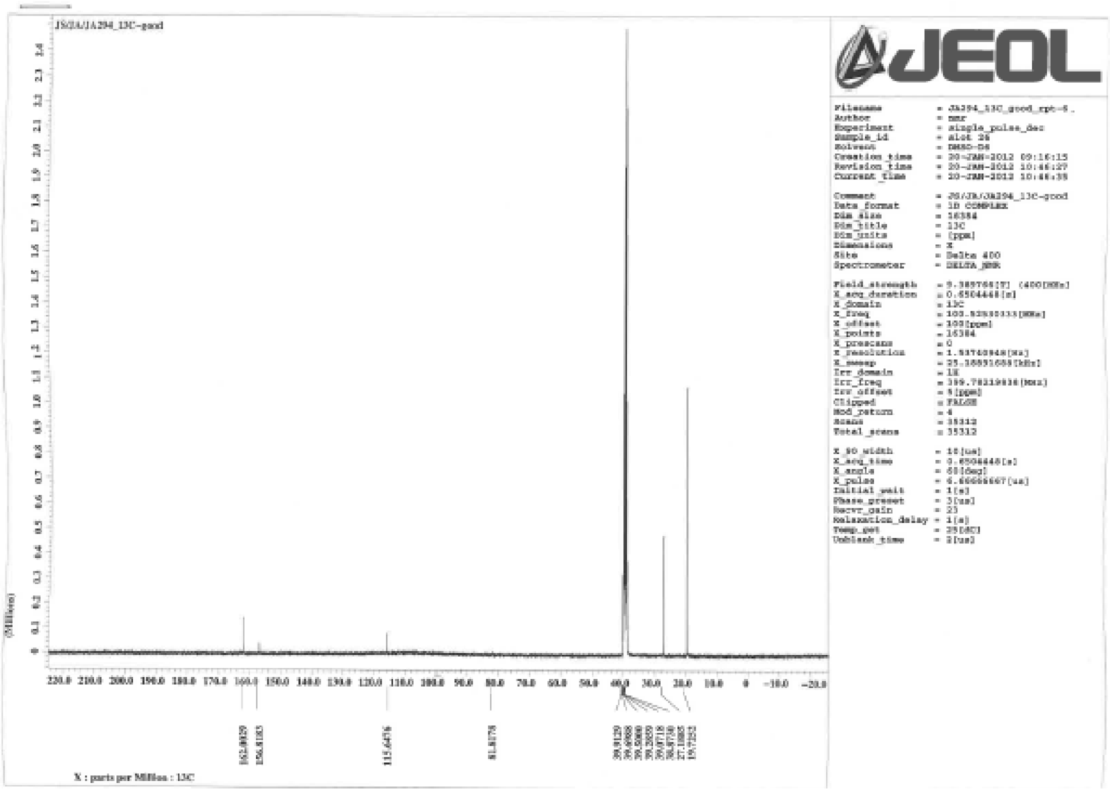

Compound **20**

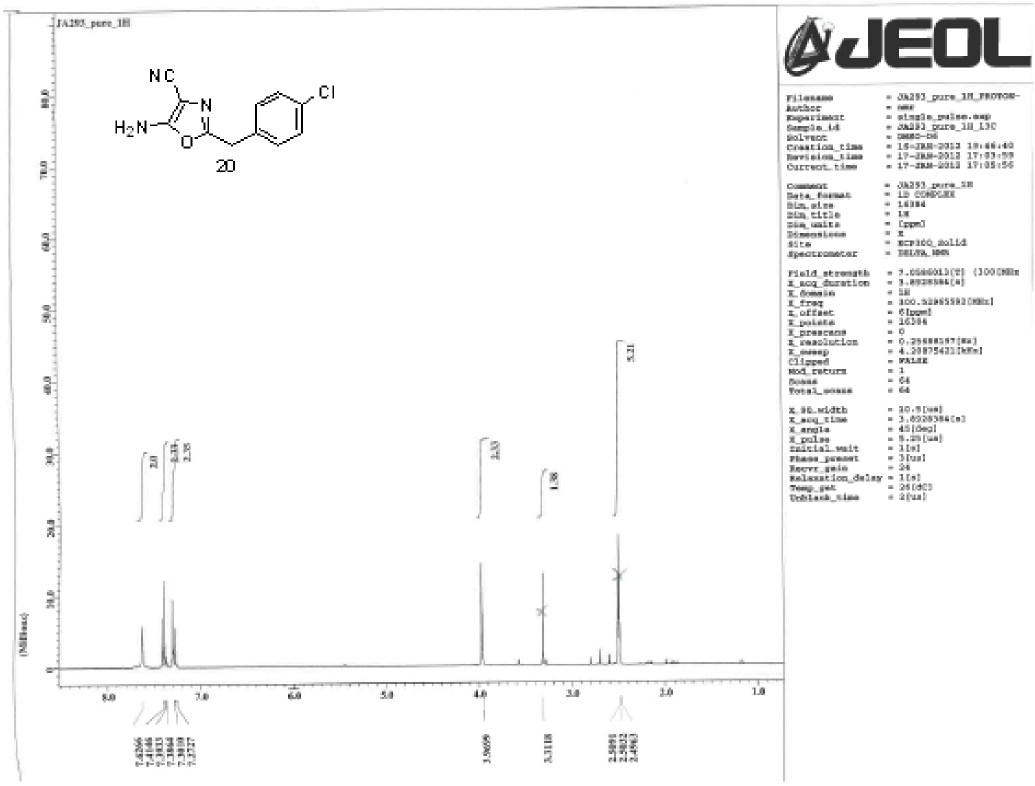

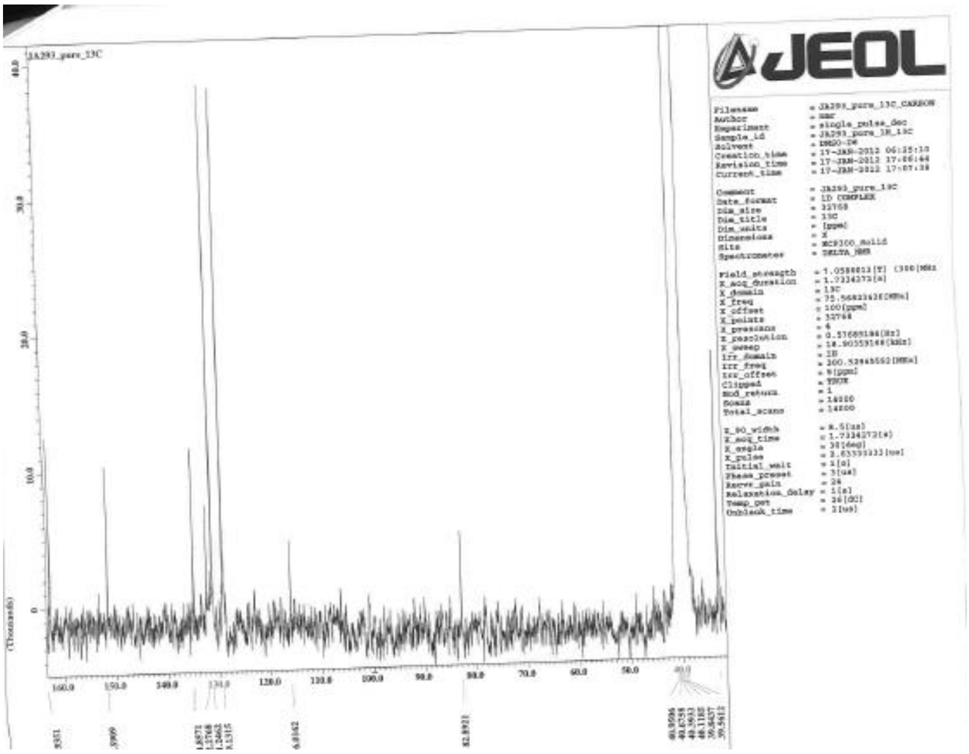

Compound **s1**

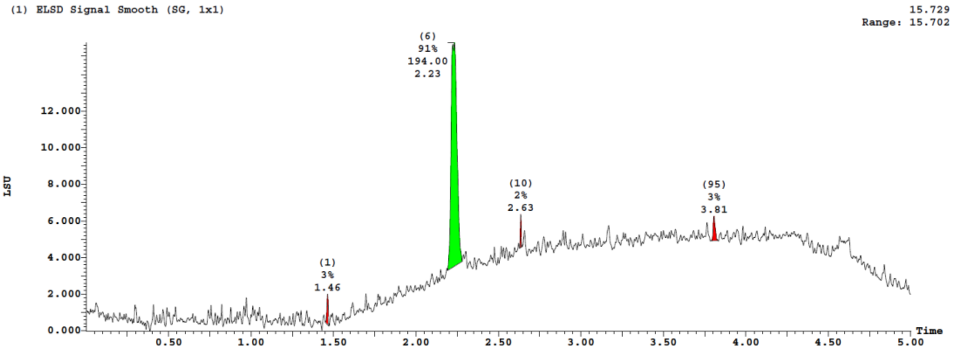

Acquisition Time (sec)	4.0894	Comment	OC 040 F H1	Date	09 Aug 2014 01:19:12		
Date Stamp	09 Aug 2014 01:19:12			File Name	\\hestia\work\chemistry\NMR Files\NMR data\OCox\140808 OC 001-040F\12-H1\pdata\1\1r		
Frequency (MHz)	400.13	Nucleus	1H	Number of Transients	16	Origin	Spect
Original Points Count	32768	Owner	sgcnmr	Points Count	65536	Pulse Sequence	zg30
Receiver Gain	194.71	SW(cyclical) (Hz)	8012.82	Solvent	CHLOROFORM-d		
Spectrum Offset (Hz)	2470.9668	Spectrum Type	STANDARD	Sweep Width (Hz)	8012.70	Temperature (degree C)	25.149

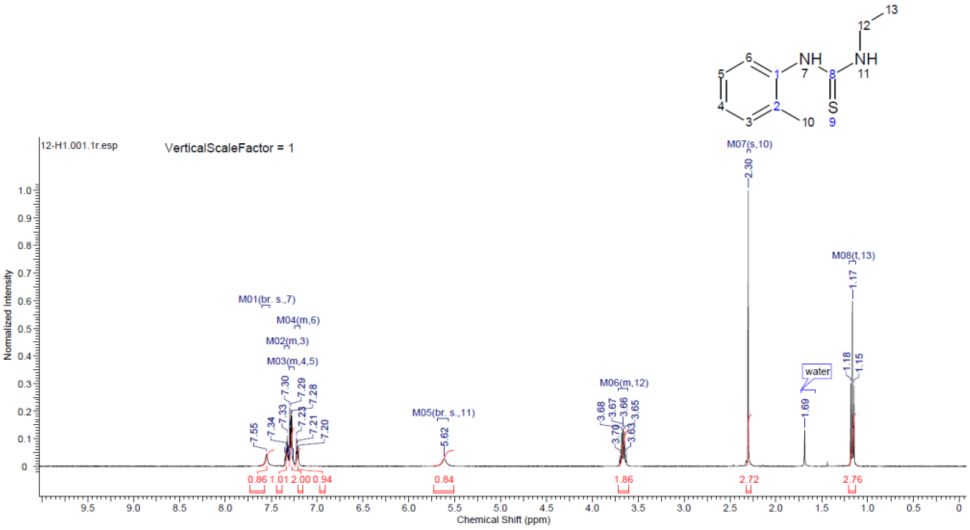

Acquisition Time (sec)	1.3631	Comment	OC 040 F C13			Date	09 Aug 2014 01:17:04
Date Stamp	09 Aug 2014 01:17:04			File Name	\\hestia\work\chemistry\NMR Files\NMR data\OCox\140808 OC 001-040F\11-C13\pdata\1\1r		
Frequency (MHz)	100.61	Nucleus	13C	Number of Transients	1024	Origin	Spect
Original Points Count	32768	Owner	sgcnmr	Points Count	32768	Pulse Sequence	jmod
Receiver Gain	194.71	SW(cyclical) (Hz)	24038.46	Solvent	CHLOROFORM-d		
Spectrum Offset (Hz)	10060.3320	Spectrum Type	APT	Sweep Width (Hz)	24037.73	Temperature (degree C)	25.161

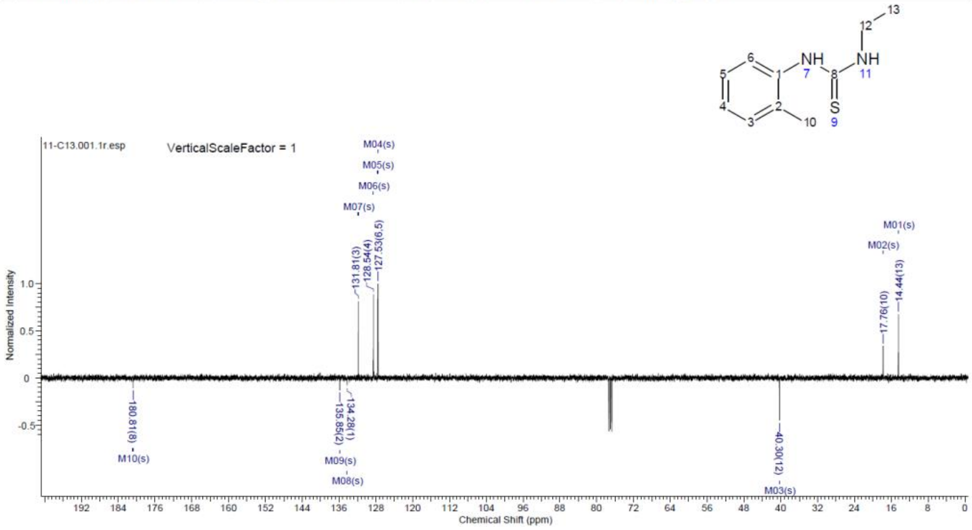

Compound **s2**

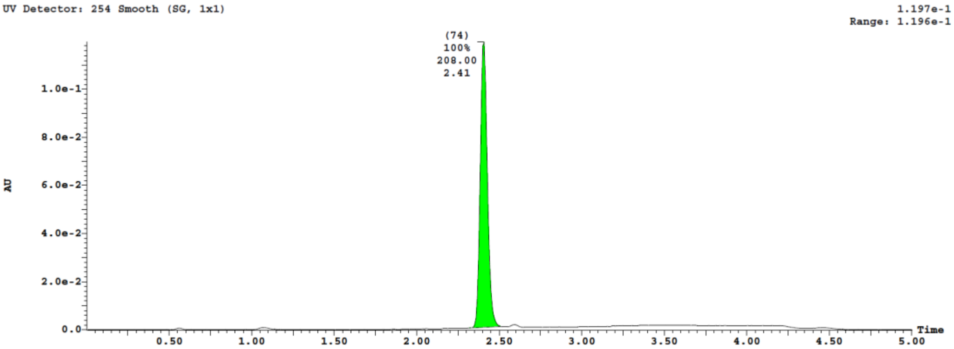

Acquisition Time (sec)	4.0894	Comment	OC_040_E_H1	Date	08 Aug 2014 23:13:20		
Date Stamp	08 Aug 2014 23:13:20			File Name	\\hestia\work\chemistry\NMR Files\NMR_data\OCox\140809_OC_001-040E\12-H1\pdata\1\1r		
Frequency (MHz)	400.13	Nucleus	1H	Number of Transients	16	Origin	Spect
Original Points Count	32768	Owner	sgcnmr	Points Count	65536	Pulse Sequence	zg30
Receiver Gain	194.71	SW(cyclical) (Hz)	8012.82	Solvent	CHLOROFORM-d		
Spectrum Offset (Hz)	2470.9668	Spectrum Type	STANDARD	Sweep Width (Hz)	8012.70	Temperature (degree C)	25.151

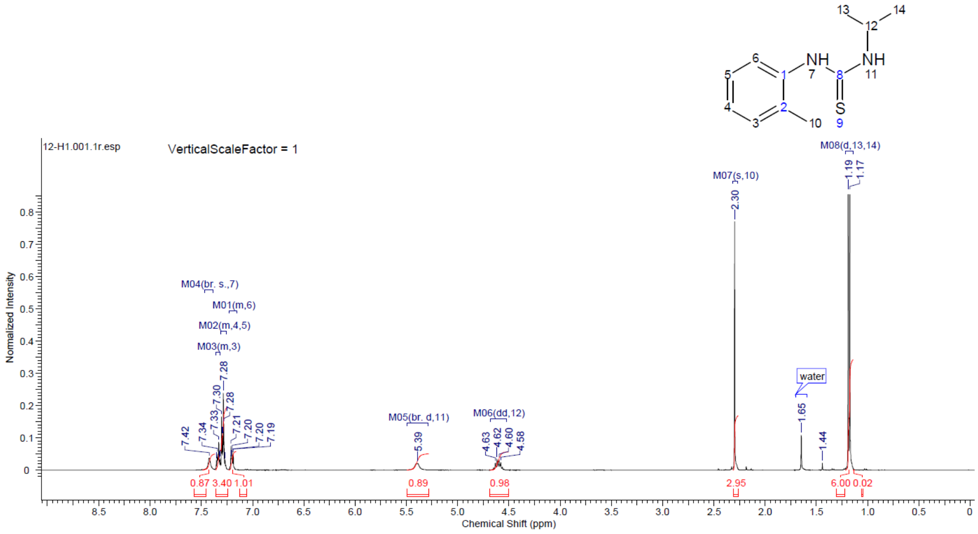

Acquisition Time (sec)	1.3631	Comment	OC_040_E_C13			Date	08 Aug 2014 23:11:12
Date Stamp	08 Aug 2014 23:11:12			File Name	\\hestia\work\chemistry\NMR Files\NMR_data\OCox\140809_OC_001-040E\11-C13\pdata\1\1r		
Frequency (MHz)	100.61	Nucleus	13C	Number of Transients	1024	Origin	Spect
Original Points Count	32768	Owner	sgcnmr	Points Count	32768	Pulse Sequence	jmod
Receiver Gain	194.71	SW(cyclical) (Hz)	24038.46	Solvent	CHLOROFORM-d		
Spectrum Offset (Hz)	10060.3320	Spectrum Type	APT	Sweep Width (Hz)	24037.73	Temperature (degree C)	25.161

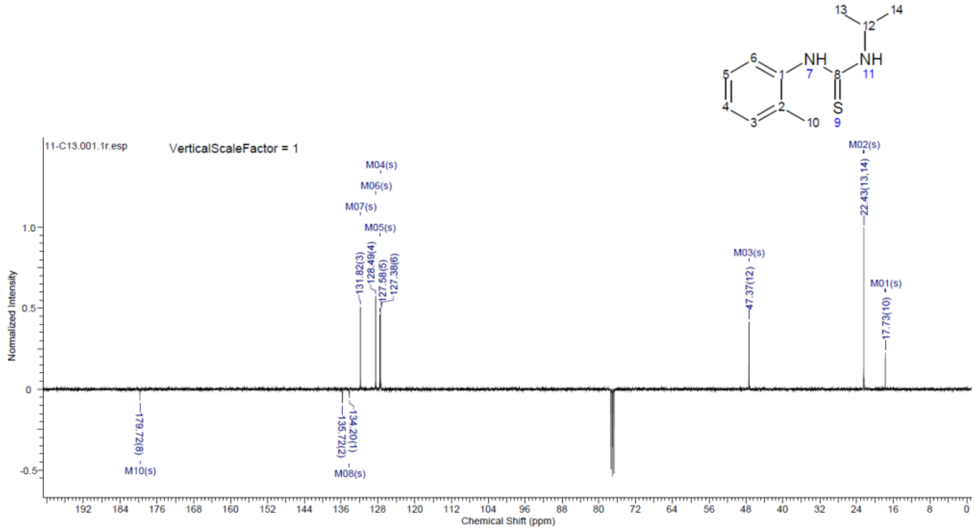

Compound **s3**

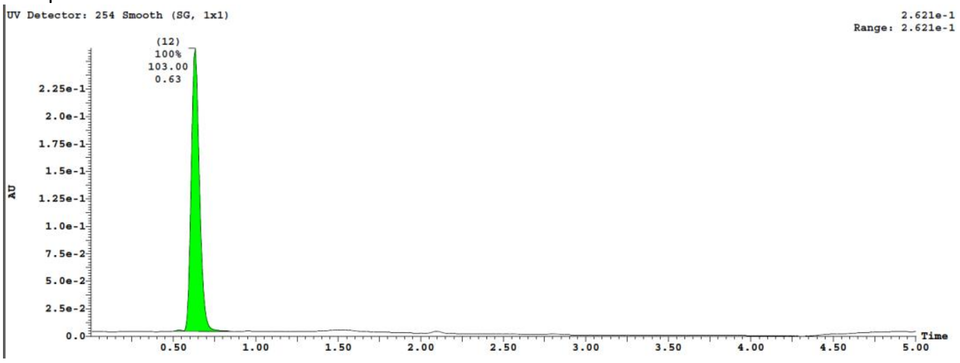

Acquisition Time (sec)	4.0894	***Comment***	OC 031 A H1	***Date***	12 Jul 2014 01:00:00		
Date Stamp	12 Jul 2014 01:00:00			***File Name***	\\hestia\work\chemistry\NMR Files\NMR data\OCox\140710 OC 001-031A\12 H1\pdata\1\1r		
Frequency (MHz)	400.13	***Nucleus***	1H	***Number of Transients***	16	***Origin***	Spect
Original Points Count	32768	***Owner***	sgcnmr	***Points Count***	65536	***Pulse Sequence***	zg30
Receiver Gain	194.71	***SW(cyclical) (Hz)***	8012.82	***Solvent***	DMSO-d6	***Spectrum Offset (Hz)***	2467.8220
Spectrum Type	STANDARD	***Sweep Width (Hz)***	8012.70	***Temperature (degree C)***	25.156		

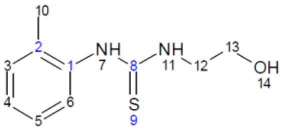

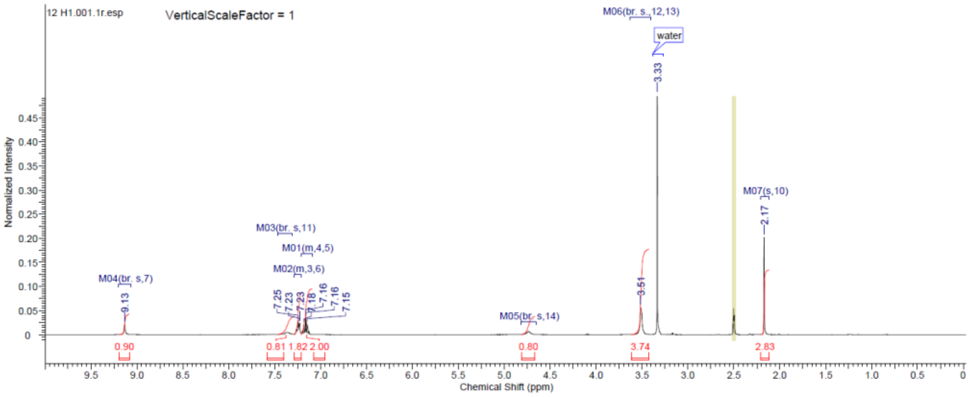

Acquisition Time (sec)	1.3631	***Comment***	OC 031 A C13			***Date***	12 Jul 2014 00:38:40
Date Stamp	12 Jul 2014 00:38:40			***File Name***	\\hestia\work\chemistry\NMR Files\NMR data\OCox\140710 OC 001-031A\10 C13\pdata\1\1r		
Frequency (MHz)	100.61	***Nucleus***	13C	***Number of Transients***	1024	***Origin***	Spect
Original Points Count	32768	***Owner***	sgcnmr	***Points Count***	32768	***Pulse Sequence***	zgpg30
Receiver Gain	194.71	***SW(cyclical) (Hz)***	24038.46	***Solvent***	DMSO-d6	***Spectrum Offset (Hz)***	10059.8594
Spectrum Type	STANDARD	***Sweep Width (Hz)***	24037.73	***Temperature (degree C)***	25.160		

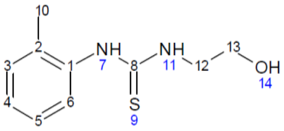

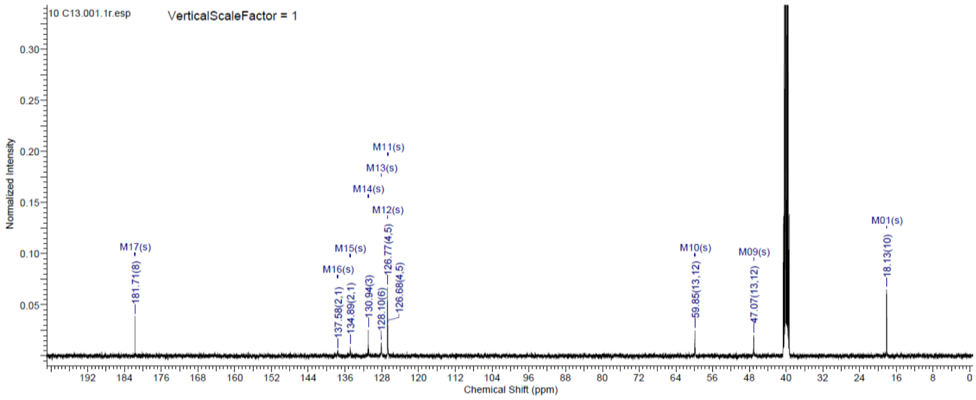

Compound **s4**

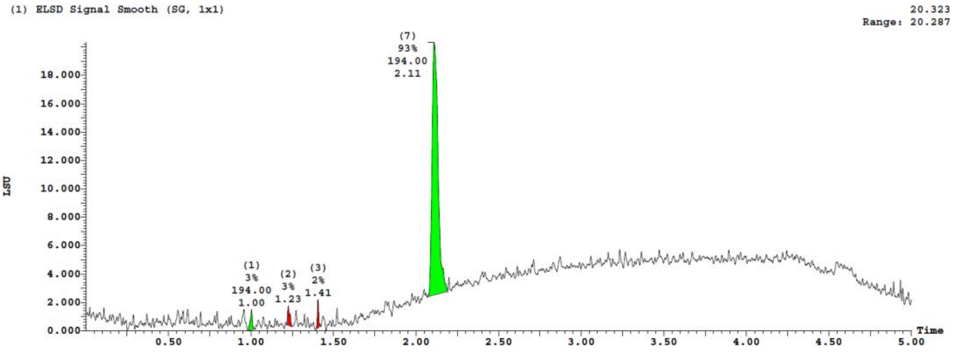

Acquisition Time (sec)	4.0894	Comment	OC_040_G_H1	Date	07 Aug 2014 21:05:20		
Date Stamp	07 Aug 2014 21:05:20			File Name	\\hestia\work\chemistry\NMR Files\NMR_data\OCox\140807_OC_001-040G\12-H1\pdata\1\1r		
Frequency (MHz)	400.13	Nucleus	1H	Number of Transients	16	Origin	Spect
Original Points Count	32768	Owner	sgcnmr	Points Count	65536	Pulse Sequence	zg30
Receiver Gain	77.20	SW(cyclical) (Hz)	8012.82	Solvent	DMSO-d6	Spectrum Offset (Hz)	2470.9668
Spectrum Type	STANDARD	Sweep Width (Hz)	8012.70	Temperature (degree C)	25.143		

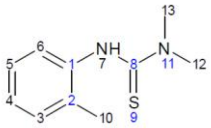

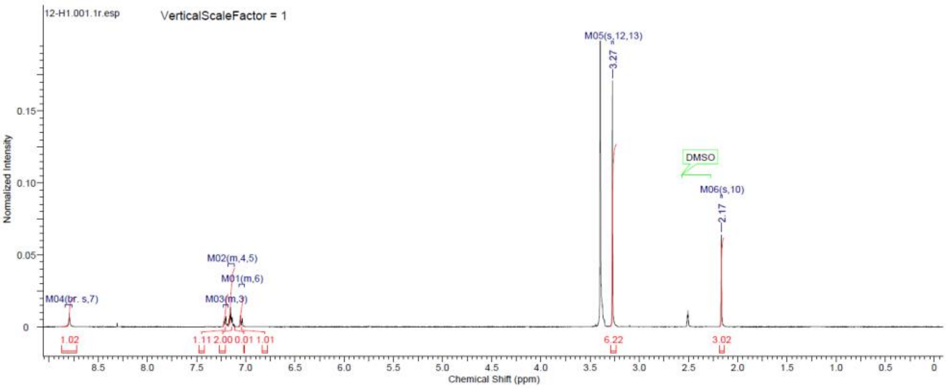

Acquisition Time (sec)	1.3631	Comment	OC_040_G_C13			Date	07 Aug 2014 21:03:12
Date Stamp	07 Aug 2014 21:03:12			File Name	\\hestia\work\chemistry\NMR Files\NMR_data\OCox\140807_OC_001-040G\11-C13\pdata\1\1r		
Frequency (MHz)	100.61	Nucleus	13C	Number of Transients	1024	Origin	Spect
Original Points Count	32768	Owner	sgcnmr	Points Count	32768	Pulse Sequence	jmod
Receiver Gain	194.71	SW(cyclical) (Hz)	24038.46	Solvent	DMSO-d6	Spectrum Offset (Hz)	10060.3320
Spectrum Type	APT	Sweep Width (Hz)	24037.73	Temperature (degree C)	25.161		

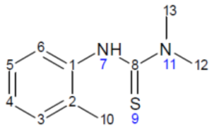

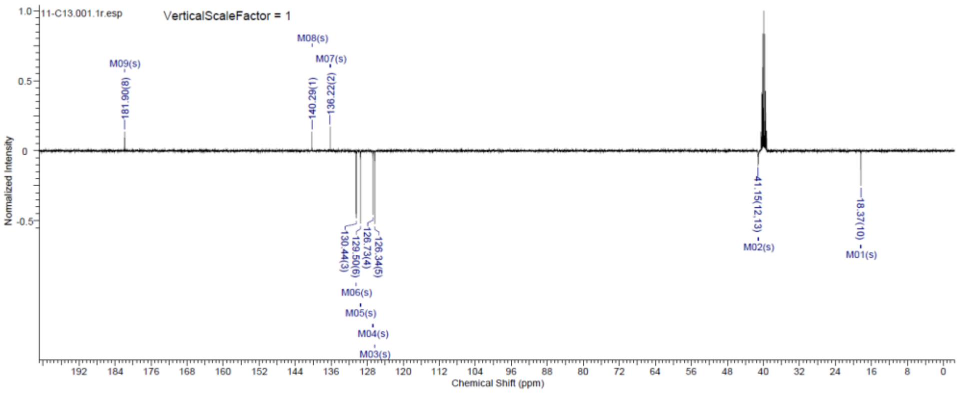

Compound **s5**

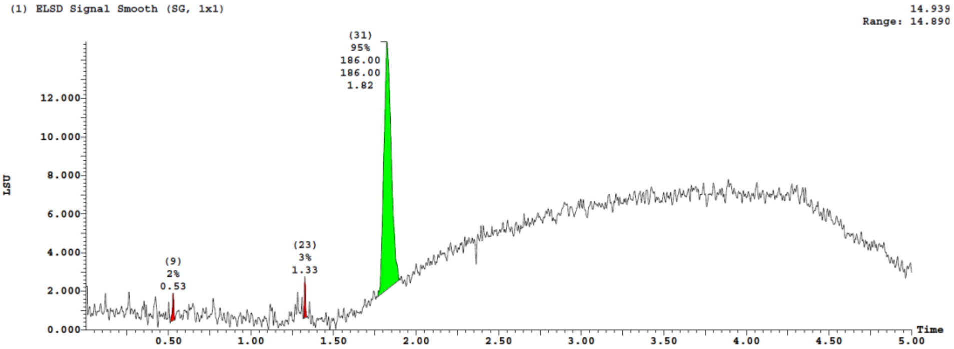

Acquisition Time (sec)	4.0894	*Comment*	OC_053_D_H1	*Date*	12 Dec 2014 16:40:32		
Date Stamp	12 Dec 2014 16:40:32			*File Name*	\\hestia\work\chemistry\NMR Files\NMR_data\OCox\141212_OC_001-053D\10-H1\pdata\1\1r		
Frequency (MHz)	400.13	*Nucleus*	1H	*Number of Transients*	16	*Origin*	Spect
Original Points Count	32768	*Owner*	sgcnmr	*Points Count*	65536	*Pulse Sequence*	zg30
Receiver Gain	194.71	*SW(cyclical) (Hz)*	8012.82	*Solvent*	DMSO-d6	*Spectrum Offset (Hz)*	2470.9668
Spectrum Type	STANDARD	*Sweep Width (Hz)*	8012.70	*Temperature (degree C)*	25.159		

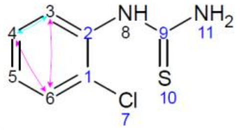

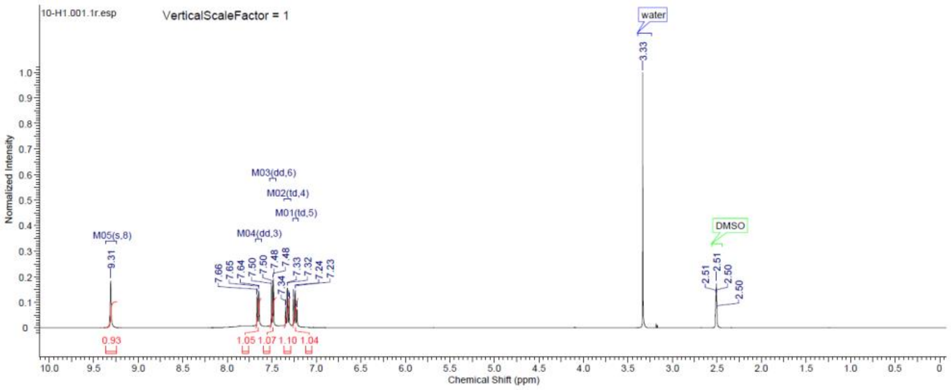

Acquisition Time (sec)	1.3631	*Comment*	OC_053_D_C13			*Date*	13 Dec 2014 08:10:40
Date Stamp	13 Dec 2014 08:10:40			*File Name*	\\hestia\work\chemistry\NMR Files\NMR_data\OCox\141212_OC_001-053D\11-C13\pdata\1\1r		
Frequency (MHz)	100.61	*Nucleus*	13C	*Number of Transients*	1024	*Origin*	Spect
Original Points Count	32768	*Owner*	sgcnmr	*Points Count*	32768	*Pulse Sequence*	jmod
Receiver Gain	194.71	*SW(cyclical) (Hz)*	24038.46	*Solvent*	DMSO-d6	*Spectrum Offset (Hz)*	10060.3320
Spectrum Type	APT	*Sweep Width (Hz)*	24037.73	*Temperature (degree C)*	25.423		

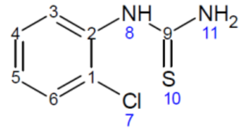

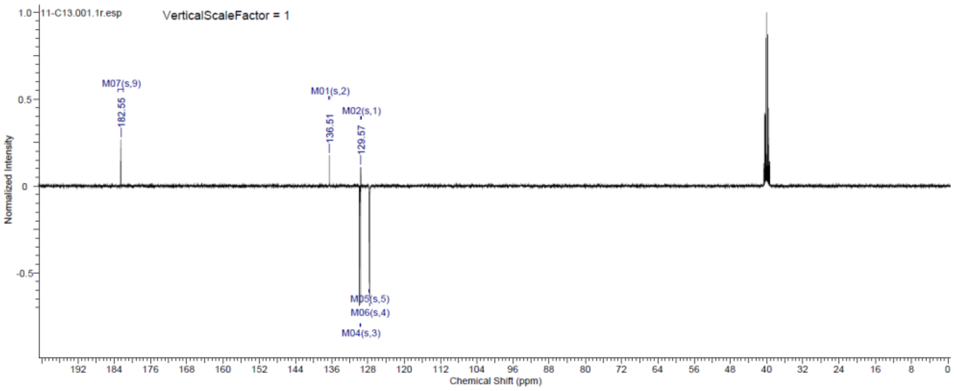

Compound **s6**

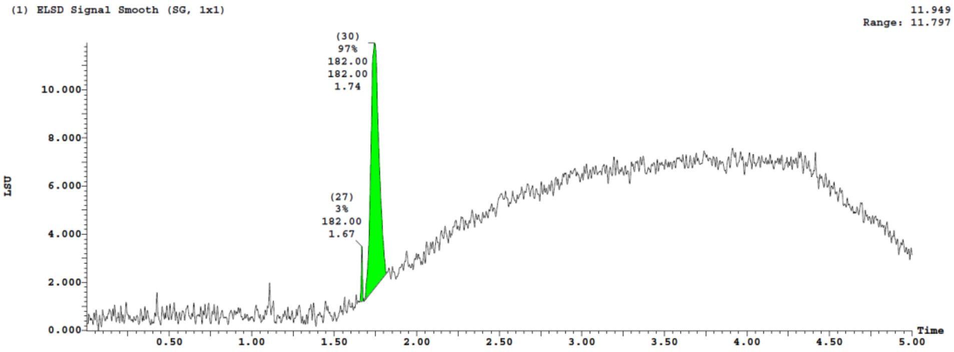

Acquisition Time (sec)	4.0894	***Comment***	OC_053_E	***Date***	05 Dec 2014 12:30:56		
Date Stamp	05 Dec 2014 12:30:56			***File Name***	\\hestia\work\chemistry\NMR Files\NMR_data\OCox\141205_OC_001-053E\10-H1\pdata\1\1r		
Frequency (MHz)	400.13	***Nucleus***	1H	***Number of Transients***	16	***Origin***	Spect
Original Points Count	32768	***Owner***	sgcnmr	***Points Count***	65536	***Pulse Sequence***	zg30
Receiver Gain	194.71	***SW(cyclical) (Hz)***	8012.82	***Solvent***	DMSO-d6	***Spectrum Offset (Hz)***	2470.9668
Spectrum Type	STANDARD	***Sweep Width (Hz)***	8012.70	***Temperature (degree C)***	25.158		

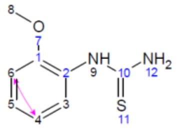

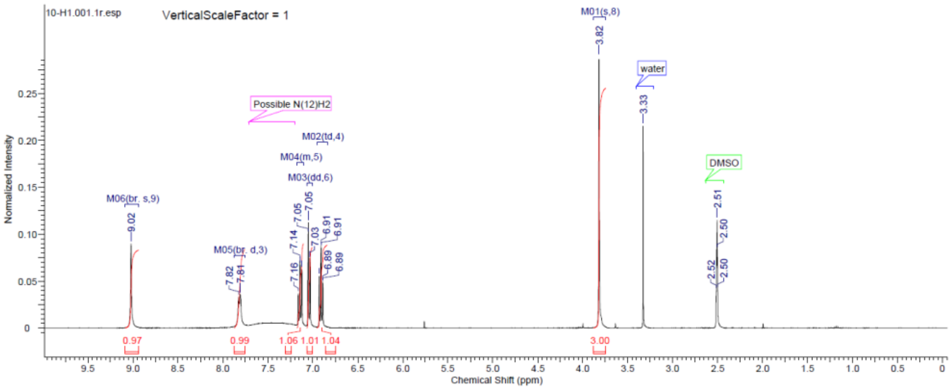

Acquisition Time (sec)	1.3631	***Comment***	OC_053_E_C13			***Date***	06 Dec 2014 10:44:16
Date Stamp	06 Dec 2014 10:44:16			***File Name***	\\hestia\work\chemistry\NMR Files\NMR_data\OCox\141205_OC_001-053E\11-C13\pdata\1\1r		
Frequency (MHz)	100.61	***Nucleus***	13C	***Number of Transients***	1024	***Origin***	Spect
Original Points Count	32768	***Owner***	sgcnmr	***Points Count***	32768	***Pulse Sequence***	jmod
Receiver Gain	194.71	***SW(cyclical) (Hz)***	24038.46	***Solvent***	DMSO-d6	***Spectrum Offset (Hz)***	10060.3320
Spectrum Type	APT	***Sweep Width (Hz)***	24037.73	***Temperature (degree C)***	25.342		

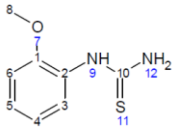

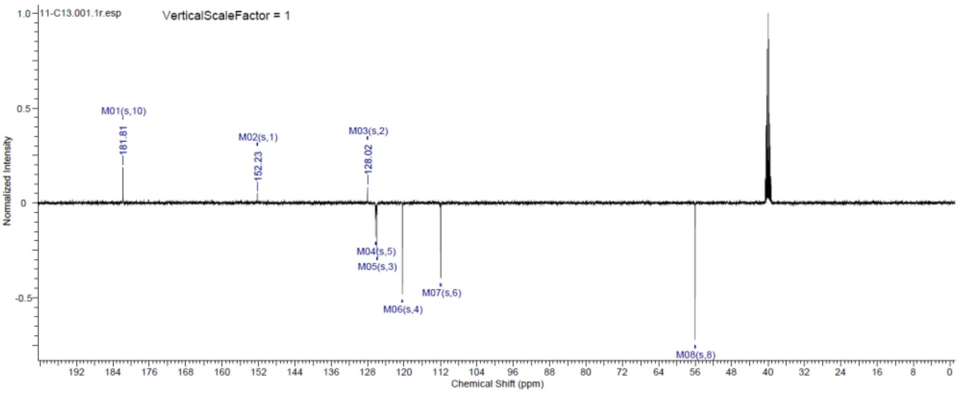

Compound **s8**

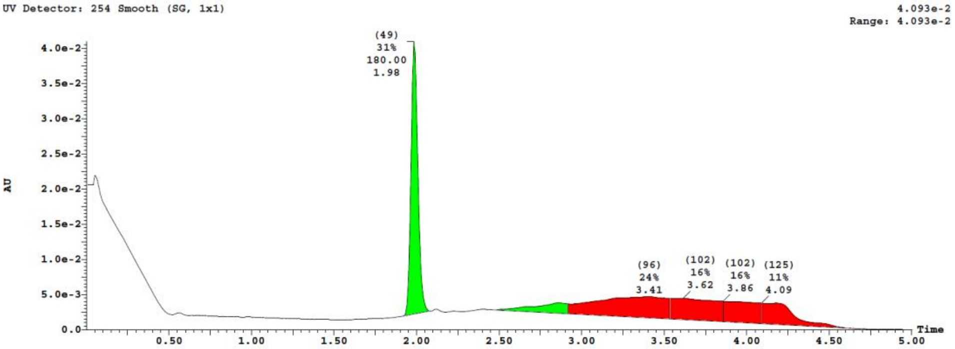

Acquisition Time (sec)	4.0894	*Comment*	OC 039 C H1	*Date*	05 Aug 2014 04:46:08
Date Stamp	05 Aug 2014 04:46:08			*File Name*	\\hestia\work\chemistry\NMR Files\NMR data\OCox\140804 OC 001-039C\12-H1\pdata\1\1r
Frequency (MHz)	400.13	*Nucleus*	1H	*Number of Transients*	16
Original Points Count	32768	*Owner*	sgcnmr	*Points Count*	65536
Receiver Gain	194.71	*SW(cyclical) (Hz)*	8012.82	*Solvent*	DMSO-d6
Spectrum Type	STANDARD	*Sweep Width (Hz)*	8012.70	*Temperature (degree C)*	25.151

Origin	Spect
Pulse Sequence	zg30
Spectrum Offset (Hz)	2470.9668

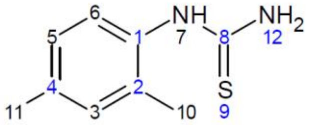

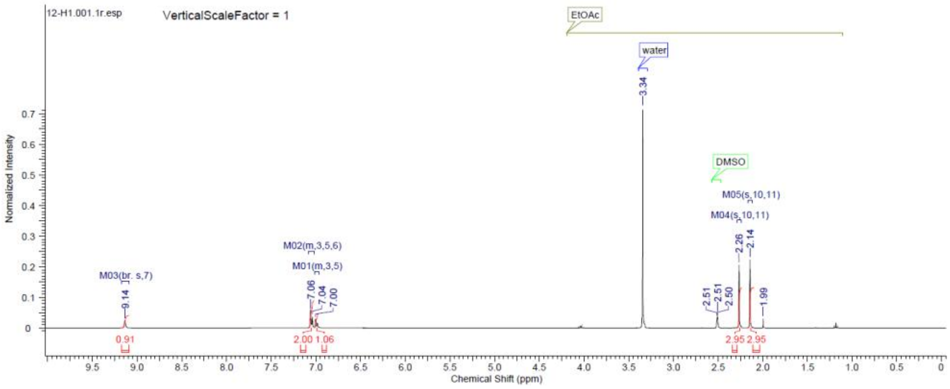

Acquisition Time (sec)	1.3631	*Comment*	OC 039 C C13	*Date*	05 Aug 2014 04:44:00
Date Stamp	05 Aug 2014 04:44:00			*File Name*	\\hestia\work\chemistry\NMR Files\NMR data\OCox\140804 OC 001-039C\11-C13\pdata\1\1r
Frequency (MHz)	100.61	*Nucleus*	13C	*Number of Transients*	1024
Original Points Count	32768	*Owner*	sgcnmr	*Points Count*	32768
Receiver Gain	194.71	*SW(cyclical) (Hz)*	24038.46	*Solvent*	DMSO-d6
Spectrum Type	APT	*Sweep Width (Hz)*	24037.73	*Temperature (degree C)*	25.161

Origin	Spect
Pulse Sequence	jmod
Spectrum Offset (Hz)	10060.3320

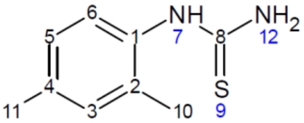

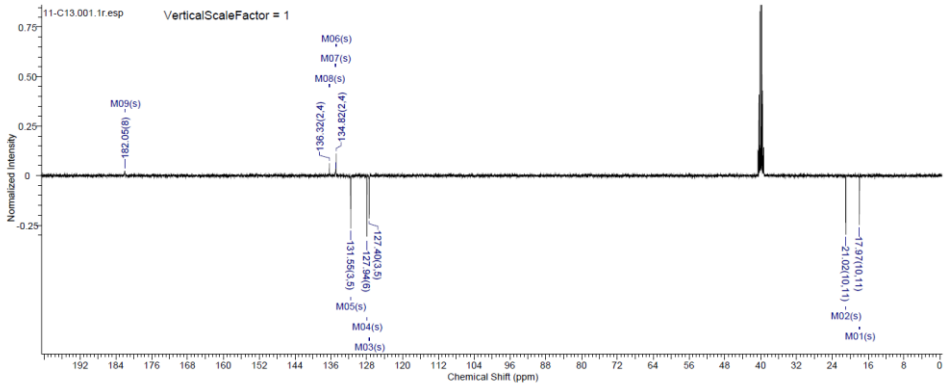

Compound **s9**

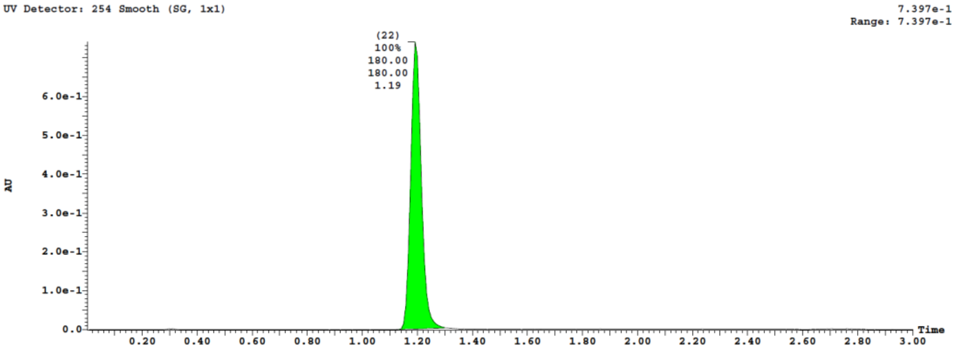

Acquisition Time (sec)	4.0894	Comment	OC 039 D H1	Date	05 Aug 2014 02:40:16		
Date Stamp	05 Aug 2014 02:40:16			File Name	\\hestia\work\chemistry\NMR Files\NMR data\OCox\140804 OC 001-039D\11-H1\pdata\1\1r		
Frequency (MHz)	400.13	Nucleus	1H	Number of Transients	16	Origin	Spect
Original Points Count	32768	Owner	sgcnmr	Points Count	65536	Pulse Sequence	zg30
Receiver Gain	194.71	SW(cyclical) (Hz)	8012.82	Solvent	DMSO-d6	Spectrum Offset (Hz)	2470.9668
Spectrum Type	STANDARD	Sweep Width (Hz)	8012.70	Temperature (degree C)	25.153		

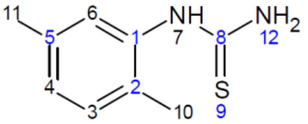

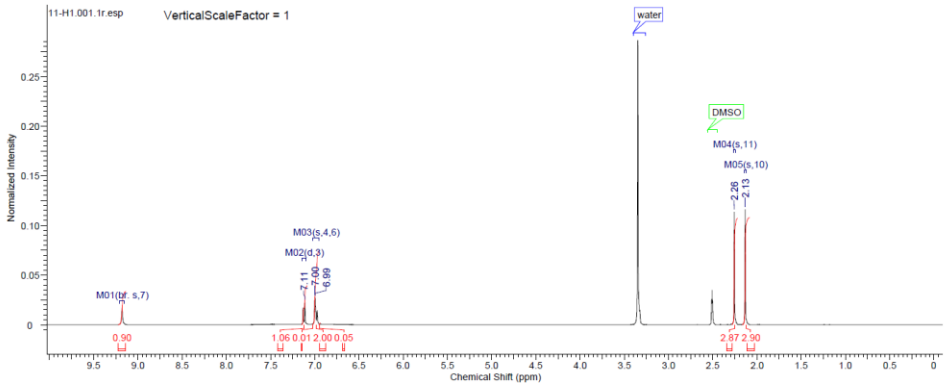

Acquisition Time (sec)	1.3631	Comment	OC 039 D C13			Date	05 Aug 2014 02:38:08
Date Stamp	05 Aug 2014 02:38:08			File Name	\\hestia\work\chemistry\NMR Files\NMR data\OCox\140804 OC 001-039D\15-C13\pdata\1\1r		
Frequency (MHz)	100.61	Nucleus	13C	Number of Transients	1024	Origin	Spect
Original Points Count	32768	Owner	sgcnmr	Points Count	32768	Pulse Sequence	jmod
Receiver Gain	194.71	SW(cyclical) (Hz)	24038.46	Solvent	DMSO-d6	Spectrum Offset (Hz)	10060.3320
Spectrum Type	APT	Sweep Width (Hz)	24037.73	Temperature (degree C)	25.158		

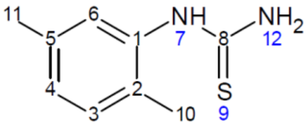

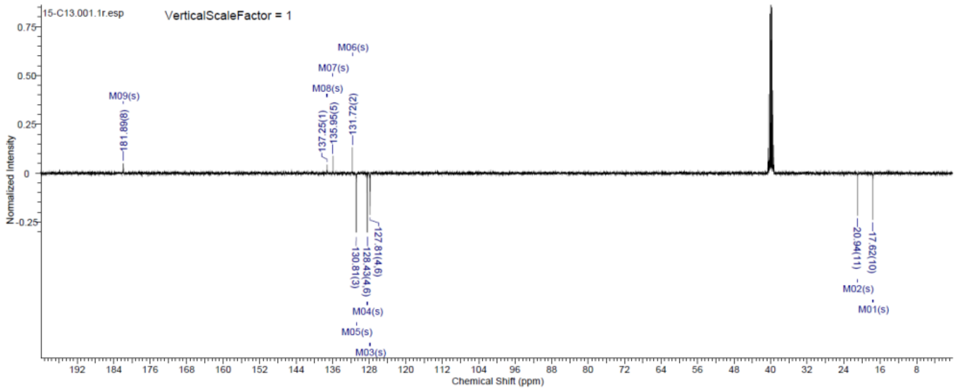

Compound **s10**

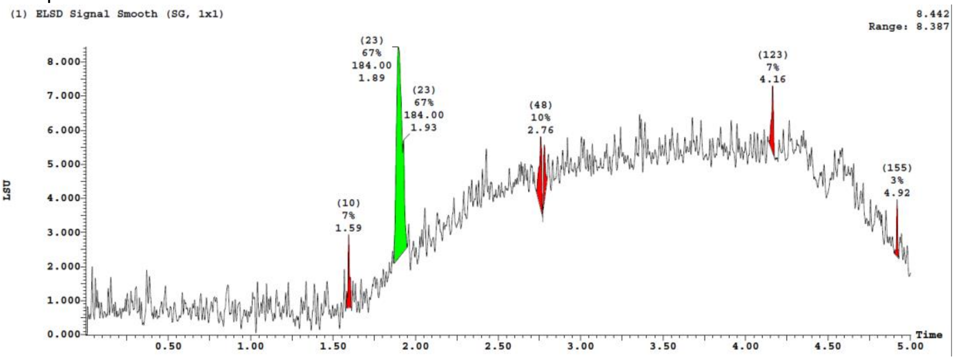

Acquisition Time (sec)	4.0894	Comment	OC_039_F_H1	Date	05 Aug 2014 00:34:24		
Date Stamp	05 Aug 2014 00:34:24			File Name	\\hestia\work\chemistry\NMR Files\NMR_data\OCox\140804_OC_001-039F\21-H1\pdata\1\1r		
Frequency (MHz)	400.13	Nucleus	1H	Number of Transients	16	Origin	Spect
Original Points Count	32768	Owner	sgcnmr	Points Count	65536	Pulse Sequence	zg30
Receiver Gain	194.71	SW(cyclical) (Hz)	8012.82	Solvent	DMSO-d6	Spectrum Offset (Hz)	2470.9668
Spectrum Type	STANDARD	Sweep Width (Hz)	8012.70	Temperature (degree C)	25.152		

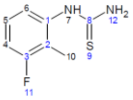

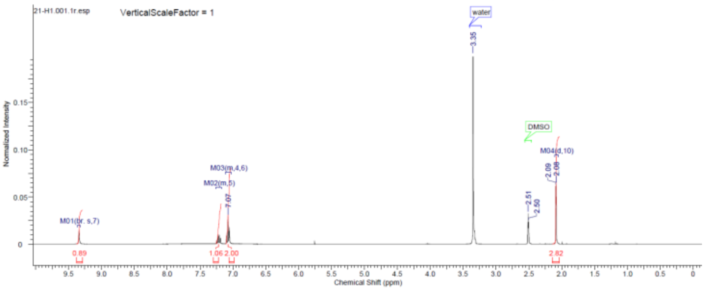

Acquisition Time (sec)	1.3631	Comment	OC_039_F_C13			Date	05 Aug 2014 00:32:16
Date Stamp	05 Aug 2014 00:32:16			File Name	\\hestia\work\chemistry\NMR Files\NMR_data\OCox\140804_OC_001-039F\20-C13\pdata\1\1r		
Frequency (MHz)	100.61	Nucleus	13C	Number of Transients	1024	Origin	Spect
Original Points Count	32768	Owner	sgcnmr	Points Count	32768	Pulse Sequence	jmod
Receiver Gain	194.71	SW(cyclical) (Hz)	24038.46	Solvent	DMSO-d6	Spectrum Offset (Hz)	10060.3320
Spectrum Type	APT	Sweep Width (Hz)	24037.73	Temperature (degree C)	25.158		

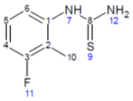

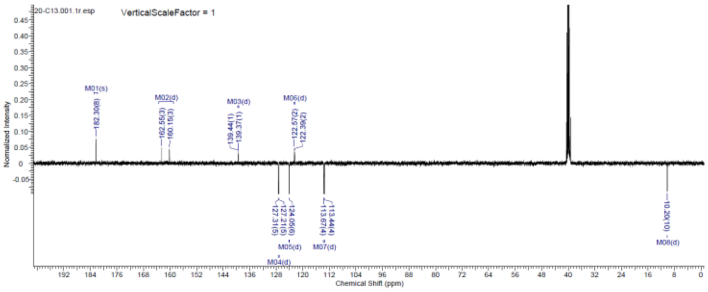

Acquisition Time (sec)	0.7330	Comment	OC_039F_F19	Date	22 Aug 2014 17:42:40		
Date Stamp	22 Aug 2014 17:42:40			File Name	\\hestia\work\chemistry\NMR Files\NMR_data\OCox\140822_OC_001-039F_F19\10\pdata\1\1r		
Frequency (MHz)	376.50	Nucleus	19F	Number of Transients	16	Origin	Spect
Original Points Count	65446	Owner	[illegible]	Points Count	131072	Pulse Sequence	zgflqn
Receiver Gain	194.71	SW(cyclical) (Hz)	89285.71	Solvent	DMSO-d6	Spectrum Offset (Hz)	-37649.6211
Spectrum Type	STANDARD	Sweep Width (Hz)	89285.03	Temperature (degree C)	25.158		

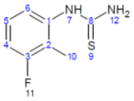

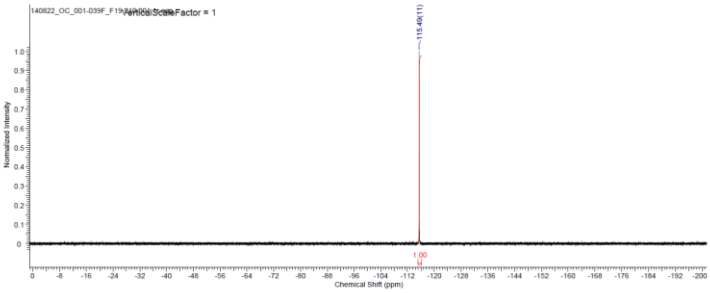

Compound **s13**

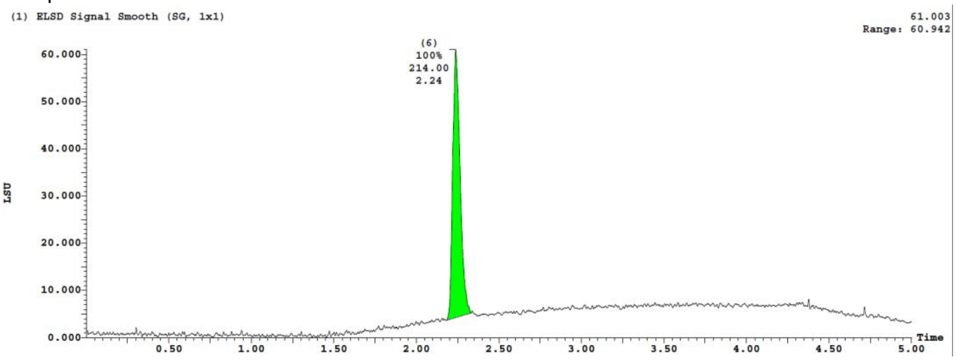

Acquisition Time (sec)	4.0894	Comment	OC_053_A_H1	Date	12 Dec 2014 16:36:16		
Date Stamp	12 Dec 2014 16:36:16			File Name	\\hestia\work\chemistry\NMR Files\NMR_data\OCox\141212_OC_001-053A\10-H1\pdata\1\1r		
Frequency (MHz)	400.13	Nucleus	1H	Number of Transients	16	Origin	Spect
Original Points Count	32768	Owner	sgcnmr	Points Count	65536	Pulse Sequence	zg30
Receiver Gain	194.71	SW(cyclical) (Hz)	8012.82	Solvent	DMSO-d6	Spectrum Offset (Hz)	2470.9668
Spectrum Type	STANDARD	Sweep Width (Hz)	8012.70	Temperature (degree C)	25.159		

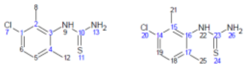

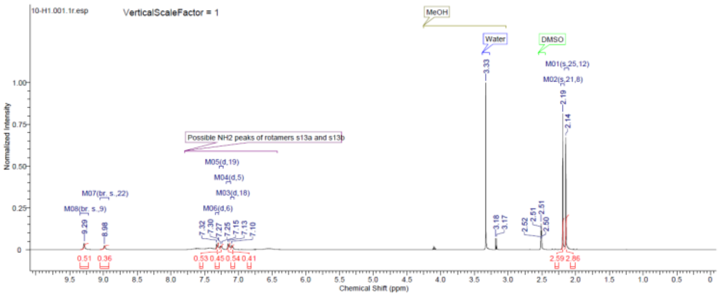

Acquisition Time (sec)	1.3631	Comment	OC_053_A_C13	Date	13 Dec 2014 06:09:04		
Date Stamp	13 Dec 2014 06:09:04			File Name	\\hestia\work\chemistry\NMR Files\NMR_data\OCox\141212_OC_001-053A\11-C13\pdata\1\1r		
Frequency (MHz)	100.61	Nucleus	13C	Number of Transients	1024	Origin	Spect
Original Points Count	32768	Owner	sgcnmr	Points Count	32768	Pulse Sequence	jmod
Receiver Gain	194.71	SW(cyclical) (Hz)	24038.46	Solvent	DMSO-d6	Spectrum Offset (Hz)	10060.3320
Spectrum Type	APT	Sweep Width (Hz)	24037.73	Temperature (degree C)	25.446		

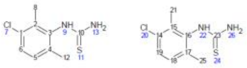

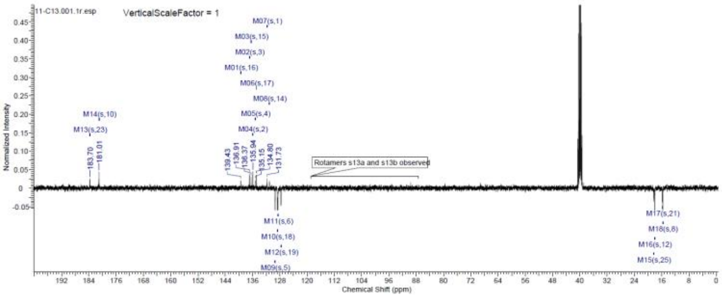

Acquisition Time (sec)	7.9299	Comment	OC_053_A_H1-70C	Date	16 Dec 2014 11:50:24		
Date Stamp	16 Dec 2014 11:50:24			File Name	\\hestia\work\chemistry\NMR Files\NMR_data\OCox\141212_OC_001-053A\15-H1-70C\pdata\1\1r		
Frequency (MHz)	400.13	Nucleus	1H	Number of Transients	8	Origin	Spect
Original Points Count	65536	Owner	sgcnmr	Points Count	131072	Pulse Sequence	zg60
Receiver Gain	141.83	SW(cyclical) (Hz)	8264.46	Solvent	DMSO-d6	Spectrum Offset (Hz)	2470.9700
Spectrum Type	STANDARD	Sweep Width (Hz)	8264.40	Temperature (degree C)	70.142		

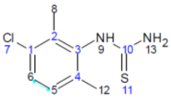

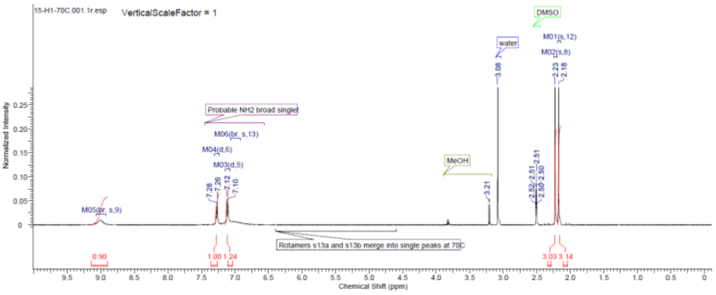

Compound **s14**

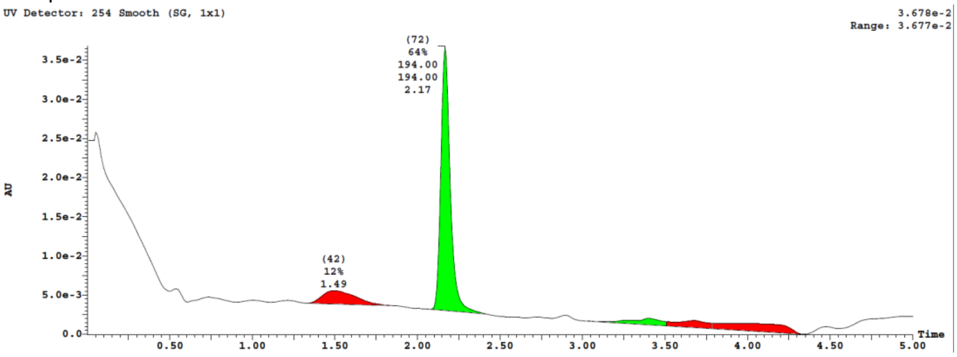

Acquisition Time (sec)	4.0894	***Comment***	OC 050 B H1	***Date***	27 Nov 2014 12:05:20		
Date Stamp	27 Nov 2014 12:05:20			***File Name***	\\hestia\work\chemistry\NMR Files\NMR data\OCox\141127 OC 001-050B\10-H1\pdata\1\1r		
Frequency (MHz)	400.13	***Nucleus***	1H	***Number of Transients***	16	***Origin***	Spect
Original Points Count	32768	***Owner***	sgcnmr	***Points Count***	65536	***Pulse Sequence***	zg30
Receiver Gain	194.71	***SW(cyclical) (Hz)***	8012.82	***Solvent***	CHLOROFORM-d		
Spectrum Offset (Hz)	2435.3630	***Spectrum Type***	STANDARD	***Sweep Width (Hz)***	8012.70	***Temperature (degree C)***	25.154

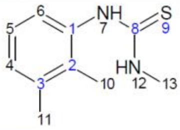

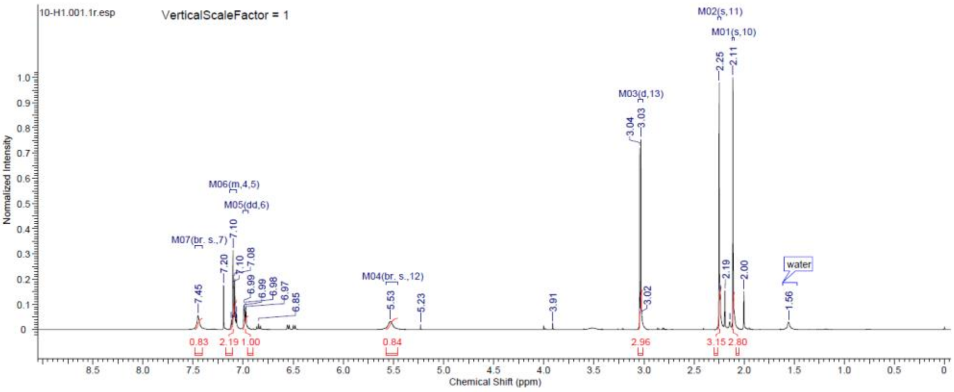

Acquisition Time (sec)	1.3631	***Comment***	OC 050 B C13			***Date***	28 Nov 2014 04:16:00
Date Stamp	28 Nov 2014 04:16:00			***File Name***	\\hestia\work\chemistry\NMR Files\NMR data\OCox\141127 OC 001-050B\11-C13\pdata\1\1r		
Frequency (MHz)	100.61	***Nucleus***	13C	***Number of Transients***	1024	***Origin***	Spect
Original Points Count	32768	***Owner***	sgcnmr	***Points Count***	32768	***Pulse Sequence***	jmod
Receiver Gain	194.71	***SW(cyclical) (Hz)***	24038.46	***Solvent***	CHLOROFORM-d		
Spectrum Offset (Hz)	10060.3320	***Spectrum Type***	APT	***Sweep Width (Hz)***	24037.73	***Temperature (degree C)***	25.243

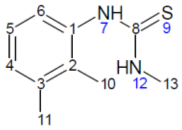

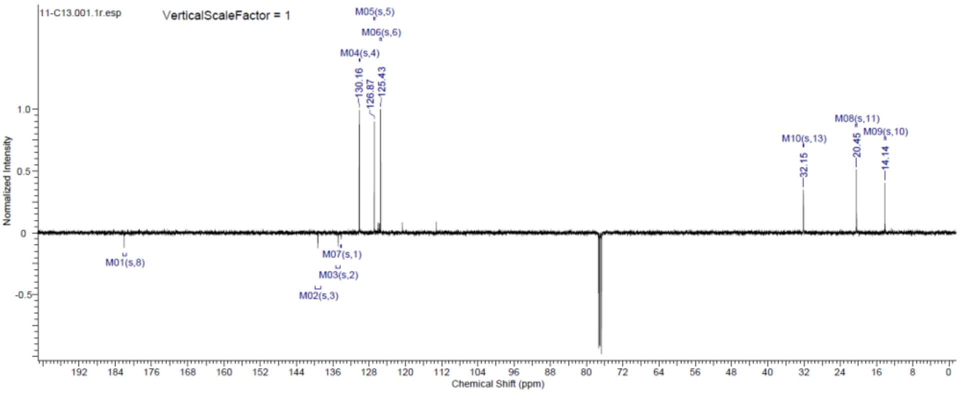

Compound **s15**

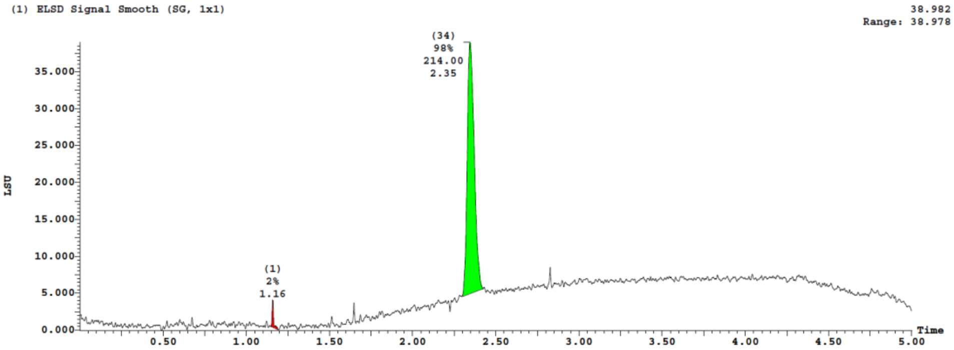

Acquisition Time (sec)	4.0894	Comment	OC_050_A_H1	Date	27 Nov 2014 11:58:56		
Date Stamp	27 Nov 2014 11:58:56			File Name	\\hestia\work\chemistry\NMR Files\NMR_data\OCox\141127_OC_001-050A\10-H1\pdata\1\1r		
Frequency (MHz)	400.13	Nucleus	1H	Number of Transients	16	Origin	Spect
Original Points Count	32768	Owner	sgcnmr	Points Count	65536	Pulse Sequence	zg30
Receiver Gain	194.71	SW(cyclical) (Hz)	8012.82	Solvent	CHLOROFORM-d		
Spectrum Offset (Hz)	2470.9668	Spectrum Type	STANDARD	Sweep Width (Hz)	8012.70	Temperature (degree C)	25.155

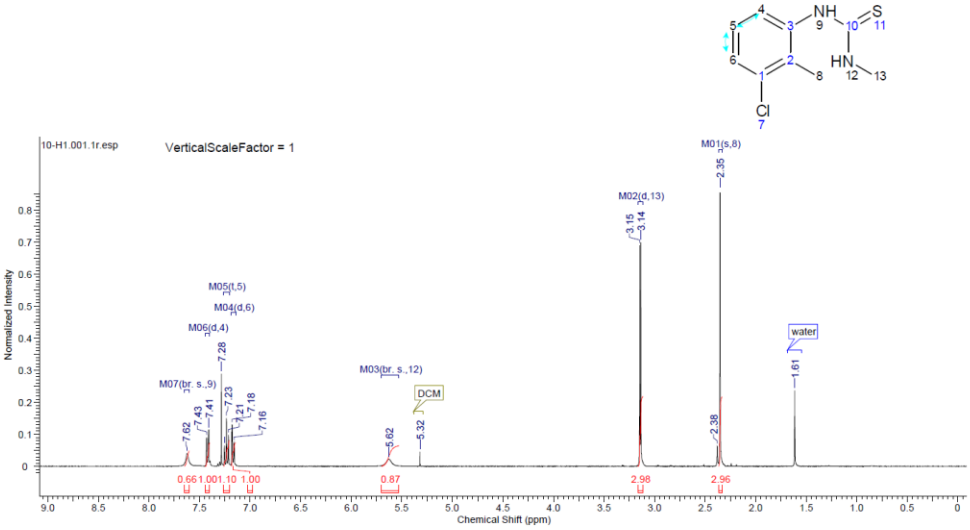

Acquisition Time (sec)	1.3631	Comment	OC_050_A_C13	Date	28 Nov 2014 02:12:16		
Date Stamp	28 Nov 2014 02:12:16			File Name	\\hestia\work\chemistry\NMR Files\NMR_data\OCox\141127_OC_001-050A\11-C13\pdata\1\1r		
Frequency (MHz)	100.61	Nucleus	13C	Number of Transients	1024	Origin	Spect
Original Points Count	32768	Owner	sgcnmr	Points Count	32768	Pulse Sequence	jmod
Receiver Gain	194.71	SW(cyclical) (Hz)	24038.46	Solvent	CHLOROFORM-d		
Spectrum Offset (Hz)	10060.3320	Spectrum Type	APT	Sweep Width (Hz)	24037.73	Temperature (degree C)	25.188

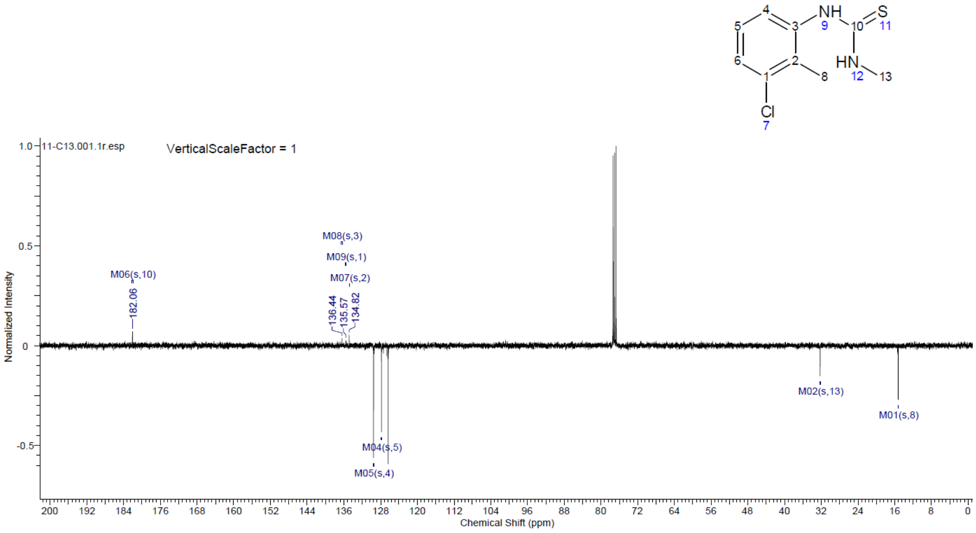

Compound **s16**

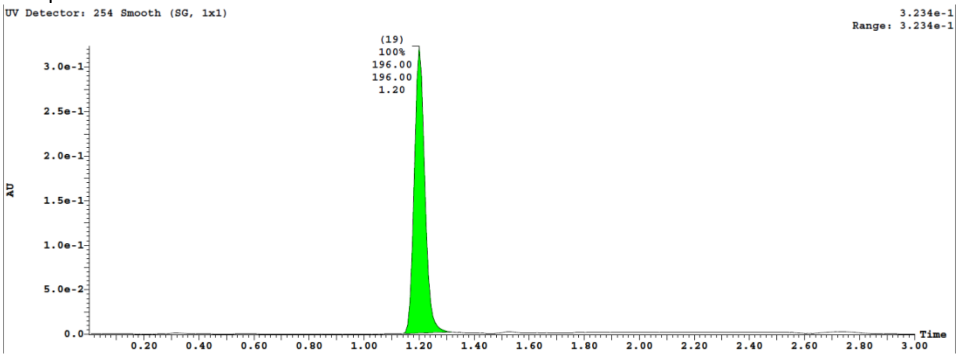

Acquisition Time (sec)	4.0894	Comment	OC 050 G	Date	05 Dec 2014 12:37:20		
Date Stamp	05 Dec 2014 12:37:20			File Name	\\hestia\work\chemistry\NMR Files\NMR data\OCox\141205 OC 001-050G\10-H1\pdata\1\1r		
Frequency (MHz)	400.13	Nucleus	1H	Number of Transients	16	Origin	Spect
Original Points Count	32768	Owner	sgcnmr	Points Count	65536	Pulse Sequence	zg30
Receiver Gain	194.71	SW(cyclical) (Hz)	8012.82	Solvent	CHLOROFORM-d		
Spectrum Offset (Hz)	2470.9668	Spectrum Type	STANDARD	Sweep Width (Hz)	8012.70	Temperature (degree C)	25.161

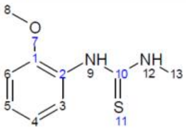

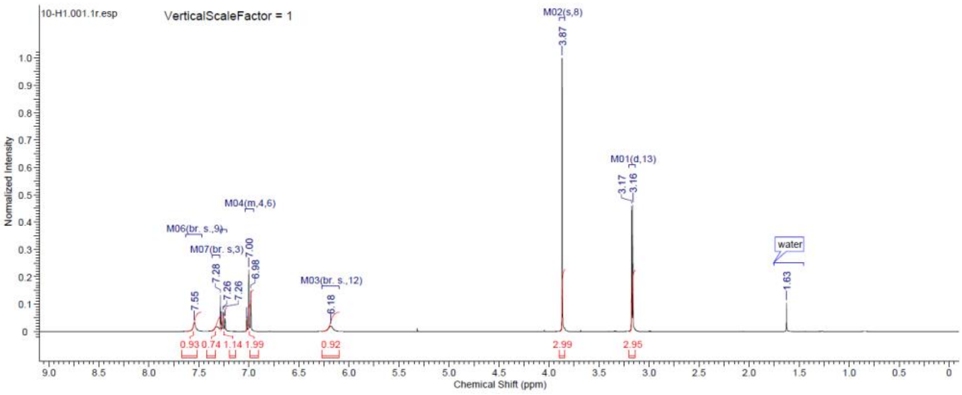

Acquisition Time (sec)	1.3631	Comment	OC 050 G 4hC13	Date	06 Dec 2014 17:44:32		
Date Stamp	06 Dec 2014 17:44:32			File Name	\\hestia\work\chemistry\NMR Files\NMR data\OCox\141205 OC 001-050G\15-C13\pdata\1\1r		
Frequency (MHz)	100.61	Nucleus	13C	Number of Transients	4096	Origin	Spect
Original Points Count	32768	Owner	sgcnmr	Points Count	32768	Pulse Sequence	zgpg30
Receiver Gain	194.71	SW(cyclical) (Hz)	24038.46	Solvent	CHLOROFORM-d		
Spectrum Offset (Hz)	10059.8594	Spectrum Type	STANDARD	Sweep Width (Hz)	24037.73	Temperature (degree C)	25.160

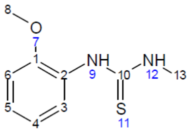

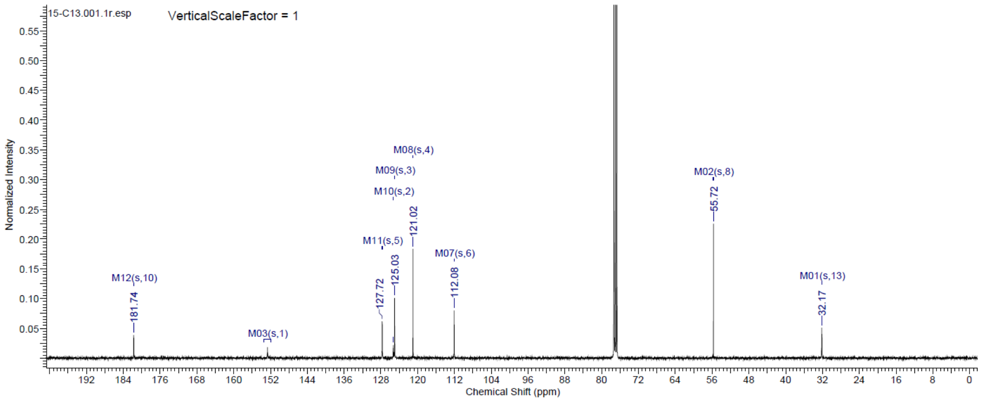

Compound **s18**

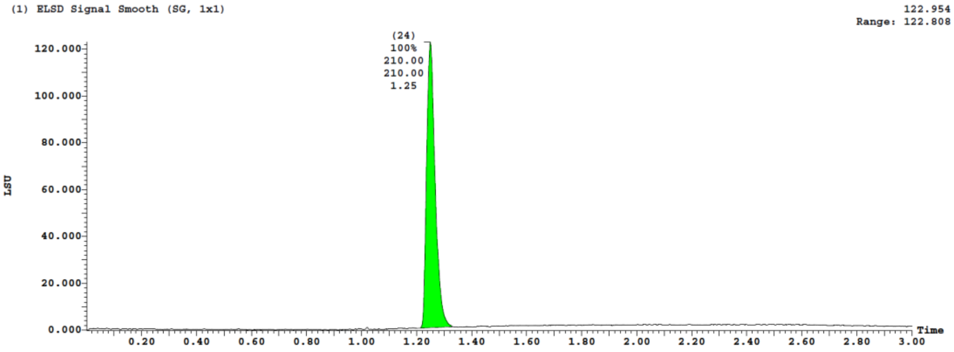

Acquisition Time (sec)	4.0894	***Comment***	OC_050_E	***Date***	02 Dec 2014 11:03:28		
Date Stamp	02 Dec 2014 11:03:28			***File Name***	\\hestia\work\chemistry\NMR Files\NMR_data\OCox\141202_OC_001-050E\10-H1\pdata\1\1r		
Frequency (MHz)	400.13	***Nucleus***	1H	***Number of Transients***	16	***Origin***	Spect
Original Points Count	32768	***Owner***	sgcnmr	***Points Count***	65536	***Pulse Sequence***	zg30
Receiver Gain	174.26	***SW(cyclical) (Hz)***	8012.82	***Solvent***	CHLOROFORM-d		
Spectrum Offset (Hz)	2436.8794	***Spectrum Type***	STANDARD	***Sweep Width (Hz)***	8012.70	***Temperature (degree C)***	25.155

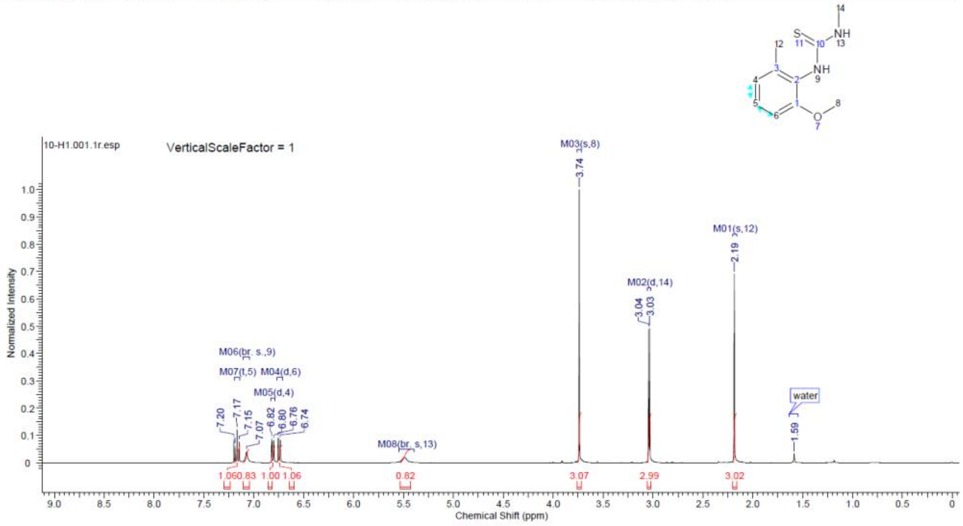

Acquisition Time (sec)	1.3631	***Comment***	OC_050_E_C13			***Date***	03 Dec 2014 04:35:12
Date Stamp	03 Dec 2014 04:35:12			***File Name***	\\hestia\work\chemistry\NMR Files\NMR_data\OCox\141202_OC_001-050E\20-C13\pdata\1\1r		
Frequency (MHz)	100.61	***Nucleus***	13C	***Number of Transients***	1024	***Origin***	Spect
Original Points Count	32768	***Owner***	sgcnmr	***Points Count***	32768	***Pulse Sequence***	jmod
Receiver Gain	194.71	***SW(cyclical) (Hz)***	24038.46	***Solvent***	CHLOROFORM-d		
Spectrum Offset (Hz)	10060.3320	***Spectrum Type***	APT	***Sweep Width (Hz)***	24037.73	***Temperature (degree C)***	25.161

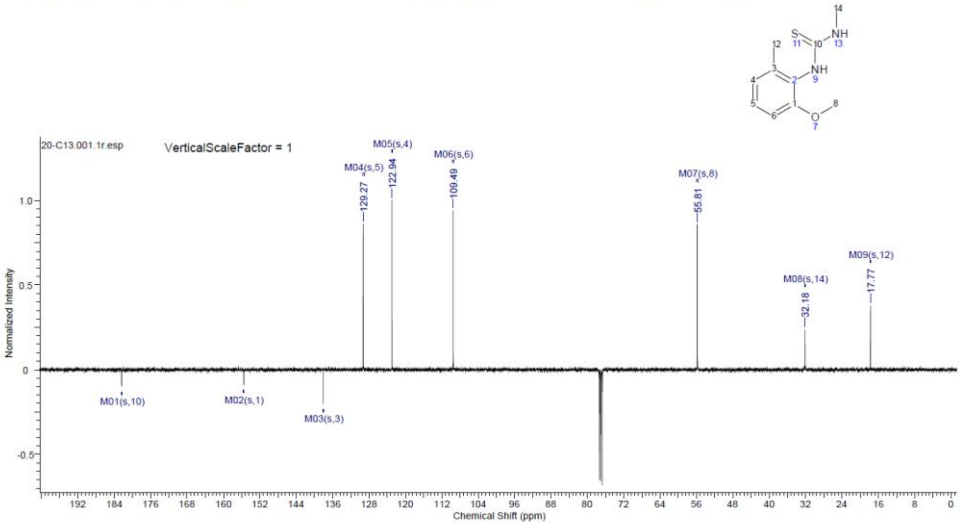

Compound **s19**

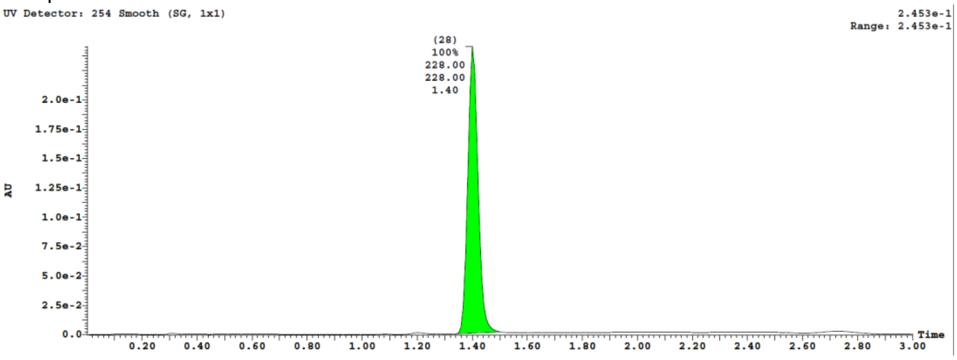

Acquisition Time (sec)	4.0894	Comment	OC 050 C	Date	02 Dec 2014 14:34:40		
Date Stamp	02 Dec 2014 14:34:40			File Name	\\hestia\work\chemistry\NMR Files\NMR data\OCox\141202 OC 001-050C\10-H1\pdata\1\1r		
Frequency (MHz)	400.13	Nucleus	1H	Number of Transients	16	Origin	Spect
Original Points Count	32768	Owner	sgcnmr	Points Count	65536	Pulse Sequence	zg30
Receiver Gain	194.71	SW(cyclical) (Hz)	8012.82	Solvent	CHLOROFORM-d		
Spectrum Offset (Hz)	2470.9668	Spectrum Type	STANDARD	Sweep Width (Hz)	8012.70	Temperature (degree C)	25.157

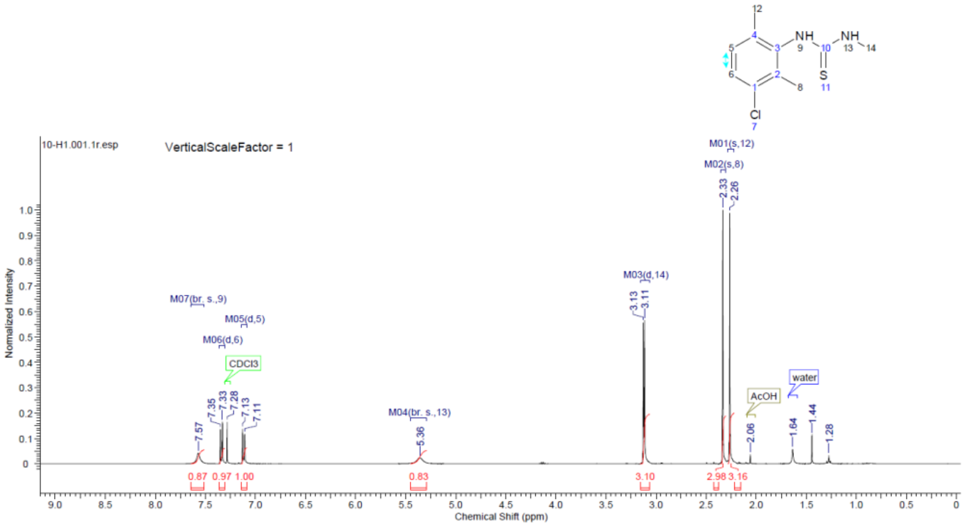

Acquisition Time (sec)	1.3631	Comment	OC 050 C C13	Date	04 Dec 2014 05:54:08		
Date Stamp	04 Dec 2014 05:54:08			File Name	\\hestia\work\chemistry\NMR Files\NMR data\OCox\141202 OC 001-050C\11-C13\pdata\1\1r		
Frequency (MHz)	100.61	Nucleus	13C	Number of Transients	1024	Origin	Spect
Original Points Count	32768	Owner	sgcnmr	Points Count	32768	Pulse Sequence	jmod
Receiver Gain	194.71	SW(cyclical) (Hz)	24038.46	Solvent	CHLOROFORM-d		
Spectrum Offset (Hz)	10060.3320	Spectrum Type	APT	Sweep Width (Hz)	24037.73	Temperature (degree C)	25.161

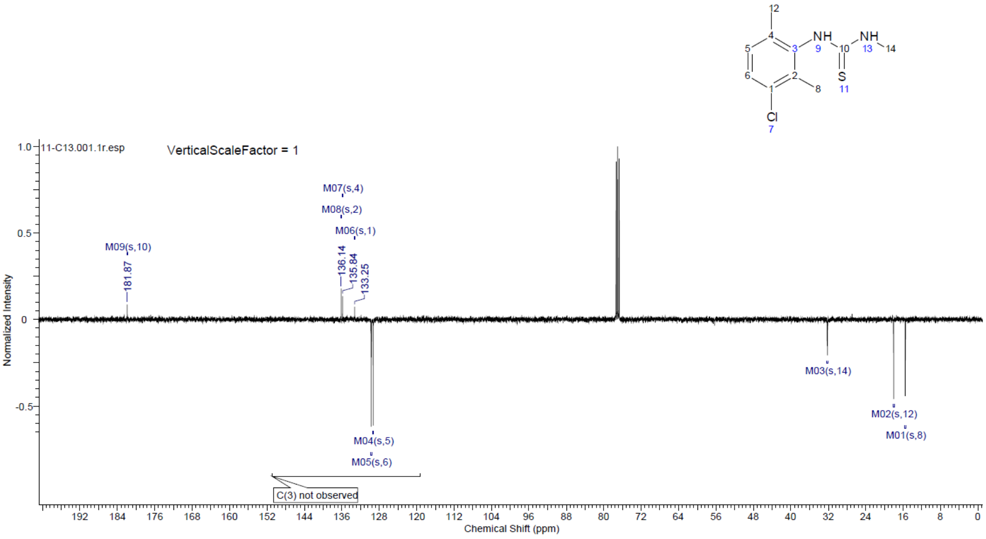

Compound **s22**

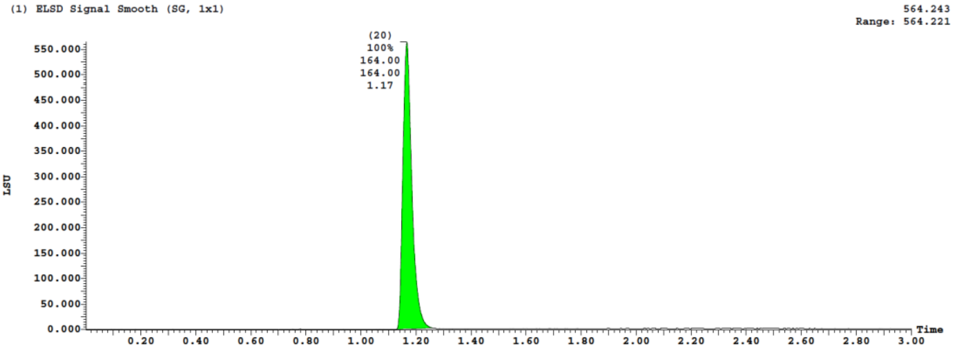

Acquisition Time (sec)	4.0894	Comment	OC_051_B_H1	Date	26 Nov 2014 17:44:32		
Date Stamp	26 Nov 2014 17:44:32			File Name	\\hestia\work\chemistry\NMR Files\NMR_data\OCox\141126_OC_001-051B\10-H1\pdata\1\1r		
Frequency (MHz)	400.13	Nucleus	1H	Number of Transients	16	Origin	Spect
Original Points Count	32768	Owner	sgcnmr	Points Count	65536	Pulse Sequence	zg30
Receiver Gain	155.34	SW(cyclical) (Hz)	8012.82	Solvent	DMSO-d6	Spectrum Offset (Hz)	2470.9668
Spectrum Type	STANDARD	Sweep Width (Hz)	8012.70	Temperature (degree C)	25.159		

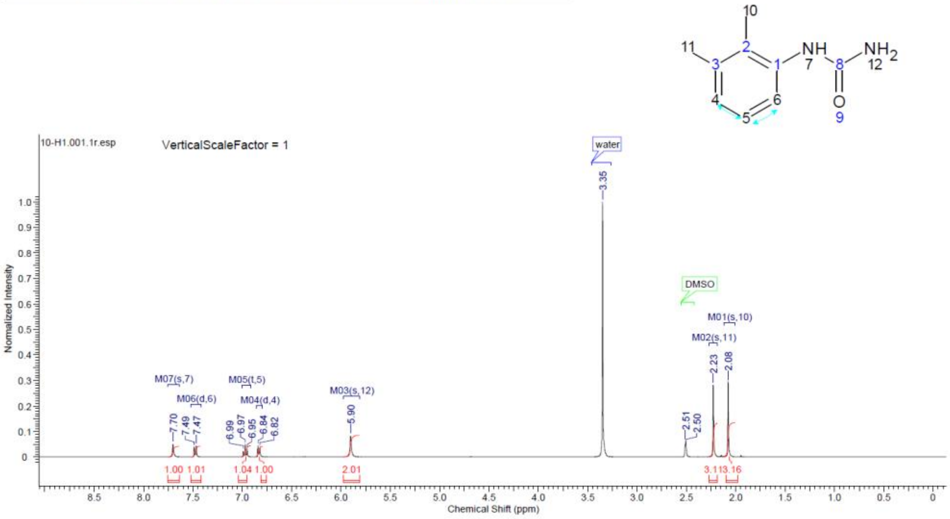

Acquisition Time (sec)	1.3631	Comment	OC_051_B_C13			Date	26 Nov 2014 21:02:56
Date Stamp	26 Nov 2014 21:02:56			File Name	\\hestia\work\chemistry\NMR Files\NMR_data\OCox\141126_OC_001-051B\11-C13\pdata\1\1r		
Frequency (MHz)	100.61	Nucleus	13C	Number of Transients	1024	Origin	Spect
Original Points Count	32768	Owner	sgcnmr	Points Count	32768	Pulse Sequence	jmod
Receiver Gain	194.71	SW(cyclical) (Hz)	24038.46	Solvent	DMSO-d6	Spectrum Offset (Hz)	10060.3320
Spectrum Type	APT	Sweep Width (Hz)	24037.73	Temperature (degree C)	25.161		

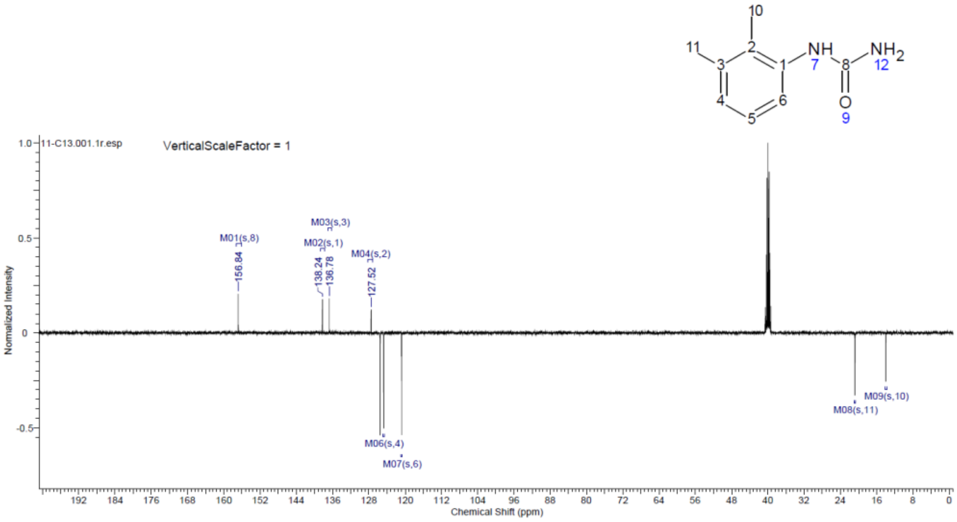

Compound **s23**

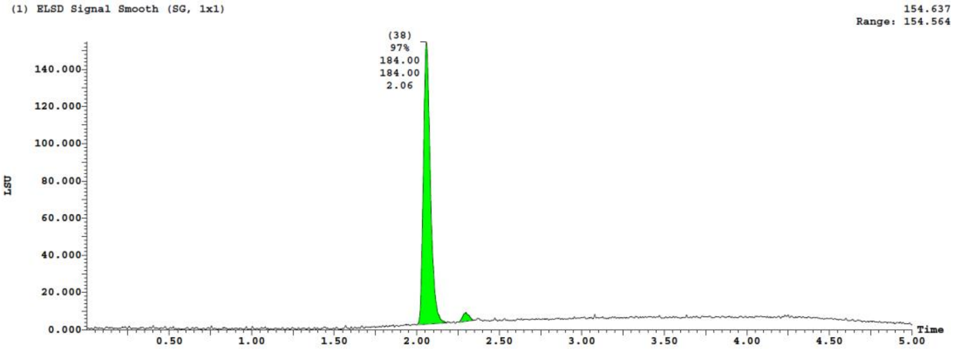

Acquisition Time (sec)	4.0894	Comment	OC_051_A	Date	02 Dec 2014 13:41:20		
Date Stamp	02 Dec 2014 13:41:20			File Name	\\hestia\work\chemistry\NMR Files\NMR_data\OCox\141202_OC_001-051A\10-H1\pdata\1\1r		
Frequency (MHz)	400.13	Nucleus	1H	Number of Transients	16	Origin	Spect
Original Points Count	32768	Owner	sgcnmr	Points Count	65536	Pulse Sequence	zg30
Receiver Gain	194.71	SW(cyclical) (Hz)	8012.82	Solvent	DMSO-d6	Spectrum Offset (Hz)	2470.9668
Spectrum Type	STANDARD	Sweep Width (Hz)	8012.70	Temperature (degree C)	25.158		

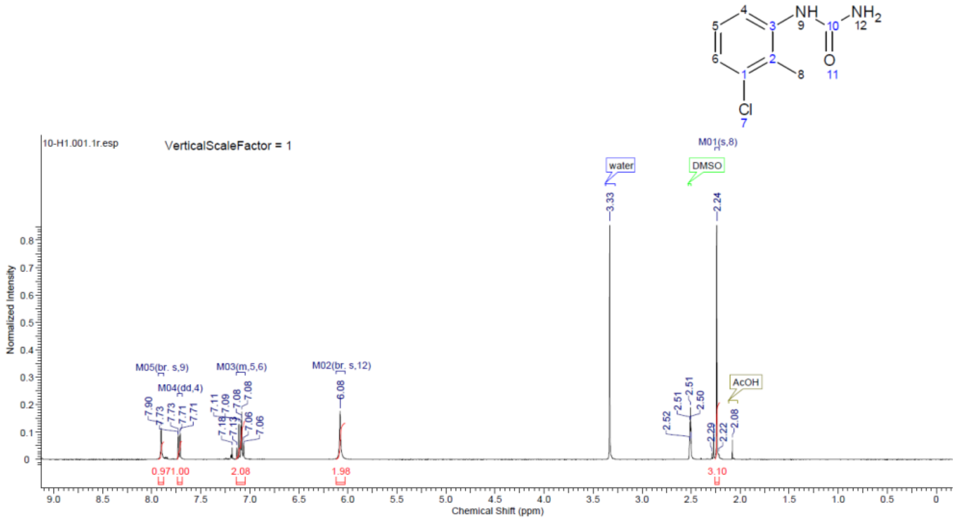

Acquisition Time (sec)	1.3631	Comment	OC_051_A_C13			Date	03 Dec 2014 23:49:20
Date Stamp	03 Dec 2014 23:49:20			File Name	\\hestia\work\chemistry\NMR Files\NMR_data\OCox\141202_OC_001-051A\11-C13\pdata\1\1r		
Frequency (MHz)	100.61	Nucleus	13C	Number of Transients	1024	Origin	Spect
Original Points Count	32768	Owner	sgcnmr	Points Count	32768	Pulse Sequence	jmod
Receiver Gain	194.71	SW(cyclical) (Hz)	24038.46	Solvent	DMSO-d6	Spectrum Offset (Hz)	10060.3320
Spectrum Type	APT	Sweep Width (Hz)	24037.73	Temperature (degree C)	25.159		

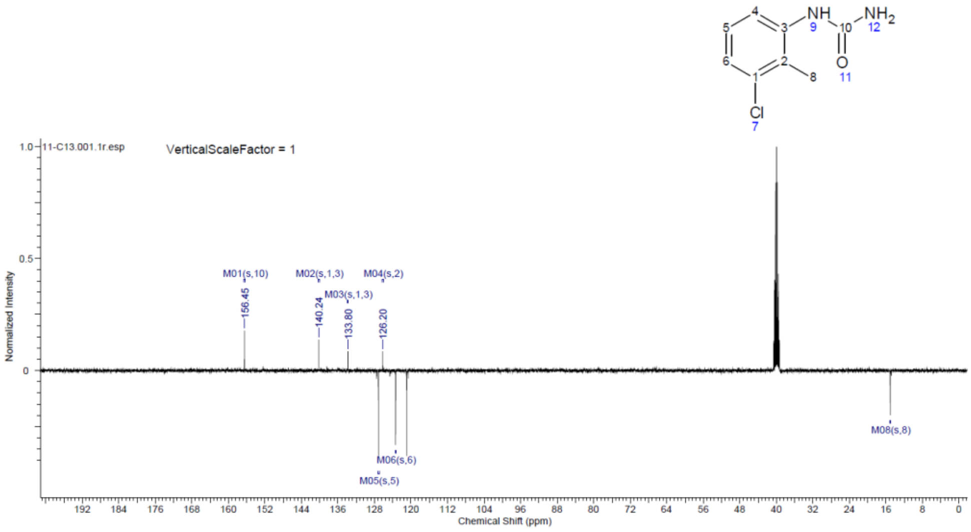

Compound **s24**

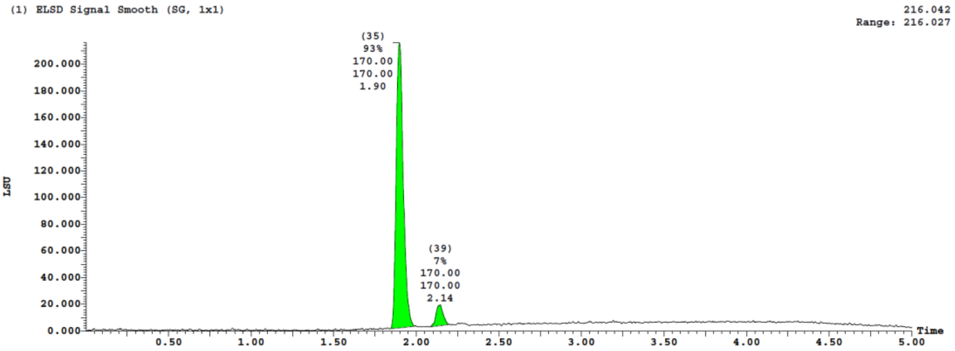

Acquisition Time (sec)	4.0894	Comment	OC_051_F	Date	02 Dec 2014 13:52:00		
Date Stamp	02 Dec 2014 13:52:00			File Name	\\hestia\work\chemistry\NMR Files\NMR_data\OCox\141202_OC_001-051F\10-H1\pdata\1\1r		
Frequency (MHz)	400.13	Nucleus	1H	Number of Transients	16	Origin	Spect
Original Points Count	32768	Owner	sgcnmr	Points Count	65536	Pulse Sequence	zg30
Receiver Gain	194.71	SW(cyclical) (Hz)	8012.82	Solvent	DMSO-d6	Spectrum Offset (Hz)	2470.9668
Spectrum Type	STANDARD	Sweep Width (Hz)	8012.70	Temperature (degree C)	25.158		

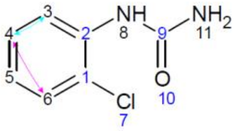

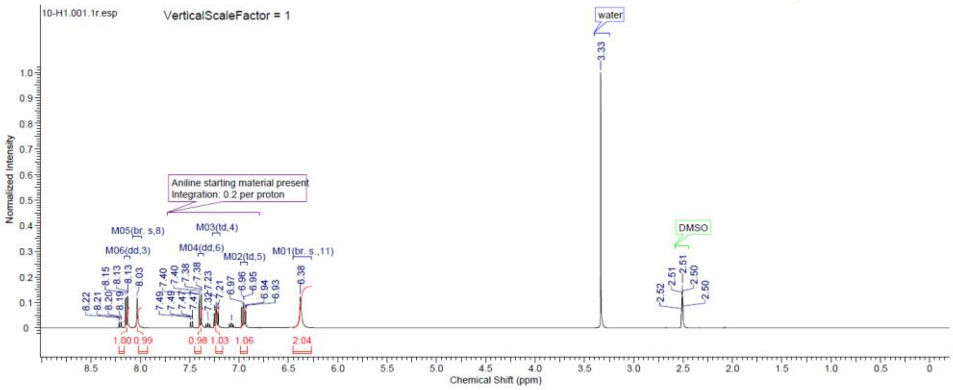

Acquisition Time (sec)	1.3631	Comment	OC_051_F_C13			Date	04 Dec 2014 03:50:24
Date Stamp	04 Dec 2014 03:50:24			File Name	\\hestia\work\chemistry\NMR Files\NMR_data\OCox\141202_OC_001-051F\11-C13\pdata\1\1r		
Frequency (MHz)	100.61	Nucleus	13C	Number of Transients	1024	Origin	Spect
Original Points Count	32768	Owner	sgcnmr	Points Count	32768	Pulse Sequence	jmod
Receiver Gain	194.71	SW(cyclical) (Hz)	24038.46	Solvent	DMSO-d6	Spectrum Offset (Hz)	10060.3320
Spectrum Type	APT	Sweep Width (Hz)	24037.73	Temperature (degree C)	25.162		

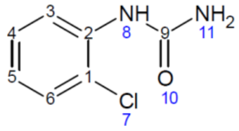

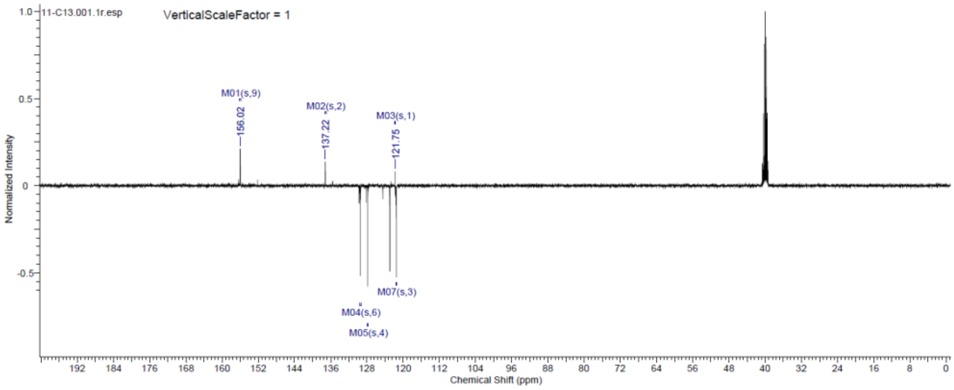

Compound **s25**

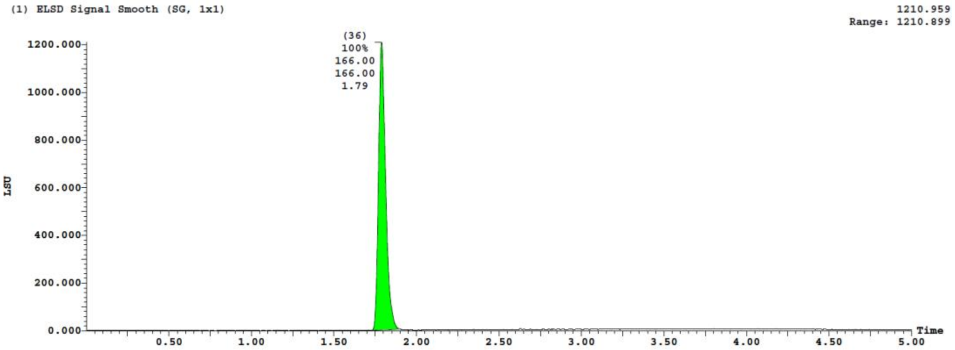

Acquisition Time (sec)	4.0894	Comment	OC 051 G	Date	01 Dec 2014 14:58:08		
Date Stamp	01 Dec 2014 14:58:08			File Name	\\hestia\work\chemistry\NMR Files\NMR data\OCox\141201 OC 001-051G\10-H1\pdata\1\1r		
Frequency (MHz)	400.13	Nucleus	1H	Number of Transients	16	Origin	Spect
Original Points Count	32768	Owner	sgcnmr	Points Count	65536	Pulse Sequence	zg30
Receiver Gain	77.20	SW(cyclical) (Hz)	8012.82	Solvent	DMSO-d6	Spectrum Offset (Hz)	2470.9668
Spectrum Type	STANDARD	Sweep Width (Hz)	8012.70	Temperature (degree C)	25.158		

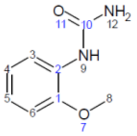

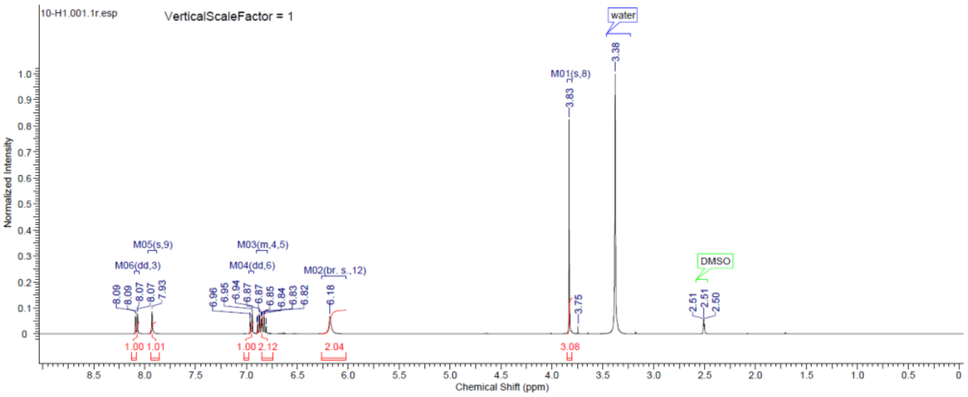

Acquisition Time (sec)	1.3631	Comment	OC 051 G C13			Date	03 Dec 2014 02:31:28
Date Stamp	03 Dec 2014 02:31:28			File Name	\\hestia\work\chemistry\NMR Files\NMR data\OCox\141201 OC 001-051G\11-C13\pdata\1\1r		
Frequency (MHz)	100.61	Nucleus	13C	Number of Transients	1024	Origin	Spect
Original Points Count	32768	Owner	sgcnmr	Points Count	32768	Pulse Sequence	jmod
Receiver Gain	194.71	SW(cyclical) (Hz)	24038.46	Solvent	DMSO-d6	Spectrum Offset (Hz)	10060.3320
Spectrum Type	APT	Sweep Width (Hz)	24037.73	Temperature (degree C)	25.161		

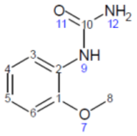

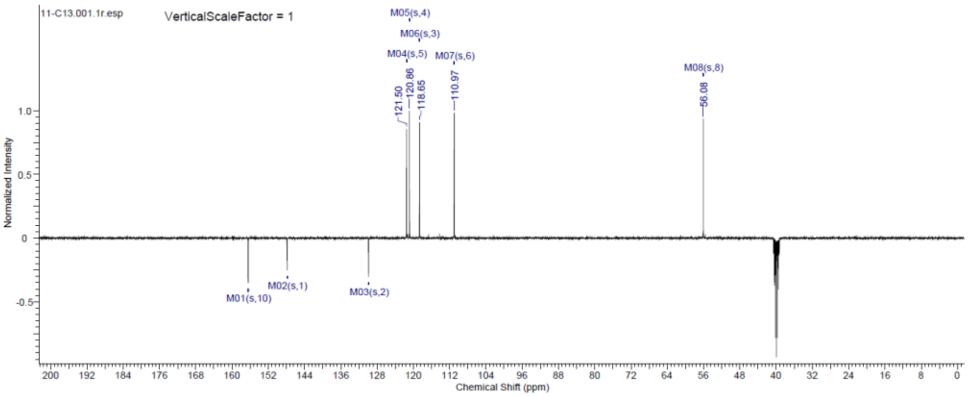

Compound **s27**

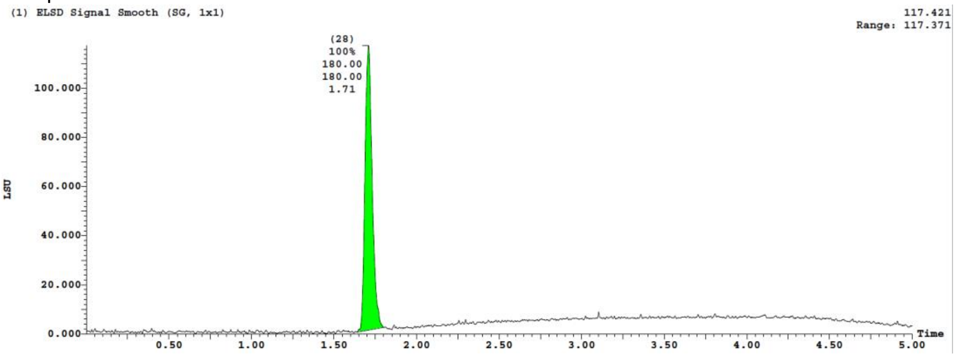

Acquisition Time (sec)	4.0894	Comment	OC_051_E	Date	02 Dec 2014 13:45:36		
Date Stamp	02 Dec 2014 13:45:36			File Name	\\hestia\work\chemistry\NMR Files\NMR_data\OCox\141202_OC_001-051E\10-H1\pdata\1\1r		
Frequency (MHz)	400.13	Nucleus	1H	Number of Transients	16	Origin	Spect
Original Points Count	32768	Owner	sgcnmr	Points Count	65536	Pulse Sequence	zg30
Receiver Gain	194.71	SW(cyclical) (Hz)	8012.82	Solvent	DMSO-d6	Spectrum Offset (Hz)	2470.9668
Spectrum Type	STANDARD	Sweep Width (Hz)	8012.70	Temperature (degree C)	25.159		

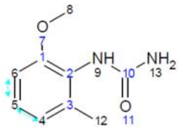

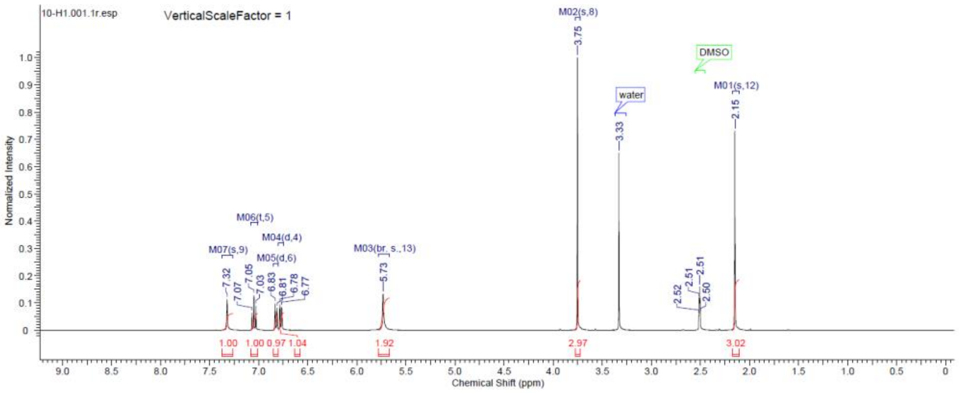

Acquisition Time (sec)	1.3631	Comment	OC_051_E_C13			Date	04 Dec 2014 01:46:40
Date Stamp	04 Dec 2014 01:46:40			File Name	\\hestia\work\chemistry\NMR Files\NMR_data\OCox\141202_OC_001-051E\11-C13\pdata\1\1r		
Frequency (MHz)	100.61	Nucleus	13C	Number of Transients	1024	Origin	Spect
Original Points Count	32768	Owner	sgcnmr	Points Count	32768	Pulse Sequence	jmod
Receiver Gain	194.71	SW(cyclical) (Hz)	24038.46	Solvent	DMSO-d6	Spectrum Offset (Hz)	10060.3320
Spectrum Type	APT	Sweep Width (Hz)	24037.73	Temperature (degree C)	25.159		

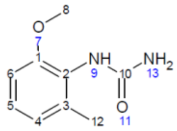

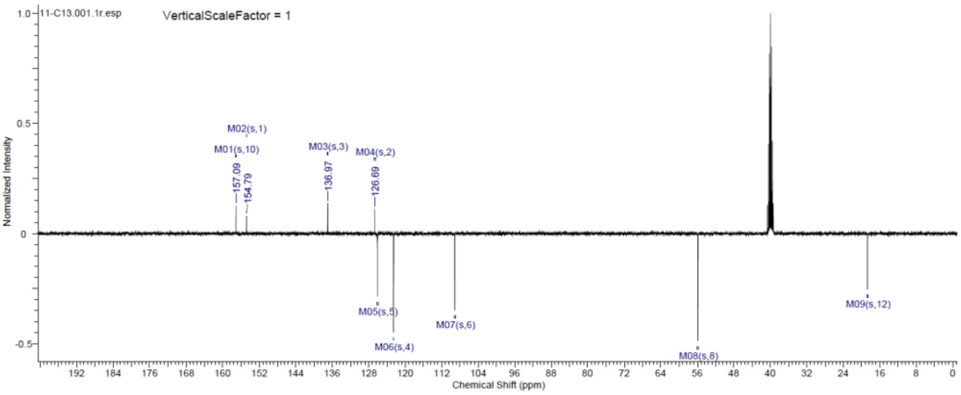

Compound **s29**

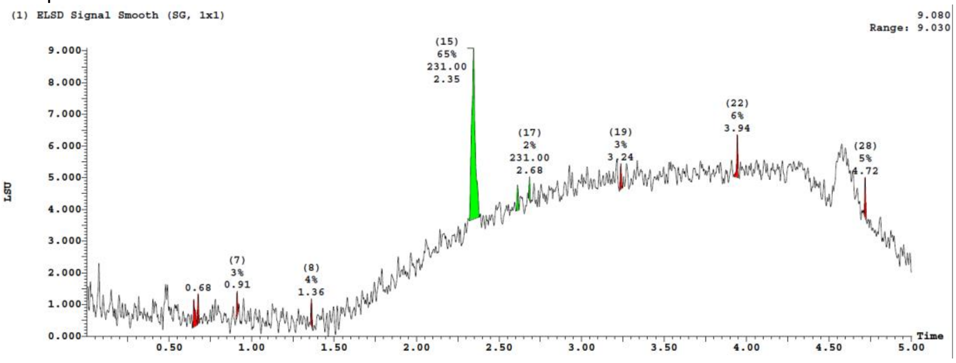

Acquisition Time (sec)	4.0894	Comment	OC_038_A_H1	Date	31 Jul 2014 07:30:24		
Date Stamp	31 Jul 2014 07:30:24			File Name	\\hestia\work\chemistry\NMR Files\NMR_data\OCox\140730_OC_001-038A\11-H1\pdata\1\1r		
Frequency (MHz)	400.13	Nucleus	1H	Number of Transients	16	Origin	Spect
Original Points Count	32768	Owner	sgcnmr	Points Count	65536	Pulse Sequence	zg30
Receiver Gain	194.71	SW(cyclical) (Hz)	8012.82	Solvent	CHLOROFORM-d		
Spectrum Offset (Hz)	2434.6272	Spectrum Type	STANDARD	Sweep Width (Hz)	8012.70	Temperature (degree C)	25.164

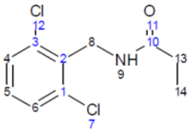

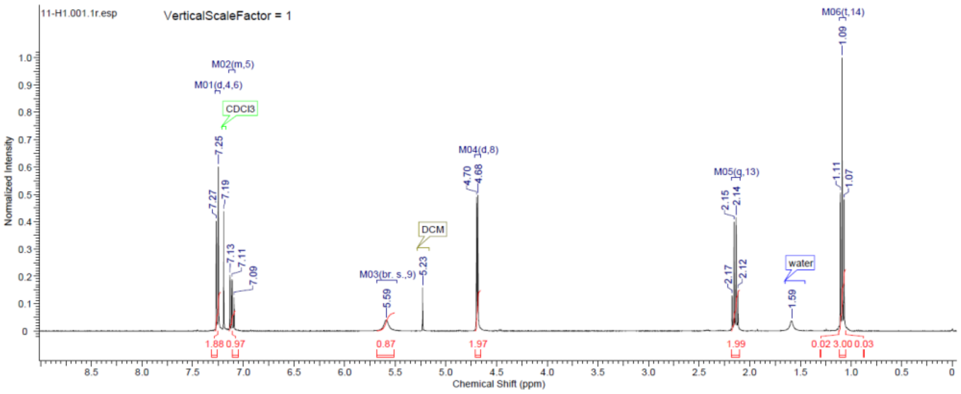

Acquisition Time (sec)	1.3631	Comment	OC_038_A_C13			Date	01 Aug 2014 03:46:24
Date Stamp	01 Aug 2014 03:46:24			File Name	\\hestia\work\chemistry\NMR Files\NMR_data\OCox\140730_OC_001-038A\10-C13\pdata\1\1r		
Frequency (MHz)	100.61	Nucleus	13C	Number of Transients	1024	Origin	Spect
Original Points Count	32768	Owner	sgcnmr	Points Count	32768	Pulse Sequence	jmod
Receiver Gain	194.71	SW(cyclical) (Hz)	24038.46	Solvent	CHLOROFORM-d		
Spectrum Offset (Hz)	10060.3320	Spectrum Type	APT	Sweep Width (Hz)	24037.73	Temperature (degree C)	25.160

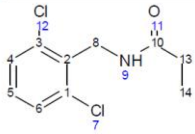

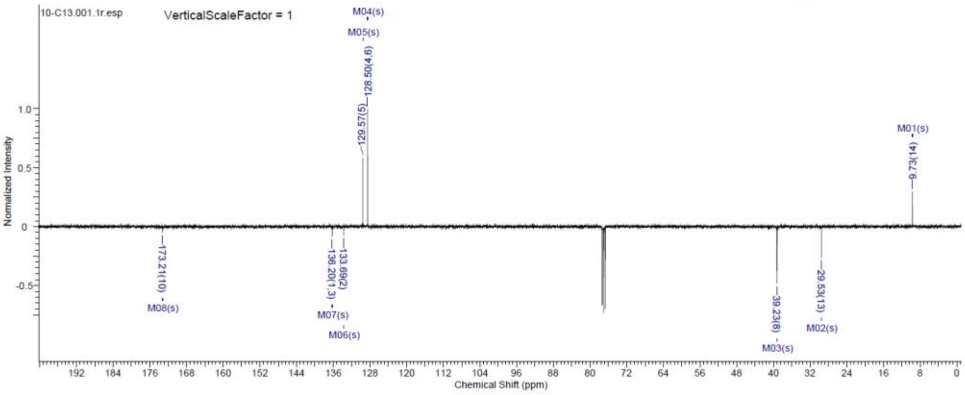

Compound **s30**

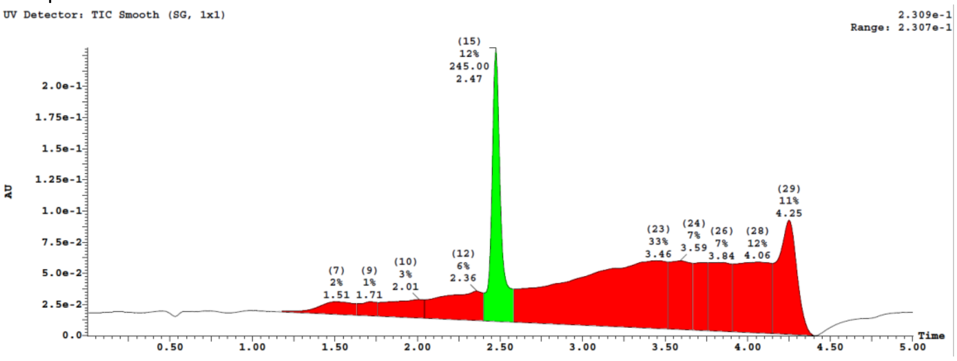

**Acquisition Time (sec)**	4.0894	**Comment**	OC_038_B_H1	**Date**	01 Aug 2014 05:45:52		
**Date Stamp**	01 Aug 2014 05:45:52			**File Name**	\\hestia\work\chemistry\NMR Files\NMR_data\OCox\140730_OC_001-038B\11-H1\pdata\1\1r		
**Frequency (MHz)**	400.13	**Nucleus**	1H	**Number of Transients**	16	**Origin**	Spect
**Original Points Count**	32768	**Owner**	sgcnmr	**Points Count**	65536	**Pulse Sequence**	zg30
**Receiver Gain**	194.71	**SW(cyclical) (Hz)**	8012.82	**Solvent**	CHLOROFORM-d		
**Spectrum Offset (Hz)**	2435.4675	**Spectrum Type**	STANDARD	**Sweep Width (Hz)**	8012.70	**Temperature (degree C)**	25.152

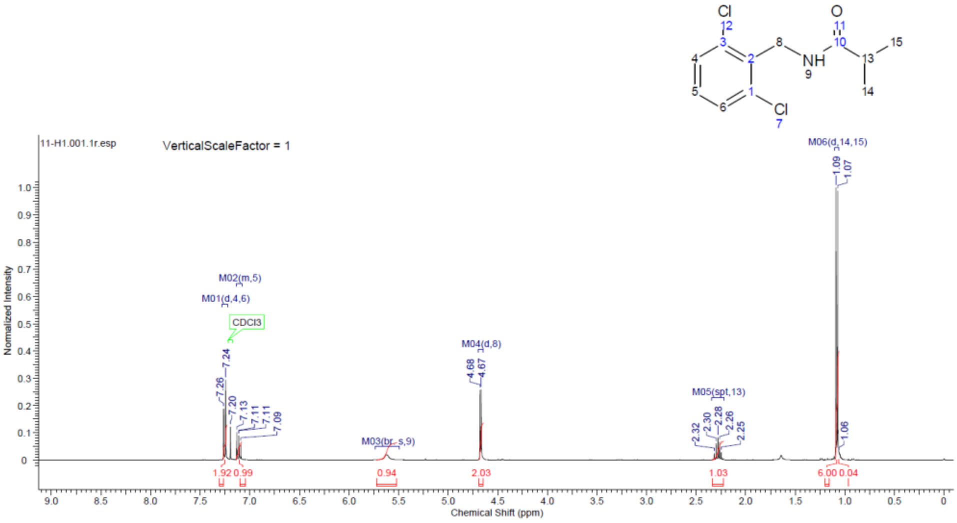

**Acquisition Time (sec)**	1.3631	**Comment**	OC_038_B_C13			**Date**	01 Aug 2014 05:43:44
**Date Stamp**	01 Aug 2014 05:43:44			**File Name**	\\hestia\work\chemistry\NMR Files\NMR_data\OCox\140730_OC_001-038B\10-C13\pdata\1\1r		
**Frequency (MHz)**	100.61	**Nucleus**	13C	**Number of Transients**	1024	**Origin**	Spect
**Original Points Count**	32768	**Owner**	sgcnmr	**Points Count**	32768	**Pulse Sequence**	jmod
**Receiver Gain**	194.71	**SW(cyclical) (Hz)**	24038.46	**Solvent**	CHLOROFORM-d		
**Spectrum Offset (Hz)**	10060.3320	**Spectrum Type**	APT	**Sweep Width (Hz)**	24037.73	**Temperature (degree C)**	25.159

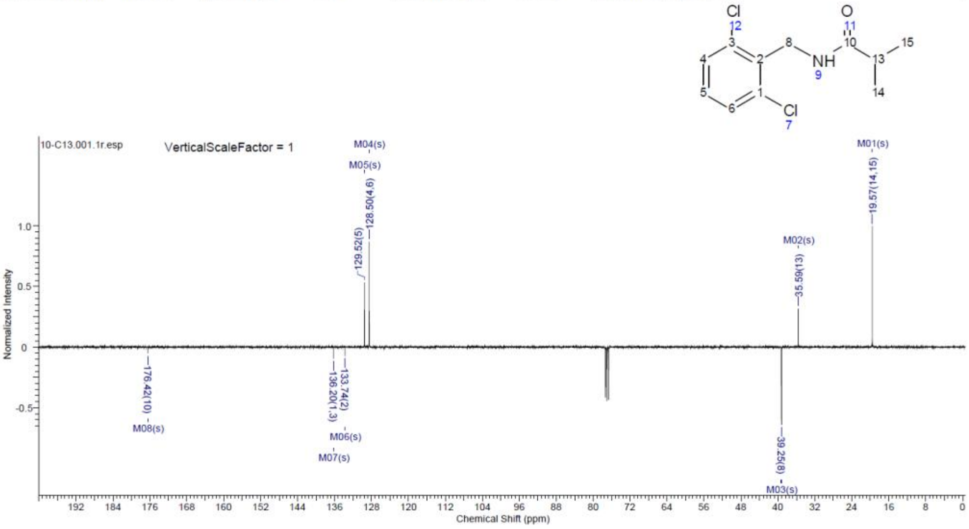

Compound **s31**

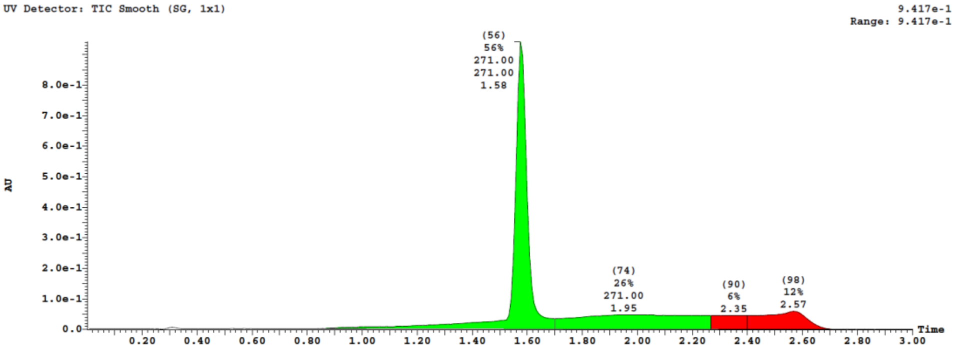

Acquisition Time (sec)	4.0894	Comment	OC_034_H1	Date	11 Jul 2014 02:36:00		
Date Stamp	11 Jul 2014 02:36:00			File Name	\\hestia\work\chemistry\NMR Files\NMR_data\OCox\140710_OC_001-034\12 H1\pdata\1\1r		
Frequency (MHz)	400.13	Nucleus	1H	Number of Transients	16	Origin	Spect
Original Points Count	32768	Owner	sgcnmr	Points Count	65536	Pulse Sequence	zg30
Receiver Gain	194.71	SW(cyclical) (Hz)	8012.82	Solvent	CHLOROFORM-d		
Spectrum Offset (Hz)	2465.1809	Spectrum Type	STANDARD	Sweep Width (Hz)	8012.70	Temperature (degree C)	25.160

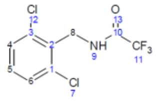

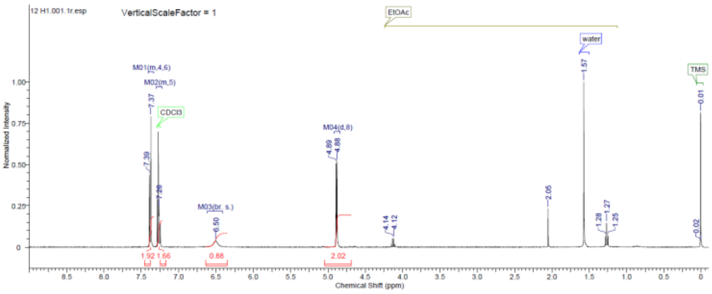

Acquisition Time (sec)	1.3631	Comment	OC_034_C13	Date	11 Jul 2014 02:12:32		
Date Stamp	11 Jul 2014 02:12:32			File Name	\\hestia\work\chemistry\NMR Files\NMR_data\OCox\140710_OC_001-034\10 C13\pdata\1\1r		
Frequency (MHz)	100.61	Nucleus	13C	Number of Transients	1024	Origin	Spect
Original Points Count	32768	Owner	sgcnmr	Points Count	32768	Pulse Sequence	zgpg30
Receiver Gain	194.71	SW(cyclical) (Hz)	24038.46	Solvent	CHLOROFORM-d		
Spectrum Offset (Hz)	10059.8594	Spectrum Type	STANDARD	Sweep Width (Hz)	24037.73	Temperature (degree C)	25.161

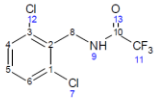

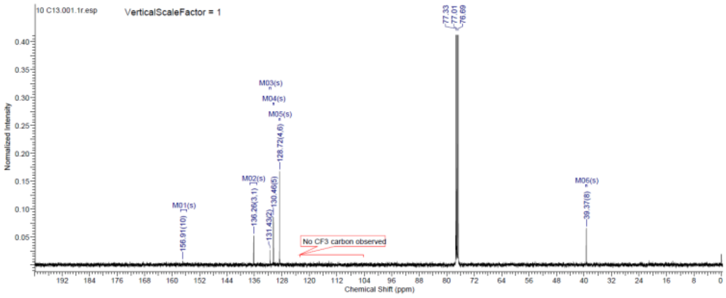

Acquisition Time (sec)	0.7330	Comment	OC_034_F19	Date	22 Aug 2014 17:32:00		
Date Stamp	22 Aug 2014 17:32:00			File Name	\\hestia\work\chemistry\NMR Files\NMR_data\OCox\140822_OC_001-034_F19\10\pdata\1\1r		
Frequency (MHz)	376.50	Nucleus	19F	Number of Transients	16	Origin	Spect
Original Points Count	65446	Owner	sgcnmr	Points Count	131072	Pulse Sequence	zgflqn
Receiver Gain	194.71	SW(cyclical) (Hz)	89285.71	Solvent	CHLOROFORM-d		
Spectrum Offset (Hz)	-37649.6211	Spectrum Type	STANDARD	Sweep Width (Hz)	89285.03	Temperature (degree C)	25.160

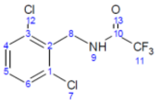

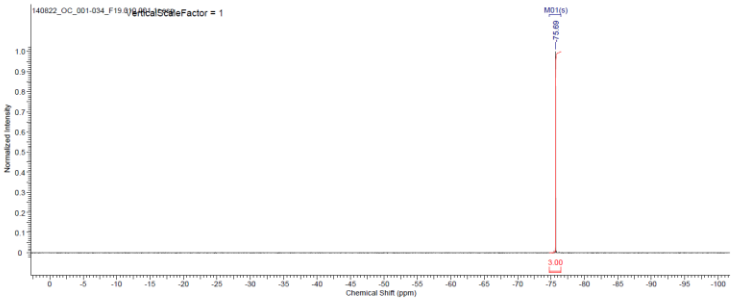

Compound **s32**

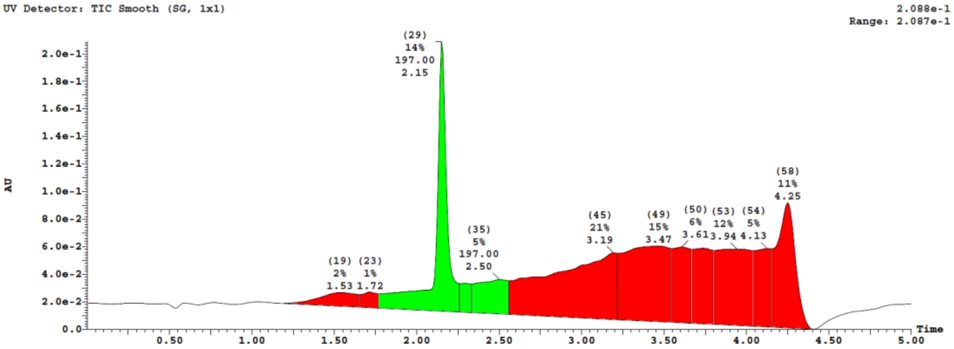

Acquisition Time (sec)	4.0894	Comment	OC_037_E_H1	Date	31 Jul 2014 05:07:28		
Date Stamp	31 Jul 2014 05:07:28			File Name	\\hestia\work\chemistry\NMR Files\NMR_data\OCox\140730_OC_001-037E\11-H1\pdata\1\1r		
Frequency (MHz)	400.13	Nucleus	1H	Number of Transients	16	Origin	Spect
Original Points Count	32768	Owner	sgcnmr	Points Count	65536	Pulse Sequence	zg30
Receiver Gain	194.71	SW(cyclical) (Hz)	8012.82	Solvent	CHLOROFORM-d		
Spectrum Offset (Hz)	2434.3870	Spectrum Type	STANDARD	Sweep Width (Hz)	8012.70	Temperature (degree C)	25.152

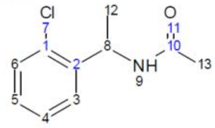

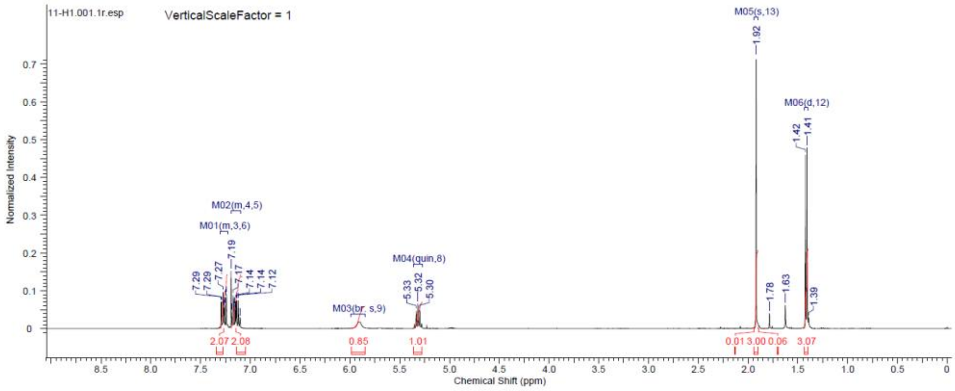

Acquisition Time (sec)	1.3631	Comment	OC_037_E_C13			Date	31 Jul 2014 05:05:20
Date Stamp	31 Jul 2014 05:05:20			File Name	\\hestia\work\chemistry\NMR Files\NMR_data\OCox\140730_OC_001-037E\10-C13\pdata\1\1r		
Frequency (MHz)	100.61	Nucleus	13C	Number of Transients	1024	Origin	Spect
Original Points Count	32768	Owner	sgcnmr	Points Count	32768	Pulse Sequence	jmod
Receiver Gain	194.71	SW(cyclical) (Hz)	24038.46	Solvent	CHLOROFORM-d		
Spectrum Offset (Hz)	10060.3320	Spectrum Type	APT	Sweep Width (Hz)	24037.73	Temperature (degree C)	25.162

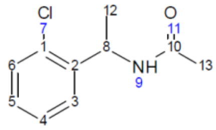

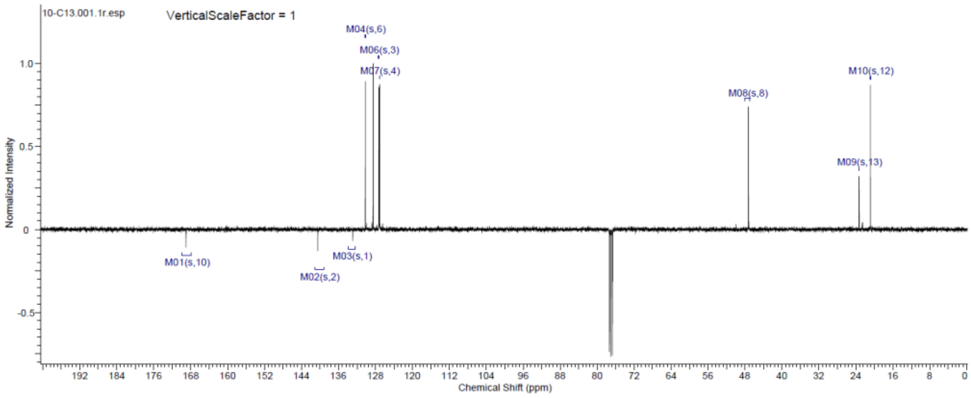

Compound **s34**

UV Detector: 254 Smooth (SG, 1x1)

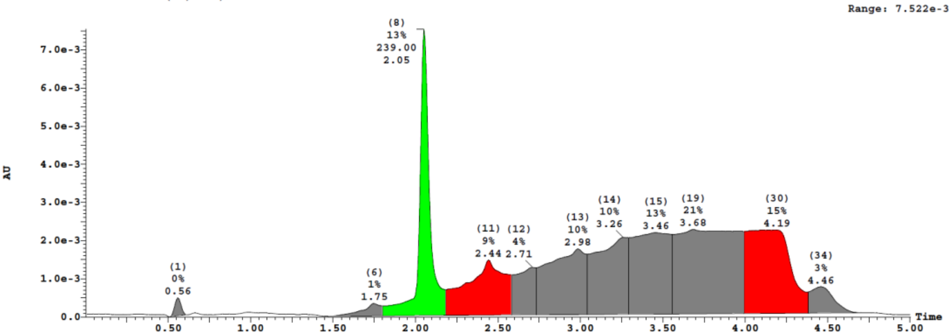

Acquisition Time (sec)	4.0894	Comment	OC 037 B H1	Date	30 Jul 2014 23:06:56		
Date Stamp	30 Jul 2014 23:06:56			File Name	\\hestia\work\chemistry\NMR Files\NMR data\OCox\140730 OC 001-037B\21-H1\pdata\1\1r		
Frequency (MHz)	400.13	Nucleus	1H	Number of Transients	16	Origin	Spect
Original Points Count	32768	Owner	sgcnmr	Points Count	65536	Pulse Sequence	zg30
Receiver Gain	194.71	SW(cyclical) (Hz)	8012.82	Solvent	CHLOROFORM-d		
Spectrum Offset (Hz)	2434.9111	Spectrum Type	STANDARD	Sweep Width (Hz)	8012.70	Temperature (degree C)	25.151

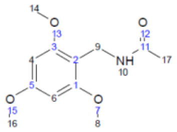

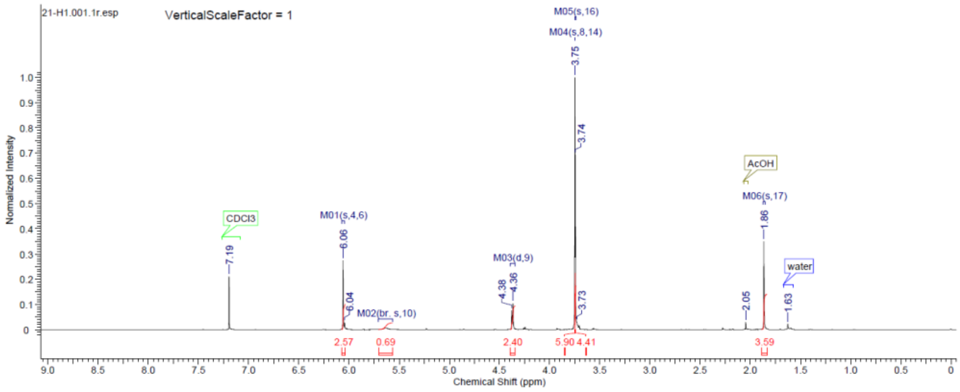

Acquisition Time (sec)	1.3631	Comment	OC 037 B C13	Date	30 Jul 2014 23:02:40		
Date Stamp	30 Jul 2014 23:02:40			File Name	\\hestia\work\chemistry\NMR Files\NMR data\OCox\140730 OC 001-037B\20-C13\pdata\1\1r		
Frequency (MHz)	100.61	Nucleus	13C	Number of Transients	1024	Origin	Spect
Original Points Count	32768	Owner	sgcnmr	Points Count	32768	Pulse Sequence	jmod
Receiver Gain	194.71	SW(cyclical) (Hz)	24038.46	Solvent	CHLOROFORM-d		
Spectrum Offset (Hz)	10060.3320	Spectrum Type	APT	Sweep Width (Hz)	24037.73	Temperature (degree C)	25.159

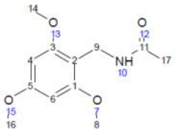

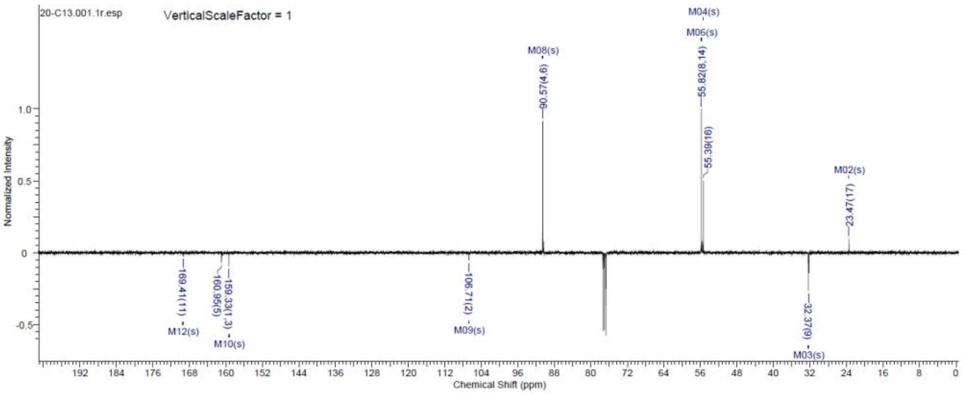

Compound **s35**

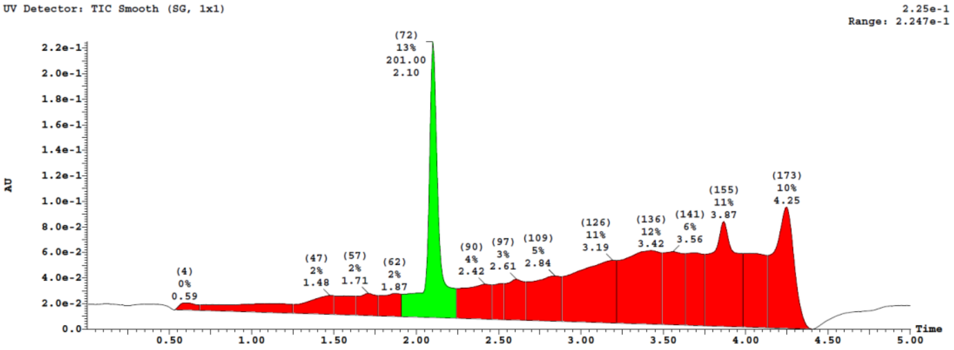

Acquisition Time (sec)	4.0894	Comment	OC_037_D_H1	Date	31 Jul 2014 03:08:00		
Date Stamp	31 Jul 2014 03:08:00			File Name	\\hestia\work\chemistry\NMR Files\NMR_data\OCox\140730_OC_001-037D\11-H1\pdata\1\1r		
Frequency (MHz)	400.13	Nucleus	1H	Number of Transients	16	Origin	Spect
Original Points Count	32768	Owner	sgcnmr	Points Count	65536	Pulse Sequence	zg30
Receiver Gain	194.71	SW(cyclical) (Hz)	8012.82	Solvent	CHLOROFORM-d		
Spectrum Offset (Hz)	2435.1111	Spectrum Type	STANDARD	Sweep Width (Hz)	8012.70	Temperature (degree C)	25.152

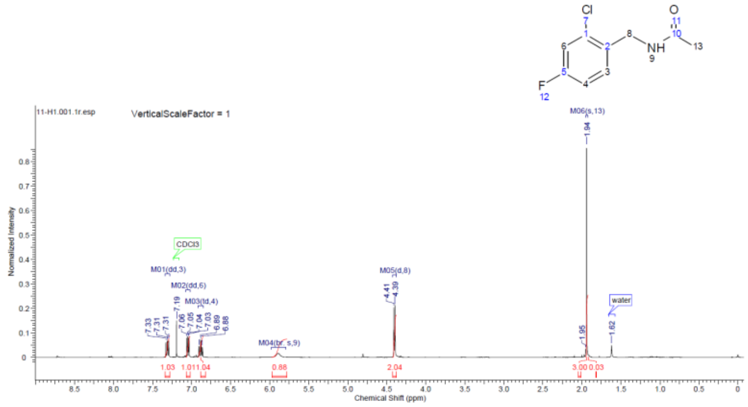

Acquisition Time (sec)	1.3631	Comment	OC_037_D_C13			Date	31 Jul 2014 03:05:52
Date Stamp	31 Jul 2014 03:05:52			File Name	\\hestia\work\chemistry\NMR Files\NMR_data\OCox\140730_OC_001-037D\10-C13\pdata\1\1r		
Frequency (MHz)	100.61	Nucleus	13C	Number of Transients	1024	Origin	Spect
Original Points Count	32768	Owner	sgcnmr	Points Count	32768	Pulse Sequence	jmod
Receiver Gain	194.71	SW(cyclical) (Hz)	24038.46	Solvent	CHLOROFORM-d		
Spectrum Offset (Hz)	10060.3320	Spectrum Type	APT	Sweep Width (Hz)	24037.73	Temperature (degree C)	25.160

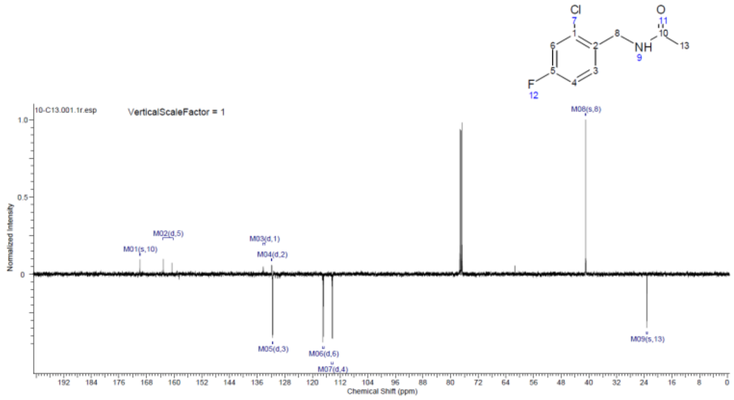

Acquisition Time (sec)	0.7330	Comment	OC_037D_F19	Date	22 Aug 2014 17:36:16		
Date Stamp	22 Aug 2014 17:36:16			File Name	\\hestia\work\chemistry\NMR Files\NMR_data\OCox\140822_OC_001-037D_F19\10\pdata\1\1r		
Frequency (MHz)	376.50	Nucleus	19F	Number of Transients	16	Origin	Spect
Original Points Count	65446	Owner	sgcnmr	Points Count	131072	Pulse Sequence	zgflqn
Receiver Gain	194.71	SW(cyclical) (Hz)	89285.71	Solvent	CHLOROFORM-d		
Spectrum Offset (Hz)	-37649.6211	Spectrum Type	STANDARD	Sweep Width (Hz)	89285.03	Temperature (degree C)	25.161

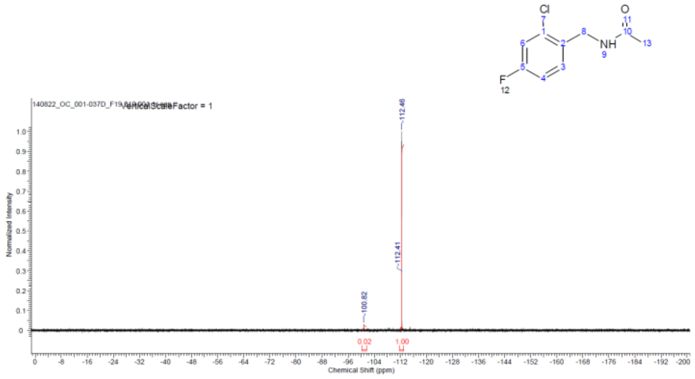

Compound **s36**

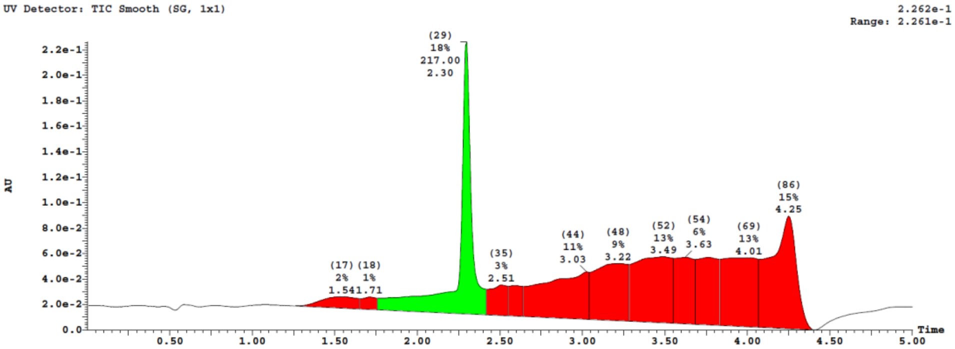

Acquisition Time (sec)	4.0894	Comment	OC_037_C_H1	Date	31 Jul 2014 01:06:24		
Date Stamp	31 Jul 2014 01:06:24			File Name	\\hestia\work\chemistry\NMR Files\NMR_data\OCox\140730_OC_001-037C\21-H1\pdata\1\1r		
Frequency (MHz)	400.13	Nucleus	1H	Number of Transients	16	Origin	Spect
Original Points Count	32768	Owner	sgcnmr	Points Count	65536	Pulse Sequence	zg30
Receiver Gain	194.71	SW(cyclical) (Hz)	8012.82	Solvent	CHLOROFORM-d		
Spectrum Offset (Hz)	2434.6350	Spectrum Type	STANDARD	Sweep Width (Hz)	8012.70	Temperature (degree C)	25.155

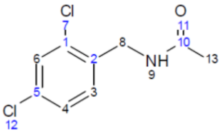

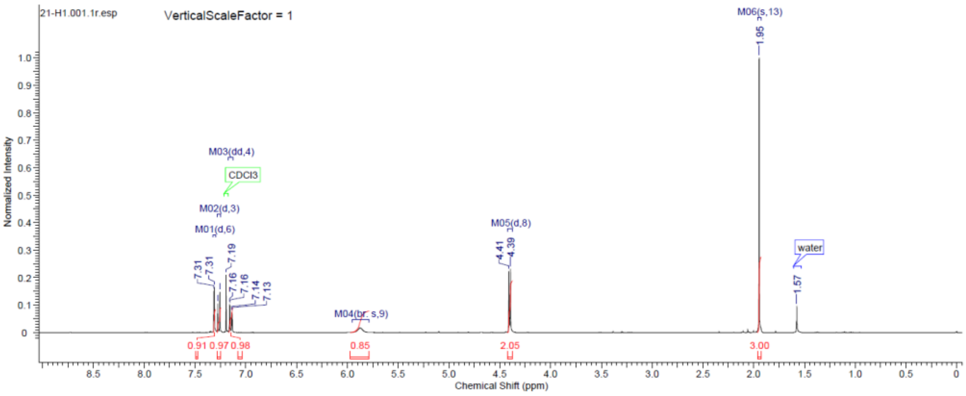

Acquisition Time (sec)	1.3631	Comment	OC_037_C_C13			Date	31 Jul 2014 01:04:16
Date Stamp	31 Jul 2014 01:04:16			File Name	\\hestia\work\chemistry\NMR Files\NMR_data\OCox\140730_OC_001-037C\20-C13\pdata\1\1r		
Frequency (MHz)	100.61	Nucleus	13C	Number of Transients	1024	Origin	Spect
Original Points Count	32768	Owner	sgcnmr	Points Count	32768	Pulse Sequence	jmod
Receiver Gain	194.71	SW(cyclical) (Hz)	24038.46	Solvent	CHLOROFORM-d		
Spectrum Offset (Hz)	9991.2051	Spectrum Type	APT	Sweep Width (Hz)	24037.73	Temperature (degree C)	25.160

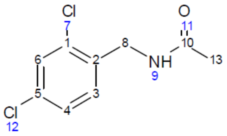

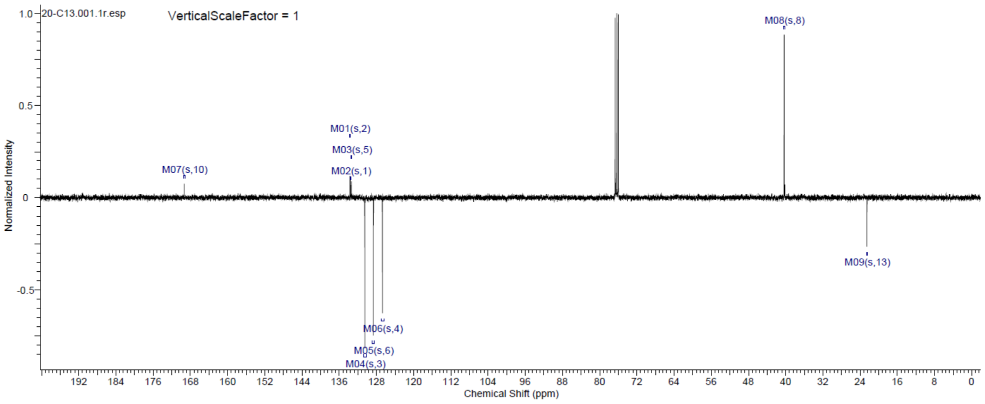

Compound **s37**

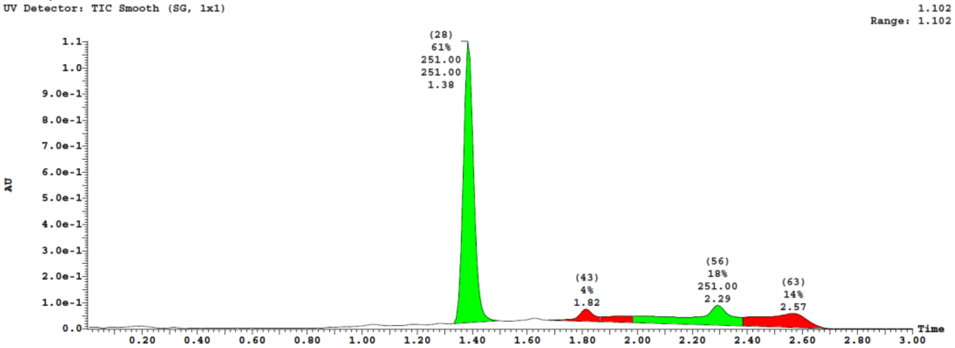

Acquisition Time (sec)	4.0894	Comment	OC 037 F H1	Date	31 Jul 2014 07:09:04		
Date Stamp	31 Jul 2014 07:09:04			File Name	\\hestia\work\chemistry\NMR Files\NMR data\OCox\140730 OC 001-037F\11-H1\pdata\1\1r		
Frequency (MHz)	400.13	Nucleus	1H	Number of Transients	16	Origin	Spect
Original Points Count	32768	Owner	sgcnmr	Points Count	65536	Pulse Sequence	zg30
Receiver Gain	194.71	SW(cyclical) (Hz)	8012.82	Solvent	CHLOROFORM-d		
Spectrum Offset (Hz)	2434.1709	Spectrum Type	STANDARD	Sweep Width (Hz)	8012.70	Temperature (degree C)	25.150

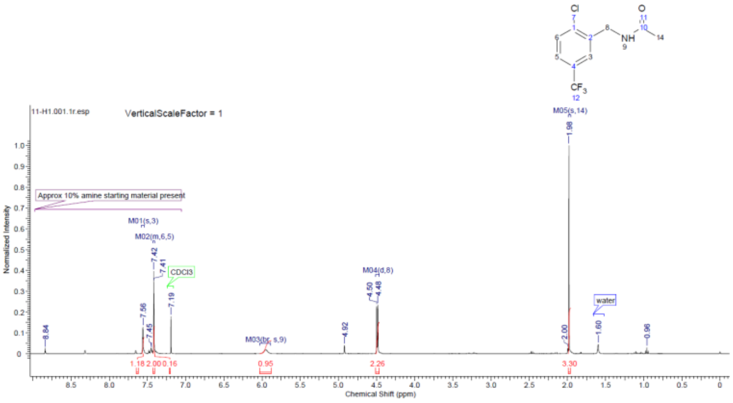

Acquisition Time (sec)	1.3631	Comment	OC 037 F C13			Date	31 Jul 2014 07:04:48
Date Stamp	31 Jul 2014 07:04:48			File Name	\\hestia\work\chemistry\NMR Files\NMR data\OCox\140730 OC 001-037F\10-C13\pdata\1\1r		
Frequency (MHz)	100.61	Nucleus	13C	Number of Transients	1024	Origin	Spect
Original Points Count	32768	Owner	sgcnmr	Points Count	32768	Pulse Sequence	jmod
Receiver Gain	194.71	SW(cyclical) (Hz)	24038.46	Solvent	CHLOROFORM-d		
Spectrum Offset (Hz)	10060.3320	Spectrum Type	APT	Sweep Width (Hz)	24037.73	Temperature (degree C)	25.159

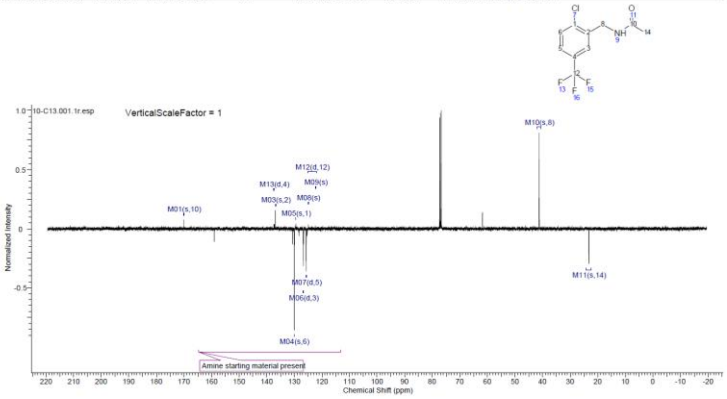

Acquisition Time (sec)	0.7330	Comment	OC 037F F19	Date	22 Aug 2014 17:38:24		
Date Stamp	22 Aug 2014 17:38:24			File Name	\\hestia\work\chemistry\NMR Files\NMR data\OCox\140822 OC 001-037F F19\10\pdata\1\1r		
Frequency (MHz)	376.50	Nucleus	19F	Number of Transients	16	Origin	Spect
Original Points Count	65446	Owner	sgcnmr	Points Count	131072	Pulse Sequence	zgflqn
Receiver Gain	194.71	SW(cyclical) (Hz)	89285.71	Solvent	CHLOROFORM-d		
Spectrum Offset (Hz)	-37649.6211	Spectrum Type	STANDARD	Sweep Width (Hz)	89285.03	Temperature (degree C)	25.159

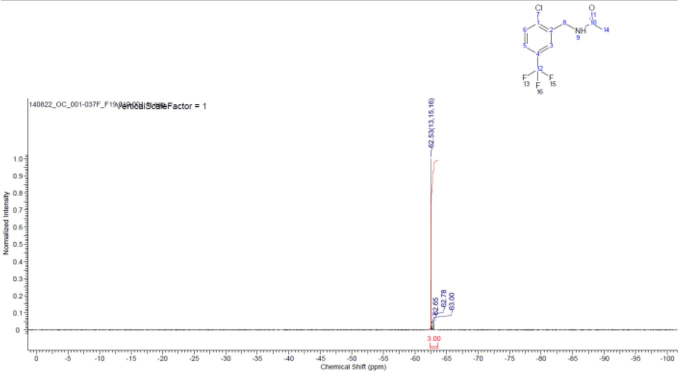

Compound **s39**

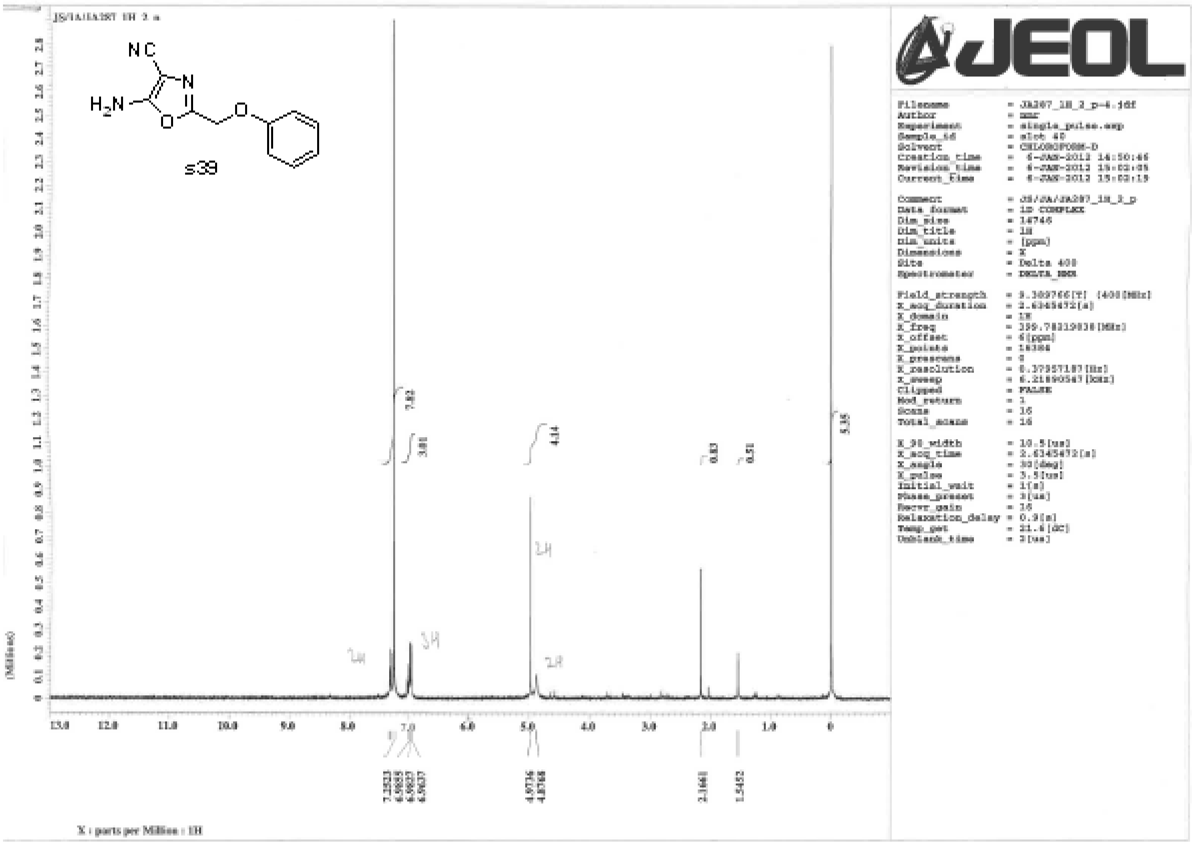

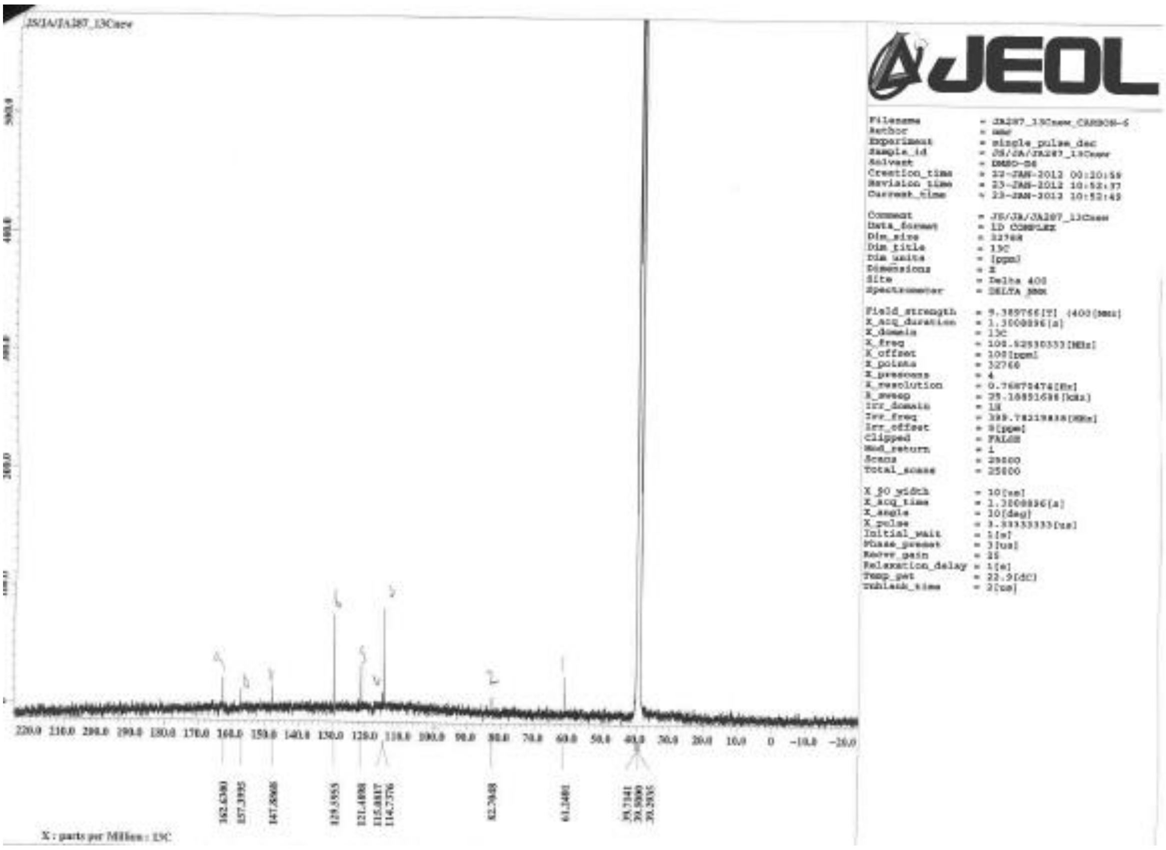

Compound **s40**

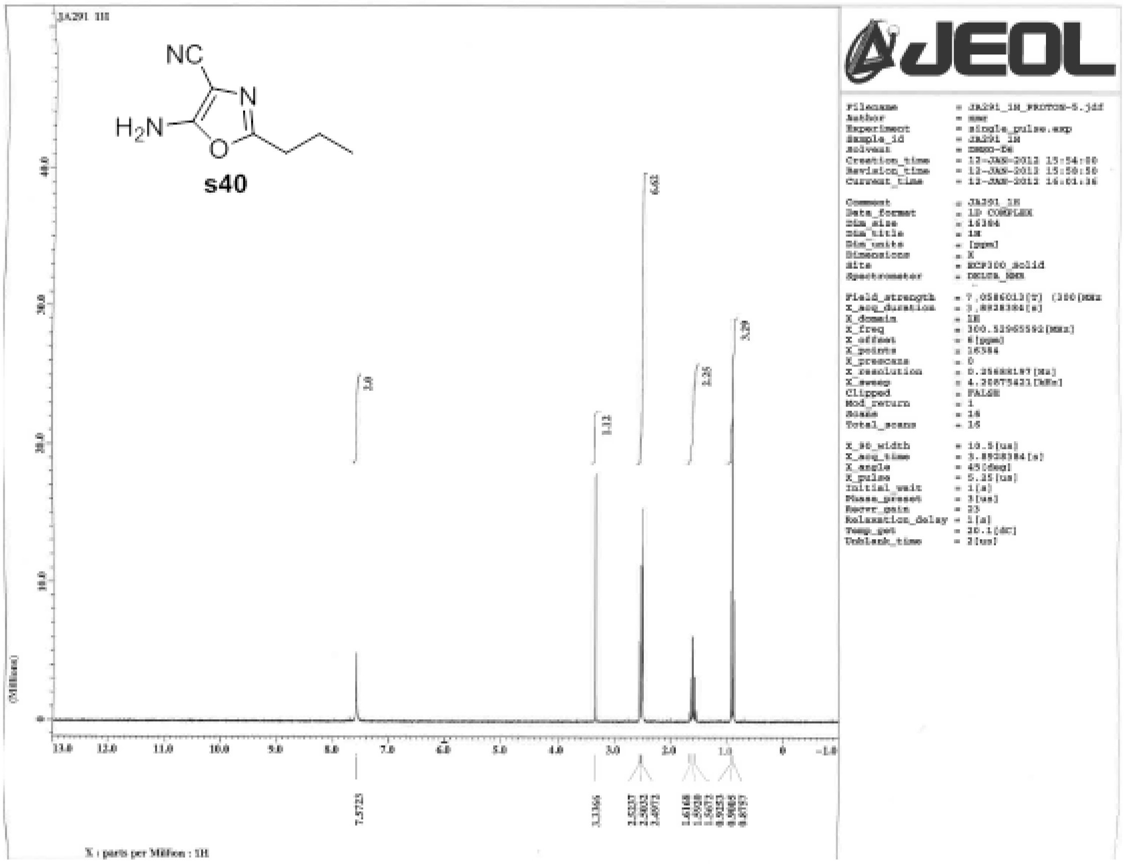

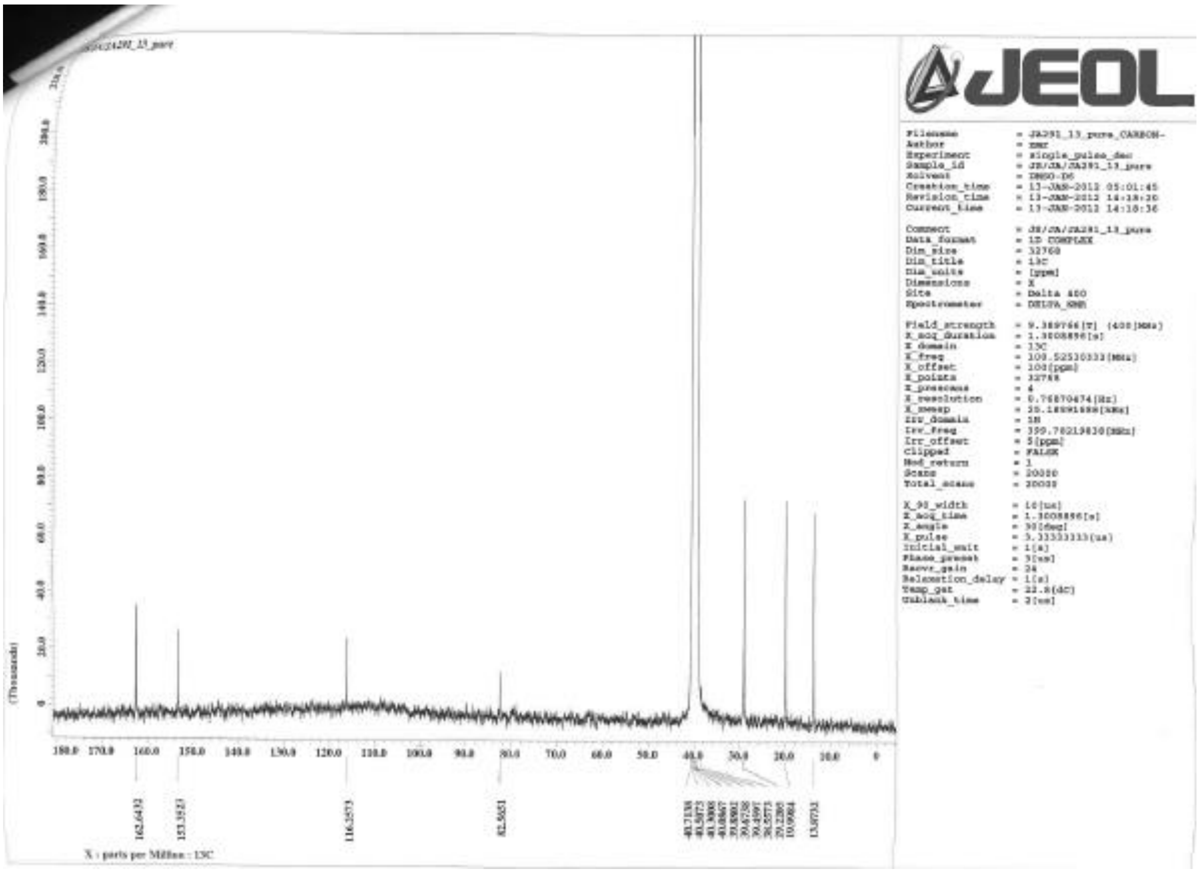

Compound **s46**

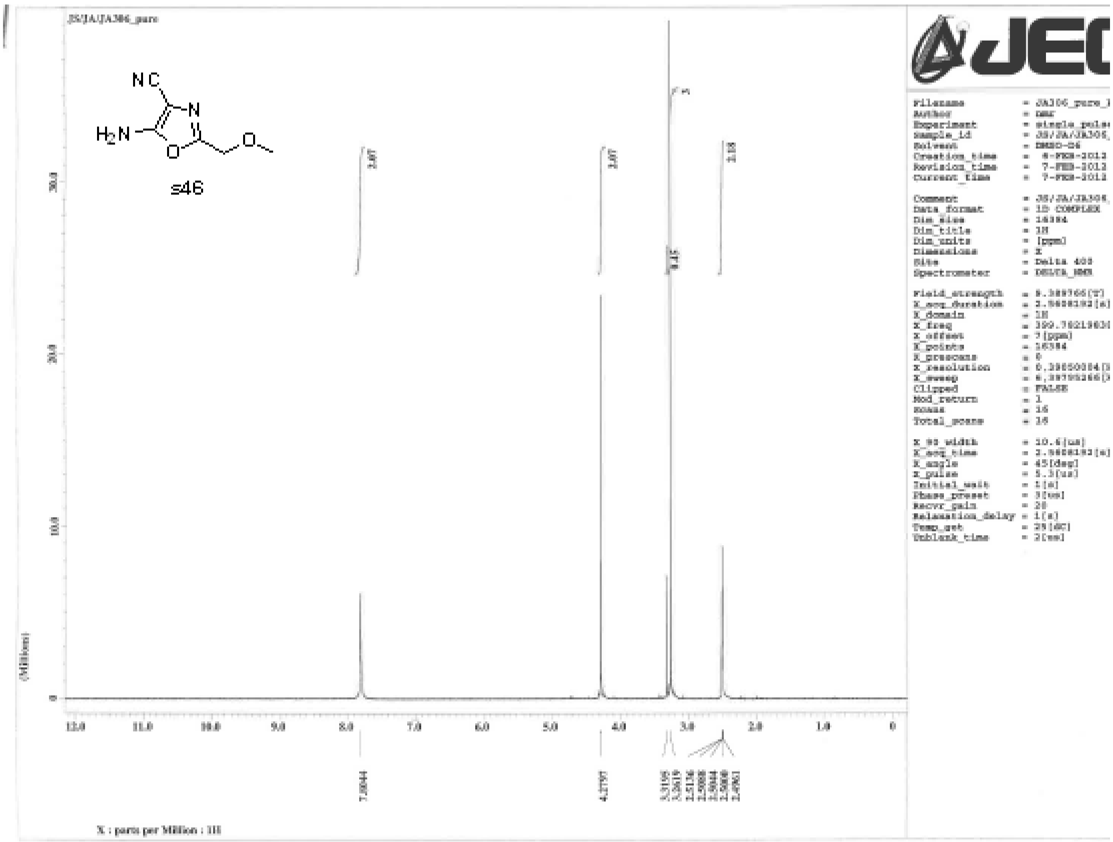

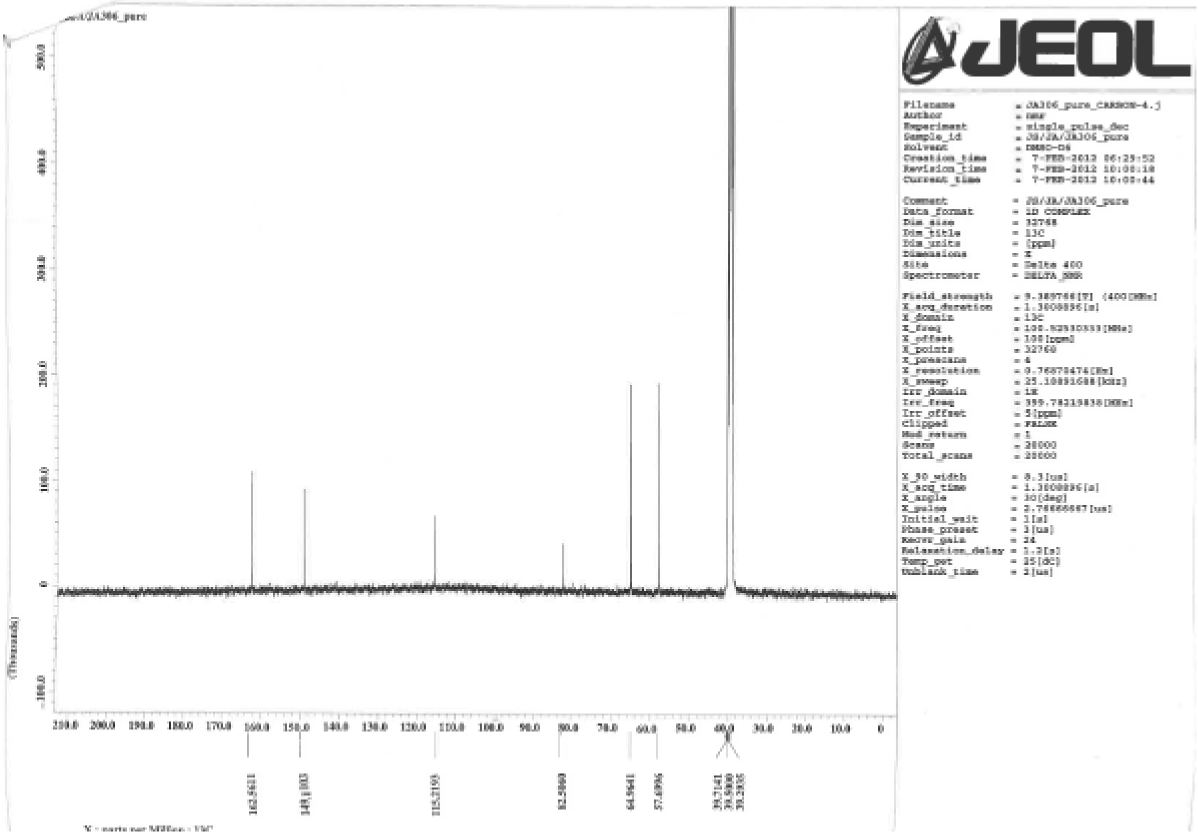

Compound **s47**

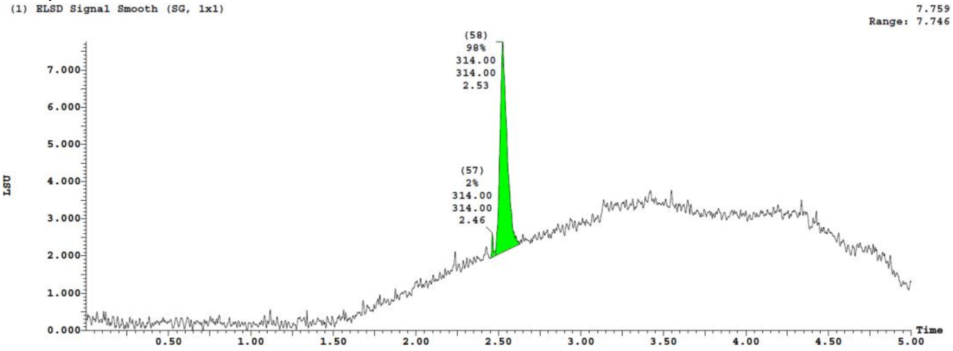

Acquisition Time (sec)	4.0894	Comment	OC_058	Date	13 Jan 2015 17:44:32				
Date Stamp	13 Jan 2015 17:44:32			File Name	\\hestia\work\chemistry\NMR Files\NMR_data\OCox\150113_OC_001-058\10\pdata\1\1r				
Frequency (MHz)	400.13	Nucleus	1H	Number of Transients	16	Origin	Spect	Original Points Count	32768
Owner	sgcnmr	Points Count	65536	Pulse Sequence	zg30	Receiver Gain	155.34	SW(cyclical) (Hz)	8012.82
Solvent	DMSO-d6	Spectrum Offset (Hz)	2470.9668	Spectrum Type	STANDARD	Sweep Width (Hz)	8012.70	Temperature (degree C)	25.158

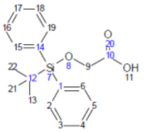

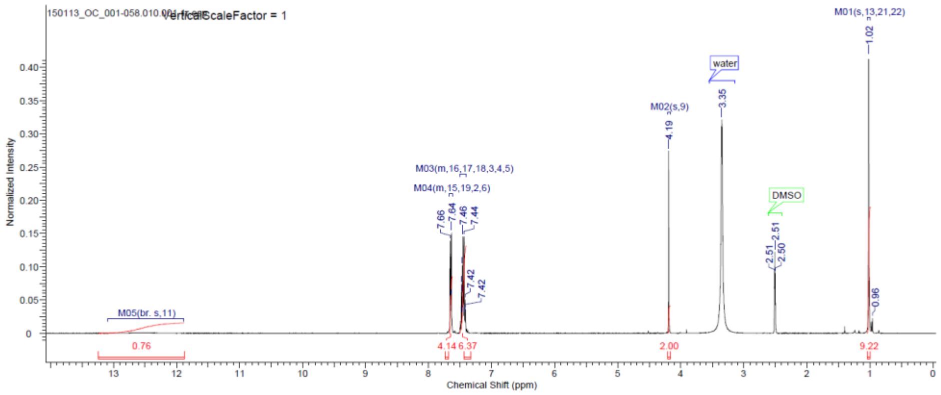

Acquisition Time (sec)	1.3631	Comment	OC_058_C13	Date	14 Jan 2015 00:27:44		
Date Stamp	14 Jan 2015 00:27:44			File Name	\\hestia\work\chemistry\NMR Files\NMR_data\OCox\150113_OC_001-058\11-C13\pdata\1\1r		
Frequency (MHz)	100.61	Nucleus	13C	Number of Transients	1024	Origin	Spect
Original Points Count	32768	Owner	sgcnmr	Points Count	32768	Pulse Sequence	jmod
Receiver Gain	194.71	SW(cyclical) (Hz)	24038.46	Solvent	DMSO-d6	Spectrum Offset (Hz)	10060.3320
Spectrum Type	APT	Sweep Width (Hz)	24037.73	Temperature (degree C)	25.164		

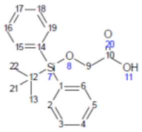

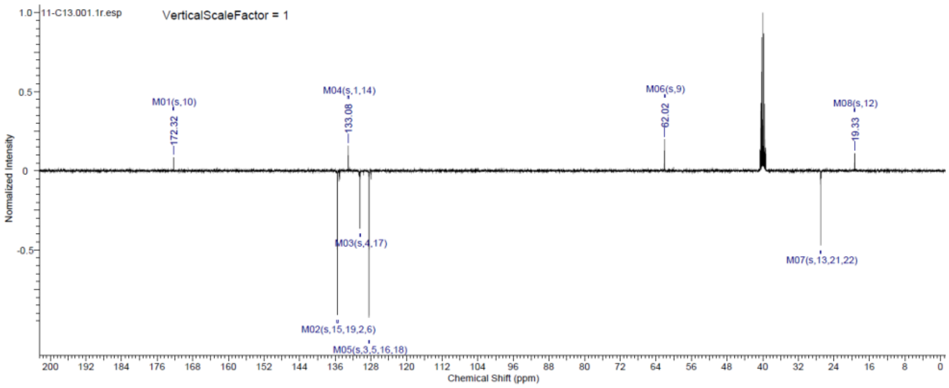

Compound **s48**

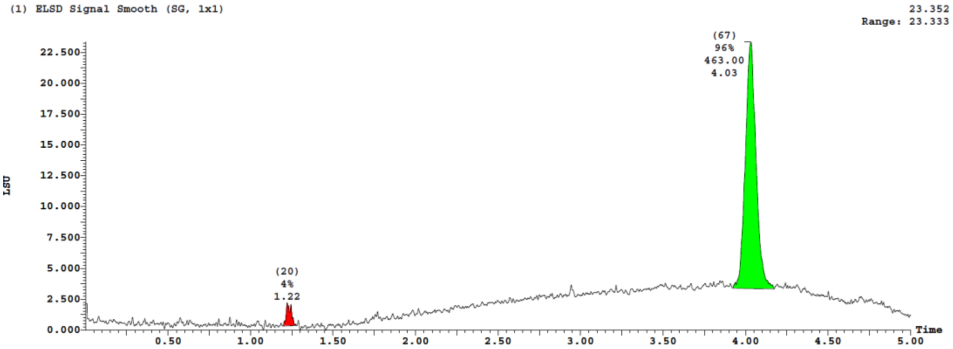

Acquisition Time (sec)	4.0894	Comment	OC_065	Date	21 Jan 2015 12:13:52		
Date Stamp	21 Jan 2015 12:13:52			File Name	\\hestia\work\chemistry\NMR Files\NMR_data\OCox\150121_OC_001-065\10\pdata\1\1r		
Frequency (MHz)	400.13	Nucleus	1H	Number of Transients	16	Origin	Spect
Original Points Count	32768	Owner	sgcnmr	Points Count	65536	Pulse Sequence	zg30
Receiver Gain	194.71	SW(cyclical) (Hz)	8012.82	Solvent	CHLOROFORM-d		
Spectrum Offset (Hz)	2470.9668	Spectrum Type	STANDARD	Sweep Width (Hz)	8012.70	Temperature (degree C)	25.156

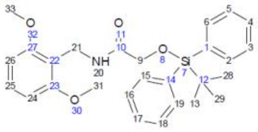

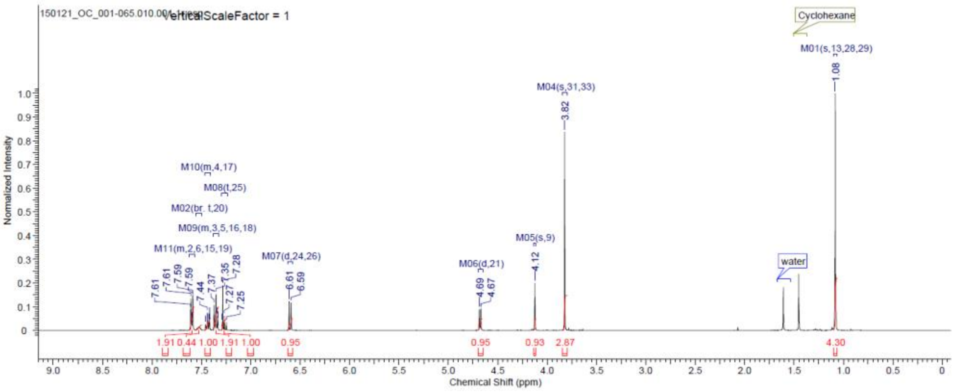

Acquisition Time (sec)	1.3631	Comment	OC_065_C13	Date	21 Jan 2015 22:04:48		
Date Stamp	21 Jan 2015 22:04:48			File Name	\\hestia\work\chemistry\NMR Files\NMR_data\OCox\150121_OC_001-065\11-C13\pdata\1\1r		
Frequency (MHz)	100.61	Nucleus	13C	Number of Transients	1024	Origin	Spect
Original Points Count	32768	Owner	sgcnmr	Points Count	32768	Pulse Sequence	jmod
Receiver Gain	194.71	SW(cyclical) (Hz)	24038.46	Solvent	CHLOROFORM-d		
Spectrum Offset (Hz)	10060.3320	Spectrum Type	APT	Sweep Width (Hz)	24037.73	Temperature (degree C)	25.298

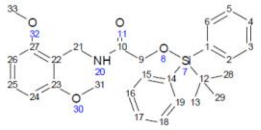

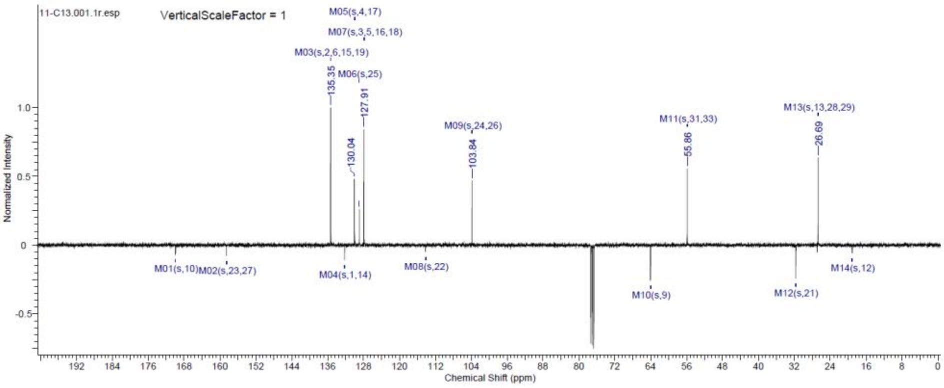

Supplement: Supplementary file 3 [file SC-007-C5SC03115J-s003.pdf]
