# Supplementary material for: A poised fragment library enables rapid synthetic expansion yielding the first reported inhibitors of PHIP(2), an atypical bromodomain
Source: Chem Sci. 2015 Dec 22;7(3):2322–30. doi: 10.1039/c5sc03115j (PMC5977933; doi:10.1039/c5sc03115j)
Supplement: Supplementary file 4 [file SC-007-C5SC03115J-s004.pdf]

Electronic Supplementary Material (ESI) for Chemical Science.
This journal is © The Royal Society of Chemistry 2015

Electronic Supplementary Information

A Poised Fragment Library Enables Rapid Synthetic Expansion Yielding First Ever Inhibitors of PHIP(2), an Atypical Bromodomain

Oakley B. Cox,[a,b,c] Tobias Krojer,[a] Patrick Collins,[c] Octovia Monteiro,[a,b] Romain Talon,[a] Anthony Bradley,[a] Oleg Fedorov,[a,b] Jahangir Amin,[d] John Spencer,[d] Brian D. Marsden,[a,e] Frank von Delft*[a,c,f] and Paul E. Brennan*[a,b]

a. Structural Genomics Consortium (SGC), University of Oxford, Oxford OX3 7DQ, UK.

b. Target Discovery Institute (TDI), Nuffield Department of Medicine, University of Oxford, Oxford OX3 7FZ, UK. E-mail: paul.brennan@sgc.ox.ac.uk

c. Diamond Light Source (DLS), Harwell Science and Innovation Campus, Didcot, OX11 0DE, UK. E-mail: frank.von-delft@diamond.ac.uk

d. Department of Chemistry, School of Life Sciences, University of Sussex, Brighton, BN1 9QJ, UK

e. Kennedy Institute of Rheumatology, Nuffield Department of Orthopaedics, Rheumatology and Musculoskeletal Sciences, University of Oxford, Roosevelt Drive, Headington, Oxford OX3 7FY, U.K.

f. Department of Biochemistry, University of Johannesburg, Aukland Park 2006, South Africa

Table of Contents

Supplemental Figures 3
- Supplemental Figure 1 3
- Supplemental Figure 2 4
- Supplemental Figure 3 4
- Supplemental Figure 4 5
- Supplemental Figure 5 6

Supplemental SAR Tables 6
- Supplementary Table 1 6
- Supplementary Table 2 7
- Supplementary Table 3 8

Synthetic procedures 8
- List of Abbreviations 8
- General Experimental 9
- General Synthetic Procedures 10
- Chemistry Experimental 11

Protein Expression and Purification 26

AlphaScreen 26

Crystallisation 26

Compound Soaking........26
Data Collection and Structure Solution........26
Supplemental Table 4........27
Design of DSPL1 (Diamond-SGC Poised Fragment Library v1.0) 27
Design of DSPL2 (Diamond-SGC Poised Fragment Library v 2.0) 28
References 28

Supplemental Figures

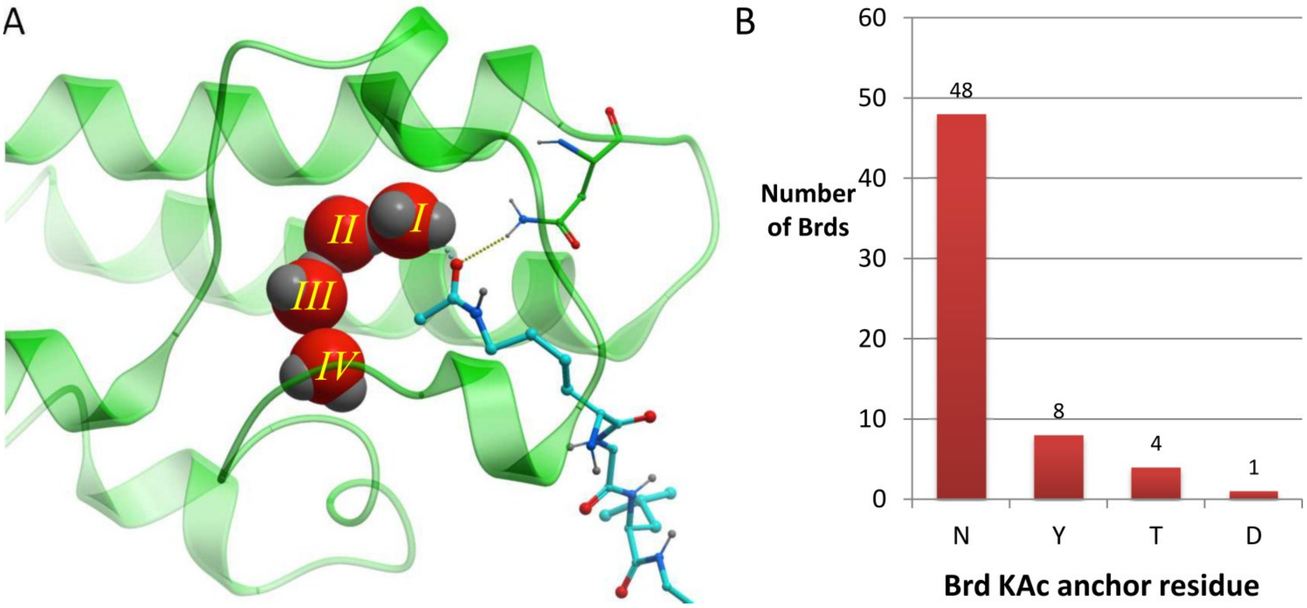

Supplemental Figure 1

(A) Canonical water network and KAc binding in typical bromodomains (Brds). The H4K8AcK12ac peptide (cyan sticks) in complex with Brd4(1) (green ribbon) is anchored by N140 (green sticks) and a network of four waters (red CPK) numbered *I-IV*. The KAc makes H-bonds to N140 and water I (dotted lines) (PDB ID 3UW9). (B) Prevanlence of typical and atypical Brds. The histogram shows the number of Brds with various residue types in the key KAc anchor position across the 61 known human bromodomains. Asparagine (N) is the most widely conserved, but tyrosine (Y), threonine (T) and aspartic acid (D) are also observed.

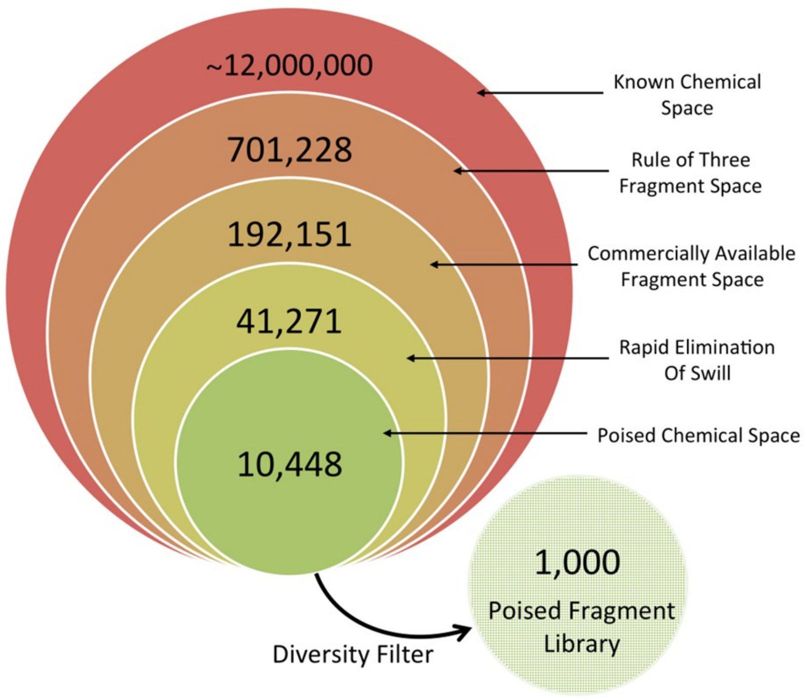

Supplemental Figure 2

Design of DSPL1. The 700k ZINC rule-of-three fragment subset was filtered for commercial availability (n ~192k), non-reactivity using Schrodinger REOS (n ~41k) and presence of a poised group (n ~10k). 1000 diverse compounds were selected based on the nature of the poised group and using USRCAT descriptors.

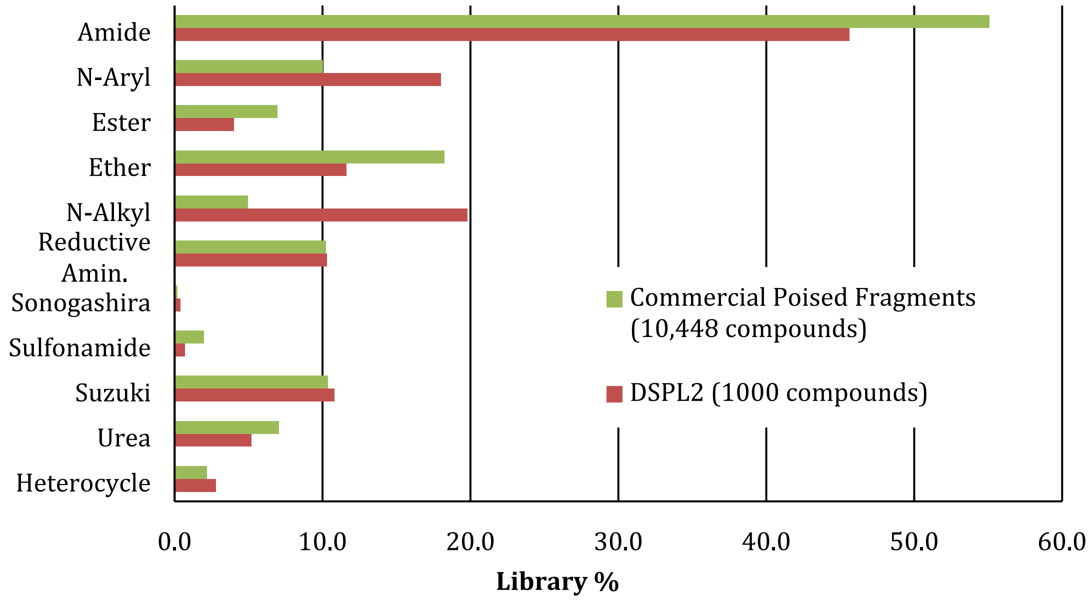

Supplemental Figure 3

Distribution of Poised Motifs in Commercial Poised Fragments & the Diamond SGC Fragment Library 2.0 (DSPL2)

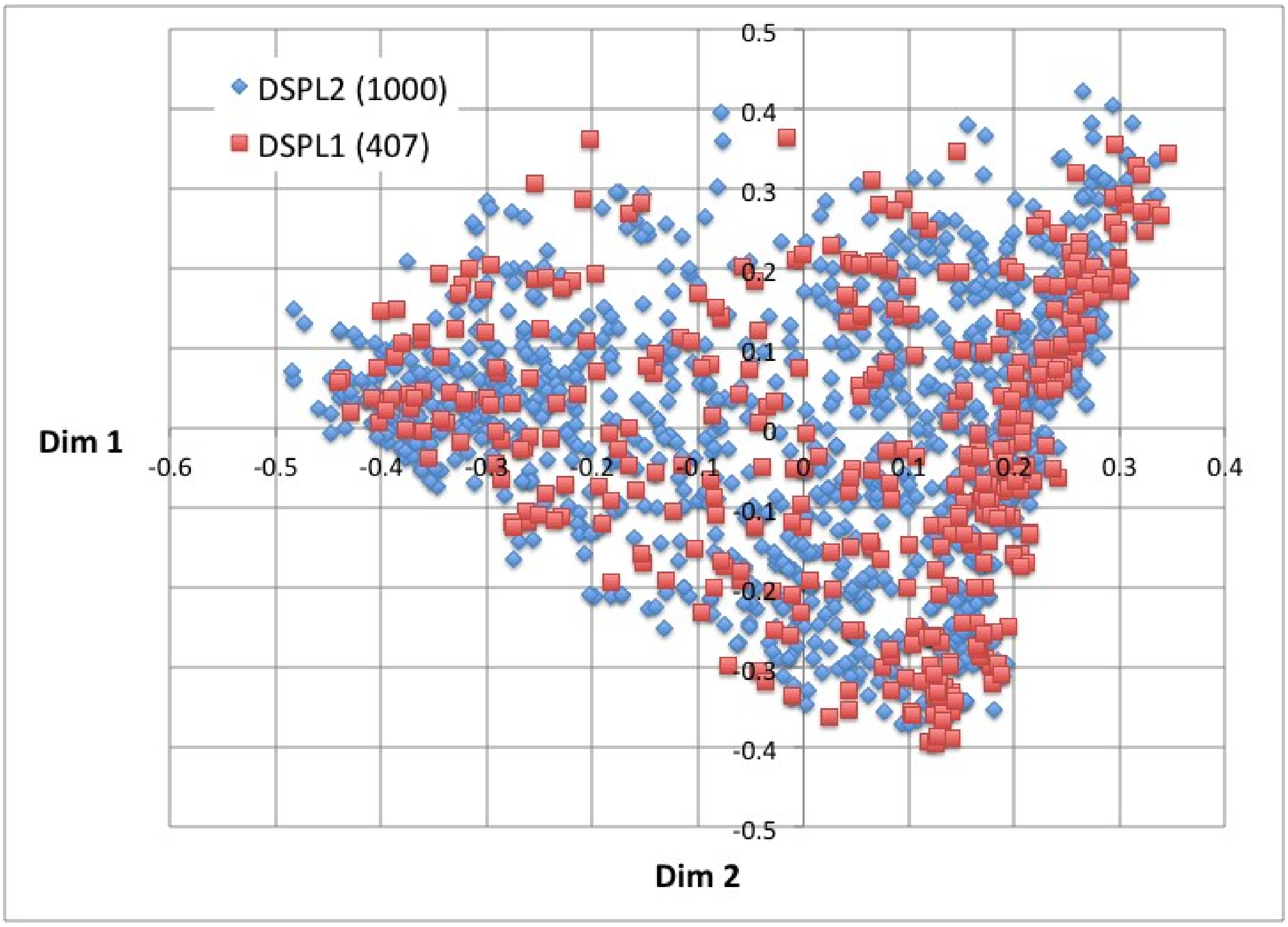

Supplemental Figure 4

Distribution in chemical space of DSPL1 and DSPL2. MACCS fingerprints were computed for all compounds and a pairwise matrix generated which was reduced to 2 dimensions for visualization. The analysis was done using Knime with Schrodinger nodes: Canvas Fingerprint Generation, Generate Pairwise Matrix and Multi-dimensional Scaling.

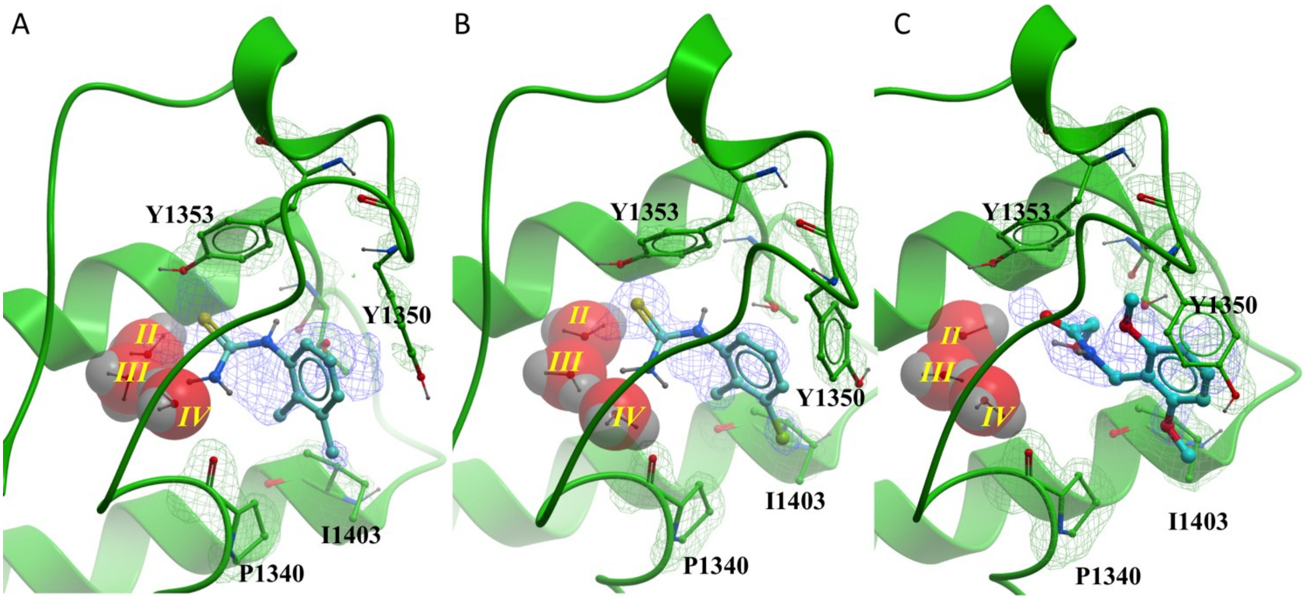

Supplemental Figure 5

Structures of compounds **6**, **8** and **15** in PHIP(2). Compounds: cyan sticks and blue mesh (2Fo-Fc). Protein: green sticks and green mesh (2Fo-Fc).

Supplemental SAR Tables

Supplementary Table 1

Synthesis and Screening of Thiourea series

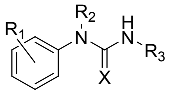

Thiourea series

Compound Number	R_1	R_2	R_3	X	Yield[a]	Soaking Outcome[c]	PHIP(2) pIC_{50}[d]	LE
**1**	2-Me	H	H	S	66%	Success	3.11 ± 0.06 (2)	0.40
**5**	2-Me-3-OMe	H	H	S	71%	Error	2.97 ± 0.18 (2)	0.32
**6**	2,3-Me_2	H	H	S	12%	Success	3.78 ± 0.06 (2)	0.45
**7**	2-Me-3-CF_3	H	H	S	33%	NL	3.85 ± 0.21 (2)	0.36
**8**	2-Me-3-Cl	H	H	S	26%	Success	3.59 ± 0.03 (2)	0.42
**9**	2,6-Me_2	H	H	S	8%	NL	3.32 ± 0.08 (2)	0.39
**10**	2-Me	H	Me	S	14%	NL	3.38 ± 0.30 (2)	0.39
**11**	2-Cl	H	Me	S	19%	NL	3.89 ± 0.05 (2)	0.45
**s1**	2-Me	H	Et	S	74%	NL	<2.30 (2)	<0.25
**s2**	2-Me	H	*i*-Pr	S	78%	NL	2.69 ± 0.63 (2)	0.27
**s3**	2-Me	H	$(CH_2)_2OH$	S	69%	Error	<2.30 (2)	<0.23
**s4**	2-Me	H	Me_2	S	65%	NL	<2.30 (2)	<0.25
**s5**	2-Cl	H	H	S	29%	NL	2.77 ± 0.14 (2)	0.35
**s6**	2-OMe	H	H	S	19%	Error	<2.30 (2)	<0.27
**s7**	2-CF_3	H	H	S	NI			
**s8**	2,4-Me_2	H	H	S	32%	Error	2.91 ± 0.06 (2)	0.34
**s9**	2,5-Me_2	H	H	S	44%	NL	3.13 ± 0.06 (2)	0.36
**s10**	2-Me-3-F	H	H	S	45%	NL	2.49 ± 0.07 (2)	0.29
**s11**	2-OMe-6-Me	H	H	S	NI			
**s12**	2-Cl-6-Me	H	H	S	NI			
**s13**	2,6-Me_2-3-Cl	H	H	S	36%	NL	2.66 ± 0.06 (2)	0.29
**s14**	2,3-Me_2	H	Me	S	33%	NL	<2.30 (2)	<0.25
**s15**	2-Me-3-Cl	H	Me	S	44%	NL	2.68 ± 0.14 (2)	0.29
**s16**	2-OMe	H	Me	S	47%	NL	<2.30 (2)	<0.25
**s17**	2-Cl-6-Me	H	Me	S	NI			
**s18**	2-OMe-6-Me	H	Me	S	48%	NL	<2.30 (2)	<0.23
**s19**	2,6-Me_2-3-Cl	H	Me	S	12%	NL	2.58 ± 0.11 (2)	0.26
**s20**	2-Me	Me	H	S	NI			
**s21**	2-Me	H	H	O	Purchased	NL	2.73 ± 0.71 (2)	0.35
**s22**	2,3-Me_2	H	H	O	49%[b]	NL	2.65 ± 0.05 (2)	0.31
**s23**	2-Me-3-Cl	H	H	O	52%[b]	NL	2.71 ± 0.75 (2)	0.32
**s24**	2-Cl	H	H	O	42%[b]	NL	<2.30 (2)	<0.29
**s25**	2-OMe	H	H	O	46%[b]	NL	2.49 ± 0.08 (2)	0.29
**s26**	2-Cl-6-Me	H	H	O	NI[b]			
**s27**	2-OMe-6-Me	H	H	O	19%[b]	NL	2.47 ± 1.04 (2)	0.27
**s28**	2,6-Me_2-3-Cl	H	H	O	NI[b]			

[a]Synthesised using procedure described in scheme 1 unless stated. NI: not isolated. [b]Reagents and conditions: KOCN, AcOH, H_2O, rt. [c]NL: No ligand found in model. Error: Experiment failure during soaking, at beamline or during data processing or model refinement. [d]By AlphaScreen peptide displacement assay.

Supplementary Table 2

Synthesis and screening of *N*-benzyl amide series

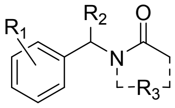

Compound Number	R_1	R_2	R_3[a]	Yield[b]	Soaking Outcome[g]	PHIP(2) pIC_{50}[h]	LE
**2**	2,6-Cl_2	H	*C*-Me	44%	Success	<2.30 (2)	<0.25
**12**	2,6-$(OMe)_2$	H	*C*-Me	79%	Success	3.72 ± 0.04 (2)	0.35
**13**	2,6-Cl_2	H	*C*-CH_2OH	15%[c]	Success	<2.30 (2)	<0.23
**14**	2,6-Cl_2	H	*N*-Me, *C*-Me	83%	Success	<2.30 (2)	<0.23
**15**	2,6-$(OMe)_2$	H	*C*-CH_2OH	23%[d]	Success	<2.30 (2)	<0.20
**16**	2,6-$(OMe)_2$	H	5-lactam	6%[e]	NL	3.25 ± 0.04 (2)	0.27
**17**	2,6-$(OMe)_2$	H	6-lactam	18%[e]	NL	3.51 ± 0.04 (2)	0.27
**s29**	2,6-Cl_2	H	*C*-Et	82%	NL	<2.30 (2)	<0.23
**s30**	2,6-Cl_2	H	*C*-^{i}Pr	82%	NL	<2.30 (2)	<0.21
**s31**	2,6-Cl_2	H	*C*-CF_3	67%	NL	<2.30 (2)	<0.20
**s32**	2-Cl	Me	*C*-Me	87%	NL	2.45 ± 5.68 (2)	0.26
**s33**	2,6-Cl_2	H	*C*-$NHCH_3$	NI[f]			
**s34**	2,4,6-$(OMe)_3$	H	*C*-Me	83%	NL	<2.30 (2)	<0.19
**s35**	2-Cl-4-F	H	*C*-Me	82%	NL	<2.30 (2)	<0.25
**s36**	2,4-Cl_2	H	*C*-Me	84%	NL	<2.30 (2)	<0.25
**s37**	2-Cl-5-CF_3	H	*C*-Me	65%	NL	<2.30 (2)	<0.20

[a]R_3 = *N*-H unless stated. [b]Synthesised using procedure described in scheme 1 unless stated. NI: not isolated. [c]Reagents and conditions: EDC, DMAP, triethylamine, DMF, rt. [d]Over three steps. See supplementary material for details. [e]Triethylamine, AcN, rt. [f]CDI, triethylamine, DCM, rt. [g]NL: No ligand found in model. [h]By AlphaScreen peptide displacement assay.

Supplementary Table 3

Synthesis and screening of oxazoles

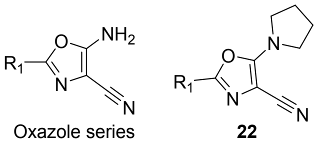

Compound Number	R_1	Yield[a]	Soaking Outcome[c]	PHIP(2) pIC_{50}[d]	LE
**3**	Bn	62%	Success	3.23 ± 0.10 (2)	0.30
**4**	^{i}Bu	42%	Success	3.57 ± 0.10 (2)	0.42
**18**	^{c}Pr	93%	NL	3.48 ± 0.03 (2)	0.44
**19**	^{i}Pr	54%	NL	<2.30 (2)	<0.29
**20**	4-Cl-Bn	46%	Error	3.95 ± 0.03 (2)	0.35
**21**	^{c}Hex	71%	NL	3.31 ± 0.06 (2)	0.33
**22**	^{c}Hex	30%[b]	NL	3.71 ± 0.16 (2)	0.29
**s38**	Phenyl	95%	NL	No Data	
**s39**	CH_2OPh	81%	NL	3.34 ± 0.05 (2)	0.29
**s40**	^{n}Pr	32%	NL	3.02 ± 0.03 (2)	0.38
**s41**	4-F phenyl	96%	NL	3.65 ± 0.03 (2)	0.34
**s42**	4-OMe phenyl	94%	NL	3.51 ± 0.04 (2)	0.31
**s43**	2-OMe phenyl	75%	NL	3.23 ± 0.03 (2)	0.28
**s44**	Thiophen-2-yl	93%	NL	3.31 ± 0.09 (2)	0.36
**s45**	Furan-2-yl	77%	NL	3.19 ± 0.06 (2)	0.34
**s46**	CH_2OCH_3	69%	NL	2.34 ± 0.08 (2)	0.30

[a]Synthesised using procedure described in scheme 1. [b]Over three steps. See Spencer *et al.* for details.[1] [c]NL: No ligand found in model. [d]By AlphaScreen peptide displacement assay.

Synthetic procedures

List of Abbreviations

Ac – Acetate

Aq. – Aqueous

Boc – *tert*-Butoxycarbonyl

DMF – Dimethylformamide

DMSO – Dimethyl sulphoxide

ESI – Electrospray Ionisation

EtOAc – Ethyl acetate

FTIR – Fourier Transform Infra Red

LCMS – Liquid Chromatography Mass Spectrometry

HPLC – High Performance Liquid Chromotography

HRMS – High Resolution Mass Spectrometry

LRMS – Low Resolution Mass Spectrometry

HNESP – High-resolution Nano-ElectroSpray Positive

Ph – Phenyl

MeCN – Acetonitrile

Mp – Melting point

MS – Mass spectrometry

NMR – Nuclear Magnetic Resonance
THF – Tetrahydrofuran
TLC – Thin Layer Chromatography
TOF – Time of flight
t_r – Retention time
UV – Ultra violet

General Experimental

All reactions involving moisture sensitive reagents were carried out under a nitrogen atmosphere using standard vacuum line techniques and glassware that was flame dried. Solvents were dried following the procedure outlined by Grubbs & co-workers.[2] Water was deionized by an Elga DV 25 system. All other solvents and reagents were used as supplied (analytical or HPLC grade) without prior purification. Organic layers were dried over Na_2SO_4 or $MgSO_4$. Parallel work-ups were carried out using a Radleys stacker and Isolute phase separation cartridges. Thin layer chromatography was performed on aluminium plates coated with 60 F_{254} silica gel. Plates were visualised using UV light (254 nm) or 1% aq. $KMnO_4$. Flash column chromatography was performed on a Biotage Isolera One flash column chromatography platform unless stated. Purification by HPLC was performed using a Waters SFO with 515 HPLC pump and Waters Binary Gradient 2545 device (5%-95% solvent A (18% water, 80% acetonitrile, 2% 0.5 M ammonium acetate pH 6.0) in solvent B (93% water, 5% acetonitrile, 2% ammonium acetate pH 6.0)). Product was detected using a SQ Detector 2 and collected using a Waters Sample Manager 2767. Melting points were recorded on a Stuart SMP40 apparatus or a Barnstead Electrothermal 9100 machine and are uncorrected. IR spectra were recorded on a Nicolet iS5 with an iD7 ATR module, neat. Selected characteristic peaks are reported in cm^{-1}. NMR spectra were recorded on a Bruker Avance spectrometer or Varian spectrometer in the deuterated solvent stated. The field was locked by external referencing to the relevant deuteron resonance. Chemical shifts (δ) are reported in ppm and coupling constants (*J*) in Hz. Spectra were recorded at room temperature unless otherwise stated. Coupling constants (*J*) are quoted in Hz and are recorded to the nearest 0.1 Hz. When peak multiplicities are reported, the following abbreviations are used: s = singlet, d = doublet, t = triplet, m = multiplet, br = broadened, dd = doublet of doublets, dt = doublet of triplets.m/z values are reported in Daltons. Compound purity was assessed by elemental analysis (Elemental Analysis Service, London Metropolitan Univeriosity) or by LCMS using either UV absorbance at 254 nm (Waters UV/Visible Detector 2489), ELSD signal (Waters ELS Detector 2424) or ESI+ TIC (SQ Detector 2). The detection method was chosen which gave the strongest signal. LCMS t_r are quoted to the nearest 0.1 min. LCMS was performed on the following system: Kinetex 5µ EVO C18 100A 100 x 3.0 mm column using a linear gradient of solvent A (93 % H_2O, 5 % acetonitrile, and 2 % of 0.5 M ammonium acetate pH 6.0) and solvent B (18 % H_2O, 80 % acetonitrile, and 2 % of 0.5 M ammonium acetate pH 6.0), eluting at a flow rate of 2 mL/min: 5% B over 0.35 min, 5% B to 95% B over 1 min, 95% B over 0.75 min, 95% to 5% B over 0.1 min and 5% B over 0.8 min. LRMS were recorded by LCMS on a Waters SQ Detector 2; data acquisition and processing was performed using Waters FractionLynx software. HRMS was performed by the EPSRC UK National Mass Spectrometry Facility, University of Swansea using HNESP or run on a Agilent 6530 Accurate Mass Q-TOF; data acquisition and processing was performed using Agilent MassHunter Workstation software.

General Synthetic Procedures

General Procedure 1: **Thiourea synthesis using 1,1′-Thiocarbonyldiimidazole[3]**

The first amine (0.6 mmol, 1.0 eq) was added to a stirred solution of 1,1′-thiocarbonyldiimidazole (100 mg, 0.6 mmol, 1.0 eq) in CH_2Cl_2 (2 mL). The reaction mixture was stirred for 1-2 h at room temperature. The second amine (0.6 mmol, 1.0 eq) was added to the reaction mixture and stirred overnight at room temperature. The crude product was washed with aq. HCl (1M, 5 mL) and water (5 mL). The organic layer was dried and purified by flash column chromatography (12%-100% EtOAc in Cyclohexane). Solvent was removed *in vacuo* to afford the desired product.

General Procedure 2: **Amide coupling between an acyl chloride and an amine**

The amine (0.5 mmol, 1.0 eq) and triethylamine (224 µL, 1.6 mmol, 3 eq) were dissolved in CH_2Cl_2 (2 mL). The acyl chloride (0.6 mmol, 1.05 eq) was added and the resulting reaction mixture stirred overnight at room temperature. The crude mixture was diluted with aq. NaOH (10%, 5 mL). The organic layer was separated, washed with water (5 mL) and dried. The solvent was removed *in vacuo* to afford the desired product.

General Procedure 3: **Oxazole formation from an acyl chloride and aminomalononitrile 4-toluenesulfonate[1]**

In a 35 mL microwave tube equipped with a magnetic stirrer, aminomalononitrile 4-toluenesulfonate (1.17 g, 4.60 mmol, 1.0 eq), *N*-methylpyrrolidone (12 mL) and the acyl chloride (5.06 mmol, 1.1 eq) were added. The vessel was then sealed using a rubber microwave septum and then placed into the microwave cavity. The reaction mixture was irradiated with 200 W of power and heated to 120°C for 20 min (dynamic profile with cooling). When at 120°C it was held by moderation of power for 20 min. The vessel was then cooled to room temperature. The reaction mixture was extracted with ethyl acetate (100 mL) and was initially washed with deionised water (2x100 mL), followed by sat. Na_2CO_3 (2 x 100 mL). The organic layer was then dried with $MgSO_4$, filtered using fluted filter paper and the solvent removed *in vacuo*. Using a combiflash R75 the crude reaction mixture was purified using 2:8 ethyl acetate/ CH_2Cl_2 as the elutant to give the desired product.

General Procedure 4: **Urea synthesis using potassium cyanate**

The amine (0.7 mmol, 1.0 eq) was added to a stirred solution of potassium cyanate (60 mg, 0.7 mmol, 1.0 eq) in acetic acid (1 mL, 1.3 mmol, 1.7 eq). The reaction mixture was stirred for 5 h at room temperature. The reaction mixture was filtered and the filtrand purified by reverse phase HPLC. Solvent was removed *in vacuo* to afford the desired product.

The synthesis and characterisation of compounds **18**, **20**, **21**, **s38** and **s41-s46** are all described in *Tetrahedron Letters*, **2012**, 53, 1656-1659.[1]

LCMS traces and NMR spectras for all compounds described below are included in the supplementary material.

Chemistry Experimental

1-(o-tolyl)thiourea **1**

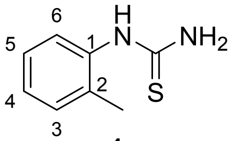

1

Following *General Procedure 1*, 1,1′-thiocarbonyldiimidazole (166 mg, 0.9 mmol, 1.0 eq) was treated with *o*-toluidine (99 µL, 0.9 mmol, 1.0 eq) followed by ammonia methanol solution (133 µL, 7 M, 1.0 eq) to afford *thiourea* **1** as white crystals (102 mg,

66%). Mp 162-163 °C (Lit.[4] 161 °C); ν_{max} (FTIR) cm^{-1} 3422 (NH_2), 3152 (NH), 1615, 1526 (C=S), 1286 (-CSNH-), 759 (aromatic CH); ^1H NMR (400 MHz, DMSO-d_6) δ 9.20 (br. s, 1H, N*H*), 7.22 - 7.26 (m, 1H, C(3)*H*), 7.12 - 7.21 (m, 3H, C(4)*H* & C(5)*H* & C(6)*H*), 2.18 (s, 3H, C*H*$_3$), (N*H*$_2$ not observed); ^{13}C NMR (101 MHz, DMSO-d_6) δ 182.1 (*C*=S), 137.6 (*C*(1) or *C*(2), 134.9 (*C*(1) or *C*(2), 131.0 (*C*(3)), 128.0 (*C*(6)), 127.1 (*C*(4) or *C*(5)), 126.8 (*C*(4) or *C*(5)), 18.1 (*C*H$_3$); *m/z* (ESI^+) 167 ($[M+H]^+$, 100%); HRMS (ESI^+) $C_8H_{11}N_2S^+$, ($[M+H]^+$) requires 167.0637, found 167.0633.

N-(2,6-dichlorobenzyl)acetamide **2**

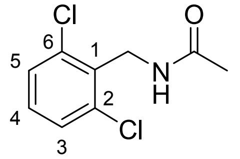

2

Following *General Procedure 2*, acetyl chloride (119 μL, 1.7 mmol, 2.5 eq) was treated with 2,6-dichlorobenzylamine (120 mg, 0.7 mmol, 1.0 eq) and triethylamine (2.9 mL, 20.8 mmol, 30 eq) to afford *amide* **2** as yellow crystals (66 mg, 44%). Mp 182-183 °C; ν_{max} (FTIR) cm^{-1} 3249 (NH), 3077 (CH_3), 1630 (C=O), 1561 (C=O), 1434, 780 (C-Cl or aromatic CH), 766 (C-Cl or aromatic CH); ^1H NMR (400 MHz, DMSO-d_6) δ 8.02 (br. s, 1H, N*H*), 7.49 (d, *J* = 7.8 Hz, 2H, C(3)*H* & C(5)*H*), 7.32 - 7.40 (m, 1H, C(4)*H*), 4.47 (d, *J* = 4.7 Hz, 2H, C*H*$_2$), 1.81 (s, 3H, C*H*$_3$); ^{13}C NMR (101 MHz, DMSO-d_6) δ 169.4 (*C*=O), 136.0 (*C*(2) & *C*(6)), 133.9 (*C*(1)), 130.7 (*C*(4)), 129.0 (*C*(3) & *C*(5)), 39.3 (*C*H$_2$), 22.7 (*C*H$_3$); *m/z* (ESI^+) 218 ($[M(^{35}Cl,^{35}Cl)+H]^+$, 100%), 220 ($[M(^{35}Cl,^{37}Cl)+H]^+$, 75%), 222 ($[M(^{37}Cl,^{37}Cl)+H]^+$, 14%); HRMS (ESI^+) $C_9H_9{}^{35}Cl_2NONa^+$, ($[M+Na]^+$) requires 239.9953, found 239.9947.

5-Amino-2-benzyl-oxazole-4-carbonitrile **3**

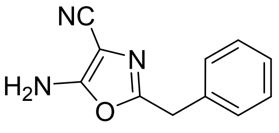

3

Following *General Procedure 3*, phenylacetylchloride (0.67 mL, 5.06 mmol) was treated with aminomalononitrile 4-toluenesulfonate to give *oxazole* **3** (0.58 g, 63% yield) as a white solid. Mp 119-121 °C; ^1H NMR (300 MHz, DMSO-d_6) δ 3.96 (2H, br. s, C*H*$_2$), 7.21-7.29 (3H, m, ArC*H*), 7.32-7.36 (2H, m, ArC*H*), 7.63 (2H, br. s, N*H*$_2$); ^{13}C NMR (70 MHz, DMSO-d_6) δ 33.2, 82.2, 115.5, 127.0, 128.6 (2C), 128.7 (2C), 135.3, 151.4, 162.3; HRMS (m/ z, +ve, HNESP) $[M+H]^+$ for $C_{11}H_{10}ON_3$ Calc. 200.0824, observed. 200.0824. Elemental. Anal. for $C_{11}H_9N_3O.0.047CH_2Cl_2$; Calc. C, 65.30%; H, 4.51%; N, 20.68%; Observed, C, 65.29%, H, 4.61%, N, 20.68%.

5-Amino-2-isobutyl-oxazole-4-carbonitrile **4**

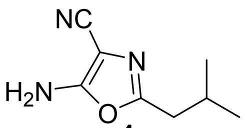

4

Following *General Procedure 3*, 3-methylbutanoyl chloride (5.06 mmol) was treated with aminomalononitrile 4-toluenesulfonate to give *oxazole* **4** (0.32 g, 42% yield) as a white solid. Mp 114-116 °C; ^1H NMR (300 MHz, DMSO-d_6) δ 0.90 (6H, d, *J* = 6.7 Hz, 2xC*H*$_3$), 1.94 (1H, *septet*, *J*= 6.7 Hz, C*H*), 2.43 (2H, d, *J*= 6.9 Hz, C*H*$_2$), 7.58 (2H, s, N*H*$_2$); ^{13}C NMR (70 MHz, DMSO-d_6) δ 22.5 (2C), 27.1, 36.2, 82.6, 116.2, 152.8, 162.6; HRMS (m/ z, +ve, HNESP) $[M+H]^+$ for $C_8H_{12}ON_3$= Calc. 166.0980 , observed. 166.0981. Anal. Calcd for $C_8H_{11}N_3O.0.1(H_2O)$: C, 57.54%; H,6.76%; Found: C, 57.24%, H, 6.85%.

1-(3-Methoxy-2-methylphenyl)thiourea **5**

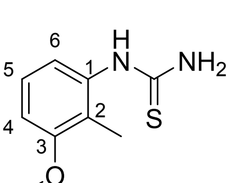

5

Following *General Procedure 1*, 1,1'-thiocarbonyldiimidazole (100 mg, 0.6 mmol, 1.0 eq) was treated with 2-methyl-3-methoxyaniline (78 mg, 0.6 mmol, 1.0 eq) followed by ammonia methanol solution (81 μL, 7 M, 1.0 eq). The crude reaction mixture was filtered and the filtrate collected to afford *thiourea* **5** as a white powder (79 mg, 71%).

Mp 207-209 °C; v_{max} (FTIR) cm^{-1} 3403 (NH_2), 3139 (NH), 1619, 1461, 1111 (C-O-C), 718 (aromatic CH); ^{1}H NMR (400 MHz, DMSO-d_6) δ 9.26 (br. s, 1H, N*H*), 7.16 (t, *J* = 8.0 Hz, 1H, C(5)*H*), 6.88 (d, *J* = 8.3 Hz, 1H, C(4)*H*), 6.78 (d, *J* = 8.0 Hz, 1H, C(6)*H*), 3.79 (s, 3H, OC*H*$_3$), 2.00 (s, 3H, C(2)C*H*$_3$), (N*H*$_2$ not observed); ^{13}C NMR (101 MHz, DMSO-d_6) δ 181.9 (*C*=S), 158.3 (*C*(3)), 138.1 (*C*(1)), 126.8 (*C*(5)), 123.5 (*C*(2)), 120.2 (*C*(6)), 109.4 (*C*(4)), 56.0 (O*C*H$_3$), 11.1 (C(2)*C*H$_3$); *m/z* (ESI$^+$) 197 ([M+H]$^+$, 100%); HRMS (ESI$^+$) $C_9H_{13}N_2OS^+$, ([M+H]$^+$) requires 197.0743, found 197.0742.

1-(2,3-Dimethylphenyl)thiourea **6**

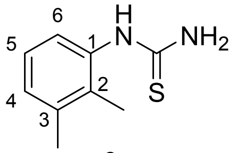

6

Following *General Procedure 1*, 1,1′-thiocarbonyldiimidazole (100 mg, 0.6 mmol, 1.0 eq) was treated with 2,3-dimethylaniline (74 µL, 0.6 mmol, 1.0 eq) followed by ammonia methanol solution (81 µL, 7 M, 1.0 eq) to afford *thiourea* **6** as a white powder (12 mg, 12%). Mp 200-202 °C (Lit.[5] 172-174 °C); v_{max} (FTIR) cm^{-1} 3419 (NH_2), 3154 (NH), 1619, 1526 (C=S), 1293 (-CSNH-), 720 (aromatic CH); ^{1}H NMR (400 MHz, DMSO-d_6) δ 9.27 (br. s, 1H, N*H*), 7.06 - 7.10 (m, 2H, C(4)*H* & C(5)*H*), 6.96 - 7.01 (m, 1H, C(6)*H*), 2.25 (s, 3H, C(3)C*H*$_3$), 2.07 (s, 3H, C(2)C*H*$_3$), (N*H*$_2$ not observed); ^{13}C NMR (101 MHz, DMSO-d_6) δ 181.9 (*C*=S), 137.9 (*C*(3)), 137.2 (*C*(1)), 134.0 (*C*(2)), 128.7 (*C*(4)), 126.1 (*C*(5)), 125.8 (*C*(6)), 20.5 (C(3)*C*H$_3$), 14.4 (C(2)*C*H$_3$); *m/z* (ESI$^+$) 181 ([M+H]$^+$, 100%); HRMS (ESI$^+$) $C_9H_{13}N_2S^+$, ([M+H]$^+$) requires 181.0794, found 181.0790.

1-(2-Methyl-3-(trifluoromethyl)phenyl)thiourea **7**

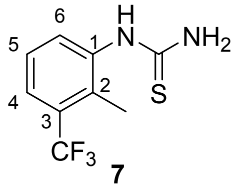

7

Following *General Procedure 1*, 1,1′-thiocarbonyldiimidazole (100 mg, 0.6 mmol, 1.0 eq) was treated with 2-methyl-3-trifluoromethylaniline (99 mg, 0.6 mmol, 1.0 eq) followed by ammonia methanol solution (81 µL, 7 M, 1.0 eq) to afford *thiourea* **7** as a white powder (44 mg, 33%). Mp 133-134 °C; v_{max} (FTIR) cm^{-1} 3157 (NH), 1631, 1314, 1122, 1098; ^{1}H NMR (400 MHz, DMSO-d_6) δ 9.36 (br. s, 1H, N*H*), 7.60 (d, *J* = 7.8 Hz, 1H, C(4)*H*), 7.50 (d, *J* = 7.7 Hz, 1H, C(6)*H*), 7.40 (t, *J* = 7.8 Hz, 1H, C(5)*H*), 2.28 (d, *J* (H-F) = 1.2 Hz, 3H, C*H*$_3$), (N*H*$_2$ not observed); ^{13}C NMR (101 MHz, DMSO-d_6) δ 182.7 (*C*=S), 139.7 (*C*(1)), 134.1 (d, *J* (C-F) = 1.5 Hz, *C*(2)), 133.0 (*C*(6)), 128.8 (d, *J* (C-F) = 29.0 Hz, *C*(3)), 127.0 (*C*(5)), 124.9 (d, *J* (C-F) = 273.0 Hz, *C*F$_3$)), 124.5 (d, *J* (C-F) = 5.9 Hz, *C*(4)), 14.1 (d, *J* (C-F) = 2.2 Hz, *C*H$_3$); ^{19}F NMR (376 MHz, DMSO-d_6) δ -59.37 (C*F*$_3$); *m/z* (ESI$^+$) 235 ([M+H]$^+$, 100%); HRMS (ESI$^+$) $C_9H_{10}F_3N_2S^+$, ([M+H]$^+$) requires 235.0511, found 235.0510.

1-(3-Chloro-2-methylphenyl)thiourea **8**

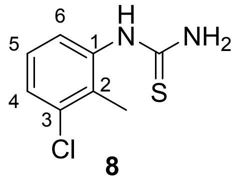

8

Following *General Procedure 1*, 1,1′-thiocarbonyldiimidazole (100 mg, 0.6 mmol, 1.0 eq) was treated with 2-methyl-3-chloroaniline (68 µL, 0.6 mmol, 1.0 eq) followed by ammonia methanol solution (81 µL, 7 M, 1.0 eq) to afford *thiourea* **8** as a white powder (30 mg, 26%). Mp 179-181 °C; v_{max} (FTIR) cm^{-1} 3431 (NH_2), 3147 (NH), 1617, 1523 (C=S), 1294 (-CSNH-), 710 (C-Cl or aromatic CH), 663 (C-Cl or aromatic CH); ^{1}H NMR (400 MHz, DMSO-d_6) δ 9.38 (br. s, 1H, N*H*), 7.32 - 7.37 (m, 1H, C(4)*H*), 7.15 - 7.24 (m, 2H, C(5)*H* & C(6)*H*), 2.21 (s, 3H, C*H*$_3$), (N*H*$_2$ not observed); ^{13}C NMR (101 MHz, DMSO-d_6) δ 182.3 (*C*=S), 139.3 (*C*(1)), 134.3 (*C*(3)), 133.7 (*C*(2)), 127.8 (*C*(4)), 127.6 (*C*(5)), 127.5 (*C*(6)), 15.5 (*C*H$_3$); *m/z* (ESI$^+$) 201 ([M(^{35}Cl)+H]$^+$, 100%), 203 ([M(^{37}Cl)+H]$^+$, 44%); HRMS (ESI$^+$) $C_8H_{10}{}^{35}ClN_2S^+$, ([M+H]$^+$) requires 201.0248, found 201.0243.

1-(2,6-Dimethylphenyl)thiourea **9**

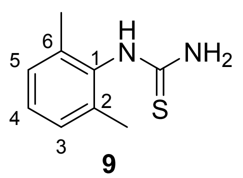

9

Following *General Procedure 1*, 1,1′-thiocarbonyldiimidazole (100 mg, 0.6 mmol, 1.0 eq) was treated with 2,6-dimethylaniline (70 µL, 0.6 mmol, 1.0 eq) followed by ammonia methanol solution (81 µL, 7 M, 1.0 eq) to afford *thiourea* **9** as a white powder (8 mg, 8%). Mp 174-176 °C (Lit.[6] 201-202 °C); v_{max} (FTIR) cm^{-1} 3414 (NH_2), 3143 (NH), 1614, 993, 780 (aromatic CH); ^{1}H NMR (400 MHz, DMSO-d_6) δ 9.16 (br. s, 1H, N*H*), 7.00 - 7.16 (m, 3H, C(3)*H*, C(4)*H* & C(5)*H*), 2.16 (s, 6H, C(2)CH_3 & C(6)CH_3), (NH_2 not observed); ^{13}C NMR (101 MHz, DMSO-d_6) δ 180.8 (*C*=S), 136.6 (*C*(1)), 135.4 (*C*(2) & *C*(6)), 128.7 (*C*(3) & *C*(5)), 127.1 (*C*(4)), 18.1 (*C*H_3); *m/z* (ESI$^+$) 181 ([M+H]$^+$, 100%); HRMS (ESI$^+$) $C_9H_{13}N_2S^+$, ([M+H]$^+$) requires 181.0794, found 181.0786.

1-Methyl-3-(o-tolyl)thiourea **10**

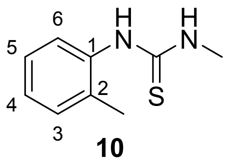

10

Following *General Procedure 1*, 1,1′-thiocarbonyldiimidazole (100 mg, 0.6 mmol, 1.0 eq) was treated with methanamine hydrochloride (63 mg, 0.9 mmol, 1 eq) and triethylamine (143 µL, 1.0 mmol, 1.1 eq) followed by *o*-toluidine (99 µL, 0.9 mmol, 1.0 eq) to afford *thiourea* **10** as a yellow solid (23 mg, 14%). Mp 161-162 °C (Lit.[7] 163-164 °C); v_{max} (FTIR) cm^{-1} 3250 (NH), 3150 (NH), 1517 (C=S), 1249 (-CSNH-), 1032, 743 (aromatic CH); ^{1}H NMR (400 MHz, $CDCl_3$) δ 7.42 (br. s, 1H, C(1)N*H*), 7.30 - 7.35 (m, 1H, C(3)*H*), 7.24 - 7.30 (m, 2H, C(4)*H* & C(5)*H*), 7.18 - 7.24 (m, 1H, C(6)*H*), 5.68 (br. s, 1H, N*H* CH_3), 3.13 (d, *J* = 4.4 Hz, 3H, NHCH_3), 2.29 (s, 3H, C(2)CH_3); ^{13}C NMR (101 MHz, $CDCl_3$) δ 182.1 (*C*=S), 135.9 (*C*(2)), 134.3 (*C*(1)), 131.8 (*C*(3)), 128.6 (*C*(4)), 127.6 (*C*(6)), 127.6 (*C*(5)), 32.2 (NH*C*H_3), 17.7 (C(2)*C*H_3); *m/z* (ESI$^+$) 181 ([M+H]$^+$, 100%); HRMS (ESI$^+$) $C_9H_{13}N_2S^+$, ([M+H]$^+$) requires 181.0794, found 181.0786.

1-(2-Chlorophenyl)-3-methylthiourea **11**

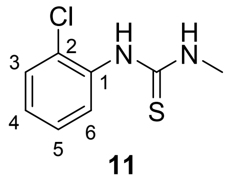

11

Following *General Procedure 1*, 1,1′-thiocarbonyldiimidazole (100 mg, 0.6 mmol, 1.0 eq) was treated with 2-chloroaniline (60 µL, 0.6 mmol, 1.0 eq) and triethylamine (87 µL, 1.0 mmol, 1.1 eq) followed by methanamine hydrochloride (38 mg, 0.6 mmol, 1 eq) to afford *thiourea* **11** as an off-white powder (22 mg, 19%). Mp 158.5-160 °C (Lit.[8] 164-167 °C); v_{max} (FTIR) cm^{-1} 3141 (NH), 1514 (-CSNH-), 1474, 1257 (-CSNH-), 1032, 745 (aromatic CH or C-Cl); ^{1}H NMR (400 MHz, $CDCl_3$) δ 7.59 (br. s, 1H, C(1)N*H*), 7.52 (dd, *J* = 1.2, 8.0 Hz, 1H, C(3)*H*), 7.47 (br. s, 1H, C(6)*H*), 7.36 (dt, *J* = 1.4, 7.7 Hz, 1H, C(5)*H*), 7.27 (dt, *J* = 1.2, 7.7 Hz, 1H, C(4)*H*), 6.03 (br. s, 1H, N*H*CH_3), 3.18 (d, *J* = 4.7 Hz, 3H, CH_3); ^{13}C NMR (101 MHz, $CDCl_3$) δ 181.7 (*C*=S), 133.6 (*C*(1)), 130.9 (*C*(3)), 130.1 (*C*(2)), 128.3 (*C*(4)), 128.1 (*C*(5)), 127.0 (*C*(6)), 32.2 (*C*H_3); *m/z* (ESI$^+$) 201 ([M(^{35}Cl)+H]$^+$, 100%), 203 ([M(^{37}Cl)+H]$^+$, 40%); HRMS (ESI$^+$) $C_8H_{10}{}^{35}ClN_2S^+$, ([M+H]$^+$) requires 201.0248, found 201.0244.

N-(2,6-Dimethoxybenzyl)acetamide **12**

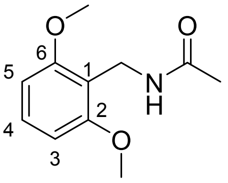

12

Following *General Procedure 2*, acetyl chloride (40 µL, 0.6 mmol, 1.05 eq) was treated with 2,6-dimethoxybenzylamine (90 mg, 0.5 mmol, 1.0 eq) and triethylamine (149 µL, 1.0 mmol, 2.0 eq) to afford *amide* **12** as a yellow solid (93 mg, 79%). Mp 120-122 °C; v_{max} (FTIR) cm^{-1} 3299 (NH), 1634 (C=O), 1594, 1474, 1114 (C-O-C), 773 (C-Cl or aromatic CH), 720 (C-Cl or aromatic CH); ^{1}H NMR (400 MHz, $CDCl_3$) δ 7.15 (t, *J* = 8.4 Hz, 1H, C(4)*H*), 6.49 (d, *J* = 8.4 Hz, 2H, C(3)*H* & C(5)*H*), 5.75 (br. s, 1H, N*H*), 4.46 (d, *J* = 5.3 Hz, 2H, CH_2), 3.77 (s, 6H, C(2)OCH_3 & C(6)OCH_3), 1.87 (s, 3H, C(=O)CH_3); ^{13}C NMR (101 MHz, $CDCl_3$) δ 169.4 (*C*=O),

158.6 (*C*(2) & *C*(6)), 129.0 (*C*(4)), 114.1 (*C*(1)), 103.8 (*C*(3) & *C*(5)), 55.9 (C(2)O*C*H_3 & C(6)O*C*H_3), 32.6 (*C*H_2), 23.5 (*C*H_3); *m/z* (ESI^+) 210 ($[M+H]^+$, 100%); HRMS (ESI^+) $C_{11}H_{15}NO_3Na^+$, ($[M+Na]^+$) requires 232.0944, found 232.0939.

N-(2,6-Dichlorobenzyl)-2-hydroxyacetamide **13**

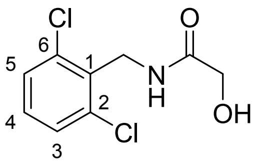

13

Glycolic acid (43 mg, 0.6 mmol, 1.0 eq) was treated with *N,N*-dimthylpyridin-4-amine (7 mg, 0.06 mmol, 0.1 eq) and N-Ethyl-N'-(3-dimethylaminopropyl)carbodiimide hydrochloride (88 mg, 0.5 mmol, 0.8 eq) and added to a stirred solution of 2,6-dichlorobenzylamine (100 mg, 0.6 mmol, 1 eq) and triethylamine (79 µL, 0.6 mmol, 1.0 eq) in DMF (2 mL). The reaction mixture was stirred overnight at room temperature. The reaction mixture was diluted with EtOAc and washed with water (2 x 20 mL) and brine (20 mL). The crude mixture was purified by flash column chromatography (0-10% MeOH in CH_2Cl_2). Pure fractions were collected and the solvent removed *in vacuo* to afford *amide* **13** as a white powder (20 mg, 15%). Mp 112-113 °C; ν_{max} (FTIR) cm^{-1} 3271 (NH), 1647 (C=O), 1099 (C-O), 785 (C-Cl or aromatic CH), 764 (C-Cl or aromatic CH); ^1H NMR (400 MHz, $CDCl_3$) δ 7.33 (d, *J* = 7.8 Hz, 2H, C(3)*H* & C(5)*H*), 7.20 (dd, *J* = 7.4, 8.5 Hz, 1H, C(4)*H*), 6.71 (br. s, 1H, N*H*), 4.81 (d, *J* = 5.6 Hz, 2H, CH_2NH), 4.12 (s, 2H, CH_2OH), 2.76 (br. s., 1H, O*H*); ^{13}C NMR (101 MHz, $CDCl_3$) δ 171.0 (*C*=O), 136.2 (*C*(2) & *C*(6)), 133.1 (*C*(1)), 129.8 (*C*(4)), 128.5 (*C*(3) & *C*(5)), 62.2 (*C*H_2OH), 38.7 (*C*H_2NH); *m/z* (ESI^+) 234 ($[M(^{35}Cl,^{35}Cl)+H]^+$, 100%), 236 ($[M(^{35}Cl,^{37}Cl)+H]^+$, 65%); HRMS (ESI^+) $C_9H_9{}^{35}Cl_2NO_2Na^+$, ($[M+Na]^+$) requires 255.9903, found 255.9907.

N-(2,6-Dichlorobenzyl)-*N*-methylacetamide **14**

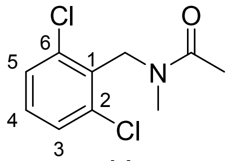

14a

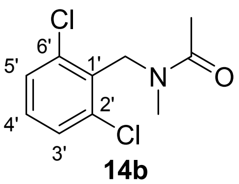

14b

Following *General Procedure 2*, acetyl chloride (40 µL, 0.6 mmol, 1.05 eq) was treated with *N*-methyl-2,6-dichlorobenzylamine hydrochloride (121 mg, 0.5 mmol, 1.0 eq) and triethylamine (224 µL, 1.6 mmol, 3.0 eq) to afford *amide* **14** as a yellow solid (103 mg, 83%). Rotamers **14a** and **14b** were found to be present in a 2:1 ratio by NMR. Mp 71-72 °C; ν_{max} (FTIR) cm^{-1} 1641 (C=O), 1435, 1400, 787 (C-Cl or aromatic CH), 755 (C-Cl or aromatic CH); **14a** ^1H NMR (400 MHz, $CDCl_3$) δ 7.24 - 7.32 (m, 2H, C(3)*H* & C(5)*H*), 7.09 - 7.19 (m, 1H, C(4)*H*), 4.90 (s, 2H, CH_2), 2.69 (s, 3H, NCH_3), 2.06 (s, 3H, C(=O)CH_3); **14a** ^{13}C NMR (101 MHz, $CDCl_3$) δ 170.4 (*C*=O), 136.9 (*C*(2) & *C*(6)), 132.2 (*C*(1)), 129.5 (*C*(4)), 128.6 (*C*(3) & *C*(5)), 44.7 (*C*H_2), 33.7 (N*C*H_3), 21.8 (C(=O)*C*H_3); **14b** ^1H NMR (400 MHz, $CDCl_3$) δ 7.24 - 7.32 (m, 2H, C(3')*H* & C(5')*H*), 7.09 - 7.19 (m, 1H, C(4')*H*), 4.74 (s, 2H, CH_2), 2.61 (s, 3H, NCH_3), 2.26 (s, 3H, C(=O)CH_3); **14b** ^{13}C NMR (101 MHz, $CDCl_3$) δ 170.4 (*C*=O), 136.7 (*C*(2') & *C*(6')), 131.2 (*C*(1')), 130.0 (*C*(4')), 128.9 (*C*(3') & *C*(5')), 48.7 (*C*H_2), 30.4 (N*C*H_3), 22.0 (C(=O)*C*H_3); *m/z* (ESI^+) 232 ($[M(^{35}Cl,^{35}Cl)+H]^+$, 100%), 234 ($[M(^{35}Cl,^{37}Cl)+H]^+$, 75%) ($[M+H]^+$, 100%), 236 ($[M(^{37}Cl,^{37}Cl)+H]^+$, 12%); HRMS (ESI^+) $C_{10}H_{11}{}^{35}Cl_2NONa^+$, ($[M+Na]^+$) requires 254.0110, found 254.0106.

N-(2,6-Dimethoxybenzyl)-2-hydroxyacetamide **15**

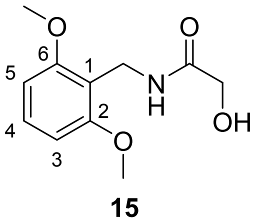

15

Compound **s48** (150 mg, 0.32 mmol, 1 eq) was treated with tetra-*N*-butylammonium fluoride (1M in THF, 0.49 mL, 0.49 mmol, 1.5 eq) in THF (5 mL). The reaction mixture was stirred for 1 h at room temperature. The solvent was removed *in vacuo* and the crude mixture was purified by flash column chromatography (0-10% MeOH in CH_2Cl_2). Pure fractions were collected and the solvent removed *in vacuo* to afford *amide* **15** as a white powder (55 mg, 75%). Mp 126.5-128 °C; ν_{max}

(FTIR) cm^{-1} 3395 (NH), 3277 (OH), 1646 (C=O), 1474 (OH), 1116 (COC); ^{1}H NMR (400 MHz, $CDCl_3$) δ 7.24 (t, *J* = 8.4 Hz, 1H, C(4)*H*), 6.73 (br. s, 1H, N*H*), 6.58 (d, *J* = 8.3 Hz, 2H, C(3)*H* & C(5)*H*), 4.61 (d, *J* = 5.6 Hz, 2H, CH_2NH), 4.06 (d, *J* = 5.3 Hz, 2H, CH_2OH), 3.86 (s, 6H, OCH_3), 2.91 (t, *J* = 5.4 Hz, 1H, O*H*); ^{13}C NMR (101 MHz, $CDCl_3$) δ 170.9 (*C*=O), 158.6 (*C*(2) & *C*(6)), 129.2 (*C*(4)), 113.6 (*C*(1)), 103.8 (*C*(3) & *C*(5)), 62.1 (*C*H_2OH), 55.9 (O*C*H_3), 32.0 (*C*H_2NH); *m/z* (ESI$^+$) 226 ([M+H]$^+$, 100%); HRMS (ESI$^+$) $C_{11}H_{15}NO_4Na^+$, ([M+Na]$^+$) requires 248.0893, found 248.0894.

1-(2,6-Dimethoxybenzyl)pyrrolidin-2-one **16**

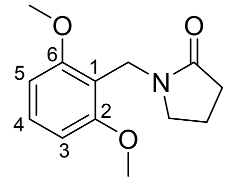

16

2,6-Dimethoxybenzylamine (50 mg, 0.3 mmol, 1.0 eq) was treated with triethylamine (46 µL, 0.33 mmol, 1.1 eq) in acetonitrile (3 mL) before the addition of ethyl 4-bromobutanoate (43 µL, 0.3 mmol, 1.0 eq) and triethylamine (46 µL, 0.33 mmol, 1.1 eq). The reaction mixture was stirred for 3 days at 70 °C. The solvent was removed *in vacuo.* The crude mixture was dissolved in CH_2Cl_2 and washed with NaOH (10%, 5 mL). The resulting solution was purified by flash column chromatography (0-10% MeOH in CH_2Cl_2). Pure fractions were collected and the solvent removed *in vacuo* to afford *lactam* **16** as a red powder (4 mg, 6%). Mp 106-107.5 °C; ν_{max} (FTIR) cm^{-1} 1675 (C=O), 1259, 1114 (COC); ^{1}H NMR (400 MHz, $CDCl_3$) δ 7.16 (t, *J* = 8.4 Hz, 1H, C(4)*H*), 6.48 (d, *J* = 8.3 Hz, 2H, C(3)*H* & C(5)*H*), 4.49 (s, 2H, C(1)CH_2), 3.74 (s, 6H, OCH_3), 3.05 (t, *J* = 7.0 Hz, 2H, NCH_2CH$_2$), 2.29 (t, *J* = 8.1 Hz, 2H, C(=O)CH_2), 1.80 (tt, *J* = 7.0, 8.1 Hz, 2H, NCH$_2$CH_2); ^{13}C NMR (101 MHz, $CDCl_3$) δ 174.3 (*C*=O), 159.2 (*C*(2) & *C*(6)), 129.2 (*C*(4)), 112.0 (*C*(1)), 103.6 (*C*(3) & *C*(5)), 55.8 (O*C*H_3), 46.1 (N*C*H$_2$CH$_2$), 34.7 (C(1)*C*H$_2$), 31.2 (C(=O)*C*H$_2$), 17.7 (NCH$_2$*C*H$_2$); *m/z* (ESI$^+$) 236 ([M+H]$^+$, 100%); HRMS (ESI$^+$) $C_{13}H_{17}NO_3Na^+$, ([M+Na]$^+$) requires 258.1101, found 258.1102.

1-(2,6-Dimethoxybenzyl)piperidin-2-one **17**

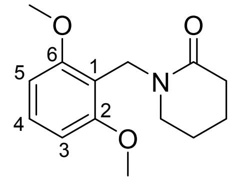

17

2,6-Dimethoxybenzylamine (100 mg, 0.6 mmol, 1.0 eq) was treated with triethylamine (92 µL, 0.66 mmol, 1.1 eq) in acetonitrile (12 mL) before the addition of ethyl 5-bromopentanoate (96 µL, 0.6 mmol, 1.0 eq) and triethylamine (92 µL, 0.66 mmol, 1.1 eq). The reaction mixture was stirred for 3 days at 70 °C. 1,8-Diazabicycloundec-7-ene (99 µL, 0.66 mmol, 1.1 eq) was added to the reaction and stirred for a further 3 days. The solvent was removed *in vacuo.* The crude mixture was dissolved in CH_2Cl_2 and washed with NaOH (10%, 5mL). The resulting solution was purified by flash column chromatography (0-10% MeOH in CH_2Cl_2). Pure fractions were collected and the solvent removed *in vacuo* to afford *lactam* **17** as a colourless oil (27 mg, 18%). ν_{max} (FTIR) cm^{-1} 2936, 1594 (C=O), 1257, 1112 (COC); ^{1}H NMR (400 MHz, $CDCl_3$) δ 7.24 (t, *J* = 8.3 Hz, 1H, C(4)*H*), 6.56 (d, *J* = 8.3 Hz, 2H, C(3)*H* & C(5)*H*), 4.78 (s, 2H, C(1)CH_2), 3.82 (s, 6H, OCH_3), 3.02 (t, *J* = 5.9 Hz, 2H, NCH_2CH$_2$), 2.42 (t, *J* = 6.8 Hz, 2H, C(=O)CH_2CH$_2$), 1.63 - 1.77 (m, 4H, NCH$_2$CH_2 & C(=O)CH$_2$CH_2); ^{13}C NMR (101 MHz, $CDCl_3$) δ 169.2 (*C*=O), 159.5 (*C*(2) & *C*(6)), 129.0 (*C*(4)), 112.6 (*C*(1)), 103.6 (*C*(3) & *C*(5)), 55.8 (O*C*H_3), 45.4 (N*C*H$_2$CH$_2$), 38.1 (C(1)*C*H$_2$), 32.6 (C(=O)*C*H$_2$CH$_2$), 23.2 (NCH$_2$*C*H$_2$), 21.4 (C(=O)CH$_2$*C*H$_2$); *m/z* (ESI$^+$) 250 ([M+H]$^+$, 100%); HRMS (ESI$^+$) $C_{14}H_{19}NO_3Na^+$, ([M+Na]$^+$) requires 272.1257, found 272.1257.

5-Amino-2-isopropyl-oxazole-4-carbonitrile **19**

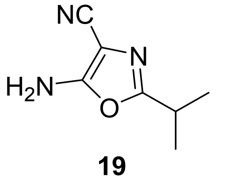

19

Following *General Procedure 3*, isobutyryl chloride (5.06 mmol) was treated with aminomalononitrile 4-toluenesulfonate to give *oxazole* **19** (0.37 g, 54% yield) as a white solid. Mp 134-136 °C; ^{1}H NMR (300 MHz, DMSO-d$_6$) δ 1.18 (6H, d, *J* = 8.0 Hz, 2xCH_3), 2.88

(1H, *septet*, *J* = 8.0 Hz, C*H*), 7.58 (2H, br. s, NH_2); ^{13}C NMR (70 MHz, DMSO-d_6) δ 19.7 (2C), 27.2, 81.8, 115.6, 156.8, 162.0; HRMS (m/ z, +ve, HNESP) $[M+H]^+$ for $C_7H_{10}ON_3$= Calc. 152.0824 observed. 152.0824; Anal. Calcd for $C_7H_9N_3O.0.1H_2O$: C, 54.96%; H, 6.06%; Found: C, 55.19%, H, 6.13%.

5-Amino-2-(4-chloro-benzyl)-oxazole-4-carbonitrile **20**

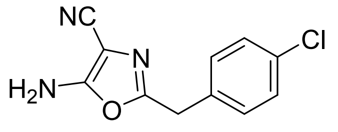

20

Following *General Procedure 3*, 2-(4-chlorophenyl)acetyl chloride (5.06 mmol) was treated with aminomalononitrile 4-toluenesulfonate to give *oxazole* **20** (0.49 g, 46% yield) as a white solid. Mp 129-131 °C; ^{1}H NMR (300 MHz, DMSO-d_6) δ 4.00 (2H, s, CH_2), 7.29 (2H, d, *J* = 8.5 Hz, ArC*H*), 7.40 (2H, d, *J* = 8.5 Hz, ArC*H*), 8.63 (2H, brs, NH_2); ^{13}C NMR (75 MHz, DMSO-d_6) δ 33.0, 82.9, 116.0, 129.1 (2C), 131.2 (2C), 132.3, 134.9, 151.6, 162.9; HRMS (m/ z, +ve, HNESP) $[M+H]^+$ for $C_{11}H_9OClN_3$: Calc. 234.0434; observed. 234.0435; Anal. Calcd for $C_{11}H_8N_3OCl.0.1H_2O$: C, 56.11%; H, 3.51%; N, 17.85%. Found: C, 55.64%, H, 3.57%, N, 18.01%.

1-Ethyl-3-(o-tolyl)thiourea **s1**

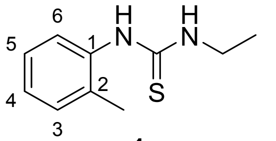

s1

Following *General Procedure 1*, 1,1′-thiocarbonyldiimidazole (100 mg, 0.6 mmol, 1.0 eq) was treated with *o*-toluidine (60 µL, 0.6 mmol, 1.0 eq) followed by ethanamine tetrahydrofuran solution (284 µL, 2 M, 1.0 eq) to afford *thiourea* **s1** as a white powder (82 mg, 74%). Mp 85-87 °C (Lit.[9] 83 °C); ν_{max} (FTIR) cm^{-1} 3147 (NH), 1536 (C=S), 1514, 1245 (-CSNH-), 737 (aromatic CH); ^{1}H NMR (400 MHz, $CDCl_3$) δ 7.55 (br. s, 1H, C(1)N*H*), 7.31 - 7.36 (m, 1H, C(3)*H*), 7.25 - 7.30 (m, 2H, C(4)*H* & C(5)*H*), 7.19 - 7.24 (m, 1H, C(6)*H*), 5.62 (br. s., 1H, N*H*CH_2), 3.61 - 3.71 (m, 2H, CH_2), 2.30 (s, 3H, C(2)CH_3), 1.17 (t, *J* = 7.2 Hz, 3H, CH_2CH_3); ^{13}C NMR (101 MHz, $CDCl_3$) δ 180.8 (*C*=S), 135.9 (*C*(2)), 134.3 (*C*(1)), 131.8 (*C*(3)), 128.5 (*C*(4)), 127.6 (*C*(5 or 6)), 127.5 (*C*(5 or 6)), 40.3 (*C*H_2), 17.8 (C(2)CH_3), 14.4 (CH_2*C*H_3); *m/z* (ESI$^+$) 195 ($[M+H]^+$, 100%); HRMS (ESI$^+$) $C_{10}H_{15}N_2S^+$, ($[M+H]^+$) requires 195.0950, found 195.0944.

1-Isopropyl-3-(o-tolyl)thiourea **s2**

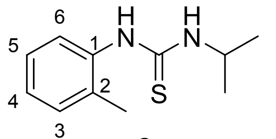

s2

Following *General Procedure 1*, 1,1′-thiocarbonyldiimidazole (100 mg, 0.6 mmol, 1.0 eq) was treated with *o*-toluidine (60 µL, 0.6 mmol, 1.0 eq) followed by isopropylamine (49 µL, 0.6 mmol, 1.0 eq) to afford *thiourea* **s2** as a white powder (92 mg, 78%). Mp 83-84 °C; ν_{max} (FTIR) cm^{-1} 3234 (NH), 1533 (C=S), 1323, 721 (aromatic CH); ^{1}H NMR (400 MHz, $CDCl_3$) δ 7.42 (br. s, 1H, C(1)N*H*), 7.32 - 7.36 (m, 1H, C(3)*H*), 7.25 - 7.31 (m, 2H, C(4)*H* & C(5)*H*), 7.15 - 7.23 (m, 1H, C(6)*H*), 5.40 (br. d, *J* = 5.1 Hz, 1H, N*H*CH), 4.61 (dd, *J* = 6.6, 14.6 Hz, 1H, C*H*), 2.30 (s, 3H, C(2)CH_3), 1.18 (d, *J* = 6.5 Hz, 6H, CH(CH_3)$_2$); ^{13}C NMR (101 MHz, $CDCl_3$) δ 179.7 (*C*=S), 135.7 (*C*(2)), 134.2 (*C*(1)), 131.8 (*C*(3)), 128.5 (*C*(4)), 127.6 (*C*(5)), 127.4 (*C*(6)), 47.4 (*C*H), 22.4 (CH(*C*H_3)$_2$), 17.7 (C(2)*C*H_3); *m/z* (ESI$^+$) 209 ($[M+H]^+$, 100%); HRMS (ESI$^+$) $C_{11}H_{17}N_2S^+$, ($[M+H]^+$) requires 209.1107, found 209.1100.

1-(2-Hydroxyethyl)-3-(o-tolyl)thiourea **s3**

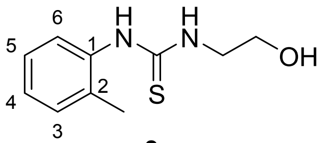

s3

Following *General Procedure 1*, 1,1′-thiocarbonyldiimidazole (166 mg, 0.9 mmol, 1.0 eq) was treated with *o*-toluidine (99 µL, 0.9 mmol, 1.0 eq) followed by 2-aminoethanol (56 µL, 0.9 mmol, 1.0 eq) to afford *thiourea* **s3** as white

crystals (136 mg, 69%). Mp 1 23-124 °C; ν_{max} (FTIR) cm^{-1} 3376 (OH), 3270 (NH), 3178 (NH), 1541, 1509 (C=S), 1060 (C-O), 740 (aromatic CH); ^{1}H NMR (400 MHz, DMSO-d_6) δ 9.13 (br. s, 1H, C(1)N*H*), 7.39 (br. s, 1H, N*H*CH_2), 7.21 - 7.28 (m, 2H, C(3)*H* & C(6)*H*), 7.09 - 7.21 (m, 2H, C(4)*H* & C(5)*H*), 4.74 (br. s, 1H, O*H*), 3.51 (br. s., 4H, CH_2CH_2OH), 2.17 (s, 3H, CH_3); ^{13}C NMR (101 MHz, DMSO-d_6) δ 181.7 (*C*(8)), 137.6 (*C*(1) or *C*(2)), 134.9 (*C*(1) or *C*(2)), 130.9 (*C*(3)), 128.1 (*C*(6)), 126.8 (*C*(4) or *C*(5)), 126.7 (*C*(4) or *C*(5)), 59.9 (CH_2CH_2OH or CH_2CH_2OH), 47.1 (CH_2CH_2OH or CH_2CH_2OH), 18.1 (CH_3); *m/z* (ESI^+) 211 ($[M+H]^+$, 100%); HRMS (ESI^+) $C_{10}H_{15}N_2OS^+$, ($[M+H]^+$) requires 211.0900, found 211.0896.

1,1-Dimethyl-3-(o-tolyl)thiourea **s4**

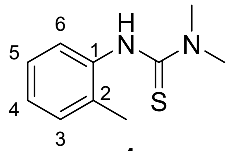

s4

Following *General Procedure 1*, 1,1′-thiocarbonyldiimidazole (100 mg, 0.6 mmol, 1.0 eq) was treated with *o*-toluidine (60 µL, 0.6 mmol, 1.0 eq) followed by dimethylamine hydrochloride (46 mg, 0.6 mmol, 1.0 eq) and triethylamine (87 µL, 0.6 mmol, 1.1 eq) to afford *thiourea* **s4** as a white powder (72 mg, 65%). Mp 137-138 °C (Lit.[10] 137-138.5 °C); ν_{max} (FTIR) cm^{-1} 3250 (NH), 1524 (C=S), 1332, 724 (724 (aromatic CH); ^{1}H NMR (400 MHz, DMSO-d_6) δ 8.79 (br. s, 1H, N*H*), 7.18 - 7.23 (m, 1H, C(3)*H*), 7.11 - 7.18 (m, 2H, C(4)*H* & C(5)*H*), 7.02 - 7.07 (m, 1H, C(6)*H*), 3.27 (s, 6H, $N(CH_3)_2$), 2.17 (s, 3H, $C(2)CH_3$); ^{13}C NMR (101 MHz, DMSO-d_6) δ 181.9 (*C*=S), 140.3 (*C*(1)), 136.2 (*C*(2)), 130.4 (*C*(3)), 129.5 (*C*(6)), 126.7 (*C*(4)), 126.3 (*C*(5)), 41.2 ($N(CH_3)_2$), 18.4 ($C(2)CH_3$); *m/z* (ESI^+) 195 ($[M+H]^+$, 100%); HRMS (ESI^+) $C_{10}H_{15}N_2S^+$, ($[M+H]^+$) requires 195.0950, found 195.0944.

1-(2-Chlorophenyl)thiourea **s5**

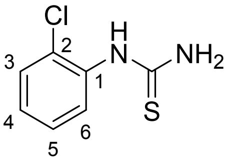

s5

Following *General Procedure 1*, 1,1′-thiocarbonyldiimidazole (100 mg, 0.6 mmol, 1.0 eq) was treated with 2-chloroaniline (60 µL, 0.6 mmol, 1.0 eq) followed by ammonia methanol solution (81 µL, 7 M, 1.0 eq) to afford *thiourea* **s5** as a white powder (31 mg, 29%). Mp 114.5-116 °C (Lit.[11] 141-143 °C); ν_{max} (FTIR) cm^{-1} 3413 (NH_2), 3122 (NH), 1614, 1511 (C=S), 1293 (-CSNH-), 723 (aromatic CH); ^{1}H NMR (400 MHz, DMSO-d_6) δ 9.31 (s, 1H, N*H*), 7.65 (dd, *J* = 1.5, 8.0 Hz, 1H, C(6)*H*), 7.49 (dd, *J* = 1.4, 8.0 Hz, 1H, C(3)*H*), 7.33 (dt, *J* = 1.5, 7.7 Hz, 1H, C(5)*H*), 7.24 (dt, *J* = 1.4, 7.5 Hz, 1H, C(4)*H*), (NH_2 not observed); ^{13}C NMR (101 MHz, DMSO-d_6) δ 182.5 (*C*=S), 136.5 (*C*(1)), 129.9 (*C*(3)), 129.7 (*C*(6)), 129.6 (*C*(2)), 127.7 (*C*(4)), 127.6 (*C*(5)); *m/z* (ESI^+) 187 ($[M(^{35}Cl)+H]^+$, 100%), 189 ($[M(^{37}Cl)+H]^+$, 33%); HRMS (ESI^+) $C_7H_8{}^{35}ClN_2S^+$, ($[M+H]^+$) requires 187.0091, found 187.0087.

1-(2-Methoxyphenyl)thiourea **s6**

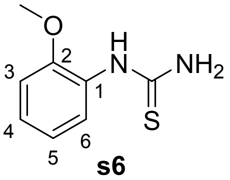

s6

Following *General Procedure 1*, 1,1′-thiocarbonyldiimidazole (100 mg, 0.6 mmol, 1.0 eq) was treated with 2-methoxyaniline (64 µL, 0.6 mmol, 1.0 eq) followed by ammonia methanol solution (81 µL, 7 M, 1.0 eq) to afford *thiourea* **s6** as a white powder (20 mg, 19%). Mp 155.5-157 °C (Lit.[11] 156 °C); ν_{max} (FTIR) cm^{-1} 3431 (NH_2), 3327, 3159 (NH), 1596, 1526 (C=S), 1253 (-CSNH-), 1011 (COC), 765 (aromatic CH); ^{1}H NMR (400 MHz, DMSO-d_6) δ 9.02 (br. s, 1H, C(1)N*H*), 7.82 (br. d, *J* = 7.5 Hz, 1H, C(6)*H*), 7.10 - 7.17 (m, 1H, C(4)*H*), 7.04 (dd, *J* = 1.2, 8.2 Hz, 1H, C(3)*H*), 6.91 (dt, *J* = 1.3, 7.6 Hz, 1H, C(5)*H*), 3.82 (s, 3H, OCH_3), (NH_2 not observed); ^{13}C NMR (101 MHz, DMSO-d_6) δ 181.8, (*C*=S), 152.2 (*C*(2)), 128.0 (*C*(1)), 126.2 (*C*(4)), 126.0 (*C*(6)), 120.3 (*C*(5)), 111.9 (*C*(3)), 56.0 (OCH_3); *m/z* (ESI^+) 183 ($[M+H]^+$, 100%); HRMS (ESI^+) $C_8H_{11}N_2OS^+$, ($[M+H]^+$) requires 183.0587, found 183.0591.

1-(2,4-Dimethylphenyl)thiourea **s8**

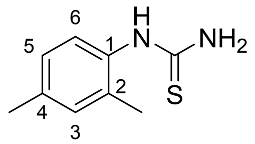

s8

Following *General Procedure 1*, 1,1′-thiocarbonyldiimidazole (100 mg, 0.6 mmol, 1.0 eq) was treated with 2,4-dimethylaniline (70 µL, 0.6 mmol, 1.0 eq) followed by ammonia methanol solution (81 µL, 7 M, 1.0 eq) to afford *thiourea* **s8** as a white powder (33 mg, 32%). Mp 185-187 °C (Lit.[12] 188-190 °C); ν_{max} (FTIR) cm^{-1} 3421 (NH_2), 3156 (NH), 1614, 1528 (C=S), 1298 (-CSNH-); ^{1}H NMR (400 MHz, DMSO-d_6) δ 9.14 (br. s, 1H, N*H*), 7.02 - 7.08 (m, 2H, C(3 or 5)*H* & C(6)*H*), 6.97 - 7.02 (m, 1H, C(3 or 5)*H*), 2.26 (s, 3H, C(2)CH_3 or C(4)CH_3), 2.14 (s, 3H, C(2)CH_3 or C(4)CH_3), (NH_2 not observed); ^{13}C NMR (101 MHz, DMSO-d_6) δ 182.1 (*C*=S), 136.3 (*C*(2 or 4)), 134.9 (*C*(1)), 134.8 (*C*(2 or 4)), 131.6 (*C*(3 or 5)), 127.9 (*C*(6)), 127.4 (*C*(3 or 5)), 21.0 (C(2)*C*H_3 or C(4)*C*H_3), 18.0 (C(2)*C*H_3 or C(4)*C*H_3); *m/z* (ESI$^+$) 181 ([M+H]$^+$, 100%); HRMS (ESI$^+$) $C_9H_{13}N_2S^+$, ([M+H]$^+$) requires 181.0794, found 181.0790.

1-(2,5-Dimethylphenyl)thiourea **s9**

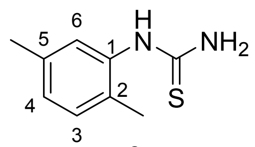

s9

Following *General Procedure 1*, 1,1′-thiocarbonyldiimidazole (100 mg, 0.6 mmol, 1.0 eq) was treated with 2,5-dimethylaniline (71 µL, 0.6 mmol, 1.0 eq) followed by ammonia methanol solution (81 µL, 7 M, 1.0 eq) to afford *thiourea* **s9** as a white powder (45 mg, 44%). Mp 143-144 °C (Lit.[12] 145 °C); ν_{max} (FTIR) cm^{-1} 3424 (NH_2), 3152 (NH), 1617, 1525 (C=S), 1295 (-CSNH-), 818 (aromatic CH); ^{1}H NMR (400 MHz, DMSO-d_6) δ 9.18 (br. s, 1H, N*H*), 7.12 (d, *J* = 7.5 Hz, 1H, C(3)*H*), 7.00 (s, 2H, C(3)*H* & C(6)*H*), 2.26 (s, 3H, C(5)CH_3), 2.13 (s, 3H, C(2)CH_3), (NH_2 not observed); ^{13}C NMR (101 MHz, DMSO-d_6) δ 181.9 (*C*=S), 137.2 (*C*(1)), 135.9 (*C*(5)), 131.7 (*C*(2)), 130.8 (*C*(3)), 128.4 (*C*(4 or 6)), 127.8 (*C*(4 or 6)), 20.9 (C(5)*C*H_3), 17.6 (C(2)*C*H_3); *m/z* (ESI$^+$) 181 ([M+H]$^+$, 100%); HRMS (ESI$^+$) $C_9H_{13}N_2S^+$, ([M+H]$^+$) requires 181.0794, found 181.0787.

1-(3-Fluoro-2-methylphenyl)thiourea **s10**

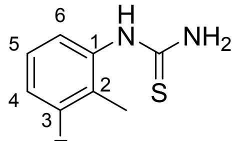

s10

Following *General Procedure 1*, 1,1′-thiocarbonyldiimidazole (100 mg, 0.6 mmol, 1.0 eq) was treated with 2-methyl-3-fluoroaniline (65 µL, 0.6 mmol, 1.0 eq) followed by ammonia methanol solution (81 µL, 7 M, 1.0 eq) to afford *thiourea* **s10** as a white powder (47 mg, 45%). Mp 169-170 °C; ν_{max} (FTIR) cm^{-1} 3444 (NH_2), 3128 (NH), 2923, 1619, 1513 (C=S), 1462, 713 (aromatic CH); ^{1}H NMR (400 MHz, DMSO-d_6) δ 9.34 (br. s, 1H, N*H*), 7.18 - 7.25 (m, 1H, C(5)*H*), 7.03 - 7.11 (m, 2H, C(4)*H* & C(6)*H*), 2.09 (d, *J* (H-F) = 2.1 Hz, 3H, CH_3), (NH_2 not observed); ^{13}C NMR (101 MHz, DMSO-d_6) δ 182.3 (*C*=S), 161.4 (d, *J* (C-F) = 255.0 Hz, *C*(3)), 139.4 (d, *J* (C-F) = 6.6 Hz, *C*(1)), 127.3 (d, *J* (C-F) = 10.3 Hz, *C*(5)), 124.1 (d, *J* (C-F) = 2.9 Hz, *C*(6)), 122.5 (d, *J* (C-F) = 17.6 Hz, *C*(2)), 113.6 (d, *J* (C-F) = 22.0 Hz, *C*(4)), 10.2 (d, *J* (C-F) = 4.4 Hz, *C*H_3); ^{19}F NMR (376 MHz, DMSO-d_6) δ -115.49 (C(4)*F*); *m/z* (ESI$^+$) 185 ([M+H]$^+$, 100%); HRMS (ESI$^+$) $C_8H_{10}FN_2S^+$, ([M+H]$^+$) requires 185.0543, found 185.0539.

1-(3-Chloro-2,6-dimethylphenyl)thiourea **s13**

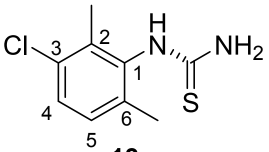

s13a

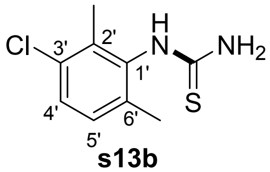

s13b

Following *General Procedure 1*, 1,1′-thiocarbonyldiimidazole (100 mg, 0.6 mmol, 1.0 eq) was treated with 3-chloro-2,6-dimethylanilne (88 mg, 0.6 mmol, 1.0 eq) followed by ammonia methanol solution (81 µL, 7 M, 1.0 eq) to afford *thiourea* **s13** as a white powder (44 mg, 36%). Rotamers s13a and s13b were found to be present in a 3:2 ratio by NMR. Absolute stereochemistry was not determined. Mp 156-157.5 °C; v_{max} (FTIR) cm^{-1} 3417 (NH_2), 3128 (NH), 1614, 1517 (C=S), 1286 (-CSNH-), 1070, 809 (aromatic CH); ^1H NMR (400 MHz, DMSO-d_6, 343 K) δ 9.00 (br. s, 1H, N*H*), 7.27 (d, *J* = 8.2 Hz, 1H, C(4)*H*), 7.11 (d, *J* = 8.2 Hz, 1H, C(5)*H*), 7.05 (br. s, 2H, NH_2), 2.23 (s, 3H, C(2)CH_3), 2.18 (s, 3H, C(6)H_3); **s13a** ^1H NMR (400 MHz, DMSO-d_6) δ 9.29 (br. s, 1H, N*H*), 7.31 (d, *J* = 8.2 Hz, 1H, C(4)*H*), 7.14 (d, *J* = 8.2 Hz, 1H, C(5)*H*), 2.19 (s, 3H, C(2)CH_3), 2.14 (s, 3H, C(6)CH_3), (NH_2 not observed); **s13b** ^1H NMR (400 MHz, DMSO-d_6) δ 8.98 (br. s, 1H, N*H*), 7.26 (d, *J* = 8.1 Hz, 1H, C(4')*H*), 7.09 (d, *J* = 8.1 Hz, 1H, C(5')*H*), 2.19 (s, 3H, C(2')CH_3), 2.14 (s, 3H, C(6')CH_3), (NH_2 not observed); **s13a** ^{13}C NMR (101 MHz, DMSO-d_6) δ 181.0 (*C*=S), 136.9 (*C*(1)), 135.9 (*C*(2)), 134.8 (*C*(6)), 131.7 (*C*(3)), 129.4 (*C*(5)), 128.5 (*C*(4)), 18.0 (C(6)CH_3), 15.7 (C(2)*C*H_3); **s13b** ^{13}C NMR (101 MHz, DMSO-d_6) δ 183.7 (*C*=S), 139.4 (*C*(1')), 136.4 (*C*(2')), 135.2 (*C*(6')), 131.0 (*C*(3')), 128.7 (*C*(5')), 127.6 (*C*(4')), 18.3 (C(6')CH_3), 15.8 (C(2')*C*H_3); *m/z* (ESI^+) 215 ([M(^{35}Cl)+H]$^+$, 100%), 217 ([M(^{37}Cl)+H]$^+$, 45%); HRMS (ESI^+) $C_9H_{12}{}^{35}ClN_2S^+$, ([M+H]$^+$) requires 215.0404, found 215.0411.

1-(2,3-Dimethylphenyl)-3-methylthiourea **s14**

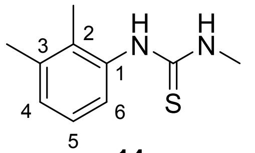

s14

Following *General Procedure 1*, 1,1′-thiocarbonyldiimidazole (100 mg, 0.6 mmol, 1.0 eq) was treated with 2,3-dimethylaniline (74 µL, 0.6 mmol, 1.0 eq) followed by methanamine hydrochloride (38 mg, 0.6 mmol, 1.0 eq) and triethylamine (87 µL, 0.62 mmol, 1.1 eq) to afford *thiourea* **s14** as a white powder (37 mg, 33%). Mp 173-174 °C (Lit.[13] 177-178 °C); v_{max} (FTIR) cm^{-1} 3178 (NH), 1514 (-CSNH-), 1259 (C=S), 1222 (-NC(=S)N-), 752 (aromatic CH); ^1H NMR (400 MHz, $CDCl_3$) δ 7.45 (br. s, 1H, C(1)N*H*), 7.06 - 7.14 (m, 2H, C(4)*H* & C(5)*H*), 6.98 (dd, *J* = 2.1, 6.9 Hz, 1H, C(6)*H*), 5.53 (br. s, 1H, N*H*Me), 3.04 (d, *J* = 4.8 Hz, 3H, NHCH_3), 2.25 (s, 3H, C(3)CH_3), 2.11 (s, 3H, C(2)CH_3); ^{13}C NMR (101 MHz, $CDCl_3$) δ 182.1 (*C*=S), 139.3 (*C*(3)), 134.8 (*C*(2)), 134.1 (*C*(1)), 130.2 (*C*(4)), 126.9 (*C*(5)), 125.4 (*C*(6)), 32.1 (NH*C*H_3), 20.4 (C(3)*C*H_3), 14.1 (C(2)*C*H_3); *m/z* (ESI^+) 195 ([M+H]$^+$, 100%); HRMS (ESI^+) $C_{10}H_{15}N_2S^+$, ([M+H]$^+$) requires 195.0950, found 195.0955.

1-(3-Chloro-2-methylphenyl)-3-methylthiourea **s15**

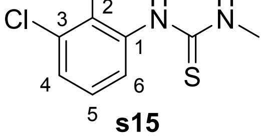

s15

Following *General Procedure 1*, 1,1′-thiocarbonyldiimidazole (100 mg, 0.6 mmol, 1.0 eq) was treated with 3-chloro-2-methylaniline (68 µL, 0.6 mmol, 1.0 eq) followed by methanamine hydrochloride (38 mg, 0.6 mmol, 1.0 eq) and triethylamine (87 µL, 0.62 mmol, 1.1 eq) to afford *thiourea* **s15** as a white powder (53 mg, 44%). Mp 157-158.5 °C; v_{max} (FTIR) cm^{-1} 3148 (NH), 1498 (-CSNH-), 1256 (C=S), 1221 (-NC(=S)N-), 798 (aromatic CH or C-Cl), 759 (aromatic CH or C-Cl); ^1H NMR (400 MHz, $CDCl_3$) δ 7.62 (br. s, 1H, C(1)N*H*), 7.42 (d, *J* = 8.0 Hz, 1H, C(6)*H*), 7.23 (t, *J* = 7.9 Hz, 1H, C(5)*H*), 7.17 (d, *J* = 7.6 Hz, 1H, C(4)*H*), 5.62 (br. s, 1H, N*H*Me), 3.14 (d, *J* = 4.8 Hz, 3H, NHCH_3), 2.35 (s, 3H, C(2)CH_3); ^{13}C NMR (101 MHz, $CDCl_3$) δ 182.1 (*C*=S), 136.4 (*C*(1)), 135.6 (*C*(3)), 134.8 (*C*(2)), 129.6 (*C*(6)), 127.8 (*C*(5)), 126.3 (*C*(4)), 32.3 (NH*C*H_3), 15.2 (C(2)*C*H_3); *m/z* (ESI^+) 215 ([M(^{35}Cl)+H]$^+$, 100%), 217 ([M(^{37}Cl)+H]$^+$, 40%); HRMS (ESI^+) $C_9H_{12}{}^{35}ClN_2S^+$, ([M+H]$^+$) requires 215.0404, found 215.0408.

1-(2-Methoxyphenyl)-3-methylthiourea **s16**

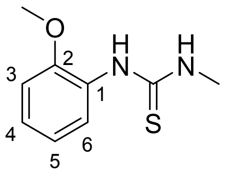

s16

Following *General Procedure 1*, 1,1′-thiocarbonyldiimidazole (100 mg, 0.6 mmol, 1.0 eq) was treated with 2-methoxyaniline (64 µL, 0.6 mmol, 1.0 eq) followed by methanamine hydrochloride (38 mg, 0.6 mmol, 1.0 eq) and triethylamine (87 µL, 0.62 mmol, 1.1 eq) to afford *thiourea* **s16** as a white powder (52 mg, 47%). Mp 141.5-142.5 °C (Lit.[8] 137-139 °C); ν_{max} (FTIR) cm^{-1} 3168 (NH), 1499 (-CSNH-), 1245 (C=S), 1025 (COC), 752 (aromatic CH), 759 (aromatic CH or C-Cl); ^{1}H NMR (400 MHz, $CDCl_3$) δ 7.55 (br. s, 1H, C(1)N*H*), 7.32 (br. s, 1H, C(6)*H*), 7.26 (dt, *J* = 1.7, 7.9 Hz, 1H, C(4)*H*), 6.96 - 7.04 (m, 2H, C(3)*H* & C(5)*H*), 6.18 (br. s, 1H, N*H*CH_3), 3.87 (s, 3H, OCH_3), 3.17 (d, *J* = 4.7 Hz, 3H, NHCH_3); ^{13}C NMR (101 MHz, $CDCl_3$) δ 181.7 (*C*=S), 152.7 (*C*(2)), 127.7 (*C*(4)), 125.3 (*C*(1)), 125.0 (*C*(6)), 121.0 (*C*(5)), 112.1 (*C*(3)), 55.7 (OCH_3), 32.2 (NHCH_3); *m/z* (ESI^+) 197 ($[M+H]^+$, 100%); HRMS (ESI^+) $C_9H_{13}N_2OS^+$, ($[M+H]^+$) requires 197.0743, found 197.0754.

1-(2-Methoxy-6-methylphenyl)-3-methylthiourea **s18**

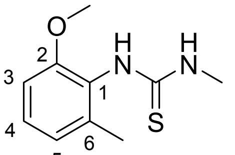

s18

Following *General Procedure 1*, 1,1′-thiocarbonyldiimidazole (100 mg, 0.6 mmol, 1.0 eq) was treated with 2-methoxy-6-methylaniline (78 mg, 0.6 mmol, 1.0 eq) followed by methanamine hydrochloride (38 mg, 0.6 mmol, 1.0 eq) and triethylamine (87 µL, 0.62 mmol, 1.1 eq) to afford *thiourea* **s18** as an off-white powder (57 mg, 48%). Mp 138.5-139.5 °C; ν_{max} (FTIR) cm^{-1} 3361 (NH), 1473 (-CSNH-), 1280 (C=S), 1242 (-NC(=S)N-), 1082 (COC), 774 (aromatic CH); ^{1}H NMR (400 MHz, $CDCl_3$) δ 7.17 (t, *J* = 8.0 Hz, 1H, C(4)*H*), 7.07 (br. s, 1H, C(1)N*H*), 6.81 (d, *J* = 7.7 Hz, 1H, C(5)*H*), 6.75 (d, *J* = 8.2 Hz, 1H, C(3)*H*), 5.49 (br. s, 1H, N*H*Me), 3.74 (s, 3H, OCH_3), 3.04 (d, *J* = 4.8 Hz, 3H, NHCH_3), 2.19 (s, 3H, C(6)CH_3); ^{13}C NMR (101 MHz, $CDCl_3$) δ 182.4 (*C*=S), 155.5 (*C*(2)), 138.1 (*C*(6)), 129.3 (*C*(4)), 122.9 (*C*(5)), 109.5 (*C*(3)), 55.8 (OCH_3), 32.2 (NHCH_3), 17.8 (C(6)CH_3), (*C*(1) not observed); *m/z* (ESI^+) 211 ($[M+H]^+$, 100%); HRMS (ESI^+) $C_{10}H_{15}N_2OS^+$, ($[M+H]^+$) requires 211.0900, found 211.0901.

1-(3-Chloro-2,6-dimethylphenyl)-3-methylthiourea **s19**

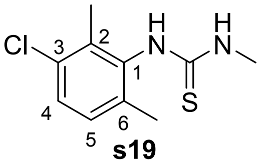

s19

Following *General Procedure 1*, 1,1′-thiocarbonyldiimidazole (100 mg, 0.6 mmol, 1.0 eq) was treated with 3-chloro-2,6-dimethylaniline (88 mg, 0.6 mmol, 1.0 eq) followed by methanamine hydrochloride (38 mg, 0.6 mmol, 1.0 eq) and triethylamine (87 µL, 0.62 mmol, 1.1 eq) to afford *thiourea* **s19** as a white powder (16 mg, 12%). Mp 162-163 °C; ν_{max} (FTIR) cm^{-1} 3165 (NH), 1507 (-CSNH-), 1257 (C=S), 1057, 824 (aromatic CH), 715 (C-Cl); ^{1}H NMR (400 MHz, $CDCl_3$) δ 7.57 (br. s, 1H, C(1)N*H*), 7.34 (d, *J* = 8.3 Hz, 1H, C(4)*H*), 7.12 (d, *J* = 8.3 Hz, 1H, C(5)*H*), 5.36 (br. s, 1H, N*H*Me), 3.12 (d, *J* = 4.7 Hz, 3H, NHCH_3), 2.33 (s, 3H, C(2)CH_3), 2.26 (s, 3H, C(6)CH_3); ^{13}C NMR (101 MHz, $CDCl_3$) δ 181.9 (*C*=S), 136.1 (*C*(2)), 135.8 (*C*(6)), 133.2 (*C*(3)), 129.7 (*C*(4)), 129.3 (*C*(5)), 32.2 (NHCH_3), 18.1 (C(6)CH_3), 15.5 (C(2)CH_3); *m/z* (ESI^+) 229 ($[M(^{35}Cl)+H]^+$, 100%), 231 ($[M(^{37}Cl)+H]^+$, 35%); HRMS (ESI^+) $C_{10}H_{15}{}^{35}ClN_2S^+$, ($[M+H]^+$) requires 229.0561, found 229.0560.

1-(2,3-Dimethylphenyl)urea **s22**

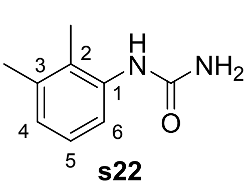

s22

Following *General Procedure 4*, 2,3-dimethylaniline (96 µL, 0.7 mmol, 1.0 eq) was treated with potassium cyanate and acetic acid to afford *urea* **s22** as a pink powder (59 mg, 49%). Mp 194-195.5 °C (Lit.[14] 219-221 °C); ν_{max} (FTIR) cm^{-1} 3436 (NH_2), 3307 (NH), 1646 (C=O), 1544, 1349, 763 (aromatic CH); ^{1}H NMR (400 MHz, DMSO-d_6) δ 7.70

(s, 1H, N*H*), 7.48 (d, *J* = 8.1 Hz, 1H, C(6)*H*), 6.97 (t, *J* = 7.8 Hz, 1H, C(5)*H*), 6.83 (d, *J* = 7.3 Hz, 1H, C(4)*H*), 5.90 (s, 2H, NH_2), 2.23 (s, 3H, C(3)CH_3), 2.08 (s, 3H, C(2)CH_3); ^{13}C NMR (101 MHz, DMSO-d$_6$) δ 156.8 (*C*=O), 138.2 (*C*(1)), 136.8 (*C*(3)), 127.5 (*C*(2)), 125.5 (*C*(5)), 124.8 (*C*(4)), 120.8 (*C*(6)), 20.8 (C(3)*C*H$_3$), 14.0 (C(2)*C*H$_3$); *m/z* (ESI$^+$) 165 ([M+H]$^+$, 100%); HRMS (ESI$^+$) $C_9H_{12}N_2ONa^+$, ([M+Na]$^+$) requires 187.0842, found 187.0847.

1-(3-Chloro-2-methylphenyl)urea **s23**

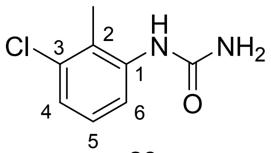
s23

Following *General Procedure 4*, 3-chloro-2-methylaniline (60 mg, 0.7 mmol, 1.0 eq) was treated with potassium cyanate and acetic acid to afford *urea* **s23** as a white powder (71 mg, 52%). Mp 269-272 °C; ν_{max} (FTIR) cm^{-1} 3424 (NH$_2$), 3307 (NH), 1647 (C=O), 1545, 1349, 777 (aromatic CH); ^{1}H NMR (400 MHz, DMSO-d$_6$) δ 7.90 (br. s, 1H, C(1)N*H*), 7.72 (dd, *J* = 1.6, 7.7 Hz, 1H, C(6)*H*), 7.05 - 7.14 (m, 2H, C(4)*H* & C(5)*H*), 6.08 (br. s, 2H, NH_2), 2.24 (s, 3H, CH_3); ^{13}C NMR (101 MHz, DMSO-d$_6$) δ 156.5 (*C*=O), 140.2 (*C*(1) or *C*(3)), 133.8 (*C*(1) or *C*(3)), 127.2 (*C*(5)), 126.2 (*C*(2)), 123.4 (*C*(4)), 121.0 (*C*(6)), 15.1 (*C*H$_3$); *m/z* (ESI$^+$) 185 ([M(^{35}Cl)+H]$^+$, 100%), 187 ([M(^{37}Cl)+H]$^+$, 30%); HRMS (ESI$^+$) $C_8H_{10}{}^{35}ClN_2O^+$, ([M+H]$^+$) requires 185.0476, found 185.0481.

1-(2-Chlorophenyl)urea **s24**

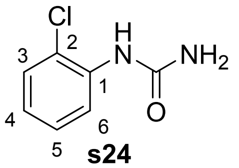
s24

Following *General Procedure 4*, 2-chloroaniline (78 µL, 0.7 mmol, 1.0 eq) was treated with potassium cyanate and acetic acid to afford *urea* **s24** as a white powder (53 mg, 42%). Mp 180-181 °C (Lit.[15] 175 °C); ν_{max} (FTIR) cm^{-1} 3426 (NH$_2$), 3310 (NH), 1648 (C=O), 1538, 1352, 732 (aromatic CH); ^{1}H NMR (400 MHz, DMSO-d$_6$) δ 8.14 (dd, *J* = 1.5, 8.4 Hz, 1H, C(6)*H*), 8.03 (br. s, 1H, C(1)N*H*), 7.39 (dd, *J* = 1.5, 8.0 Hz, 1H, C(3)*H*), 7.23 (dt, *J* = 1.5, 7.8 Hz, 1H, C(5)*H*), 6.95 (dt, *J* = 1.5, 7.8 Hz, 1H, C(4)*H*), 6.38 (br. s, 2H, NH_2); ^{13}C NMR (101 MHz, DMSO-d$_6$) δ 156.0 (*C*=O), 137.2 (*C*(1)), 129.5 (*C*(3)), 127.8 (*C*(5)), 123.0 (*C*(4)), 121.8 (*C*(2)), 121.5 (*C*(6)); *m/z* (ESI$^+$) 171 ([M(^{35}Cl)+H]$^+$, 100%), 173 ([M(^{37}Cl)+H]$^+$, 30%); HRMS (ESI$^+$) $C_7H_7{}^{35}ClN_2ONa^+$, ([M+Na]$^+$) requires 193.0139, found 193.0141.

1-(2-Methoxyphenyl)urea **s25**

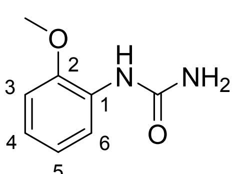
s25

Following *General Procedure 4*, 2-methoxyaniline (83 µL, 0.7 mmol, 1.0 eq) was treated with potassium cyanate and acetic acid to afford *urea* **s25** as a white powder (56 mg, 46%). Mp 141-143 °C; ν_{max} (FTIR) cm^{-1} 3470 (NH$_2$), 3326 (NH), 1664 (C=O), 1530, 1457, 1251, 736 (aromatic CH); ^{1}H NMR (400 MHz, DMSO-d$_6$) δ 8.08 (dd, *J* = 1.9, 7.8 Hz, 1H, C(6)*H*), 7.93 (br. s, 1H, C(1)N*H*), 6.95 (dd, *J* = 1.6, 7.9 Hz, 1H, C(3)*H*), 6.80 - 6.90 (m, 2H, C(4)*H* & C(5)*H*), 6.18 (br. s, 2H, NH_2), 3.83 (s, 3H, CH_3); ^{13}C NMR (101 MHz, DMSO-d$_6$) δ 156.5 (*C*=O), 147.8 (*C*(2)), 129.9 (*C*(1)), 121.5 (*C*(4)), 120.9 (*C*(5)), 118.6 (*C*(6)), 111.0 (*C*(3)), 56.1 (*C*H$_3$); *m/z* (ESI$^+$) 167 ([M+H]$^+$, 100%); HRMS (ESI$^+$) $C_8H_{10}N_2O_2Na^+$, ([M+Na]$^+$) requires 189.0635, found 189.0632.

1-(2-Methoxy-6-methylphenyl)urea **s27**

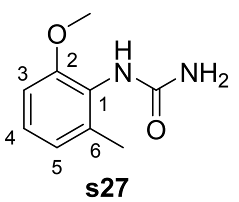
s27

Following *General Procedure 4*, 2-methoxy-6-methylaniline (60 mg, 0.7 mmol, 1.0 eq) was treated with potassium cyanate and acetic acid to afford *urea* **s27** as a white powder (25 mg, 19%). Mp 197.5-199 °C; ν_{max} (FTIR) cm^{-1} 3453 (NH$_2$), 3262 (NH), 1654 (C=O), 1535, 1079 (COC), 767 (aromatic CH); ^{1}H NMR (400 MHz, DMSO-d$_6$) δ 7.32 (br. s, 1H, C(1)N*H*), 7.05 (t, *J* = 7.9 Hz, 1H, C(4)*H*), 6.82 (d, *J* = 8.1 Hz, 1H, C(3)*H*), 6.78 (d, *J* = 7.6

Hz, 1H, C(5)*H*), 5.73 (br. s, 2H, NH_2), 3.75 (s, 3H, OCH_3), 2.15 (s, 3H, C(6)CH_3); ^{13}C NMR (101 MHz, DMSO-d$_6$) δ 157.1 (*C*=O), 154.8 (*C*(2)), 137.0 (*C*(6)), 126.7 (*C*(1)), 126.1 (*C*(4)), 122.5 (*C*(5)), 109.1 (*C*(3)), 55.9 (OCH_3), 18.7 (C(6)CH_3); *m/z* (ESI$^+$) 181 ([M+H]$^+$, 100%); HRMS (ESI$^+$) $C_9H_{12}N_2O_2Na^+$, ([M+Na]$^+$) requires 203.0791, found 203.0796.

N-(2,6-Dichlorobenzyl)propionamide **s29**

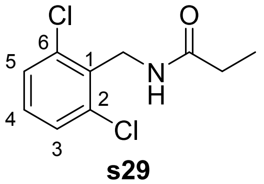

s29

Following *General Procedure 2*, propionyl chloride (52 µL, 0.6 mmol, 1.05 eq) was treated with 2,6-dichlorobenzylamine (100 mg, 0.6 mmol, 1.0 eq) and triethylamine (158 µL, 1.1 mmol, 2.0 eq) to afford *amide* **s29** as a white powder (108 mg, 82%). Mp 148-149 °C; v_{max} (FTIR) cm^{-1} 3281 (NH), 1635 (C=O), 1537, 1434, 778 (C-Cl or aromatic CH), 765 (C-Cl or aromatic CH); ^{1}H NMR (400 MHz, $CDCl_3$) δ 7.26 (d, *J* = 8.0 Hz, 2H, C(3)*H* & C(5)*H*), 7.08 - 7.14 (m, 1H, C(4)*H*), 5.59 (br. s, 1H, N*H*), 4.69 (d, *J* = 5.4 Hz, 2H, C(1)CH_2), 2.15 (q, *J* = 7.6 Hz, 2H, CH_2CH$_3$), 1.09 (t, *J* = 7.6 Hz, 3H, CH$_2$CH_3); ^{13}C NMR (101 MHz, $CDCl_3$) δ 173.2 (*C*=O), 136.2 (*C*(2) & *C*(6)), 133.7 (*C*(1)), 129.6 (*C*(4)), 128.5 (*C*(3) & *C*(5)), 39.2 (C(1)CH_2), 29.5 (CH_2CH$_3$), 9.7 (CH$_2$$CH_3$); *m/z* (ESI$^+$) 232 ([M(^{35}Cl,^{35}Cl)+H]$^+$, 100%), 234 ([M(^{35}Cl,^{37}Cl)+H]$^+$, 73%), 236 ([M(^{37}Cl,^{37}Cl)+H]$^+$, 11%); HRMS (ESI$^+$) $C_{10}H_{11}{}^{35}Cl_2NONa^+$, ([M+Na]$^+$) requires 254.0110, found 254.0105.

N-(2,6-Dichlorobenzyl)isobutyramide **s30**

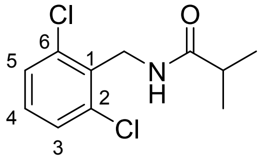

s30

Following *General Procedure 2*, isobutyryl chloride (62 µL, 0.6 mmol, 1.05 eq) was treated with 2,6-dichlorobenzylamine (100 mg, 0.6 mmol, 1.0 eq) and triethylamine (158 µL, 1.1 mmol, 2.0 eq) to afford *amide* **s30** as a white powder (114 mg, 82%). Mp 149-150 °C; v_{max} (FTIR) cm^{-1} 3241 (NH), 2967 (aliphatic CH), 1639 (C=O), 1537, 1435, 779 (C-Cl or aromatic CH), 767 (C-Cl or aromatic CH); ^{1}H NMR (400 MHz, $CDCl_3$) δ 7.25 (d, *J* = 7.8 Hz, 2H, C(3)*H* & C(5)*H*), 7.08 - 7.14 (m, 1H, C(4)*H*), 5.61 (br. s, 1H, N*H*), 4.67 (d, *J* = 5.4 Hz, 2H, CH_2), 2.28 (spt, *J* = 6.9 Hz, 1H, C*H*), 1.08 (d, *J* = 6.9 Hz, 6H, CH(CH_3)$_2$); ^{13}C NMR (101 MHz, $CDCl_3$) δ 176.4 (*C*=O), 136.2 (*C*(2) & *C*(6)), 133.7 (*C*(1)), 129.5 (*C*(4)), 128.5 (*C*(3) & *C*(5)), 39.3 (C(1)CH_2), 35.6 (*C*H), 19.6 (CH(CH_3)$_2$); *m/z* (ESI$^+$) 246 ([M(^{35}Cl,^{35}Cl)+H]$^+$, 100%), 248 ([M(^{35}Cl,^{37}Cl)+H]$^+$, 74%), 250 ([M(^{37}Cl,^{37}Cl)+H]$^+$, 12%); HRMS (ESI$^+$) $C_{11}H_{13}{}^{35}Cl_2NONa^+$, ([M+Na]$^+$) requires 268.0266, found 268.0263.

N-(2,6-Dichlorobenzyl)-2,2,2-trifluoroacetamide **s31**

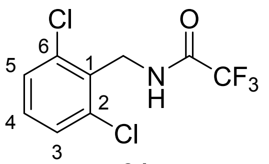

s31

Ethyl 2,2,2-trifluoroacetate (135 µL, 1.1 mmol, 2.0 eq) and triethylamine (79 µL, 0.6 mmol, 1.0 eq) were added to a stirred solution of 2,6-dichlorobenzylamine (100 mg, 0.6 mmol, 1.0 eq) in MeOH (1 mL). The reaction mixture was stirred overnight at room temperature. The solvent was removed *in vacuo* and the crude solid dissolved in EtOAc. The solution was washed with water (2 x 20 mL) and brine (20 mL). The organic layer was dried and the solvent removed *in vacuo* to afford *amide* **s31** as a white powder (103 mg, 67%). Mp 115-117 °C; v_{max} (FTIR) cm^{-1} 3276 (NH), 1693 (C=O), 1167 (C-F), 787 (aromatic CH or C-Cl); ^{1}H NMR (400 MHz, $CDCl_3$) δ 7.33 - 7.41 (m, 2H, C(3)*H* & C(5)*H*), 7.22 - 7.30 (m, 1H, C(4)*H*), 6.50 (br. s, 1H, N*H*), 4.89 (d, *J* = 5.6 Hz, 2H, CH_2); ^{13}C NMR (101 MHz, $CDCl_3$) δ 156.9 (*C*=O), 136.3 (*C*(2) & *C*(6)), 131.4 (*C*(1)), 130.5 (*C*(4)), 128.7 (*C*(3) & *C*(5)), 39.4 (CH_2), (CF_3 not observed); ^{19}F NMR (376 MHz, $CDCl_3$) δ -75.69 (CF_3); *m/z* not observed in (ESI$^+$) or (ESI$^-$); HRMS (ESI$^-$) $C_9H_5{}^{35}Cl_2F_3NO^-$, ([M-H]$^-$) requires 269.9706, found 396.9711.

N-(1-(2-Chlorophenyl)ethyl)acetamide **s32**

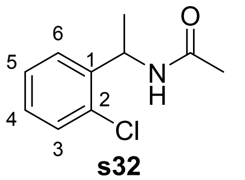

s32

Following *General Procedure 2*, acetyl chloride (40 µL, 0.6 mmol, 1.05 eq) was treated with 1-(2-chlorophenyl)ethanamine (83 mg, 0.5 mmol, 1.0 eq) and triethylamine (149 µL, 1.0 mmol, 2.0 eq) to afford *amide* **s32** as a white powder (92 mg, 87%). Mp 109-110 °C; ν_{max} (FTIR) cm^{-1} 3269 (NH), 1644 (C=O), 1558, 756 (C-Cl or aromatic CH); ^{1}H NMR (400 MHz, $CDCl_3$) δ 7.23 - 7.30 (m, 2H, C(3)*H* & C(6)*H*), 7.10 - 7.19 (m, 2H, C(4)*H* & C(5)*H*), 5.92 (br. s, 1H, N*H*), 5.32 (quin, *J* = 7.3 Hz, 1H, C(1)C*H*), 1.92 (s, 3H, C(=O)CH_3), 1.42 (d, *J* = 7.0 Hz, 3H, CHCH_3); ^{13}C NMR (101 MHz, $CDCl_3$) δ 169.0 (*C*=O), 140.4 (*C*(1)), 132.9 (*C*(2)), 130.2 (*C*(3)), 128.5 (*C*(4)), 127.3 (*C*(6)), 127.1 (*C*(5)), 47.3 (C(1)*C*H), 23.3 (C(=O)*C*H$_3$), 20.9 (CH*C*H$_3$); *m/z* (ESI^+) 198 ([M(^{35}Cl)+H]$^+$, 100%), 200 ([M(^{37}Cl)+H]$^+$, 34%); HRMS (ESI^+) $C_{10}H_{12}{}^{35}ClNONa^+$, ([M+Na]$^+$) requires 220.0500, found 220.0492.

N-(2,4,6-Trimethoxybenzyl)acetamide **s34**

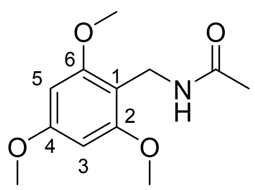

s34

Following *General Procedure 2*, acetyl chloride (40 µL, 0.6 mmol, 1.05 eq) was treated with 2,4,6-trimethoxybenzylamine hydrochloride (125 mg, 0.5 mmol, 1.0 eq) and triethylamine (224 µL, 1.6 mmol, 3.0 eq) to afford *amide* **s34** as a white powder (107 mg, 83%). Mp 153-154 °C; ν_{max} (FTIR) cm^{-1} 3310 (NH), 1643 (C=O), 1598, 1130 (C-O-C); ^{1}H NMR (400 MHz, $CDCl_3$) δ 6.06 (s, 2H, C(3)*H* & C(5)*H*), 5.63 (br. s, 1H, N*H*), 4.37 (d, *J* = 5.0 Hz, 2H, CH_2), 3.75 (s, 6H, C(2)OCH_3 & C(6)OCH_3), 3.74 (s, 3H, C(4)OCH_3), 1.86 (s, 3H, C(=O)CH_3); ^{13}C NMR (101 MHz, $CDCl_3$) δ 169.4 (*C*=O), 160.9 (*C*(4)), 159.3 (*C*(2) & *C*(6)), 106.7 (*C*(1)), 90.6 (*C*(3) & *C*(5)), 55.8 (C(2)O*C*H$_3$ & C(6)O*C*H$_3$), 55.4 (C(4)O*C*H$_3$), 32.4 (*C*H$_2$), 23.5 (C(=O)*C*H$_3$); *m/z* (ESI^+) 240 ([M+H]$^+$, 100%); HRMS (ESI^+) $C_{12}H_{17}NO_4Na^+$, ([M+Na]$^+$) requires 262.1050, found 262.1049.

N-(2-Chloro-4-fluorobenzyl)acetamide **s35**

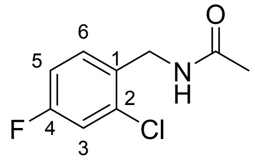

s35

Following *General Procedure 2*, acetyl chloride (40 µL, 0.6 mmol, 1.05 eq) was treated 2-chloro-4-fluorobenzylamine (86 mg, 0.5 mmol, 1.0 eq) and triethylamine (149 µL, 1.0 mmol, 2.0 eq) to afford *amide* **s35** as a white powder (89 mg, 82%). Mp 80-81 °C; ν_{max} (FTIR) cm^{-1} 3285 (NH), 1639 (C=O), 1549, 1491, 903; ^{1}H NMR (400 MHz, $CDCl_3$) δ 7.31 (dd, *J* = 6.1, 8.6 Hz, 1H, C(6)*H*), 7.05 (dd, *J* = 2.6, 8.4 Hz, 1H, C(3)*H*), 6.88 (dt, *J* = 2.6, 8.3 Hz, 1H, C(5)*H*), 5.88 (br. s, 1H, N*H*), 4.40 (d, *J* = 6.1 Hz, 2H, CH_2), 1.94 (s, 3H, CH_3); ^{13}C NMR (101 MHz, $CDCl_3$) δ 170.0 (*C*=O), 161.9 (d, *J* (C-F) = 249.0 Hz, *C*(4)), 134.2 (d, *J* (C-F) = 10.3 Hz, *C*(2)), 131.8 (d, *J* (C-F) = 3.7 Hz, *C*(1)), 131.5 (d, *J* (C-F) = 8.8 Hz, *C*(6)), 116.9 (d, *J* (C-F) = 25.0 Hz, *C*(3)), 114.3 (d, *J* (C-F) = 22.0 Hz, *C*(5)), 41.0 (*C*H$_2$), 23.2 (*C*H$_3$); ^{19}F NMR (376 MHz, $CDCl_3$) δ -112.46 (C(4)*F*); *m/z* (ESI^+) 202 ([M(^{35}Cl)+H]$^+$, 100%), 204 ([M(^{37}Cl)+H]$^+$, 33%); HRMS (ESI^+) $C_9H_{10}{}^{35}ClFNO^+$, ([M+H]$^+$) requires 202.0435, not found.

N-(2,4-Dichlorobenzyl)acetamide **s36**

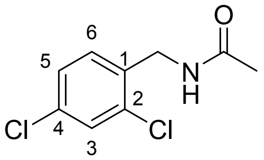

s36

Following *General Procedure 2*, acetyl chloride (40 µL, 0.6 mmol, 1.05 eq) was treated with 2,4-dichlorobenzylamine (72 µL, 0.5 mmol, 1.0 eq) and triethylamine (149 µL, 1.0 mmol, 2.0 eq) to afford *amide* **s36** as a white powder (98 mg, 84%). Mp 88-89 °C (Lit.[16] 88-90 °C); ν_{max} (FTIR) cm^{-1} 3288 (NH), 1639 (C=O), 1543, 848 (aromatic CH), 708 (C-Cl); ^{1}H NMR (400 MHz, $CDCl_3$) δ 7.31 (d, *J* = 2.1 Hz, 1H, C(3)*H*), 7.27 (d, *J* = 8.3 Hz, 1H, C(6)*H*), 7.15 (dd, *J* = 2.1, 8.3 Hz, 1H, C(5)*H*), 5.87 (br. s, 1H, N*H*), 4.40 (d, *J* = 6.1 Hz, 2H,

CH_2), 1.95 (s, 3H, CH_3); ^{13}C NMR (101 MHz, $CDCl_3$) δ 169.3 (*C*=O), 133.7 (*C*(1)), 133.5 (*C*(2)), 133.4 (*C*(4)), 130.5 (*C*(6)), 128.6 (*C*(3)), 126.7 (*C*(5)), 40.3 (*C*H_2), 22.5 (*C*H_3); *m/z* (ESI^+) 218 ([M(^{35}Cl,^{35}Cl)+H]$^+$, 100%), 220 ([M(^{35}Cl,^{35}Cl)+H]$^+$, 73%), 222 ([M(^{37}Cl,^{37}Cl)+H]$^+$, 14%); HRMS (ESI^-) $C_9H_8{}^{35}Cl_2NO^-$, ([M-H]$^-$) requires 215.9988, found 216.0001.

N-(2-Chloro-5-(trifluoromethyl)benzyl)acetamide **s37**

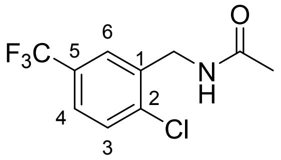

s37

Following *General Procedure 2*, acetyl chloride (40 µL, 0.6 mmol, 1.05 eq) was treated with 2-chloro-5-trifluoromethylbenzylamine (75 µL, 0.5 mmol, 1.0 eq) and triethylamine (149 µL, 1.0 mmol, 2.0 eq) to afford *amide* **s37** as a yellow solid (98 mg, 65%). Mp 86-87 °C; ν_{max} (FTIR) cm^{-1} 3282 (NH), 1644 (C=O), 1326, 1119, 1080; ^{1}H NMR (400 MHz, $CDCl_3$) δ 7.56 (s, 1H, C(6)*H*), 7.41 - 7.43 (m, *J* = 1.2 Hz, 2H, C(3)*H* & C(4)*H*), 5.96 (br. s, 1H, N*H*), 4.49 (d, *J* = 6.1 Hz, 2H, CH_2), 1.98 (s, 3H, CH_3); ^{13}C NMR (101 MHz, $CDCl_3$) δ 170.1 (*C*=O), 137.4 (d, *J* (C-F) = 22.0 Hz, *C*(5)), 136.8 (*C*(1)), 130.0 (*C*(3)), 129.4 (*C*(2)), 126.7 (d, *J* (C-F) = 3.7 Hz, *C*(6)), 125.7 (d, *J* (C-F) = 3.7 Hz, *C*(4)), 123.6 (d, *J* (C-F) = 272.0 Hz, *C*F_3), 41.3 (*C*H_2), 23.2 (*C*H_3); ^{19}F NMR (376 MHz, $CDCl_3$) δ -62.53 (CF_3); *m/z* (ESI^+) 252 ([M(^{35}Cl)+H]$^+$, 100%), 254 ([M(^{37}Cl)+H]$^+$, 31%); HRMS (ESI^-) $C_{10}H_9{}^{35}ClF_3NO^-$, ([M-H]$^-$) requires 250.0252, found 250.0256.

5-Amino-2-phenoxymethyl-oxazole-4-carbonitrile **s39**

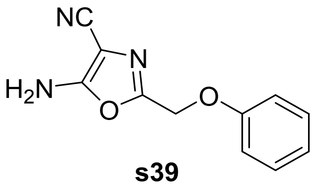

s39

Following *General Procedure 3*, 2-phenoxyacetyl chloride (5.06 mmol) was treated with aminomalononitrile 4-toluenesulfonate to give *oxazole* **s39** (0.80g, 81% yield) as a white solid. Mp 110-112 °C; ^{1}H NMR (300 MHz, $CDCl_3$) δ 4.88 (2H, br. s, NH_2), 4.97 (2H, s, CH_2), 6.95-7.03 (2H, m, ArC*H*), 7.31 (2H, dd, J_1=J_2=8.0 Hz, ArC*H*). ^{13}C NMR (75 MHz, DMSO-d_6) δ 61.2, 82.7, 114.7 (2C), 115.1, 121.5, 129.6 (2C), 147.9, 157.4, 162.6; HRMS (m/ z, +ve, HNESP) [M+H]$^+$ for $C_{11}H_{10}O_2N_3$= Calc. 216.0773, observed. 216.0782; Anal Calcd for $C_{11}H_9N_3O_2.H_2O$: C, 58.92; H, 4.50; Found: C, 59.85, H, 4.85.

5-Amino-2-propyl-oxazole-4-carbonitrile **s40**

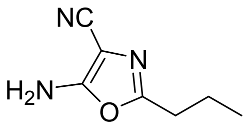

s40

Following *General Procedure 3*, butyryl chloride (0.77 mmol) was treated with aminomalononitrile 4-toluenesulfonate (0.70 mmol) to give *oxazole* **s40** (0.13g, 93% yield) as a light brown oil. ^{1}H NMR (300 MHz, DMSO-d_6) δ 0.92 (3H, t, *J* = 8.0 Hz, CH_3), 1.60 (2H, *sextet*, CH_2), 2.50 (2H, t, *J* = 8.0 Hz, CH_2), 7.57 (2H, br. s, NH_2); ^{13}C NMR (75 MHz, DMSO-d_6) 13.9, 20.0, 29.2, 82.6, 116.3, 153.4, 162.6; HRMS (m/ z, +ve, HNESP) [M+H]$^+$ for $C_7H_{10}ON_3$. Calc. 152.0824; observed. 152.0825.

5-Amino-2-methoxymethyl-oxazole-4-carbonitrile **s46**

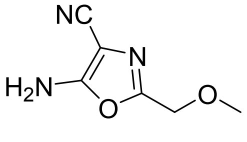

s46

Following *General Procedure 3*, 2-methoxyacetyl chloride (5.06 mmol) was treated with aminomalononitrile 4-toluenesulfonate to give *oxazole* **s46** (0.48g, 69% yield) as a white solid. Mp 120-122 °C; ^{1}H NMR (300 MHz, DMSO-d_6) δ 3.26 (3H, s, OCH_3), 4.28 (2H, s, CH_2), 7.80 (2H, br. s, NH_2). ^{13}C NMR (75 MHz, DMSO-d_6) δ NMR 57.7, 65.0, 82.5, 115.2, 149.1, 162.6; HRMS (m/ z, +ve, HNESP) [(M—CH_3OH)+H]$^+$ for $C_5H_4ON_3$= Calc. 122.0354 observed. 122.0355; Elemental anal. for $C_6H_6N_3O_2$; Calc.; C, 47.06%; H, 4.61%; N, 27.44%; Observed C, 47.22%, H, 4.53%, N, 27.26%.

2-((*tert*-Butyldiphenylsilyl)oxy)acetic acid **s47**

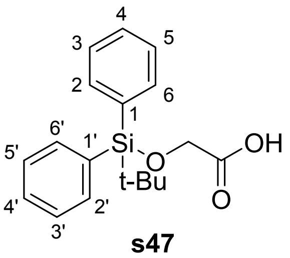

s47

tert-Butylchlorodiphenylsilane (513 µL, 2.0 mmol, 1.5 eq) was added to a stirred solution of glycolic acid (100 mg, 1.3 mmol, 1.0 eq) and imidazole (179 mg, 2.6 mmol, 2.0 eq) in DMF (1.5 mL). The reaction was stirred at room temperature for 2 h before quenching with water (5 mL). The reaction was washed with aq. HCl (0.5 M, 5 mL) and extracted using EtOAc (10 mL). The organic layer was dried and purified by flash column chromatography (7%-60% EtOAc in Cyclohexane). Solvent was removed *in vacuo* to afford *acid* **s47** (244 mg, 59%) as a colourless oil. ν_{max} (FTIR) cm^{-1} 3426, 2856 (OH), 1676 (C=O), 1426, 1112, 703 (aromatic CH); ^1H NMR (400 MHz, DMSO-d_6) δ 12.56 (br. s, 1H, COO*H*), 7.63 - 7.68 (m, 4H, C(2)*H*, C(6)*H*, C(2')*H* & C(6')*H*), 7.41 - 7.51 (m, 6H, C(3)*H*, C(4)*H*, C(5)*H*, C(3')*H*, C(4')*H* & C(5')*H*), 4.19 (s, 2H, C*H*$_2$), 1.02 (s, 9H, C(C*H*$_3$)$_3$); ^{13}C NMR (101 MHz, DMSO-d_6) δ 172.3 (*C*=O), 135.5 (*C*(2), *C*(6), *C*(2') and *C*(6')), 133.1 (*C*(1) and *C*(1')), 130.4 (*C*(4) and *C*(4')), 128.4 (*C*(3), *C*(5), *C*(3') and *C*(5')), 62.0 (*C*H$_2$), 27.0 (C(*C*H$_3$)$_3$), 19.3 (*C*(CH$_3$)$_3$); *m/z* (ESI^+) 315 ($[M+H]^+$, 100%); HRMS (ESI^+) $C_{18}H_{22}O_3SiNa^+$, ($[M+Na]^+$) requires 337.1230, found 337.1231.

2-((*tert*-Butyldiphenylsilyl)oxy)-*N*-(2,6-dimethoxybenzyl)acetamide **s48**

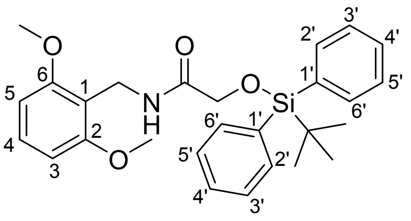

s48

1-[Bis(dimethylamino)methylene]-1H-1,2,3-triazolo[4,5-b]pyridinium 3-oxid hexafluorophosphate (HATU) (242 mg, 0.64 mmol, 1.0 eq) was added to a stirred solution of compound **s47** (200 mg, 0.64 mmol. 1.0 eq) and *N*-methylmorpholine (140 µL, 1.28 mmol, 2.0 eq) in acetonitrile (5 mL). The reaction was stirred at room temperature for 3 h before the addition of 2,6-dimethoxybenzylamine (106 mg, 0.64 mmol, 1.0 eq). The reaction was stirred for a further 1 h. Solvent was removed *in vacuo* and the crude solid redissolved in CH_2Cl_2 (5 mL). The resulting solution was washed with aq. NaOH (10%, 10 mL) and purified by flash column chromatography (7%-60% EtOAc in Cyclohexane). Solvent was removed *in vacuo* to afford *amide* **s48** (171 mg, 58%) as white crystals. Mp 128.5-130 °C; ν_{max} (FTIR) cm^{-1} 3427 (NH), 2854, 1676 (C=O), 1471, 1113 (COC), 774 (aromatic CH), 704 (aromatic CH); ^1H NMR (400 MHz, $CDCl_3$) δ 7.57 - 7.63 (m, 4H, C(2')*H* & C(6')*H*), 7.53 (br. t, *J* = 5.9 Hz, 1H, N*H*), 7.41 - 7.47 (m, 2H, C(4')*H*), 7.32 - 7.38 (m, 4H, C(3')*H* & C(5')*H*), 7.27 (t, *J* = 8.0 Hz, 1H, C(4)*H*), 6.60 (d, *J* = 8.4 Hz, 2H, C(3)*H* & C(5)*H*), 4.68 (d, *J* = 5.9 Hz, 2H, C*H*$_2$NH), 4.12 (s, 2H, C(=O)C*H*$_2$), 3.82 (s, 6H, OC*H*$_3$), 1.08 (s, 9H, C(C*H*$_3$)$_3$); ^{13}C NMR (101 MHz, $CDCl_3$) δ 169.9 (*C*=O), 158.6 (*C*(2) & *C*(6)), 135.3(*C*(2') & *C*(6')), 132.3 (*C*(1')), 130.0 (*C*(4')), 128.9 (*C*(4)), 127.9 (*C*(3') & *C*(5')), 114.2 (*C*(1)), 103.8 (*C*(3) & *C*(5)), 64.0 (C(=O)*C*H$_2$), 55.9 (O*C*H$_3$), 31.7 (*C*H$_2$NH), 26.7 (C(*C*H$_3$)$_3$), 19.1 (*C*(CH$_3$)$_3$); *m/z* (ESI^+) 464 ($[M+H]^+$, 100%); HRMS (ESI^+) $C_{27}H_{34}NO_4Si^+$, ($[M+H]^+$) requires 464.2252, found 464.2250.

Protein Expression and Purification

cDNA encoding PHIP(2) was cloned, expressed and purified as previously described.[17, 18]

AlphaScreen Assay

Assays were performed as described previously[19] with minor modifications from the manufacturer's protocol (PerkinElmer, USA). All reagents were diluted in 25 mM HEPES, 100 mM NaCl, 0.1 % BSA, pH 7.4 supplemented with 0.05 % CHAPS and allowed to equilibrate to room temperature prior to addition to

plates. A 11-point 1:2.0 serial dilution of the ligands was prepared on low-volume 384-well plates (ProxiPlateTM-384 Plus, PerkinElmer, USA), using LabCyte Echo liquid handler. Plates were filled with 12 µL/well with a mix of the assay buffer, biotinylated peptide [H-YSGRGK$_{ac}$GGK$_{ac}$GLGK$_{ac}$GGAK$_{ac}$RHRK(Biotin)-OH] and His-tagged protein to achieve final assay concentrations of 50 nM. Plates were sealed and incubated for a further 30 minutes, before the addition of 8 µl of the mixture of streptavidin-coated donor beads (12.5 µg/ml) and nickel chelate acceptor beads (12.5 µg/ml) under low light conditions. Plates were foil-sealed to protect from light, incubated at room temperature for 60 minutes and read on a PHERAstar FS plate reader (BMG Labtech, Germany) using an AlphaScreen 680 excitation/570 emission filter set. IC_{50} values were calculated in Prism 5 (GraphPad Software, USA) after normalization against corresponding DMSO controls and are given as the final concentration of compound in the 20 µl reaction volume.

Crystallisation

PHIPA(2) Brd was crystallized by mixing 100nl of 13mg/ml protein in 20mM HEPES pH7.5, 500mM NaCl, 5% Glycerol with 100nl of reservoir solution containing 0.1M HEPES pH 7.5, 0.15M Magnesium Chloride, 34% PEG3350. Crystals appeared overnight from sitting drop plates at 4°C. PHIP(2) Brd crystallized in space group $P2_12_12$ with typical unit cell dimensions of a=60Å, b=92Å, c=24Å, corresponding to one PHIP(2) Brd molecule in the asymmetric unit.

Compound Soaking

All compounds of DSPL0 were dissolved in ethylene glycol at a nominal concentration of 400mM. It should be noted that not all compounds could be dissolved at such high concentrations, but in this case we assumed that the solution was saturated. 600nl of each compound was mixed with 600nl of reservoir solution and the mixture was added to the crystals by using a Mosquito® crystallization robot (TTP Labtech). The plates were resealed and incubated for at least 12 hours at 4°C before the crystals were mounted in nylon loops and immediately flash frozen in liquid nitrogen.

Data Collection and Structure Solution

All datasets were collected on beamline I04-1 at the Diamond Light Source. Data were integrated and scaled with XIA2[20] which is part of the Diamond Light Source autoprocessing pipeline.[21] Initial electron density maps were calculated with DIMPLE[22] and inspected with COOT.[23] Once a hit was identified, further rounds of refinement with REFMAC[24] and manual rebuilding with COOT were carried out. ACEDRG[22] was used to generate compound coordinates and restraint files. The quality of the final models was validated with MOLPROBITY.[25] Statistics for data collection and refinement are summarized in supplementary table 4.

Supplemental Table 4

PHIP(2) Crystallographic Data Collection and Refinement Statistics

	Compound	1	**2**	**3**	**4**	**12**	**13**	**14**
	**PDB ID**	5ENB	5ENC	5ENE	5ENF	5ENH	5ENI	5ENJ
**Data Collection**	Space Group	$P\ 2_1 2_1 2$						
Cell Dimensions	a,b,c (Å)	60.30<br>92.17<br>24.25	60.69<br>91.58<br>24.03	60.55<br>91.94<br>24.08	60.81<br>91.96<br>24.09	59.84<br>92.61<br>24.06	60.17<br>91.40<br>23.99	61.01<br>91.26<br>24.13
	α, β, γ (°)	90.00 90.00 90.00						
	Resolution (Å)	50.46 - 1.73 (1.77 - 1.73)*	50.59 - 1.59 (1.63 - 1.59)	50.57 - 1.49 (1.53 - 1.49)	50.72 - 1.37 (1.41 - 1.37)	50.26 - 1.95 (2.00 - 1.95)	50.26 - 1.69 (1.73 - 1.69)	50.72 - 1.63 (1.67 - 1.63)
	Unique Observations	14789 (1045)	18820 (1343)	22778 (1641)	27767 (2973)	10378 (763)	15419 (1122)	16909 (1079)
	Completeness (%)	99.80 (99.90)	99.80 (98.70)	99.40 (99.00)	95.40 (91.40)	99.80 (99.40)	99.20 (99.40)	96.60 (88.40)
	Multiplicity	6.4 (6.7)	6.4 (6.2)	6.4 (6.5)	3.6 (3.6)	6.4 (6.6)	6.5 (6.8)	6.7 (6.3)
	R_{merge}	0.032 (0.746)	0.041 (0.726)	0.036 (0.703)	0.057 (0.524)	0.042 (0.805)	0.034 (0.850)	0.031 (0.839)
	$I/\sigma I$	25.5 (2.6)	21.8 (2.4)	23.3 (2.3)	11.9 (2.2)	22.2 (2.3)	26.1 (2.5)	28.3 (2.2)
	Wavelength	0.9200Å						
**Refinement**	R_{work} / R_{free} (%)	21.3 / 23.6	20.4 / 24.7	20.5 / 23.8	18.1 / 22.0	21.2 / 25.7	22.0 / 26.5	24.0 / 29.8
Number of atoms	protein / ligand / solvent	1004 / 19 / 123	1026 / 21 / 180	1015 / 15 / 123	996 / 20 / 123	991 / 15 / 123	1004 / 14 / 130	1020 / 15 / 111
B-Factors (Å^2)	protein / ligand / solvent	39.0 / 56.9 / 44.3	30.4 / 41.5 / 40.3	29.8 / 46.2 / 40.0	26.6 / 32.2 / 46.8	46.1 / 70.1 / 49.0	33.9 / 38.4 / 41.2	38.0 / 42.3 / 41.0
	R.M.S.D. Bond (Å)	0.016	0.014	0.012	0.014	0.013	0.010	0.018
	R.M.S.D. Angle (°)	1.74	1.67	1.55	1.69	1.55	1.33	1.86
Ramachandran	Favored (%)	99.18	99.18	100	100	99.18	100	100
statistics	Outliers (%)	0	0	0	0	0	0	0

*Highest resolution shell (in Å) shown in parentheses

Design of DSPL1 (Diamond-SGC Poised Fragment Library v1.0)

DSPL1 was designed using the same algorithms as explained above. Fragment libraries already owned by Diamond and the SGC were mined for poised fragments.

Design of DSPL2 (Diamond-SGC Poised Fragment Library v 2.0)

Commercial poised fragments were identified using a workflow prepared using Schrodinger extensions for KNIME (2.6.4 workbench version 2.5.1 v15901 from the Schrodinger 2012 Suite).[26-28] To build DSPL2, the ZINC Fragment-Like library (clogP ≤ 3.5, MW ≤ 250, rotatable bonds ≤ 5) was used.[29,30, 31] The library contained 701,228 compounds in SMILES format. 192,151 compounds were selected based upon their commercial availability from vendors we routinely source compounds from. Rapid elimination of swill (REOS) was performed using sub-structure matching nodes to ensure drug-like characteristics and remove known toxic and promiscuous functionalities.[32] The resulting fragment library contained 41,271 compounds.

Fragments were filtered for poised functional groups using the substructures defined in Figure 4A. By this definition, 28,438 fragments were found to be poised, covering 42,997 reactions with a large number of fragments being found to be poised via multiple reaction types. Each fragment was deconstructed into two synthons and kept if both starting materials were believed to be commercially available. Of the >28,000 fragments, 11,875 poised fragments (42%) were believed to have two, well-represented commercially available starting synthons, covering 15,277 reactions.

Further filters were applied to remove compounds without ring systems,[33] with quaternary nitrogens or containing imines and diacyl hydrazides. In total, 1,347 compounds were removed, leaving a library of 10,448 fragments.

The Knime protocol used to build DSPL1 and DSP2 can be downloaded as zip files with the electronic supplementary material.

The full DSPL1 and DSPL2 libraries can be downloaded in sdf format with the electronic supplementary information.

References

1. J. Spencer, H. Patel, J. Amin, S. K. Callear, S. J. Coles, J. J. Deadman, C. Furman, R. Mansouri, P. Chavatte and R. Millet, *Tetrahedron Lett.*, 2012, 53, 1656-1659.
2. A. B. Pangborn, M. A. Giardello, R. H. Grubbs, R. K. Rosen and F. J. Timmers, *Organometallics*, 1996, 15, 1518-1520.
3. I.-J. Kang, L.-W. Wang, C.-C. Lee, Y.-C. Lee, Y.-S. Chao, T.-A. Hsu and J.-H. Chern, *Bioorg. Med. Chem. Lett.*, 2009, 19, 1950-1955.
4. S. S. Ubarhande, V. G. Thakare and B. N. Berad, *J. Indian Chem. Soc.*, 2010, 87, 1137-1141.
5. G. Vasilev, *Farmatsiya (Sofia)*, 1966, 16, 20-24.
6. G. I. Izvekov and I. E. Skorikov, *Neftepererabotka i Neftekhim.*, 1966, 43-45.
7. L. Doub, L. M. Richardson, D. R. Herbst, M. L. Black, O. L. Stevenson, L. L. Bambas, G. P. Youmans and A. S. Youmans, *J. Am. Chem. Soc.*, 1958, 80, 2205-2217.
8. A. Yahyazadeh, A. R. Aliakbar and F. Habibi, *J. Pharm. Res.*, 2010, 3, 429-431.
9. G. V. Nair, *J. Indian Chem. Soc.*, 1965, 42, 359-366.
10. P.-L. Ho, H.-C. Yang and S.-N. Fang, *Huaxue Xuebao*, 1960, 26, 1-6.
11. M. P. Hay, S. Turcotte, J. U. Flanagan, M. Bonnet, D. A. Chan, P. D. Sutphin, P. Nguyen, A. J. Giaccia and W. A. Denny, *J. Med. Chem.*, 2010, 53, 787-797.
12. D. Karba, *Arch. Pharm. (Weinheim)*, 1967, 300, 844-848.
13. US3376194A, 1968.
14. F. Friedrich and R. Pohloudek-Fabini, *Pharmazie*, 1967, 22, 543-548.
15. P. Gold-Aubert and L. Toribio, *Arch. Sci.*, 1963, 16, 405-410.

16. J.-T. Li, X.-T. Meng and X.-L. Zhai, *Ultrason. Sonochem.*, 2009, 16, 590-592.
17. P. Filippakopoulos, S. Picaud, M. Mangos, T. Keates, J. P. Lambert, D. Barsyte-Lovejoy, I. Felletar, R. Volkmer, S. Muller, T. Pawson, A. C. Gingras, C. H. Arrowsmith and S. Knapp, *Cell*, 2012, 149, 214-231.
18. P. G. Clark, L. C. Vieira, C. Tallant, O. Fedorov, D. C. Singleton, C. M. Rogers, O. P. Monteiro, J. M. Bennett, R. Baronio, S. Muller, D. L. Daniels, J. Mendez, S. Knapp, P. E. Brennan and D. J. Dixon, *Angew. Chem. Int. Ed.*, 2015, 54, 6217-6221.
19. M. Philpott, J. Yang, T. Tumber, O. Fedorov, S. Uttarkar, P. Filippakopoulos, S. Picaud, T. Keates, I. Felletar, A. Ciulli, S. Knapp and T. D. Heightman, *Mol. BioSyst.*, 2011, 7, 2899-2908.
20. G. Winter, *J Appl Crystallogr*, 2010, 43, 186-190.
21. D. Pons, S. Trompet, A. J. M. de Craen, P. E. Thijssen, P. H. A. Quax, M. R. de Vries, R. J. Wierda, P. J. van den Elsen, P. S. Monraats, M. M. Ewing, B. T. Heijmans, P. E. Slagboom, A. H. Zwinderman, P. A. F. M. Doevendans, R. A. Tio, R. J. de Winter, M. P. M. de Maat, O. A. Iakoubova, N. Sattar, J. Shepherd, R. G. J. Westendorp, J. W. Jukema, a. o. b. o. t. PROSPER, WOSCOPS and G. s. groups*, *Heart*, 2011, 97, 143-150.
22. M. D. Winn, C. C. Ballard, K. D. Cowtan, E. J. Dodson, P. Emsley, P. R. Evans, R. M. Keegan, E. B. Krissinel, A. G. W. Leslie, A. McCoy, S. J. McNicholas, G. N. Murshudov, N. S. Pannu, E. A. Potterton, H. R. Powell, R. J. Read, A. Vagin and K. S. Wilson, *Acta Crystallogr D*, 2011, 67, 235-242.
23. P. Emsley, B. Lohkamp, W. G. Scott and K. Cowtan, *Acta Crystallogr D*, 2010, 66, 486-501.
24. G. N. Murshudov, P. Skubak, A. A. Lebedev, N. S. Pannu, R. A. Steiner, R. A. Nicholls, M. D. Winn, F. Long and A. A. Vagin, *Acta Crystallogr D*, 2011, 67, 355-367.
25. V. B. Chen, W. B. Arendall, J. J. Headd, D. A. Keedy, R. M. Immormino, G. J. Kapral, L. W. Murray, J. S. Richardson and D. C. Richardson, *Acta Crystallogr D*, 2010, 66, 12-21.
26. SchrodingerRelease2013-1, *version 9.4*, 2013, Schrodinger, LLC, New York.
27. Small-MoleculeDrugDiscoverySuite2013-1, *version 3.6*, 2013, Schrodinger, LCC, New York.
28. M. R. Berthold, N. Cebron, F. Dill, T. R. Gabriel, T. Kötter, T. Meinl, P. Ohl, C. Sieb, K. Thiel and B. Wiswedel, in *Studies in Classification, Data Analysis, and Knowledge Organization (GfKL 2007)*, Springer, 2007.
29. The ZINC Fragment-Like Library, version 2013-01-26 was downloaded from http://www.zinc.docking.org.
30. J. J. Irwin and B. K. Shoichet, *J Chem Inf Model*, 2005, 45, 177-182.
31. J. J. Irwin, T. Sterling, M. M. Mysinger, E. S. Bolstad and R. G. Coleman, *J. Chem. Inf. Model.*, 2012, 52, 1757-1768.
32. W. P. Walters, M. T. Stahl and M. A. Murcko, *Drug Discovery Today*, 1998, 3, 160-178.
33. W. R. Pitt, D. M. Parry, B. G. Perry and C. R. Groom, *J. Med. Chem.*, 2009, 52, 2952-2963.
